# The Association of Tobacco Control Policies and the Risk of Acute Myocardial Infarction Using Hospital Admissions Data

**DOI:** 10.1371/journal.pone.0088784

**Published:** 2014-02-10

**Authors:** Carmen Jan, Marcos Lee, Reina Roa, Víctor Herrera, Michael Politis, Jorge Motta

**Affiliations:** 1 Gorgas Memorial Institute for Health Studies, Panamá City, Panamá; 2 Ministry of Health, Panamá City, Panamá; Center for Interdisciplinary Research in Biology (CIRB) is a novel Collège de France/CNRS/INSERM, France

## Abstract

**Objective:**

To evaluate the association of a nationwide comprehensive smoking ban (CSB) and tobacco tax increase (TTI) on the risk of acute myocardial infarctions (AMI) in Panama for the period of 2006 – 2010 using hospital admissions data.

**Methods:**

Data of AMI cases was gathered from public and private hospitals in the country for the period of January 1, 2006 to December 31, 2010. The number of AMI cases was calculated on a monthly basis. The risk of AMI was estimated for the pre-CSB period (January 2006 to April 2008) and was used as a reference point. Three post-intervention periods were examined: (1) post-CSB from May 2008 to April 2009 (12 months); (2) post-CSB from May 2009 to November 2009 (7 months); and (3) post-TTI from December 2009 to December 2010 (13 months). Relative risks (RR) of AMI were estimated for each post intervention periods by using a Poisson regression model. Mortality registries for the country attributed to myocardial infarction (MI) were obtained from January 2001 to December 2012. The annual percentage change (APC) of the number of deaths from MI was calculated using Joinpoint regression analysis.

**Results:**

A total sample size of 2191 AMI cases was selected (monthly mean number of cases 36.52±8.24 SD). Using the pre-CSB as a reference point (RR = 1.00), the relative risk of AMI during the first CSB period, the second CSB period and post-TTI were 0.982, 1.049, and 0.985, respectively. The APC of deaths from MI from January 2001 to April 2008 was 0.5%. From January 2001 to June 2010 the APC trend was 0.47% and from July 2010 to December 2012 the APC was –0.3%.

**Conclusions:**

The implementation of a CSB and TTI in Panama were associated with a decrease in tobacco consumption and a reduction of the RR of AMI.

## Introduction

Smoking and secondhand smoke (SHS) have been identified as modifiable risk factors for acute myocardial infarction (AMI) and acute coronary syndrome (ACS). [1], [2] Tobacco smoke affects humans in 2 different ways: by direct smoke while smoking or by the effects of second hand smoke (SHS). SHS is difficult to avoid when there are no regulations for smokers. The risk of AMI decreases when a person stops smoking or when a person stops being exposed to SHS. [3], [4]

Some studies have documented reductions of cardiovascular disease (CVD) with smoking bans, and the reported decreases have ranged from 2% to 17%. [5]–[11] These reductions of CVD seen with smoking bans are thought to come about mainly because of reductions of SHS. Studies in Scotland, England and Ireland have evaluated the effect of nation-wide smoking bans on CVD in more than 50% of the population. [9], [11], [12] Other studies have concluded that smoking bans show reductions in CVD when applied to small populations but this effect is not usually seen when applied to large populations. [13]

In the Netherlands, a study noted a reduction of CVD with the implementation of a smoking ban but, probably due to low compliance, this effect was not sustained. [8] Furthermore, studies in Greece and Portugal have concluded that smoking bans have not produced reductions of cardiovascular disease because of poor compliance with the bans. [14], [15] Moreover, other studies have shown insignificant or no effect in the face of good compliance. In addition, some of these studies conclude that smoking bans may have produced a reduction of AMI admissions, but it cannot be treated with certainty without further research. [6], [10], [16], [17]

A few studies have shown that increases in taxation of tobacco induce changes in the smoking behavior of adults that, in turn, results in reduced cigarette consumption or a decrease in the prevalence of smoking. [18]–[23] One study reported an uncertain relationship between taxation and a reduction of AMI incidence. [24]

In 2005, Panama took measures to control tobacco consumption by prohibiting tobacco advertisement, and mandating the use of health warnings and pictograms on cigarette packages. In January 2008, Panama introduced a nationwide comprehensive smoking ban law (CSB) that took full effect by May 2008. The law banned smoking in all public and private institutions, in closed working and domestic spaces, in areas with a high conglomeration of people and in all public places, with the exception of areas where there is a high flow of natural air circulation. [25] In November 2009, a tobacco tax increase (TTI) went into effect for the entire country raising the retail price of a pack of cigarettes from US $1.84 to US $4.20.[26]

The aim of this study is to evaluate the association of a nationwide CSB and TTI, and the risk of developing AMI during the period of 2006 – 2010 using hospital admissions data.

## Materials and Methods

### Area of The Study and Population

The study area was the Republic of Panama, which has a population of 3 405 813, according to the last census of 2010. [27]

### Study Design

This is a retrospective study of AMI hospital admissions in the Republic of Panama from 2006 to 2010.

### Data Source

Licensed health care professionals reviewed patients’ charts from 13 regional public hospitals, which included the two largest public hospitals, and from the 3 largest private hospitals in the country, for the period of January 1, 2006 to December 31, 2010. We utilized the diagnostic codes of the 10th revision of the International Classification of Diseases (ICD-10). To be considered, one of the following diagnosis needed to be present: AMI and all of its sub-classifications (ICD-10: I21 – I21.9) or unstable angina, which includes coronary syndrome (ICD-10: I20). To be included in the study, the cases identified as AMI had to be substantiated by three diagnostic criteria: symptoms, ECG changes compatible with AMI and abnormal cardiac biomarkers.

We included only those patients admitted between 2006 and 2010 who were ≥ 30 years of age and permanent residents or citizens of the country. We excluded duplicate cases, foreigners, patients younger than 30 years of age and cases in which AMI was a secondary diagnosis.

Initially, we identified a total of 2536 AMI cases that met the three diagnostic criteria, and after applying the exclusion criteria, we ended with a total sample size of 2191 cases.

The mortality registries generated by the National Institute of Statistics and Census (*Instituto Nacional de Estadística y Censo*) were obtained from 2001 to 2012. All registries for which the principal cause of death was MI were included and identified as such, using the codes of the 10th revision of the International Classification of Diseases (ICD-10: I21 – I21.9)

### Statistical Analysis

To analyze the effect of the CSB, we divided it into two post-CSB periods to assess the immediate effect (≤12 months) and the delayed effect (>12 months) of the intervention. We evaluated the effect of TTI as a third post-intervention period.

We utilized a Poisson regression model to evaluate changes in the risk for AMI during the two CSB periods and after the TTI implementation. The dependent variable was the monthly number of hospital admissions of AMI cases.

To estimate the population at risk, we included an offset variable in the Poisson regression model as a log-transformed estimate of the monthly population size of the Republic of Panama who were 30 years of age or older. We obtained the monthly population estimates from 2006 to 2010 by linearly interpolating the annual population provided by the agency responsible for the National Census (*Contraloría General de la República de Panamá*). The population size of the Republic of Panama ≥ 30 years of age was estimated to be 1 406 778 in the first month of the study period, and 1 598 139 in the last month of the study period.

The pre-CSB period (January 2006 to April 2008) was used as a reference point (RR = 1.00). We evaluated three post-intervention periods, the first starting in May 2008, immediately after the start of the CSB and ending on April 2009. The second period, 12 months after the CSB implementation, started in May 2009 and ended on November 2009. The third period started in December 2009, immediately after the TTI implementation and ended on December 2010. A similar regression model was also performed by gender to evaluate the changes in AMI risk for the three post-intervention periods.

To test the robustness of the model, we included additional variables such as monthly mean levels of maximal temperature, ozone, and particulate matter with an aerodynamic diameter <10 µm (PM10) in a second adjusted Poisson regression model. [28]–[31] The electrical company *Empresa de Transmisión Eléctrica, S.A.* provided national monthly maximal temperature values from January 2006 to December 2010. The Institute of Specialized Analysis of the University of Panama provided the monthly mean levels of ozone and the levels of particulate matter with an aerodynamic diameter <10 µm (PM10) from five strategic locations distributed throughout the city of Panama. We used the variable PM 10 to isolate the influence of environmental variables, such as pollution from motor fuel that might have contributed to the incidence of AMI. Environmental control measures were introduced to reduce ozone and other pollutants levels on February 2009 (Executive decree No.5) and went into full effect in May 2009.

A Joinpoint regression model was used to estimate the trend of the monthly number of MI deaths from January 2001 to April 2008 and from January 2001 to December 2012 (Joinpoint Regression Program, Version 4.0.4. of May 2013; Statistical Research and Applications Branch, National Cancer Institute). An annual percentage change was estimated and the points in time at which significant changes in trends occurred were identified. The Monte Carlo permutation test was used to select the simplest model that best fitted the data. [32]. All the settings remained in the default mode.

All other data analyses were calculated using IBM SPSS Statistics, Version 20.0. Armonk, NY: IBM Corp.

### Ethics Statement

At the time of admission, the patients gave written consent for their information to be stored in the hospital’s medical records and for this information to be reviewed by the competent health authorities for research purposes.

This study was approved by the National Bioethics Committee of the Gorgas Memorial Institute for Health Studies and has been conducted in accordance with the ethical standards laid down in the 1965 Declaration of Helsinki and its later amendments. The Institutional Review Board (IRB) approved the review of charts for this research study and waived obtaining a consent form from each patient.

## Results

Of the 2191 AMI cases that fulfilled the inclusion criteria, 1387 (63.3%) occurred in males and 804 (36.7%) in females. The mean age of those diagnosed with AMI was 67±13 and their ages varied from 30 to 98 years. By hospital type, 1864 (85.1%) AMI cases were admitted to public hospitals and 327 (14.9%) to private hospitals. According to their condition at discharge, 1946 (88.8%) were alive, 208 (9.5%) were deceased, and 37 (1.7%) did not have their condition registered at discharge.

Table 1 summarizes the annualized mean values of the covariates used in the adjusted Poisson analysis. The average maximal temperature was almost constant during the time of the study. Annual mean ozone values decreased from 15.92 µg/m^3^ in 2006 to 12.80 µg/m^3^ in 2010. The mean value of PM10 for the time of the study was 39.11 µg/m^3^ with the maximal peak of 54.79 µg/m^3^ in 2008 and the minimum peak of 32.29 µg/m^3^ in 2010.[33].

**Table 1:** Additional predictors of AMI by year, 2006 to 2010.

	2006	2007	2008	2009	2010
Maximal Temperature, °C, mean, (SD)	32.10 (0.57)	31.81 (0.91)	31.68 (0.75)	32.09 (0.56)	32.13 (1.39)
Ozone, µg/m^3^, mean, (SD)	15.92 (7.80)	16.61 (8.85)	12.73 (4.14)	13.06 (4.81)	12.80 (4.71)
PM 10, µg/m^3^, mean, (SD)	35.12 (14.11)	37.57 (19.32)	54.79 (7.75)	35.79 (4.45)	32.29 (11.48)

Abbreviations: °C, degree Celsius; µg/m^3^, microgram per cubic meter; PM10, particulate matter <10 µm; SD, standard deviation.

As compared to the pre-CSB reference risk of AMI (RR = 1.00), the unadjusted Poisson regression model estimated a RR decrease of 1.5% or a RR = 0.985 (0.970–1.000), p = 0.045 in the first 12 months of the CSB. However, during the following seven months (second CSB period), the RR was 1.048 (1.021–1.075), p = 0.000. After the TTI came into effect, the RR was 0.985 (0.972–0.998), p = 0.028, over a 13 months period (Table 2). The likelihood-ratio chi-square test of this model, when compared to the null model, showed a *χ* ^2^ = 24 998, df = 3, p = 0.000.

**Table 2:** Unadjusted Poisson regression model of AMI cases by gender and study periods.

	*T O T A L*				*M A L E S*				*F E M A L E S*			
*Period*	*RR*	*Lower 95% Cl*	*Upper 95%Cl*	*p-Value*	*RR*	*Lower 95% Cl*	*Upper 95%Cl*	*p-Value*	*RR*	*Lower 95% Cl*	*Upper 95%Cl*	*p-Value*
***Pre-CSB***	1.000*				1.000*				1.000*			
***Post-CSB 1***	0.985	0.970	1.000	0.045	0.996	0.978	1.014	0.655	0.964	0.938	0.990	0.007
***Post-CSB 2***	1.048	1.021	1.075	0.000	1.030	0.996	1.066	0.089	1.074	1.032	1.117	0.000
***Post-TTI***	0.985	0.972	0.998	0.028	0.992	0.976	1.009	0.351	0.972	0.950	0.995	0.017

Abbreviations: RR, relative risk; CI, confidence interval; CSB, comprehensive smoking ban; TTI, tobacco tax increase.

*RR for each period is given in reference to the pre-CSB period.

The adjusted Poisson regression model showed similar results (Table 3). As compared to the pre-CSB reference risk period of AMI (RR = 1.00), the adjusted Poisson regression model estimated a RR decrease of 1.8% or a RR = 0.982 (0.967–0.997), p = 0.023, in the first 12 months of the CSB. However, during the following seven months (second CSB period), the RR was 1.049 (1.022–1.077), p = 0.000. After the TTI came into effect, the RR was 0.985 (0.971–0.999), p = 0.041, during 13 months. The likelihood-ratio chi-square test of this model, when compared to the null model, showed a *χ* ^2^ = 36 448 df = 6, p = 0.000.

**Table 3:** Adjusted Poisson regression model of AMI cases by gender and study periods.

	T O T A L				M A L E S				F E M A L E S			
Period	RR	Lower 95% Cl	Upper 95%Cl	p-Value	RR	Lower 95% Cl	Upper 95%Cl	p-Value	RR	Lower 95% Cl	Upper 95% Cl	p-Value
Pre-CSB	1.000*				1.000*				1.000*			
Post-CSB 1	0.982	0.967	0.997	0.023	0.995	0.976	1.014	0.587	0.959	0.933	0.985	0.003
Post-CSB 2	1.049	1.022	1.077	0.000	1.032	0.997	1.069	0.069	1.075	1.033	1.119	0.000
Post-TTI	0.985	0.971	0.999	0.041	0.994	0.977	1.012	0.530	0.969	0.946	0.993	0.012

Abbreviations: RR, relative risk; CI, confidence interval; CSB, comprehensive smoking ban; TTI, tobacco tax increase.

*RR for each period is given in reference to the pre-CSB period.

The Joinpoit regression analysis from January 2001 to April 2008 revealed an 0.5% (p<0.05) APC trend of deaths from MI. From January 2001 to June 2010 the APC trend was 0.47% (p<0.05), with a trend change of –0.3% starting in July 2010 to December 2012 but this change did not reach statistical significance.

We did not find evidence of stable seasonality at the 0.1 % level (F-value  = 1.041), nor evidence of moving seasonality at the 5% level (F-value  =  1.367) of the incidence of AMI cases during the study period utilizing a census X-12 ARIMA time series analysis. Furthermore, we did not find evidence of a linear trend in the AMI case series (p = 0.413).

## Discussion

For Panama, this is the first study that has evaluated the effect of a CSB and a TTI on the risk of myocardial infarction.

The first year of the CSB implementation was associated with an AMI RR reduction of –1.5% in the unadjusted and –1.8% in the adjusted Poisson regression models. Although we do not have information on the number of AMI cases prior to the pre-CSB period of 2006 to 2008, the increasing mortality trend from MI seen from January 2000 to 2008 suggests that there was not a preexisting decreasing trend of AMI cases that could have accounted for the decrease in risk seen in the first 12 months of the CSB.

As in our study, several published studies have found reductions of cardiovascular morbidity and mortality associated to smoking bans, [34]–[37] while others have found insignificant or no effects of smoking bans even in the face of good compliance. [6], [8], [10], [16], [17], [38], [39]. This lack of effect of smoking bans on cardiovascular events could be attributed to an increase in the number of active smokers in the face of a decrease in exposure of passive smoking produced by the bans.

After the first year of the CSB, there was a transient increase of the RR of AMI. This increase might have resulted from random background changes in the number of AMIs or produced by the way the CSB period was partitioned or by a lack in the effect of the CSB.

Moreover, some studies have concluded that lower compliance or reduced reinforcement may decrease the effect of smoking bans. Although we did not measure yearly estimates of active smoking prevalence in our sample, the estimates of prevalence of active smoking from 2007 and 2010 indicate that there was a decreased from 9% to 6.4%.[40], [41] The most recent national survey of tobacco consumption, the Global Adult Tobacco Survey of Panama (GATS) estimated the national prevalence of smoking to be 6%. [42] In addition, cigarette importation decreased from 729 243 kg in 2007, to 657 657 kg in 2008, to 615 288 kg in 2009 and almost halved to 344 185 kg in 2010, reinforcing the finding that there was a decrease in smoking prevalence from 2007 to 2010. [43], [44]

After the implementation of the TTI at the end of 2009, the AMI RR showed similar numbers as the first post-CSB with no further reduction of the RR in both, unadjusted and adjusted Poisson regression models. Some studies have shown that an increase of US $1 has resulted in a 6 – 8% reduction in smoking and that there is an association between an increase in the taxation of tobacco and a reduction in the number of premature deaths. [18], [45]

The sustained low smoking prevalence and the drop of cigarette importation during the TTI suggests a connection between price sensitivity and smoking behavior. This in turn, may be associated with a reduction of AMI incidence and risk. [18] Supporters of tobacco tax increases indicate that increasing cigarette taxes may lead to better health because it induces cessation of smoking and also prompts savings in medical care costs. [20]

The effects seen with the unadjusted and the adjusted Poisson regression analysis showed a small RR differences and this has been seen in other studies. [8], [34] When evaluated by gender, women appear to have received a greater benefit of the CBS and the TTI than males. The possible explanations for this gender difference might be related to a higher likelihood that females would follow smoking restrictions at home than males [46] or to a possible higher sensitivity of females to the deleterious effects of tobacco smoke. [47]

We cannot ascertain which measure, the CSB or the TTI, might have had the greatest impact in reducing the risk of AMI. The CSB of 2008 probably had an effect mostly, but not only, by reducing passive smoking. The TTI of 2010 probably had an effect primarily through a decrease of active smoking. Although the prevalence of active smoking estimate for 2010 and the marked decrease in cigarette importation documented in 2010 suggest an impact of the TTI on active smoking, the decrease in importation cigarettes seen from 2007 to 2009 suggests that CSB also had an effect on active smoking.[43]

Finally, from 2007 to 2012, there were no other national initiatives to reduce cardiovascular risk factors in Panama with an equivalent level of organization or government support that could explain the observed risk reduction of AMI or the recent reduction in the trend of MI deaths noted in the country.

The limitations of this study were the following: we were not able to review the hospital records of all the hospitals in Panama and this led to an underestimation of the national yearly number of AMIs. However, the hospitals included in the study were the largest healthcare facilities in the country.

The reason why not all hospitals and clinics were included in the study was because many of them lacked the analysis of cardiac enzymes required as diagnostic criteria and many did not have structured medical records systems from where we could identify patients admitted with the diagnosis of AMI or verify if the diagnosis of AMI was primary or secondary. Cases that did not fulfill the diagnostic criteria, those which were given other diagnosis, and those who died outside of a hospital facility also diminished the number of cases for review.

Small variations in the AMI admissions in the catchment areas hospitals could have induced an apparent effect of the ban. These variations might have occurred because of random changes in the number of admissions. Nevertheless, we believe that the fall in the trend of MI-induced mortality gives indirect support to our finding that the CSB and the TTI were associated to a reduction in the number of AMI’s

We did not have yearly estimates of the prevalence of smoking in the nation, nor did we have evaluations of compliance to the CSB. Nevertheless, there is evidence that from 2007 to 2010 the number of active smokers in the nation decreased approximately 9% to 6.4% and there was a large drop in the importation of tobacco. [40]–[42]

## Conclusions

The results of this national-scale study suggest that the risk of AMI may be sensitive to tobacco-control measures such as a CSB and a TTI.

In many countries, the recent introduction of tobacco control policies represents a revolutionary event in the history of public health. Laws banning tobacco consumption are associated mainly with decrease exposure of passive smoke. This, in turn, is associated with multiple beneficial health outcomes. The reduction of AMI events seen in the first twelve months of our study may be an example of one of these beneficial outcomes. The TTI appears to have been an effective intervention to achieve reductions in tobacco consumption and may also be an effective public health intervention to reduce the risk of suffering AMI.

## Supporting Information

Dataset S1 **Census 2010, Republic of Panama.**
(XLS)

Document S1 **ENSCAVI 2007 - Tobacco Consumption Prevalence in Panama.**
(PDF)

Document S2 **Global Adult Tobacco Survey in Panama 2013 - Fact Sheet.**
(PDF)

Document S3 **Law 13, 2008 - Smoking ban in Panama.**
(PDF)

Document S4 **Law 69, 2009 - Tobacco Tax Increase in Panama.**
(PDF)

Document S5 **PREFREC 2010-Tobacco Consumption Prevalence in Panama.**
(PDF)

Document S6 **Tobacco Importation in Panama Report.**
(PDF)

Document S7 **Air Pollution Emissions Control in Panama.**
(PDF)

Document S8 **IRB approval letter.**
(JPG)
