# Supplementary material for: The Association of Tobacco Control Policies and the Risk of Acute Myocardial Infarction Using Hospital Admissions Data
Source: PLoS One. 2014 Feb 10;9(2):e88784. doi: 10.1371/journal.pone.0088784 (PMC3919809; doi:10.1371/journal.pone.0088784)

Consumo de Tabaco, Alcohol y Drogas

<u>Tabaco</u>

Prevalencia de Vida

Los entrevistados (n=25748) indicaron haber ***masticado o fumado tabaco o productos del tabaco***, alguna vez en su vida en un 22.8%. En las **áreas urbanas** se reflejó que de las 14072 personas que respondieron a esta pregunta el 37.2% eran **<u>hombres</u>** y el 62.8% eran **<u>mujeres</u>,** de las cuales 3023 respondieron haber consumido productos de tabaco alguna vez en su vida, para una prevalencia de 21.5%. De las 5879 personas que manifestaron este consumo 1 de cada 2 (51.4%) eran residentes en el **área urbana**, con una prevalencia para **<u>hombres</u>** de 38.2% y en **<u>mujeres</u>** de 11.6%. La prevalencia de vida en las **áreas rurales** fue del 24% de los 8498 residentes en dichas áreas para una prevalencia en **<u>hombres</u>** de 41.4% y en **<u>mujeres</u>** de 10.7%. Para las **áreas indígenas** la prevalencia de vida fue de 25.8% de los 3178 entrevistados, alcanzando un 45.4% en **<u>hombres</u>** y 10.9% en **<u>mujeres</u>**.

MAPA No.6

POBLACION DE 18 AÑOS Y MAS POR HABER MASTICADO O FUMADO TABACO O PRODUCTOS DE TABACO ALGUNA VEZ EN SU VIDA SEGUN DISTRITO. REPUBLICA DE PANAMA. AÑO: 2007. (n = 25748)

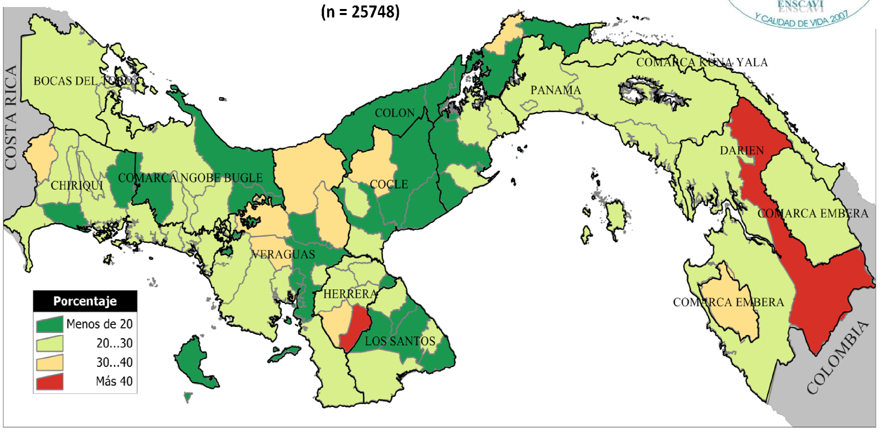

Los distritos en que se reportaron las mayores prevalencia de vida para el consumo de tabaco fueron: Los Pozos y Pinogana con cifras superiores al 40%, seguidos Renacimiento, Cañazas, La Mesa, Soná, Calobre, La pintada, Portobelo y Sambú, con cifras entre el 30%y el 40%.

Fuente: Encuesta Nacional de Salud y Calidad de Vida. República de Panamá. Año: 2007.

Edad de Inicio

En los 5879 entrevistados que admitieron <u>haber fumado alguna vez</u>, la edad de su primera experiencia se concentró entre los <u>18 a los 24 años</u> con un 43.2%, de <u>13 a 15 años</u> con un 18.1% y del grupo de <u>16 a 17 años</u> con 15.9%; en un 7.9% la edad de inicio estuvo <u>antes de los 12 años</u> y el 15.1% admitió que el inicio del consumo fue con <u>25 y más años</u> de edad. El rango de edad fluctuó entre 5 y 64 años, con una **edad promedio** de 19.7 años, una **mediana** de 19.3 años y una **moda** de 18 años. El promedio en **<u>hombres</u>** fue de 18.8 y en **<u>mujeres</u>** de 21.9 años, en tanto que la **mediana** fue de 18.7 y 20.6 años para **<u>hombres</u>** y **<u>mujeres</u>**, respectivamente. Los que <u>nunca han fumado</u> sumaron 19869, alcanzando al 77.2% de la muestra de 25748 personas.

GRAFICA No.8

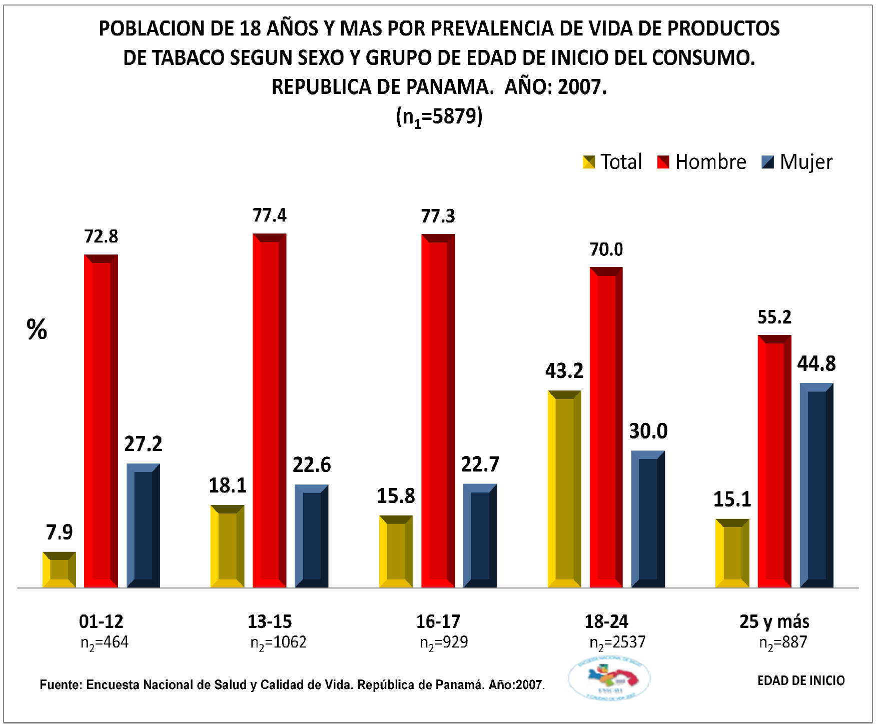

En las **áreas urbanas** de los 3023 que consumieron alguna vez en su vida, cerca de 4 de cada 10 (44.2%) inició el consumo entre los 18 y 24 años de edad, seguido del grupo de menores de edad con un 41.2% y del de 25 años y más con un 14.7%. De los 1998 **hombres** que consumieron productos de tabaco alguna vez en su vida, cuatro de cada 10 (44.2%) tenía entre 18 y 24 años al momento de iniciar el consumo; cerca de 5 de cada 10 (45.7%) eran menores de edad y 11.5% mayores de 24 años. En el caso de las **mujeres** de las 1025 entrevistadas, el 46.8% inició el consumo de productos de tabaco entre los 18 y 24 años; 1 de cada 3 (32.3%) lo hizo siendo menor de edad y 1 de cada 5 (20.9%) con 25 años y más. La **edad promedio** de 19.6 años, una **mediana** de 19.3 años. El **promedio** en **hombres** fue de 18.7 y en **mujeres** de 21.2 años, en tanto que la **mediana** fue de 18.6 y 20.5 años para **hombres** y **mujeres**, respectivamente.

En las **áreas rurales** de los 2037 que consumieron alguna vez en su vida, cerca de 4 de cada 10 (42.1%) inició el consumo entre los 18 y 24 años de edad, seguido del grupo de menores de edad con un 42.6% y del de 25 años y más con un 15.3%. De los 1523 **hombres** que consumieron productos de tabaco alguna vez en su vida. Cuatro de cada 10 (42.6%) tenía entre 18 y 24 años al momento de iniciar el consumo; cerca de 5 de cada 10 (45.0%) eran menores de edad y 12.3% mayores de 24 años. En el caso de las **mujeres** de las 514 entrevistadas, el 40.5% inició el consumo de productos de tabaco entre los 18 y 24 años; cerca de 1 de cada 3 (35.4%) lo hizo siendo menor de edad y 1 de cada 4 (24.1%) con 25 años y más. La **edad promedio** de 19.8 años, una **mediana** de 19.1 años. El **promedio** en **hombres** fue de 18.9 y en **mujeres** de 22.5 años, en tanto que la **mediana** fue de 18.7 y 20 años para **hombres** y **mujeres**, respectivamente.

En las **áreas indígenas** de los 819 que consumieron alguna vez en su vida, cerca de 4 de cada 10 (42.1%) inició el consumo entre los 18 y 24 años de edad, seguido del grupo de menores de edad con un 41.8% y del de 25 años y más con un 16.1%. De los 623 **hombres** que consumieron productos de tabaco alguna vez en su vida. Cuatro de cada 10 (43.7%) tenía entre 18 y 24 años al momento de iniciar el consumo; cerca de 4 de cada 10 (44.6%) eran menores de edad y 11.7% mayores de 24 años. En el caso de las **mujeres** de las 196 entrevistadas, el 37.2% inició el consumo de productos de tabaco entre los 18 y 24 años; cerca de 1 de cada 3 (32.7%) lo hizo siendo menor de edad y cerca de 1 de cada 3 (30.1%) con 25 años y más. La **edad promedio** de 20.1 años, una **mediana** de 19.2 años. El **promedio** en **hombres** fue de 19 y en **mujeres** de 23.9 años, en tanto que la **mediana** fue de 18.8 y 19.3 años para **hombres** y **mujeres**, respectivamente.

La ***edad de inicio del consumo de tabaco*** reflejó que no hay diferencias estadísticamente significativas en ninguna de las áreas, al comparar el grupo de edad de menores de 12 años con los grupos de 13 a 15 años, de 16 a 17 años, de 18 a 24 años y con los de 25 años y más, excepto cuando se trata de la comparación **rural** – **urbana**, para los grupos de 16 a 17 años y de 18 a 24 años. Al reagrupar los grupos de edad y efectuar el análisis comparando a los entrevistados que empezaron a fumar con 15 años o menos y los que iniciaron con 16 y más años, se encontraron diferencias estadísticamente significativas únicamente en la relación **rural** – **urbana**, lo que refleja mayor riesgo de inicio del consumo a temprana edad en la población **rural**.

Las pruebas de significancia estadística evidenciaron que no existen diferencias estadísticamente significativas en la edad de inicio del consumo de tabaco entre **hombres y mujeres** para ninguno de los grupos de edad antes descritos (Cuadro N°7).

CUADRO Nº7. ESTIMADORES DE RIESGO, LIMITES DE CONFIANZA Y PRUEBA DE SIGNIFICANCIA POR AREAS SEGUN EDAD DE INICIO DEL CONSUMO DE TABACO. REPUBLICA DE PANAMA. AÑO: 2007.

Variable	AREA		RESULTADOS		
Edad de inicio del consumo de tabaco	Rural	Urbano	OR	LC	p
Menos de 13 años	200	205	1.2	0.94 - 1.53	0.1452013
13-15	406	499			
Menos de 13 años	200	205	2.01	1.57 - 2.59	0.000
16-17	262	541			
Menos de 13 años	200	205	1.52	1.22 - 1.89	0.0001356
18-24	857	1335			
Menos de 13 años	200	205	1.39	1.08 -1.78	0.0100888
25 y mas	312	443			
Edad de inicio del consumo de tabaco	Indígena	Urbano	OR	LC	p
Menos de 13 años	59	205	0.91	0.64 - 1.30	0.6694545
13-15	157	499			
Menos de 13 años	59	205	1.24	0.86 -1.78	0.2710356
16-17	126	541			
Menos de 13 años	59	205	1.11	0.80 - 1.54	0.5530145
18-24	345	1335			
Menos de 13 años	59	205	0.97	0.67 - 1.39	0.9152652
25 y mas	132	443			
Edad de inicio del consumo de tabaco	Indígena	Rural	OR	LC	p
Menos de 13 años	59	200	0.76	0.53 - 1.09	0.1443087
13-15	157	406			
Menos de 13 años	59	200	0.61	0.42 - 0.89	0.0097378
16-17	126	262			
Menos de 13 años	59	200	0.73	0.53 - 1.02	0.0634234
18-24	345	857			
Menos de 13 años	59	200	0.7	0.48 - 1.01	0.0560919
25 y mas	132	312			

Variable	AREA		RESULTADOS		
Edad de inicio del consumo de tabaco	Rural	Urbano	OR	LC	p
15 o menos	606	704	1.39	1.23 – 1.59	0.000
16 y más	1431	2319			
Edad de inicio del consumo de tabaco	Indígena	Urbano	OR	LC	p
15 o menos	216	704	1.18	0.98 – 1.41	0.0736
16 y más	603	2319			
Edad de inicio del consumo de tabaco	Indígena	Rural	OR	LC	p
15 o menos	216	606	0.85	0.7 – 1.02	0.7899
16 y más	603	1431			

Fuente: Encuesta Nacional de Salud y Calidad de Vida. República de Panamá. Año: 2007.

Prevalencia Actual

GRAFICA No.9

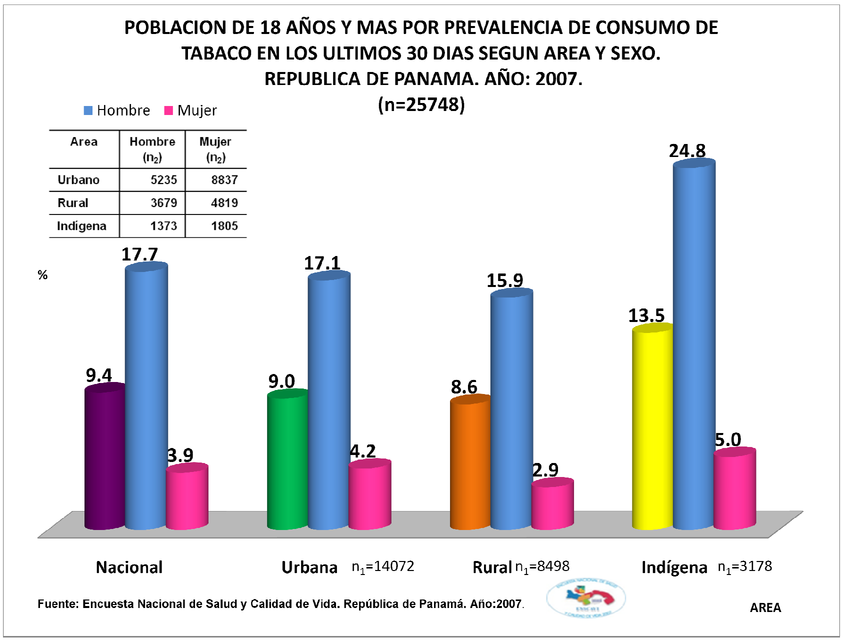

De los que manifestaron ***haber fumado alguna vez en su vida*** (5879), el 58.8% negó el consumo en el último mes, para una prevalencia actual de 9.4%, siendo la prevalencia en **hombres** de 17.7% y en **mujeres** de 3.9%. De los fumadores actuales (2421), cerca de 1 de cada 2 fumadores actuales lo hace 7 días o menos. De un total de 1819 **hombres** que consumen productos de tabaco en la actualidad, 1 de cada dos consumió estos productos de 1 a 7 días, mientras que cerca de 2 de cada 7 (27.3%) lo hizo de 22 a 31 días y uno de cada 5 consumieron de 8 a 21 días. Cifras similares de consumo se presentan al hacer el diferencial por sexo en el grupo de fumadores que consumen de 22 a 31 días.

De los 3023 que respondieron haber consumido productos de tabaco alguna vez en su vida y que residían en el **área urbana,** 1760 no había fumado en el último mes (50.9%). Un total de 1263 de los 14072 entrevistados residentes en **áreas urbanas** fumó en el último mes para una prevalencia actual de 9%, con una prevalencia en **hombres** de 17.0% y en **mujeres** de 4.2%. El mayor consumo se concentró en el grupo de fumadores que consumen de 1 a 7 días en el último mes con un 49.1%, seguido de los consumidores de 22 a 31 días con el 29.3%.

De los 2037 que respondieron haber consumido productos de tabaco alguna vez en su vida y que residían en el **área rural,** 1309 no había fumado en el último mes (64%). Un total de 728 de los 8498 entrevistados residentes en **áreas rurales** fumó en el último mes para una prevalencia actual de 8.6%, con un diferencial de 15.9% en **hombres** y de 2.9% en **mujeres**. En general en el área rural el consumo se concentró de 1 a 7 días con el 46.7%, seguido de 22 a 31 días de consumo en el último mes con 28.4%.

De los 819 que respondieron haber consumido productos de tabaco alguna vez en su vida y que residían en el **área indígena,** 389 no había fumado en el último mes (47.4%). Un total de 430 de los 3178 entrevistados residentes en **áreas indígenas** fumó en el último mes, para una prevalencia actual de 13.5%, con un diferencial por sexo de 24.8% en **hombres** y 5% en **mujeres**. Para esta área, también el consumo se concentró de 1 a 7 días con el 56.3%, seguido de 22 a 31 días de

consumo en el último mes con 19.5%. El comportamiento en los patrones de consumo fue similar entre **hombres** y **mujeres**, aunque en el consumo de 22 y más días la relación **hombre-mujer** fue de 1.2:1.

El ***consumo actual de tabaco en los últimos 30 días*** fue 2 veces mayor en las **áreas indígenas** que en las **rurales** y 1.6 veces mayor que en las **urbanas**, con diferencias estadísticamente significativas. En relación con al sexo, no se identificaron diferencias estadísticamente significativas (Cuadro N°8).

CUADRO Nº8. ESTIMADORES DE RIESGO, LIMITES DE CONFIANZA Y PRUEBA DE SIGNIFICANCIA POR AREAS SEGUN CONSUMO DE TABACO EN LOS ULTIMOS 30 DIAS. REPUBLICA DE PANAMA. AÑO: 2007.

Variable	AREA		RESULTADOS		
Consumo de tabaco en los últimos 30 días	Rural	Urbano	OR	LC	p
Sí	698	1227	0.76	0.68 - 0.86	0.0000084
No	1309	1760			
Consumo de tabaco en los últimos 30 días	Indígena	Urbano	OR	LC	p
Sí	422	1227	1.56	1.33 - 1.82	0.000
No	389	1760			
Consumo de tabaco en los últimos 30 días	Indígena	Rural	OR	LC	p
Sí	422	698	2.03	1.72 - 2.41	0.000
No	389	1309			

Fuente: Encuesta Nacional de Salud y Calidad de Vida. República de Panamá. Año: 2007.

Exposición al Humo de Tabaco de Segunda Mano

Al indagar sobre la ***exposición al humo de tabaco de segunda mano (EHTSM)*** en **la casa** del entrevistado, cerca de 1 de cada 5 (19.3%), indicaron que han fumado en su presencia en los últimos 30 días; en tanto que en **el trabajo** el 26.4% indicó estar expuesto. En el caso de los **centros recreativos** esta respuesta fue afirmativa en el 24.3%. Hubo un porcentaje de no respuesta de 3.4%, 18.9%, 4.2%, en casa, en el trabajo y en centros recreativos, respectivamente. El porcentaje de exposición fue 1.1 veces mayor en los **trabajos** que en los **centros recreativos** y 1.4 veces mayor en el **trabajo** que en la **casa**.

La exposición al humo de tabaco de segunda mano en **casa,** fue expresada en el 18.9% y 18.4% de los entrevistados de **áreas urbanas y rurales**, respectivamente. Cerca de uno de cada 4 (23.4%) entrevistados **indígenas** reportó EHTSM en sus casas. El comportamiento de esta variable por sexo indica que los **hombres rurales e indígenas** reportaron ligeramente mayor exposición en las casas que las **mujeres**, a excepción de las **áreas urbanas**.

Cuando se trata de la exposición al humo de tabaco de segunda mano en el **trabajo** el 12.5% en el **área urbana**, el 11.8% y el 5.3% en las **áreas rurales e indígenas** respectivamente, reportó este problema de salud pública. Cuando se trata de los **hombres** cerca de 6 de cada 10 (63.6%) residentes **urbanos** y cerca 8 de cada 10 (78.5%) residentes rurales manifestaron estar expuestos en su **trabajo**, siendo esta exposición 1.3 veces menor en las **áreas indígenas** con respecto a las **urbanas**. Las **mujeres** residentes del **área urbana** estuvieron 1.9 veces más expuestas que las de las **áreas indígenas** y 1.7 veces más que en las **rurales**.

GRAFICA No.10

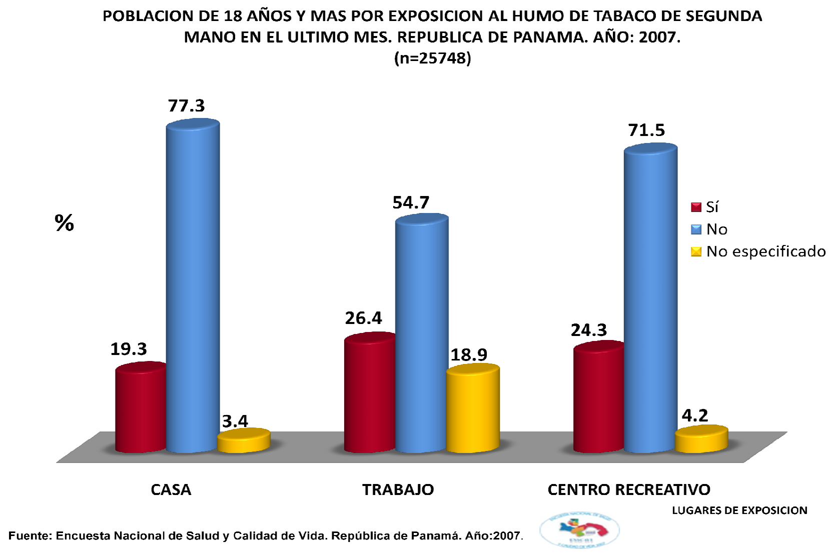

En el caso de los **centros de recreación** cerca de 1 de cada 4 (26.5%) y 1 de cada 5 (20.6%) de las **áreas urbanas y rurales**, respectivamente, reportaron exposición al HTSM. **Las áreas indígenas** se destacaron con el 24.4%. La diferencia entre los **hombres** indica que uno de cada tres (32.8%) de los **urbanos** y 31.4% de los **indígenas** reportaron exposición a esta sustancia en centros recreativos, en tanto que a nivel **rural** 1 de cada 5 (20.6%) se reportó como expuesto. Por su parte el 22.8% de las **mujeres** **urbanas** y el 19.1% de las **indígenas** estuvieron expuestas, frente a un 15.5% de las **mujeres** **rurales**.

Hay más riesgo de ***exposición al humo de tabaco*** en las casas de la población **indígena** con relación a las residencias **urbanas y rurales**. Los centros laborales en las **áreas indígenas** son un factor protector con relación a la exposición al humo de tabaco, al compararlos los centros laborales de las **áreas urbanas** y **rurales**, con diferencias estadísticamente significativas. Los centros de recreación de las **áreas rurales** e **indígenas** son factores protectores cuando se comparan con los de las **áreas urbanas**, sin embargo en los centros de recreación **indígenas** hay mayor riesgo a la exposición al humo de tabaco, con respecto a los de las **áreas rurales**, con diferencias estadísticamente significativas (Cuadro Nº9).

CUADRO Nº9. ESTIMADORES DE RIESGO, LIMITES DE CONFIANZA Y PRUEBA DE SIGNIFICANCIA POR AREAS SEGUN LUGARES DE EXPOSICION AL HUMO DE TABACO. REPUBLICA DE PANAMA. AÑO: 2007.

Variable	AREA		RESULTADOS		
Casa	Rural	Urbano	OR	LC	p
Sí	1561	2654	0.97	0.90 -1.04	0.3985853
No	6632	10936			
Casa	Indígena	Urbano	OR	LC	p
Sí	742	2654	1.3	1.19 - 1.43	0.000
No	2346	10936			
Casa	Indígena	Rural	OR	LC	P
Sí	742	1561	1.34	1.22 - 1.49	0.000
No	2346	6632			
Trabajo	Rural	Urbano	OR	LC	p
Sí	1005	1763	0.95	0.87 - 1.03	0.2169111
No	6840	11377			
Trabajo	Indígena	Urbano	OR	LC	p
Sí	168	1763	0.44	0.37 - 0.51	0.000
No	2491	11377			
Trabajo	Indígena	Rural	OR	LC	p
Sí	168	1005	0.46	0.39 - 0.55	0.000
No	2491	6840			
Recreación	Rural	Urbano	OR	LC	p
Sí	1749	3732	0.72	0.67 - 0.77	0.000
No	6385	9778			
Recreación	Indígena	Urbano	OR	LC	p
Sí	776	3732	0.91	0.83 - 0.99	0.0384668
No	2238	9778			
Recreación	Indígena	Rural	OR	LC	p
Sí	776	1749	1.27	1.15 - 1.40	0.0000022
No	2238	6385			

Fuente: Encuesta Nacional de Salud y Calidad de Vida. República de Panamá. Año: 2007.

Alcohol

Prevalencia de Vida

Para una muestra de 25748 personas, seis de cada diez entrevistados (59.1%), **han consumido bebidas alcohólicas alguna vez en su vida**, es decir 15221 entrevistados, con una prevalencia de vida para **hombres** de 83.8% y para las **mujeres** de 42.7%.

La prevalencia de vida para las **áreas urbanas** fue de 63.4%, en tanto que uno de cada 2 entrevistados de las

GRAFICA No.11

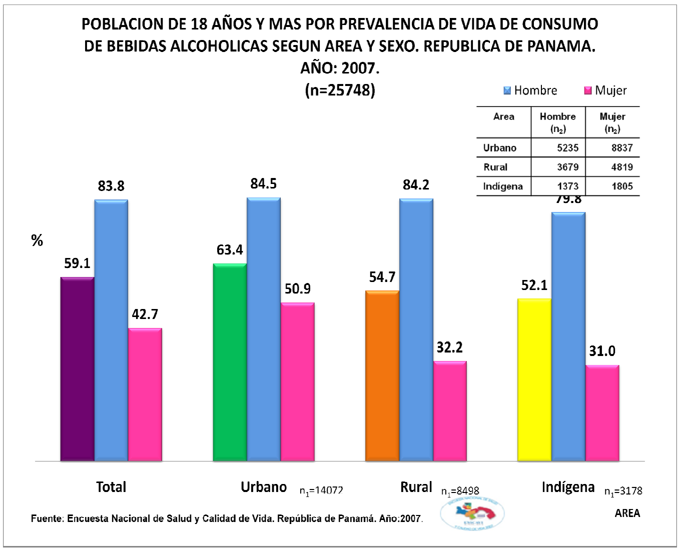

Supplement: Document S1 — ENSCAVI 2007 - Tobacco Consumption Prevalence in Panama. (PDF) [file pone.0088784.s002.pdf]
