# Supplementary material for: The Association of Tobacco Control Policies and the Risk of Acute Myocardial Infarction Using Hospital Admissions Data
Source: PLoS One. 2014 Feb 10;9(2):e88784. doi: 10.1371/journal.pone.0088784 (PMC3919809; doi:10.1371/journal.pone.0088784)
Supplement: Document S2 — Global Adult Tobacco Survey in Panama 2013 - Fact Sheet. (PDF) [file pone.0088784.s003.pdf]

GATS | ENCUESTA MUNDIAL DE TABACO EN ADULTOS

Panamá 2013

Objetivos de GATS

La Encuesta Mundial de Tabaquismo en Adultos (GATS) es el estándar mundial para monitorear en forma sistemática el consumo de tabaco en adultos (tabaco con humo y tabaco sin humo), así como los principales indicadores de control del tabaco.

GATS es una encuesta representativa a nivel nacional y regional que utiliza un protocolo estándar para los distintos países incluyendo Panamá.

GATS fortalece la capacidad de los países para diseñar, implementar y evaluar programas para el control del tabaco. Además, ayuda a los países a cumplir con sus obligaciones en relación al Convenio Marco para el Control del Tabaco de la Organización Mundial de la Salud (CMCT-OMS) con el fin de generar datos comparables entre los países. La OMS ha desarrollado el MPOWER, un paquete de asistencia técnica compuesto por 6 políticas basadas en la evidencia que incluye:

Monitorear el consumo de tabaco y las políticas de prevención.
Proteger a las personas del humo de tabaco.
Ofrecer ayuda para abandonar el consumo.
Warn (Advertir) acerca de los peligros del tabaco
Enforce (Hacer cumplir) la prohibición de publicidad, promoción y patrocinio.
Raise (Aumentar) los impuestos al tabaco.

Metodología de GATS

GATS utiliza una metodología estandarizada a nivel mundial. Incluye información sobre las características de los encuestados, uso de tabaco (con humo y sin humo), cesación del consumo de tabaco, exposición al humo de tabaco de segunda mano, economía, medios de comunicación y conocimientos, actitudes y percepciones acerca del uso de tabaco. En Panamá, GATS fue conducido en 2013 como una encuesta de hogares a personas de 15 años o más, por el Instituto Conmemorativo Gorgas de Estudios de la Salud (ICGES), bajo la coordinación del Ministerio de Salud de Panamá. Se utilizó un diseño muestral multietápico, estratificado por conglomerados, para producir datos representativos a nivel nacional. Un total de **19,603 hogares** fueron muestreados y un individuo en forma aleatoria en cada vivienda seleccionada, para completar la encuesta. La información fue recogida usando dispositivos electrónicos manuales. Un total de **16,962** entrevistas individuales fueron completadas con una tasa de respuesta total de **88.4%.**

Principales Resultados de GATS

USO DEL TABACO

- 9.4% de los hombres, 2.8% de las mujeres, y 6.1% del total (163,000) fuman tabaco actualmente.
- 1.0% de los hombres, 0.5% de las mujeres, del total 0.8% (20,000) consume tabaco no fumado.

CESACIÓN

- 6 de cada 10 fumadores actuales planearon o estaban pensando dejar de fumar.
- 5 de cada 10 fumadores hicieron un intento para dejar de fumar en los últimos 12 meses.

EXPOSICIÓN AL HUMO DE SEGUNDA MANO

- 5.6% de los adultos que trabajan en interiores (41,000) manifestaron que están expuestos al humo del tabaco en su lugar de trabajo.
- 4.4% de los adultos (118,000) manifestaron que están expuestos al humo del tabaco en el hogar.
- 12.4% de los adultos (157,000) manifestaron que están expuestos al humo del tabaco cuando visitaban restaurantes.

ECONOMÍA

- En promedio, un fumador de cigarrillos actual gasta 72.6 USD por mes en cigarrillos manufacturados.
- 36.3% de los fumadores de cigarrillos manufacturados actuales han comprado cigarrillos ilegales en los últimos 12 meses.

MEDIOS DE COMUNICACIÓN

- 6 de cada 10 adultos había visto información contra el tabaco en la televisión o la radio.
- 2 de cada 10 adultos había visto mercadeo de cigarrillos en las tiendas donde se venden cigarrillos.
- 3 de cada 10 adultos había visto comercialización de cigarrillos (que no sea en el comercio) o patrocinio de eventos deportivos.

CONOCIMIENTOS, ACTITUDES Y PERCEPCIONES

- 90.6% de los adultos cree que fumar causa enfermedades graves.
- 87.5% de los adultos cree que el humo que respiran de otras personas fumadoras causa enfermedades graves en los no fumadores.

FACT SHEET

GATS | ENCUESTA MUNDIAL DE TABACO EN ADULTOS

Panamá 2013

CONSUMO DEL TABACO

FUMADORES DE TABACO	HOMBRS (%)	MUJERES (%)	TOTAL (%)
Fumadores actuales de tabaco	9.4	2.8	6.1
Fumadores diarios de tabaco	4.4	1.2	2.8
Fumadores actuales de cigarrillos [1]	8.9	2.7	5.8
Fumadores diarios de cigarrillos [1]	3.6	1.2	2.4
Ex fumadores de tabaco diarios[2] *(entre todos los adultos)*	3.1	2.2	2.6
Ex fumadores de tabaco diarios[2] *(entre los adultos fumadores)*	30.6	52.3	37.0
	HOMBRES (#)	MUJERES (#)	TOTAL (#)
Promedio del número de cigarrillos fumados por día entre los fumadores de cigarrillos	16.3	10.1	14.8
**FUMADORES DE TABACO SIN HUMO**	HOMBRES (%)	MUJERES (%)	TOTAL (%)
Fumadores de Tabaco Sin Humo	1.0	0.5	0.8
**FUMADORES DE TABACO** *(con humo y/o sin humo)*			
Fumadores Actuales	9.7	3.1	6.4

Fumadores de Tabaco por Edad y Sexo, GATS Panamá 2013

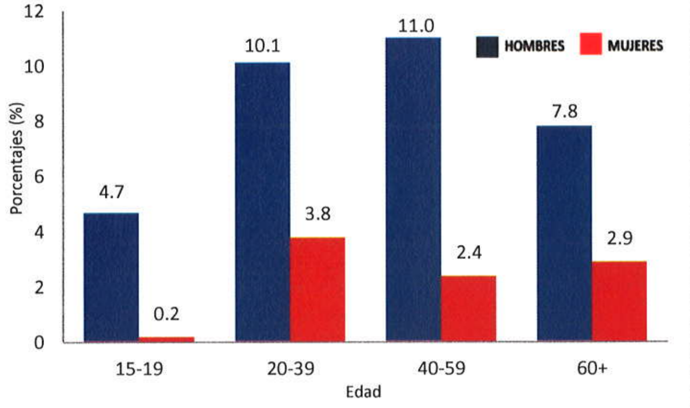

CESACIÓN

	HOMBRES (%)	MUJERES (%)	TOTAL (%)
Fumadores que hicieron un intento por dejar de fumar en los últimos 12 meses[3]	44.4	48.2	45.2
Fumadores actuales que planearon o piensan intentar dejar de fumar.	62.1	71.8	64.4
Fumadores que fueron aconsejados a dejar de fumar por un proveedor de salud en los últimos 12 meses[3,4]	63.7	53.0	60.4

EXPOSICIÓN AL HUMO DE SEGUNDA MANO

	HOMBRES (%)	MUJERES (%)	TOTAL (%)
Adultos expuestos al humo de tabaco en su lugar de trabajo[5,†]	7.4	3.7	5.6
Adultos expuestos al humo de tabaco en su hogar en el último mes	5.3	3.5	4.4
Adultos expuestos al humo de tabaco en restaurantes[6]	14.0	10.8	12.4

ECONOMÍA

Gasto promedio en un paquete de 20 cigarrillos manufacturados [USD]	6.55
Gasto medio de cigarrillos al mes entre los fumadores de cigarrillos manufacturados [USD]	72.56
Costo de 100 paquetes de cigarrillos manufacturados como porcentaje del producto interno bruto (PIB) 2013[7]	5.9%
Fumadores de cigarrillos manufacturados actuales que han comprado cigarrillos ilegales en los últimos 12 meses[8]	36.3%

MEDIOS DE COMUNICACIÓN

PUBLICIDAD DE LA INDUSTRIA TABACALERA	FUMADORES (%)	NO FUMADORES (%)	TOTAL (%)
Adultos que notaron publicidad de cigarrillos en los sitios de venta de cigarrillos[9†]	27.8	19.6	20.1
Adultos que notaron publicidad o promoción de cigarrillos en otros sitios (distinto de los de sitios de venta), eventos deportivos o patrocinio de eventos†	31.6	28.3	28.5
**PUBLICIDAD EN CONTRA**	HOMBRES (%)	MUJERES (%)	TOTAL (%)
Fumadores actuales que pensaron en dejar de fumar como consecuencia de las advertencias en los paquetes de cigarrillos†	39.0	47.7	41.0
	FUMADORES (%)	NO FUMADORES (%)	TOTAL (%)
Adultos que habían visto información en contra de fumar en la televisión o radio†	53.3	56.9	56.7

CONOCIMIENTOS, ACTITUDES Y PERCEPCIONES

	FUMADORES (%)	NO FUMADORES (%)	TOTAL (%)
Adultos que creen que fumar causa enfermedades graves	93.1	90.5	90.6
Adultos que creen que respirar humo de tabaco ajeno causa enfermedades graves en los no fumadores	92.7	87.2	87.5
Adultos que apoyan la ley nacional que prohíbe fumar en todos los lugares públicos.	91.5	87.2	87.5
	FUMADORES (%)	NO FUMADORES (%)	TOTAL (%)
Adultos que creen que fumar tabaco con humo causa enfermedades graves	80.1	83.3	83.3

[1] Incluye los cigarrillos manufacturados, los cigarrillos enrollados a mano, y kreteks. [2] No Fumadores actuales. [3] Incluye fumadores actuales y quienes intentaron dejar de fumar en los pasados 12 meses [4] Entre los que visitaron un proveedor de Salud en los últimos 12 meses. [5] Entre los que trabajaron fuera de su hogar, usualmente trabajan en interiores o ambos interiores y exteriores. [6] Entre los que visitaron algún restaurante en los últimos 30 días. [7] 2013 PIB estimado en 11,149.664 del sitio web del Fondo Monetario Internacional (FMI) (accedido el 2 de Octubre de 2013). [8] Definido como la compra de paquetes de cigarrillos que no tenían ninguna advertencia sanitaria gráfica. [9] Incluye quienes advirtieron cigarrillos a precios de venta, regalos gratis u ofertas de descuentos ofrecidos en productos cuando compraban cigarrillos, o cualquier publicidad o signo promocionando cigarrillos en tiendas donde son vendidos. [†] Durante los pasados 30 días.

NOTA: El uso actual se refiere a todos los días y menos de uso diario. Los adultos se refieren a las personas mayores de 15 años y más. Los datos han sido ponderados para ser representativos a nivel nacional de todos los hombres y mujeres no institucionalizados de 15 años y más. Los porcentajes reflejan la prevalencia de cada indicador en cada grupo, no la distribución a través de grupos.

El financiamiento para GATS Panamá fue proporcionado por el Gobierno de Panamá. Asistencia Técnica fue proporcionada por Centro de Control de Enfermedades - Centers for Disease Control and Prevention (CDC), la Organización Mundial de la Salud (OMS), la Escuela de Salud Pública Johns Hopkins Bloomberg y RTI Internacional. El Soporte del Programa fue proporcionado por la Fundación.
