# Supplementary material for: The Association of Tobacco Control Policies and the Risk of Acute Myocardial Infarction Using Hospital Admissions Data
Source: PLoS One. 2014 Feb 10;9(2):e88784. doi: 10.1371/journal.pone.0088784 (PMC3919809; doi:10.1371/journal.pone.0088784)
Supplement: Document S3 — Law 13, 2008 - Smoking ban in Panama. (PDF) [file pone.0088784.s004.pdf]

LEY No.13

De 24 de enero de 2008

Que adopta medidas para el control del tabaco

y sus efectos nocivos en la salud

LA ASAMBLEA NACIONAL

DECRETA:

Capítulo I

Disposiciones Generales

Artículo 1. La presente Ley tiene por objeto adoptar medidas para proteger la salud de la población panameña del efecto nocivo y de los perjuicios que tiene el tabaco para la salud, en virtud de la Constitución Política.

Artículo 2. Le corresponderá al Ministerio de Salud informar sobre las consecuencias sanitarias, la naturaleza adictiva y la amenaza mortal del consumo de tabaco y de la exposición al humo del tabaco para los seres humanos. Para tal fin, el Gobierno Nacional destinará las partidas presupuestarias correspondientes.

Artículo 3. El Estado, con la participación de la sociedad civil, elaborará políticas apropiadas para prevenir, controlar y reducir el consumo de tabaco, la adicción a la nicotina y la exposición al humo del tabaco, y adoptará lo necesario para aplicar efectivamente dichas políticas de salud pública.

Artículo 4. Para efectos de la presente Ley, los siguientes términos se entenderán así:

1. *Ambiente laboral cerrado*. Área en la que no existe ventilación natural y donde uno o más trabajadores realizan actividades de producción que involucran la exposición a riesgos químicos, físicos, biológicos, higiénicos y psicosociales.
2. *Control del tabaco*. Estrategias para la reducción de la oferta y la demanda de los productos del tabaco, así como de los daños que estos producen, con el objeto de mejorar la salud de la población y de eliminar o reducir el consumo de los productos del tabaco y la exposición al humo de este.
3. *Empaque y etiqueta externos*. Son las cajetillas, los cartones y similares utilizados en los productos del tabaco para la venta al por menor.
4. *Industria tabacalera*. Aquella que abarca a los fabricantes, distribuidores, mayoristas e importadores de los productos del tabaco.
5. *Patrocinio del tabaco*. Contribución que promueve directa o indirectamente un producto del tabaco o el uso de este, en cualquier acto o actividad o a cualquier persona.
6. *Producto del tabaco*. Producto preparado, total o parcialmente, utilizando como materia prima las hojas de tabaco y destinado a ser fumado, chupado, mascado o utilizado como rapé.
7. *Publicidad y promoción del tabaco*. Cualquier forma de comunicación, recomendación o acción comercial, que promueve directa o indirectamente un producto del tabaco o el uso de este.
8. *Publicidad, promoción y patrocinio transfronterizo del tabaco*. Cualquier forma de comunicación, recomendación o acción comercial, así como contribución a cualquier acto o actividad o persona, que se origina fuera de la República de Panamá y puede ser captada por un medio tecnológico disponible en el territorio nacional, para promover, directa o indirectamente, un producto del tabaco o el uso de este.

Capítulo II

Medidas Relacionadas con la Reducción

de la Demanda de Tabaco

Artículo 5. Se prohíbe el consumo de tabaco y de los productos de este en:

1. Las oficinas públicas y privadas nacionales, provinciales, comarcales y locales.
2. Los medios de transporte público en general y en las terminales de transporte terrestre, marítimo y aéreo.
3. Los lugares cerrados de acceso público donde haya concurrencia de personas.
4. Los ambientes públicos y privados, abiertos y cerrados, destinados a actividades deportivas.
5. Las áreas comunes de los edificios públicos y privados de uso comercial y doméstico.
6. Los ambientes laborales cerrados.
7. Las instituciones educativas y de salud, públicas y privadas.

Los gerentes o los encargados de los establecimientos, públicos o privados, serán los responsables de hacer cumplir al público en general y a sus empleados lo establecido en la presente Ley y, de ser necesario, podrán recurrir al auxilio de la Policía Nacional.

Artículo 6. En los paquetes y envases de productos del tabaco deberán aparecer impresas:

1. La advertencia: "FUMAR PUEDE CAUSAR LA MUERTE". Esta advertencia deberá aparecer a un costado del paquete o envase de los productos del tabaco, en letras claras, visibles, legibles y en colores contrastantes.
2. Una advertencia sanitaria adicional en la parte inferior de la cara frontal y posterior de cada paquete o envase de los productos del tabaco, con una imagen o pictograma, según lo dispuesto en esta Ley.
3. La información sobre el origen del producto, la fecha de producción y caducidad, el lugar donde se venderá el producto, el lote y el registro. El código de barras del producto no podrá verse adulterado ni tener etiquetas adheridas encima.

Artículo 7. En atención a lo dispuesto en el numeral 2 del artículo anterior, las advertencias sanitarias adicionales establecidas por el Ministerio de Salud se presentarán de la siguiente manera:

1. **Rotativas anualmente.**
2. **Claras, visibles y legibles.**
3. **Escritas en idioma español.**

Artículo 8. En atención al numeral 3 del artículo 6 de la presente Ley, dicha información deberá estar impresa a un costado de las etiquetas, de los paquetes o los envases de los productos del tabaco, deberá aparecer en forma destacada y estar protegida de adulteraciones por un código de barras.

De igual forma, deberá estar impreso el contenido tóxico de los productos del tabaco, especialmente la nicotina y el alquitrán, así como el contenido de sus emisiones, en especial el monóxido de carbono y el benzopireno. Esta información deberá estar a un costado de las etiquetas, de los paquetes o los envases de los productos del tabaco, en letra clara, visible y legible, inserta en un recuadro.

Los laboratorios que analicen y verifiquen los químicos de los productos del tabaco deberán estar acreditados y aprobados por el Ministerio de Salud.

Artículo 9. Las advertencias que deben hacerse de acuerdo con esta Ley contendrán información sobre los riesgos y daños para la salud, la cual ocupará el cincuenta por ciento (50%) de la cara frontal y posterior del paquete de los productos del tabaco, y será impresa en el envase y no en el envoltorio exterior desechable.

Las advertencias sanitarias se colocarán en la parte inferior de los envases, con excepción de la base de estos. El área física será distribuida entre las caras principales y los bordes laterales de los envases. En ambas caras principales deberán aparecer el pictograma y el mensaje escrito de la advertencia.

Durante cada periodo rotativo circularán en el mercado nacional cinco clases de advertencias sanitarias, distribuidas proporcionalmente al volumen de envases.

Artículo 10. Las advertencias deberán estar claramente visibles, escritas en letra tipo arial en colores primarios contrastantes que faciliten la lectura, y tener un tamaño que no sea menor de catorce puntos. El texto de la advertencia sanitaria se insertará en un recuadro.

Artículo 11. Para la impresión de las imágenes o pictogramas en los paquetes y envases de los productos del tabaco, se utilizará la técnica de separación de colores, y su tamaño mínimo será del sesenta por ciento (60%) del espacio designado para la advertencia sanitaria establecida por el Ministerio de Salud.

Artículo 12. Los temas contenidos en las advertencias sanitarias y los pictogramas serán establecidos por el Ministerio de Salud. La industria tabacalera o sus subsidiarias los reproducirán en todos los envases de los productos del tabaco destinados al consumidor final.

Artículo 13. La promoción de productos del tabaco en sus etiquetas, paquetes o envases no se hará de manera falsa, equívoca, engañosa o que pueda inducir a error en sus características o efectos para la salud, riesgos o emisiones.

Para tal efecto, se prohíbe la utilización de términos, elementos descriptivos, marcas de fábrica o de comercio, o signos figurativos o de otra clase, que produzca directa o indirectamente la falsa impresión de que un determinado producto del tabaco es menos nocivo que otro.

Artículo 14. Se prohíbe totalmente cualquier forma de publicidad, promoción y patrocinio del tabaco y de sus productos, así sea a través de medios indirectos o subliminales, dirigida a menores o mayores de edad. Igualmente se prohíbe toda forma de publicidad, promoción y patrocinio transfronterizo del tabaco y sus productos, que penetren en el territorio nacional.

Artículo 15. El Estado diseñará y aplicará programas de promoción del abandono del consumo de tabaco. Para este propósito, adoptará las medidas necesarias a fin de que el sector público de salud facilite el acceso a tratamientos contra la dependencia del tabaco, incluidos productos farmacéuticos, con la finalidad de disminuir la dependencia del tabaco en la población.

Capítulo III

Medidas Relacionadas con la Reducción

de la Oferta de Tabaco

Artículo 16. Se prohíbe la venta de productos importados derivados del tabaco que no estén expresamente dirigidos al mercado panameño.

Artículo 17. Se prohíbe la distribución, venta, regalo o cualquier forma de comercialización de productos del tabaco que hayan sido introducidos al país sin cumplir los trámites aduaneros vigentes o que no estén destinados para la distribución dentro del territorio nacional.

Artículo 18. El Estado, a través de reglamentación, adoptará y aplicará en todas las áreas económicas especiales o zonas libres o francas del país medidas para vigilar, documentar y controlar, en forma específica, el almacenamiento y la distribución de productos del tabaco que se encuentren o se desplacen en régimen de suspensión de impuestos o derechos.

Las personas naturales o jurídicas que incumplan las obligaciones que se deriven del presente artículo serán sancionadas pecuniariamente, de acuerdo con la gravedad de la falta y, en caso de reincidencia, se les revocará la licencia para operar.

Artículo 19. Se prohíbe la venta de productos del tabaco a los menores de edad, para lo cual se adoptan las siguientes medidas:

1. Los proveedores y expendedores de productos del tabaco tendrán la obligación de colocar, a su costo, carteles visibles, claros y destacados en el interior de los lugares de venta, que indiquen que se prohíbe la venta de productos del tabaco a los menores de edad.
2. Ningún almacén de venta al por menor podrá tener los productos del tabaco en lugares directamente accesibles para el cliente.
3. Se prohíbe la fabricación, importación y venta de dulces, refrigerios, juguetes y otros objetos que tengan la forma y el diseño de productos del tabaco y que puedan resultar atractivos para los menores de edad.
4. Los comerciantes que vendan productos del tabaco están obligados a comprobar que la persona que los adquiere sea mayor de edad. La comprobación se hará mediante la presentación de la cédula de identidad personal, la licencia de conducir, el pasaporte o cualquier otra identificación oficial válida.
5. El Ministerio de Salud y los municipios destinarán una línea telefónica para que la población efectúe las denuncias que violen esta disposición.

Artículo 20. Se prohíbe la venta de cigarrillos sueltos o en paquetes pequeños, que contengan menos de veinte cigarrillos, que hagan más accesible este producto a los menores de edad.

Artículo 21. Los menores de edad no podrán dedicarse a la venta de productos del tabaco ni ser empleados por otras personas para tal fin.

Artículo 22. Se prohíbe la utilización de máquinas expendedoras o dispensadoras de tabaco.

Artículo 23. A partir de la entrada en vigencia de la presente Ley, queda prohibida la venta de productos derivados del tabaco en establecimientos deportivos, educativos y de salud, públicos o privados. Esta disposición incluye a los concesionarios ubicados en ellos.

Artículo 24. El incumplimiento de la prohibición de la publicidad y de las advertencias sanitarias contenidas en esta Ley constituye una afectación a la salud de la población nacional y conlleva el decomiso y la destrucción de los productos del tabaco y del material publicitario. En caso de reincidencia, la autoridad revocará la licencia respectiva.

Artículo 25. Los planes y programas de estudio de la educación general básica y de la educación media en ambas modalidades deberán considerar objetivos y contenidos destinados a educar e instruir a los alumnos sobre los daños que provoca en el organismo el consumo de productos hechos con tabaco y la exposición al humo de este, así como su carácter adictivo.

Capítulo IV

Infracciones

Artículo 26. Constituye infracciones a lo previsto en esta Ley lo siguiente:

1. Fumar en los lugares en donde exista prohibición total.
2. No exponer en lugar visible, en los establecimientos en los que esté autorizada la venta de productos del tabaco, los carteles que informen de la prohibición de venta del tabaco a los menores de edad y adviertan sobre los perjuicios para la salud derivados del uso del tabaco.
3. No informar en la entrada de los establecimientos de la prohibición de fumar.
4. Permitir que se fume en los lugares en que exista prohibición total.
5. Entregar o distribuir muestras, gratuitas o no gratuitas, de cualquier producto del tabaco.
6. Vender o entregar a personas menores de edad productos del tabaco o que imiten productos del tabaco que induzcan a fumar, que tengan forma de productos del tabaco y puedan resultar atractivos para los menores de edad.
7. Comercializar productos del tabaco utilizando el nombre, la marca, el símbolo o cualquier otro signo distintivo de cualquier otro bien o servicio en condiciones distintas de las permitidas en esta Ley.
8. Vender, ceder o suministrar productos del tabaco, en condiciones distintas de las permitidas en esta Ley.
9. Publicitar, promocionar y patrocinar los productos del tabaco en todo medio de comunicación.

Artículo 27. La cuantía de las sanciones que se impongan se graduará teniendo en cuenta el riesgo generado para la salud, la capacidad económica del infractor, la repercusión social de la infracción, el beneficio que haya reportado al infractor la conducta sancionada, si el afectado es un menor de edad y la reincidencia del infractor.

Artículo 28. Cuando la infracción se trate de publicidad serán considerados responsables la empresa publicitaria y el beneficiario de la publicidad solidariamente, entendiendo por este último el titular de la marca o producto anunciado, así como el titular del establecimiento o espacio en el que se emite el anuncio.

Artículo 29. Las infracciones a la presente Ley podrán ser denunciadas ante las autoridades competentes, por cualquier persona, y serán sancionadas por el Ministerio de Salud, conforme a lo preceptuado en el Código Sanitario.

Artículo 30. Los fondos obtenidos en concepto de multa por las infracciones a la presente Ley se utilizarán para la implementación de programas de prevención, de promoción y de educación, con relación a los efectos nocivos del tabaco.

Capítulo V

Disposiciones Finales

Artículo 31. El artículo 171 del Código Sanitario queda así:

Artículo 171. Queda prohibida cualquier forma de publicidad o propaganda referente a la higiene, a la medicina preventiva y curativa, a las drogas y a los productos de uso higiénico o medicinal, cosméticos y productos de belleza, que no fueran previamente aprobados por el Ministerio de Salud, el cual objetará toda propaganda encaminada a engañar o explotar al público, o que en cualquier forma pueda resultar perjudicial para la salud.

Constituye engaño o perjuicio público recomendar por cualquier método de propaganda servicios médicos no autorizados oficialmente o en desacuerdo con los hechos científicos; preconizar medicina a la que se atribuyan propiedades que no posean o que no figuran entre las aceptables por el Ministerio al momento de la inscripción; y viciar en cualquier forma las disposiciones reglamentarias preestablecidas.

El Estado, a través del Ministerio de Salud o por ley, prohibirá cualquier tipo de publicidad, promoción o patrocinio de drogas, aun cuando sean lícitas, siempre que científicamente se compruebe que estas son perjudiciales a la salud humana y que constituyen una amenaza a la salud pública.

Artículo 32. El Órgano Ejecutivo tendrá un término no mayor de tres meses, contado a partir de la promulgación, para reglamentar la presente Ley.

Artículo 33. Esta Ley modifica el artículo 171 del Código Sanitario y deroga el Decreto Ejecutivo 17 de 11 de marzo de 2005.

Artículo 34. Esta Ley comenzará a regir tres meses después de su promulgación.

COMUNÍQUESE Y CÚMPLASE.

Proyecto 25 de 2004 aprobado en tercer debate en el Palacio Justo Arosemena, ciudad de Panamá, a los tres días del mes de diciembre del año dos mil siete.

El Presidente,

Pedro Miguel González P.

El Secretario General,

Carlos José Smith S.

ÓRGANO EJECUTIVO NACIONAL. PRESIDENCIA DE LA REPÚBLICA.

PANAMÁ, REPÚBLICA DE PANAMÁ, DE 24 DE enero DE 2008.

MARTÍN TORRIJOS ESPINO
Presidente de la República

ROSARIO TURNER MONTENEGRO
Ministra de Salud
