# Supplementary material for: The Association of Tobacco Control Policies and the Risk of Acute Myocardial Infarction Using Hospital Admissions Data
Source: PLoS One. 2014 Feb 10;9(2):e88784. doi: 10.1371/journal.pone.0088784 (PMC3919809; doi:10.1371/journal.pone.0088784)
Supplement: Document S4 — Law 69, 2009 - Tobacco Tax Increase in Panama. (PDF) [file pone.0088784.s005.pdf]

LEY No.69

De 6 de Noviembre de 2009

Que prohíbe la equiparación en los contratos y otras modalidades jurídicas

en los que el Estado sea parte, reforma disposiciones de contrataciones públicas

y dicta otras disposiciones

LA ASAMBLEA NACIONAL

DECRETA:

Artículo 1. Esta Ley prohíbe cambios, ajustes, modificaciones o adecuaciones, bajo el concepto de equiparación, en las tarifas, exenciones, términos y condiciones establecidos en las concesiones, arrendamientos, contratos de inversión en general y cualesquiera otras modalidades jurídicas administrativas, mediante adendas y similares, otorgados por el Estado, a través de cualesquiera de sus dependencias de Gobierno, que impliquen, en cualquier forma, detrimento para el Estado y para sus asociados.

Artículo 2. La presente Ley regirá los contratos públicos que realicen el Gobierno Central, las entidades autónomas y semiautónomas, los municipios, los intermediarios financieros y las empresas mixtas en que el Estado sea propietario de, por lo menos, el 51% de sus acciones o patrimonio, así como los que se efectúen con fondos públicos o bienes nacionales, en virtud de los presupuestos establecidos en el artículo 1 de la Ley 22 de 2006, de contratación pública.

Artículo 3. El numeral 46 del artículo 2 de la Ley 22 de 2006 queda así:

Artículo 2. Glosario. Para los fines de la presente Ley, los siguientes términos se entenderán así:

...

46. *Tribunal Administrativo de Contrataciones Públicas*. Es el Tribunal independiente e imparcial que conocerá, en única instancia, de:

a. El recurso de impugnación contra el acto de adjudicación, la declaratoria de deserción o el acto o resolución por la cual se rechazan las propuestas emitidos por las entidades, en los procedimientos de selección de contratista.

b. El recurso de apelación contra la resolución administrativa del contrato y la inhabilitación del contratista.

c. Las acciones de reclamo no resueltas por la Dirección General de Contrataciones Públicas, dentro del término de los cinco días hábiles que tiene para resolver.

...

Artículo 4. Se adiciona el numeral 49 al artículo 2 de la Ley 22 de 2006, así:

Artículo 2. Glosario. Para los fines de la presente Ley, los siguientes términos se entenderán así:

...

49. *Licitación abreviada.* Procedimiento de selección de contratista en el que el Estado selecciona y adjudica con base en el menor precio o, en los actos de mejor valor, en la mayor ponderación, siempre que se cumpla con todos los requisitos y aspectos técnicos exigidos en el pliego de cargos. Se podrá utilizar cuando el monto de la contratación sea superior a los treinta mil balboas (B/.30,000.00), el objeto de la contratación responda a la necesidad de satisfacer el interés social y se requiera que se efectúe en términos de tiempo menores a los dispuestos en otras modalidades de contratación descritas en la presente Ley.

Artículo 5. Se adiciona un párrafo final al artículo 11 de la Ley 22 de 2006, así:

Artículo 11. Derechos de las entidades contratantes. Son derechos de las entidades contratantes los siguientes:

…

Cuando se trata de contratos de concesión, las entidades contratantes quedan facultadas para realizar inspecciones sobre las áreas, los bienes o los servicios objeto del contrato, a fin de verificar el cumplimiento de las obligaciones adquiridas por los concesionarios.

Artículo 6. Se adiciona el numeral 15 al artículo 12 de la Ley 22 de 2006, así:

Artículo 12. Obligaciones de las entidades contratantes. Son obligaciones de las entidades contratantes las siguientes:

…

15. Vigilar el estricto cumplimiento del contrato y denunciar todas las contrataciones públicas que lesionen el interés o patrimonio de la Nación.

Artículo 7. El artículo 14 de la Ley 22 de 2006 queda así:

Artículo 14. Obligaciones y deberes del contratista. Son obligaciones del contratista las siguientes:

1. **Cumplir con el objeto del contrato y sus condiciones, dentro del término pactado.**
2. **Colaborar con la entidad licitante en lo necesario para que el objeto del contrato se cumpla y sea de la mejor calidad.**
3. **Acatar las instrucciones que durante el desarrollo del contrato le sean impartidas por la entidad contratante, siempre que estén amparadas dentro de la relación contractual.**
4. **Actuar con lealtad y buena fe en las distintas etapas contractuales, evitando las dilaciones que puedan presentarse.**
5. **Garantizar la calidad de las obras realizadas, así como de los bienes y los servicios contratados, y responder por ello de acuerdo con lo pactado.**
6. **Ser legalmente responsable cuando formule propuestas en que se fijen condiciones económicas y de contrataciones artificialmente subvaluadas, con el propósito de obtener la adjudicación del contrato.**
7. **Ser legalmente responsable por haber ocultado, al contratar, inhabilidades, incompatibilidades o prohibiciones o por haber suministrado información falsa.**
8. **Permitir el libre acceso a las instalaciones objeto de contratación para los fines indicados en este artículo.**
9. **Responder exclusivamente por las obligaciones o reclamaciones que surjan de las relaciones contractuales adquiridas dentro del periodo de vigencia contractual, incluyendo las de naturaleza administrativa, civil, comercial, laboral o cualquiera otra que implique algún tipo de responsabilidad en materia de obligaciones.**

En el caso del numeral 8, deberá permitir el ingreso de los funcionarios designados y autorizados por los organismos, las instituciones o las entidades estatales correspondientes, así como de las personas naturales y jurídicas que sean designadas o contratadas por el Estado para evaluar, fiscalizar y auditar, así como para cualquier otro fin pertinente al contrato. Además deberá facilitar los originales de los documentos que se soliciten, incluyendo los libros contables, siempre que estos incidan directamente en la determinación de los pagos que deba realizar. El incumplimiento de esta disposición dará lugar a la resolución administrativa del contrato de concesión o al rescate administrativo, según corresponda, conforme al procedimiento establecido para tal efecto en esta Ley.

Cuando sea una persona jurídica, el ciento por ciento (100%) de sus acciones deberán ser nominativas.

Artículo 8. El artículo 20 de la Ley 22 de 2006 queda así:

Artículo 20. Equilibrio contractual. En los contratos públicos de duración prolongada se podrán pactar cláusulas y condiciones encaminadas a mantener, durante la vigencia del contrato el equilibrio contractual existente al momento de la celebración del contrato con la finalidad de que, si tales condiciones se alteran por hechos extraordinarios e imprevisibles, se pueda modificar para mantener el equilibrio.

Las partes podrán suscribir los acuerdos y pactos que sean necesarios para restablecer el equilibrio contractual, incluyendo montos, condiciones, forma de pago de gastos adicionales, reconocimiento de costos financieros e intereses, si a ello hubiera lugar, en la forma prevista en la modificación del contrato, cuyo pago adicional, si lo hubiera, se realizará de la manera establecida en el contrato modificado y de acuerdo con las disposiciones sobre erogaciones previstas en el Presupuesto General del Estado de la vigencia en que se deba hacer dicha erogación.

El equilibrio contractual al que se refiere este artículo no comprenderá, en ningún caso, la modificación de las cláusulas del contrato celebrado con el Estado para conseguir la equiparación de las condiciones y los términos de la contratación. En consecuencia, queda eliminada toda forma de equiparación para garantizar la competitividad y el desarrollo de obras y actividades, así como la prestación de servicios a través de normas uniformes, claras y transparentes en concordancia con el equilibrio contractual.

Parágrafo. En los contratos de obra, suministro de artículos de construcción o llave en mano, cuando por hechos o circunstancias posteriores a la celebración del contrato que no hayan podido preverse en ese momento o por causa de fuerza mayor o caso fortuito, se produzca una alteración u obstaculización sustancial de los costos que impida el cumplimiento del objeto del contrato, el Estado podrá tener como incluida en el contrato la cláusula de equilibrio contractual, aunque no haya sido pactada, a efectos de permitir la correspondiente adenda.

Artículo 9. Se adiciona el artículo 24-A a la Ley 22 de 2006, así:

Artículo 24-A. Requisitos de participación para personas jurídicas. Todo acto de selección de contratistas y contrataciones directas cuya cuantía exceda de tres millones de balboas (B/.3,000,000.00) en que participen personas jurídicas, las acciones de estas deben ser en su totalidad nominativas.

Independientemente de que las acciones nominativas sean emitidas a favor de otra persona jurídica, se deberá conocer con claridad la identidad de cada persona natural que sea directa o indirectamente el beneficiario final de por lo menos el cinco por ciento por ciento (5%) del capital accionario emitido y en circulación. Se exceptúan las personas jurídicas cuyas acciones comunes se coticen públicamente en bolsas de valores de una jurisdicción reconocida por la Comisión Nacional de Valores de Panamá. La falta de la documentación pertinente será impedimento para la participación de la persona jurídica como proponente en el acto de selección de contratista.

En el mismo sentido, será causal de incumplimiento aunque no se exprese en el contrato cualquier cambio en la composición accionaria de la sociedad contratista, concesionaria o inversionista, que no sea debidamente notificado a la entidad contratante o que impida conocer en todo momento quién es la persona natural que es finalmente el beneficiario de tales acciones, tomando en consideración que esta persona sea directa o indirectamente el beneficiario final de por lo menos el cinco por ciento (5%) del capital accionario emitido y en circulación.

En concordancia con el principio de transparencia, el contratista, concesionario o inversionista está obligado a presentar y publicar sus estados financieros, reservándose el Estado el derecho de publicarlos y darlos a conocer ampliamente, así como la lista de las personas naturales accionistas de la persona jurídica. El Estado podrá requerir en los actos de selección de contratista mencionados, cuya cuantía no exceda de los tres millones de balboas (B/.3,000,000.00), las mismas condiciones contenidas en este artículo.

Artículo 10. El artículo 31 de la Ley 22 de 2006 queda así:

Artículo 31. Publicación de la convocatoria. Dependiendo del monto y de la complejidad de las obras, los bienes y los servicios que se van a contratar, la publicación de la convocatoria se efectuará tomando en consideración los plazos mínimos que a continuación se detallan:

1. No menor de cuatro días hábiles, si el objeto del contrato recae en bienes o servicios y el monto es mayor de treinta mil balboas (B/.30,000.00) y no excede los ciento setenta y cinco mil balboas (B/.175,000.00).

2. No menor de cuarenta días calendario, si el objeto del contrato recae en bienes o servicios y el monto excede los ciento setenta y cinco mil balboas (B/.175,000.00).

No obstante, la entidad contratante podrá establecer un plazo menor a lo dispuesto en este numeral que, en ningún caso, será menor de diez días calendario, en las siguientes circunstancias:

a) Cuando la entidad contratante haya publicado un aviso en el Sistema Electrónico de Contrataciones Públicas "PanamaCompra" que contenga una descripción del acto público, los plazos aproximados para la presentación de las ofertas y, cuando resulte apropiado, las condiciones para la participación en dicho acto.

b) Cuando una entidad contrate mercancía o servicios comerciales que se venden o se ofrecen para la venta, y son regularmente comprados y utilizados por compradores no gubernamentales para propósitos no gubernamentales.

c) Cuando se produzca un estado de urgencia, debidamente acreditado, que haga impráctico o no viable cumplir con el plazo previsto. En este caso, la entidad contratante emitirá una resolución al respecto, la cual deberá ser publicada en el Sistema Electrónico de Contrataciones Públicas "PanamaCompra".

Cuando el objeto del contrato recae en obras, la publicación de la convocatoria se efectuará tomando en consideración los plazos mínimos que a continuación se detallan:

1. No menor de cuatro días hábiles, si el monto del contrato es mayor de treinta mil balboas (B/.30,000.00) y no excede los ciento setenta y cinco mil balboas (B/.175,000.00).

2. No menor de ocho días hábiles, si el monto del contrato es mayor de ciento setenta y cinco mil balboas (B/.175,000.00) y no excede los cinco millones de balboas (B/.5,000,000.00).

3. No menor de cuarenta días calendario, si el monto del contrato excede los cinco millones de balboas (B/.5,000,000.00).

No obstante, la entidad contratante podrá establecer un plazo menor a lo dispuesto en este numeral que, en ningún caso, será menor de diez días calendario, en las siguientes circunstancias:

a) Cuando la entidad contratante haya publicado un aviso en el Sistema Electrónico de Contrataciones Públicas "PanamaCompra" que contenga una descripción del acto público, los plazos aproximados para la presentación de las ofertas y, cuando resulte apropiado, las condiciones para la participación en dicho acto.

b) Cuando se produzca un estado de urgencia, debidamente acreditado, que haga impráctico o no viable cumplir con el plazo previsto. En este caso, la entidad contratante emitirá una resolución al respecto, la cual deberá ser publicada en el Sistema Electrónico de Contrataciones Públicas "PanamaCompra".

El reglamento de la presente Ley desarrollará la materia y será adoptado mediante decreto ejecutivo.

Artículo 11. El artículo 38 de la Ley 22 de 2006 queda así:

Artículo 38. Procedimientos de selección de contratista. Los procedimientos para seleccionar a quienes contraten con el Estado son los siguientes:

1. Contratación menor.

2. Licitación pública.

3. Licitación por mejor valor.

4. Licitación para convenio marco.

5. Licitación de subasta en reversa.

6. Licitación abreviada.

7. Subasta de bienes públicos.

Artículo 12. El artículo 39 de la Ley 22 de 2006 queda así:

Artículo 39. Contratación menor. El procedimiento para la contratación menor permitirá, de manera expedita, la adquisición de bienes, obras y servicios que no excedan los treinta mil balboas (B/.30,000.00), cumpliéndose con un mínimo de formalidades y con sujeción a los principios de contratación que dispone la presente Ley. La contratación menor se podrá dividir en rangos para garantizar la celeridad de este procedimiento y la adjudicación o declaración de desierto se hará en el cuadro de cotizaciones, el cual deberá contener la información originada en el acto y será firmado por el jefe de la entidad contratante o el funcionario en quien se delegue, al cual se adjuntarán los documentos de cada propuesta recibida.

Este procedimiento será establecido por la Dirección General de Contrataciones Públicas y su reglamentación será adoptada mediante decreto ejecutivo.

Artículo 13. El numeral 14 y el último párrafo del artículo 40 de la Ley 22 de 2006 quedan así:

Artículo 40. Licitación pública. …

En la celebración de la licitación pública, se observarán las siguientes reglas:

…

14. A partir de la fecha de la publicación descrita en el numeral anterior, los participantes de este acto público tendrán derecho a recibir, por parte de la entidad licitante, copia del expediente, incluyendo las propuestas de los participantes en el acto, y tendrán tres días hábiles para hacer observaciones a dicho informe, las cuales se unirán al expediente. Los costos asociados a la reproducción de los expedientes deberán ser pagados por los interesados.

…

Las entidades deberán solicitar a la Dirección General de Contrataciones Públicas que efectúe un proceso de selección de contratista para productos y servicios ya incluidos en el Catálogo Electrónico de Productos y Servicios que, por razones fundadas, les será más beneficioso.

Artículo 14. El numeral 13 y el último párrafo del artículo 41 de la Ley 22 de 2006 quedan así:

Artículo 41. Licitación por mejor valor. En la celebración de la licitación por mejor valor, se observarán las siguientes reglas:

…

13. A partir de la fecha de la publicación descrita en el numeral anterior, los participantes de este acto público tendrán derecho a recibir, por parte de la entidad licitante, copia del expediente, incluyendo las propuestas de los participantes en el acto, y tendrán tres días hábiles para hacer observaciones a dicho informe, las cuales se unirán al expediente. Los costos asociados a la reproducción de los expedientes deberán ser pagados por los interesados.

...

Dependiendo de una necesidad particular, las entidades deberán solicitar a la Dirección General de Contrataciones Públicas que efectúe un proceso de selección de contratista para productos y servicios ya incluidos en el Catálogo Electrónico de Productos y Servicios que, por razones fundadas, les será más beneficioso.

Artículo 15. Se adiciona el artículo 43-A a la Ley 22 de 2006, así:

Artículo 43-A. Licitación abreviada. La licitación abreviada es el procedimiento de selección de contratista en el que el Estado selecciona y adjudica con base en el menor precio o, en los actos de mejor valor, en la mayor ponderación, siempre que se cumpla con todos los requisitos y aspectos técnicos exigidos en el pliego de cargos. Se podrá utilizar cuando el monto de la contratación sea superior a los treinta mil balboas (B/.30,000.00), el objeto de la contratación responda a la necesidad de satisfacer el interés social y se requiera que se efectúe en términos de tiempo menores a los dispuestos en otras modalidades de contratación descritas en la presente Ley.

La licitación abreviada se sujetará a las siguientes reglas:

1. Se anunciará mediante publicación en el Sistema Electrónico de Contrataciones Públicas "PanamaCompra" y en el tablero de anuncios de la entidad con un plazo mínimo de cinco días hábiles. La entidad licitante podrá invitar a las personas naturales o jurídicas con idoneidad y capacidad demostrada en el objeto de la contratación, de manera simultánea a la publicación en el Sistema Electrónico de Contrataciones Públicas "PanamaCompra".

2. Los proponentes entregarán su oferta, la cual contendrá el precio ofertado con su correspondiente fianza de propuesta y la propuesta técnica ajustada a las exigencias del pliego de cargos.

3. La oferta de los proponentes será entregada en la fecha, la hora y el lugar señalados en el pliego de cargos.

4. Vencida la hora para la entrega de las propuestas, conforme a lo establecido en el pliego de cargos, no se recibirá ninguna más y se procederá a abrir las propuestas de cada uno de los proponentes en el orden en que fueron recibidas, las cuales se darán a conocer públicamente.

5. Quien presida el acto rechazará de plano las propuestas que no estén acompañadas por fianzas con montos o vigencias inferiores a los establecidos en el pliego de cargos.

La presente disposición es de carácter restrictivo, por lo que en ningún caso podrán ser rechazadas las propuestas por causas distintas a las aquí señaladas.

Contra el acto de rechazo, el agraviado podrá reclamar hasta el siguiente día hábil ante la Dirección General de Contrataciones Públicas, que tendrá un plazo máximo de tres días hábiles para resolver el reclamo.

6. Una vez conocidas las propuestas, quien presida el acto preparará un acta que se adjuntará al expediente, en la que se dejará constancia de todas las propuestas admitidas o rechazadas en el orden en que hayan sido presentadas, con expresión del precio propuesto, del nombre de los participantes, de los proponentes rechazados que hayan solicitado la devolución de la fianza de propuesta, del nombre y el cargo de los funcionarios que hayan participado en el acto de selección de contratista, así como de los particulares que hayan intervenido en representación de los proponentes y de los reclamos o las incidencias ocurridos en el desarrollo del acto. Esta acta será de conocimiento inmediato de los presentes en el acto y será publicada en el Sistema Electrónico de Contrataciones Públicas "PanamaCompra" y en los tableros de información de la entidad licitante.

7. Concluido el acto público, se unirán al expediente las propuestas presentadas, incluso las que se hubieran rechazado, así como las fianzas de propuesta, a menos que los licitantes vencidos o rechazados soliciten su devolución, entendiéndose con ello que renuncian a toda reclamación sobre la adjudicación de la licitación.

8. Inmediatamente después de levantada el acta, se remitirá el expediente, que contiene las propuestas de los participantes, a una comisión verificadora o evaluadora, que deberá ser previamente constituida por la entidad licitante. La comisión estará integrada por profesionales idóneos en el objeto de la contratación.

9. Para la verificación y evaluación de las propuestas, la comisión aplicará las reglas de evaluación determinadas para la licitación pública o licitación por mejor valor dispuestas en esta Ley.

10. El plazo para emitir el informe de la comisión no será superior a cinco días hábiles, a menos que la complejidad del acto amerite una única prórroga que no será superior a cinco días hábiles adicionales.

11. Una vez emitido el informe, este será publicado obligatoriamente en el Sistema Electrónico de Contrataciones Públicas "PanamaCompra" y estará disponible, ese mismo día, una copia impresa de este para los participantes en el acto que la deseen. Igualmente, la entidad licitante comunicará sobre la publicación de este informe a los proponentes que, en su propuesta, hayan incluido su correo electrónico o fax.

12. A partir de la fecha de la publicación descrita en el numeral anterior, los participantes de este acto público tendrán derecho a recibir, por parte de la entidad licitante, copia del expediente, incluyendo las propuestas de los participantes en el acto, y tendrán tres días hábiles para hacer observaciones a dicho informe, las cuales se unirán al expediente. Los costos asociados a la reproducción de los expedientes deberán ser pagados por los interesados.

13. Transcurrido el plazo descrito en el numeral anterior, el jefe de la entidad licitante o el funcionario en quien se delegue procederá, mediante resolución motivada, a adjudicar el acto público o a declararlo desierto, en un plazo no mayor de cinco días hábiles.

En los casos en que se presente un solo proponente y este cumpla con todos los requisitos y las exigencias del pliego de cargos, la recomendación de la adjudicación podrá recaer en él, siempre que el precio ofertado sea conveniente para el Estado o cumpla con un mínimo del ochenta por ciento (80%) del total de puntos y el precio ofertado sea conveniente para el Estado.

Las entidades del Estado deberán consultar el Catálogo Electrónico de Productos y Servicios antes de proceder a llamar a un acto de selección de contratista, así como verificar si los productos o servicios requeridos por la entidad están o no incluidos en dicho Catálogo.

Si los productos o servicios requeridos por la entidad licitante están en el Catálogo Electrónico de Productos y Servicios, la entidad está obligada a adquirirlos a través de dicho Catálogo.

Las entidades deberán solicitar a la Dirección General de Contrataciones Públicas que efectúe un proceso de selección de contratista para productos y servicios ya incluidos en el Catálogo Electrónico de Productos y Servicios que, por razones fundadas, les será más beneficioso.

Artículo 16. El numeral 2 del artículo 44 de la Ley 22 de 2006 queda así:

Artículo 44. Subasta de bienes públicos. La venta o el arrendamiento de los bienes muebles o inmuebles del Estado podrá realizarse mediante una subasta pública, y para ello se seguirán las siguientes reglas:

…

2. Se anunciará, mediante publicación, en la forma y con la antelación establecidas en los artículos 30 y 31 de la presente Ley, de acuerdo con la cuantía del acto público. En adición a lo anterior, deberán incluirse los bienes que hayan de venderse o arrendarse, el lugar en que se encuentran, el valor estimado de cada uno y la hora de inicio y de finalización de la subasta. El periodo de duración de la subasta no deberá ser mayor a tres horas.

…

Artículo 17. El artículo 58 de la Ley 22 de 2006 queda así:

Artículo 58. Contrataciones que celebre el Fondo de Inversión Social. Los contratos que celebre el Fondo de Inversión Social estarán exceptuados de la aplicación de la presente Ley.

Artículo 18. Se adiciona el artículo 72-A a la Ley 22 de 2006, así:

Artículo 72-A. Plazo máximo de los contratos. Las contrataciones con el Estado se otorgarán por un plazo máximo de veinte años y podrán ser prorrogadas a solicitud del contratista por un plazo que no exceda al señalado originalmente en el contrato. Para tal efecto, el contratista deberá solicitar dicha prórroga un año antes de su vencimiento.

Sin perjuicio de lo anterior, no será obligatorio para el Estado convenir en la prórroga de los contratos de concesión en los mismos términos y condiciones previstos en el contrato original.

El Estado tendrá derecho de incluir en los contratos cualquiera otra cláusula o condición que estime conveniente, a fin de asegurar sus intereses, respetando el principio de transparencia y el equilibrio contractual de las partes contenidos en esta Ley.

El Estado no podrá conceder periodos de gracia, entendiéndose que los pagos que deban hacer los contratistas serán exigibles desde el momento que se establezca en el respectivo contrato de conformidad con las reglamentaciones vigentes y en ningún caso podrán ser posteriores a la fecha de inicio de operaciones que impliquen ingresos.

Artículo 19. Se adiciona un párrafo al artículo 75 de la Ley 22 de 2006, así:

Artículo 75. Inicio de la ejecución de la obra. …

Cuando el solicitante de una contratación pública inicie la construcción de cualquier tipo de infraestructura o el acondicionamiento de terreno, la prestación de los servicios o la provisión de bienes objeto de la concesión otorgada, sin el refrendo del respectivo contrato y sin las autorizaciones requeridas, no se le otorgará la concesión y se le sancionará con multa equivalente al doble del daño causado o al doble del monto que el Estado recibiría en virtud del contrato de concesión durante el tiempo que haya utilizado sin autorización el área de la concesión, y deberá restablecer, a su costo, el área afectada a sus condiciones originales. Para la imposición de la sanción descrita en el presente artículo se actuará conforme al procedimiento establecido en la Ley 38 de 2000.

Artículo 20. El artículo 84 de la Ley 22 de 2006 queda así:

Artículo 84. Clasificación de los contratos llave en mano. Podrán celebrarse contratos llave en mano completos o parciales.

Se consideran contratos llave en mano completos los que el Estado celebra con el contratista para la realización de una obra que incluye, por lo general, todas las obligaciones inherentes a ella, como son los suministros, el diseño, la construcción y la prestación de servicios.

Se consideran contratos llave en mano parciales los que celebra la entidad contratante con el contratista, de acuerdo con la fusión o combinación de algunas de las obligaciones, como son diseño y/o construcción y/o equipamiento y/o prestación de servicios.

La obligación principal que asume el Estado en los contratos llave en mano es el pago del precio de la obra, previamente negociado con los proponentes y regulado en el pliego de cargos.

En estos contratos, el monto de la fianza de cumplimiento a consignar por el contratista podrá ser de hasta el ciento por ciento (100%) del valor del contrato.

Artículo 21. El artículo 90 de la Ley 22 de 2006 queda así:

Artículo 90. Fianza de recurso de impugnación. La fianza de recurso de impugnación es la garantía que el proponente debe adjuntar al recurso de impugnación, cuando este considere que se han violado sus derechos en un procedimiento de selección de contratista.

Esta fianza será por un monto equivalente al diez por ciento (10%) del valor de la propuesta del impugnante sin exceder de doscientos cincuenta mil balboas (B/.250,000.00) para actos públicos relacionados con adquisición de bienes y servicios, y sin exceder la suma de un millón de balboas (B/.1,000,000.00) para actos relacionados con la realización de obras. La fianza será de cien mil balboas (B/.100,000.00) cuando la impugnación recaiga sobre la decisión en una licitación para convenio marco.

La Dirección General de Contrataciones Públicas preparará las bases de la reglamentación de los aspectos concernientes a esta fianza, la cual será aprobada mediante decreto ejecutivo.

Artículo 22. Se adiciona el artículo 99-A a la Ley 22 de 2006, así:

Artículo 99-A. Rescate administrativo. Cuando se trate de contratos de concesión, la entidad contratante está facultada, por razones de interés público, para ordenar el rescate administrativo de los bienes y las obras dados en concesión, previa autorización del Consejo de Gabinete.

No obstante lo estipulado en el respectivo contrato de concesión, las sumas que el concesionario deba en concepto de impuestos, tasas, multas, recargos o cualesquiera cuentas pendientes con alguna institución del Estado o el Municipio serán descontadas por la entidad concedente privativa y automáticamente del pago por concepto de rescate administrativo que deba recibir el concesionario y aplicadas a los impuestos, tasas, multas, recargos o cuentas pendientes, según corresponda.

En caso de que los bienes que deben revertir a la entidad concedente conforme al respectivo contrato de concesión estén gravados, la entidad concedente deberá retener de la indemnización o compensación por concepto de rescate administrativo que tiene derecho a recibir el concesionario un monto equivalente a las sumas pendientes de pago por las obligaciones garantizadas, según lo certifiquen sus respectivos acreedores, de manera que las garantías correspondientes sean liberadas y la entidad concedente reciba libres de gravámenes los bienes objeto de rescate. Estos montos retenidos correspondientes a las obligaciones garantizadas del concesionario serán cancelados directamente a los acreedores hipotecarios, cuando esté en firme la resolución por la cual se aplica la facultad del Estado de declarar el rescate administrativo de la concesión. Cumplidas las retenciones descritas, se hará el pago al concesionario de cualquier remanente de la indemnización. En ningún caso el Estado cancelará montos de obligaciones garantizadas que no puedan ser satisfechos exclusivamente con la indemnización o compensación reconocida al concesionario.

Artículo 23. Se adiciona el artículo 103-A a la Ley 22 de 2006, así:

Artículo 103-A. Incumplimiento de órdenes de compra en convenio marco. En caso de incumplimiento de órdenes de compra amparadas por un convenio marco, la entidad contratante aplicará el procedimiento de resolución administrativa y la sanción que corresponda será establecida por la Dirección General de Contrataciones Públicas. La decisión que ordena la resolución administrativa del contrato podrá ser recurrida en apelación ante el Tribunal Administrativo de Contrataciones Públicas.

Ejecutoriada la resolución, la Dirección General de Contrataciones Públicas procederá a sancionar al contratista la primera vez con el retiro temporal del Catálogo Electrónico de Productos y Servicios por un periodo de tres meses de todos los productos o servicios incluidos en el convenio marco.

Si la Dirección General de Contrataciones Públicas recibe una segunda resolución administrativa de contrato por incumplimiento debidamente ejecutoriada, contra el mismo contratista, la sanción corresponderá a la inhabilitación por un periodo mínimo de seis meses y un máximo de tres años.

Artículo 24. El artículo 104 de la Ley 22 de 2006 queda así:

Artículo 104. Creación. Se crea el Tribunal Administrativo de Contrataciones Públicas como ente independiente e imparcial, que tendrá jurisdicción en todo el territorio de la República. Este Tribunal tendrá competencia privativa, por naturaleza del asunto, para conocer en única instancia de:

1. El recurso de impugnación contra el acto de adjudicación, la declaratoria de deserción o el acto o resolución por la cual se rechazan las propuestas emitidos por las entidades, en los procedimientos de selección de contratista.
2. El recurso de apelación contra la resolución administrativa del contrato y la inhabilitación del contratista.
3. Las acciones de reclamo no resueltas por la Dirección General de Contrataciones Públicas, dentro del término de cinco días hábiles que esta tiene para resolver.

Artículo 25. El tercer párrafo del artículo 113 de la Ley 22 de 2006 queda así:

Artículo 113. Notificación. ...

Transcurridos dos días hábiles, después de que la entidad contratante haya publicado en el Sistema Electrónico de Contrataciones Públicas "PanamaCompra" y en el tablero de anuncios las resoluciones mencionadas en el presente artículo, se darán por notificadas y el interesado, si se considera agraviado con dicha decisión, podrá interponer el recurso de impugnación que establece esta Ley o el recurso de apelación contra la resolución administrativa del contrato. En las contrataciones menores las notificaciones se darán transcurrido un día hábil después de la publicación en el Sistema Electrónico de Contrataciones Públicas "PanamaCompra" y en el tablero de anuncios del cuadro de cotizaciones.

…

Artículo 26. El artículo 114 de la Ley 22 de 2006 queda así:

Artículo 114. Recurso de impugnación. Todos los proponentes que se consideren agraviados por una resolución que adjudique, declare desierto un acto de selección de contratista o el acto o resolución por la cual se rechazan las propuestas, en el cual consideren que se han cometido acciones u omisiones ilegales o arbitrarias, podrán presentar recursos de impugnación ante el Tribunal Administrativo de Contrataciones Públicas, acompañando las pruebas o anunciándolas al momento de formalizar la impugnación, si las hubiera.

Dicho recurso deberá ser interpuesto en un plazo de cinco días hábiles, contado a partir de la notificación de la resolución objeto de la impugnación, que se surtirá en el efecto suspensivo. En los casos de licitación abreviada, y en los contratos de obra por montos que no excedan los cinco millones de balboas (B/.5,000,000.00) el recurso de impugnación se dará en el efecto devolutivo.

Admitido el recurso, el Tribunal Administrativo de Contrataciones Públicas dará traslado a la entidad correspondiente, la cual deberá remitir un informe de conducta acompañado de toda la documentación correspondiente al acto impugnado, en un término no mayor de cinco días hábiles. Dentro del mismo término, podrá comparecer cualquier persona en interés de la ley o en interés particular para alegar sobre la impugnación presentada. Los que así comparezcan se tendrán como parte única y exclusivamente dentro de esta etapa.

Si el objeto de la impugnación versa sobre aspectos estrictamente jurídicos, el Tribunal pasará sin mayor trámite a resolver dentro de un plazo de diez días hábiles. En caso contrario, abrirá un periodo para practicar las pruebas de hasta diez días hábiles. En ambos casos, el Tribunal podrá decretar de oficio las pruebas que estime necesarias o convenientes.

Vencido el término de pruebas, se podrán presentar alegatos por las partes en un término común de tres días, vencido el cual el Tribunal tendrá un periodo de diez días hábiles para resolver.

En las contrataciones menores los proponentes que se consideren afectados tendrán un plazo de dos días hábiles para presentar el recurso de impugnación, contado a partir de la notificación de la decisión objeto de la impugnación, que se surtirá en el efecto suspensivo.

Todo recurso de impugnación debe ir acompañado de la fianza de recurso de impugnación prevista en el artículo 90.

Artículo 27. Se adiciona el artículo 80-A al Código Fiscal, así:

Artículo 80-A. Si los bienes ocultos debidamente reconocidos y recuperados a favor del Tesoro Nacional, incluyendo los intangibles, se originan o son producto de una concesión, arrendamiento, inversión o cualquiera otra modalidad jurídica contratada con el Estado, la recompensa a que tiene derecho el denunciante investido será sufragada por el denunciado, sin perjuicio de las sumas determinadas a ser recuperadas.

En todo caso, el denunciado está obligado a pagar, en concepto de daños y perjuicios, una indemnización a favor del Estado del quince por ciento (15%) de los montos determinados a ser recuperados o que se recuperen.

En el evento de que el perjuicio de un bien oculto obedezca a sumas de dinero pagadas por el Tesoro Nacional, los montos a recuperar, la recompensa y la indemnización podrán ser retenidos mediante compensación sobre futuros pagos que puedan adeudarse al denunciado.

Artículo 28. El párrafo quinto del literal d del artículo 701 del Código Fiscal queda así:

Artículo 701. ...

d. ...

Los ingresos provenientes de comisiones que reciben por los servicios que se prestan a personas naturales o jurídicas dentro de la Zona Libre de Colón y de otras zonas libres que existan o sean creadas en el futuro, tales como almacenamiento y bodega, arrendamientos y subarrendamientos, movimientos internos de mercancías y carga, servicios de facturación, reempaque y similares, se consideran operaciones locales y, en consecuencia, pagarán el Impuesto sobre la Renta conforme al artículo 699 ó 700 de este Código. Con excepción de los arrendamientos y subarrendamientos, los servicios descritos en este párrafo que surten su efecto en el exterior serán considerados como operaciones exteriores o de exportación. Se excluyen de la aplicación de lo dispuesto en este párrafo, los ingresos provenientes de actividades enunciadas en los literales b, d, h, i, j y k del artículo 60 de la Ley 41 de 2004, modificado por la Ley 31 de 2009.

Artículo 29. El artículo 733 del Código Fiscal queda así:

Artículo 733. Toda persona jurídica que requiera el Aviso de Operación de que trata la Ley 5 de 2007 queda obligada a retener el impuesto de dividendo o cuota de participación del diez por ciento (10%) de las sumas que distribuya a sus accionistas o socios cuando estas sean de fuente panameña y del cinco por ciento (5%) cuando se trate de renta proveniente de:

1. Fuente extranjera.
2. Operaciones exteriores o de exportación.
3. Renta local exenta del Impuesto sobre la Renta contenida en los literales e, f, l y n del artículo 708 del Código Fiscal.

Siempre que una persona jurídica distribuya dividendos o cuotas de participación deberá agotar primero las rentas de fuente panameña antes de repartir dividendos o cuotas de participación sobre las rentas de fuente extranjera, de las operaciones exteriores o de exportación y de la renta local que establece el numeral 3 de este artículo.

En los casos de empresas establecidas o que se establezcan en cualquier zona libre de la República de Panamá pagarán el impuesto de dividendo o cuota de participación a una tarifa fija del cinco por ciento (5%) de las sumas que distribuyan a sus accionistas o socios, independientemente de la fuente de origen.

En la distribución de dividendos o cuotas de participación prevalecerá el régimen fiscal previsto en los tratados o convenios para evitar la doble imposición fiscal que sean suscritos por la República de Panamá con el país de que se trate; en caso de no existir tratados o convenios para evitar la doble imposición fiscal, quedará obligada a retener el impuesto de dividendo o cuota de participación al cinco por ciento (5%) de las utilidades que distribuyan independientemente de la fuente de origen.

En el caso de que no haya distribución de dividendos o de que la suma total distribuida como dividendo o cuota de participación sea menor del cuarenta por ciento (40%) del monto de las ganancias netas del periodo fiscal correspondiente, menos los impuestos pagados por la persona jurídica, esta deberá cubrir el diez por ciento (10%) de la diferencia. En los casos de empresas establecidas o que se establezcan en cualquier zona libre de la República, que no distribuyan dividendos o que la suma total distribuida como dividendo o cuota de participación sea menor del veinte por ciento (20%) del monto de las ganancias netas del periodo fiscal correspondiente, se deberá cubrir el diez por ciento (10%) de la diferencia. Las sumas así retenidas serán remitidas al funcionario recaudador del impuesto dentro de los diez días siguientes a la fecha de retención. Tales deducciones y retenciones serán definitivas.

Las sucursales de personas jurídicas extranjeras pagarán como impuesto el diez por ciento (10%) sobre el ciento por ciento (100%) de su renta gravable obtenida en Panamá, menos los impuestos pagados por esa misma renta en el país. Esta retención tendrá carácter definitivo y se pagará conjuntamente con la presentación de la declaración jurada correspondiente.

Las personas jurídicas no estarán obligadas a hacer la retención de que trata este artículo sobre la parte de sus rentas que provenga de dividendos, siempre que las personas jurídicas que distribuyan tales dividendos hayan pagado el impuesto correspondiente y hayan hecho la retención de que trata este artículo.

Las personas jurídicas tampoco estarán obligadas a hacer la retención de que trata este artículo sobre la parte de sus rentas que provengan de dividendos, siempre que las personas jurídicas que distribuyan tales dividendos también hayan estado exentas de la obligación de hacer la retención.

Toda persona natural o jurídica que deba remitir a una persona natural o jurídica no residente en la República de Panamá sumas provenientes de rentas de cualquier clase producidas en el territorio panameño, excepto dividendos o participaciones, deberá deducir y retener, al momento de remitir dichas sumas en cualquier forma, la cantidad que establece el artículo 699 ó 700 de este Código y entregará lo así retenido al funcionario recaudador del impuesto dentro de los diez días siguientes a la fecha de retención.

Para calcular el monto de la retención, deberán sumarse al monto que se pague, gire o acredite las sumas que se hubieran pagado, girado, acreditado o abonado al contribuyente durante el año y sobre el cincuenta por ciento (50%) de este total se aplicará la tasa del artículo 699 ó 700 de este Código. Del importe así establecido se deducirán las retenciones ya efectuadas en el año gravable.

PARÁGRAFO. No obstante lo dispuesto en este artículo, los tenedores de las acciones al portador pagarán este impuesto a la tasa del veinte por ciento (20%).

La persona jurídica que distribuya tales dividendos practicará la retención, la que tendrá carácter definitivo. En caso de que la sociedad que distribuya dividendos tenga diferentes clases de acciones, el impuesto se pagará de conformidad con las tasas aquí establecidas y según el tipo de acciones.

Cuando la distribución sea menor del cuarenta por ciento (40%) de las ganancias netas o en caso de que no haya distribución, se aplicarán las disposiciones del impuesto complementario, con independencia del tipo de acciones que haya emitido la sociedad.

Las personas naturales o jurídicas establecidas en el Área Económica Especial Panamá-Pacífico quedan sujetas a la obligación dispuesta en este artículo, salvo las dedicadas a las actividades enunciadas en los literales del artículo 60 de la Ley 41 de 2004, modificado por la Ley 31 de 2009.

Artículo 30. El numeral 7 del artículo 13 de la Ley 5 de 1988 queda así:

Artículo 13. Los concesionarios están obligados a lo siguiente:

…

7. No traspasar, disponer o gravar las mejoras que construya el concesionario, sin previo consentimiento del Consejo Económico Nacional para montos de hasta tres millones de balboas (B/.3,000,000.00) y del Consejo de Gabinete para montos mayores a esta suma.

Artículo 31. El numeral 2 del artículo 2 de la Ley 4 de 1994 queda así:

Artículo 2. ...

2.

Los préstamos interbancarios, los préstamos garantizados con depósitos bancarios, los préstamos externos, el financiamiento a través de la emisión de bonos y valores, debidamente registrados ante la Comisión Nacional de Valores, así como los préstamos concedidos a las entidades financieras reguladas por la Ley 42 de 2001. La presente Ley tiene efectos sobre los créditos garantizados con depósitos bancarios que se hayan generado a partir de la entrada

en vigencia de la Ley 49 de 2009, y se ordena a las instituciones bancarias devolver la tasa del FECI que haya sido aplicada desde el 18 de septiembre de 2009 sobre los créditos garantizados con depósitos bancarios.

…

Artículo 32. El artículo 28 de la Ley 45 de 1995 queda así:

Artículo 28. La tarifa del impuesto selectivo al consumo de cigarrillos será del ciento por ciento (100%) del precio de venta al consumidor declarado por el productor nacional o el importador al Ministerio de Economía y Finanzas, con un mínimo de un balboa con cincuenta centavos (B/.1.50) por cajetilla.

La tarifa del impuesto selectivo al consumo de tabacos, habanos y otros productos derivados del tabaco será del ciento por ciento (100%) del precio de venta al consumidor declarado por el productor nacional o el importador al Ministerio de Economía y Finanzas.

El cincuenta por ciento (50%) del importe recaudado de los impuestos establecidos en este artículo se destinará y distribuirá directamente de la siguiente manera:

1. Un cuarenta por ciento (40%) al Instituto Oncológico Nacional.
2. Un cuarenta por ciento (40%) al Ministerio de Salud para que sea invertido en actividades de prevención y tratamiento de enfermedades producto del consumo de tabaco, a través de clínicas de cesación.
3. Un veinte por ciento (20%) a la Autoridad Nacional de Aduanas para que sea invertido en actividades destinadas a la prevención y persecución del contrabando de productos derivados del tabaco.

Artículo 33. El numeral (4) del literal (f) del artículo 71 del Decreto-Ley 2 de 1998 queda así:

Artículo 71. Toda persona que solicite un Contrato de Operación y Administración a la Junta de Control de Juegos deberá:

...

(f). Suministrar la información que pueda ser requerida por la Junta de Control de Juegos, incluyendo pero no limitada a la siguiente:

...

(4). Los nombres de todos los accionistas del administrador operador, entendiéndose que en ningún caso se aceptarán acciones emitidas al portador por parte del administrador operador ni de las posibles personas jurídicas dueñas de las acciones del administrador operador, sin importar que se trate de accionistas minoritarios.

...

Artículo 34. El artículo 5 del Decreto-Ley 7 de 1998 queda así:

Artículo 5. Constituyen el patrimonio de la Autoridad:

1. Todos los bienes muebles e inmuebles que a la fecha pertenezcan a todas las dependencias de la administración pública que, por razón del presente Decreto Ley, pasan a formar parte de la Autoridad.
2. Las herencias, donaciones y legados que se le transmitan, los cuales se recibirán a beneficio de inventario.
3. El producto de las acciones, obligaciones, títulos y demás valores que posea.
4.

Las subvenciones que reciba del Estado.

5.

Las tasas, tarifas, derechos y contribuciones que perciba como resultado de los servicios que preste y los ingresos que provengan de la gestión directa o de las concesiones que otorgue.

Parágrafo. Las tasas, tarifas, derechos y contribuciones, fijados o determinados por la Autoridad Marítima de Panamá, por el cobro o retención de servicios públicos o nacionales y por derechos de las concesiones que otorgue serán administrados, recaudados y cobrados exclusivamente por la Autoridad Marítima de Panamá.

En consecuencia, se deja sin efecto cualquier norma, reglamento, acuerdo o acto administrativo contrario a esta disposición, salvo los contratos-leyes suscritos por el Estado.

6.

El producto de las sanciones pecuniarias impuestas por la Autoridad.

7.

Cualesquiera otros bienes o haberes que autoricen las disposiciones legales, los reglamentos o la Junta Directiva.

Artículo 35. Se deroga el literal c del artículo 60 de la Ley 41 de 2004, modificado por la Ley 31 de 2009.

Artículo 36. El numeral 32 del artículo 5 de la Ley 56 de 2008 queda así:

Artículo 5. Para los efectos de la aplicación de la presente Ley y sus reglamentos, se establecen las siguientes definiciones:

…

32. *Movimiento.* Transferencia de una unidad de carga que involucra cruzar los bordes laterales de la nave, ya sea en la carga o en la descarga, tanto por el lado del muelle como el lado del agua, sin importar que la carga tenga como destino el territorio de la República de Panamá o para el comercio exterior.

...

Artículo 37. Se adicionan dos definiciones al artículo 5 de la Ley 56 de 2008, así:

Artículo 5. Para los efectos de la aplicación de la presente Ley y sus reglamentos, se establecen las siguientes definiciones:

...

Afiliada. Se entenderá como empresa afiliada de la empresa concesionaria la que, aun cuando mantenga su personalidad individual, se dedique dentro del área del proyecto a las mismas actividades a las que se dedica la empresa concesionaria, o a actividades complementarias y relativas a la operación de la terminal, siempre que la afiliada sea una persona jurídica dirigida o controlada económica, financiera o administrativamente, de manera directa o indirecta, por la empresa concesionaria.

Subsidiaria. Se entenderá como empresa subsidiaria de la empresa concesionaria la que, aun cuando mantenga su personalidad individual, se dedique dentro del área del proyecto a las mismas actividades a las que se dedica la empresa concesionaria o a actividades complementarias y relativas a la operación de la terminal, siempre que la subsidiaria sea una persona jurídica dirigida o controlada económica, financiera o administrativamente, de manera directa o indirecta, por la empresa concesionaria.

Artículo 38. El artículo 64 de la Ley 56 de 2008 queda así:

Artículo 64. La Autoridad Marítima de Panamá considerará, entre otras, las siguientes tarifas fijas o variables por el derecho de uso de la concesión, derecho de uso de bienes de instalaciones portuarias y Licencias de Operación:

1.

Tarifa por movimiento.

2.

Tarifa por manejo de carga a granel.

3.

Tarifa por prestación de servicios marítimos, dependiendo del servicio autorizado.

4.

Tarifas por servicio prestado a las naves, como uso del canal de navegación, fondeo en áreas marítimas, faros y boyas, siempre que estos servicios no estén siendo pagados a otras entidades del Estado.

5.

Tarifas por muellaje.

6.

Tarifa de inspección o supervisión de áreas a ser incorporadas en la concesión.

7.

Tarifa por arribo de carga local contenerizada destinada al territorio de la República de Panamá.

Parágrafo. Los operadores portuarios, ya sean concesionarios privados o entidades estatales, cobrarán la tarifa por arribo de carga local contenerizada destinada al territorio de la República de Panamá, por el uso de las instalaciones portuarias, como un servicio que presta el Estado. Esta tarifa será por cuenta de los consignatarios de la carga local contenerizada y los operadores portuarios remitirán el cargo correspondiente a la Autoridad Marítima de Panamá.

Artículo 39. Se adiciona el artículo 64-A a la Ley 56 de 2008, así:

Artículo 64-A. Las tasas, tarifas, derechos y contribuciones, fijados o determinados por la Autoridad Marítima de Panamá, por el cobro o retención de servicios públicos o nacionales y por derechos de las concesiones que otorgue serán administrados, recaudados y cobrados exclusivamente por la Autoridad Marítima de Panamá.

Queda sin efecto cualquier norma, reglamento, acuerdo o acto administrativo contrario a esta disposición, salvo los contratos-leyes suscritos por el Estado.

Artículo 40. El artículo 65 de la Ley 56 de 2008 queda así:

Artículo 65. Para los efectos del método de facturación, los concesionarios y los proveedores de servicios remitirán a la Autoridad Marítima de Panamá informes claros y completos con la indicación de la cantidad de movimientos de carga y descarga, así como los servicios prestados durante el periodo respectivo.

La Autoridad Marítima de Panamá proporcionará el formato de facturación e indicará la periodicidad con la que debe suministrarse dicha información y con fundamento en las inspecciones que realice, conforme a lo establecido en el numeral 4 del artículo 23 de esta Ley y en los informes remitidos, elaborará la factura correspondiente, la cual será remitida al concesionario o proveedor de servicios.

Los operadores portuarios deberán someterse a auditorías, arqueos e inspecciones por auditores externos, con el objeto de que se verifique el reporte de los movimientos que pagan a la Autoridad Marítima de Panamá. Esta revisión será efectuada, por lo menos una vez al año, por una firma auditora externa, contratada por los operadores portuarios y aprobada por la Autoridad Marítima de Panamá, la cual verificará el número de movimientos realizados y pagados al Estado. Igualmente, la Autoridad podrá verificar esta información a través de la Dirección de Auditoría Interna y Fiscalización de la institución o del cotejo de la información que maneja el Sistema Integrado de Comercio Exterior, la Autoridad del Canal de Panamá, la Autoridad Nacional de Aduanas o cualquiera otra institución del Estado.

Artículo 41. El artículo 66 de la Ley 56 de 2008 queda así:

Artículo 66. Para los efectos del pago a la Autoridad Marítima de Panamá, cuando la carga sea transbordada o transferida de un contenedor en el ciclo de descarga y carga, de una nave a otra nave dentro del mismo recinto portuario, los movimientos que se realicen en la descarga de una nave y la posterior carga hacia otra serán facturados como un solo movimiento, siempre que la carga no tenga como destino final el territorio de la República de Panamá.

Artículo 42. Se deroga el artículo 120 de la Ley 56 de 2008.

Artículo 43. La presente Ley es de interés social y tendrá efectos retroactivos sobre las contrataciones otorgadas por el Estado, cuyas tarifas, exenciones, términos y condiciones hayan sido modificados sobre la base del concepto de equiparación, en detrimento del Estado.

Estas contrataciones deberán someterse a un proceso de revisión conforme al procedimiento que para tales efectos se establezca mediante decreto ejecutivo. Se excluye de la aplicación de este artículo a la Ley 56 de 2008.

Artículo 44. La presente Ley modifica el numeral 46 del artículo 2, los artículos 14, 20, 31, 38 y 39, el numeral 14 y el último párrafo del artículo 40, el numeral 13 y el último párrafo del artículo 41, el numeral 2 del artículo 44, los artículos 58, 84, 90 y 104, el tercer párrafo del artículo 113 y el artículo 114 de la Ley 22 de 27 de junio de 2006; el párrafo quinto del literal d del artículo 701 y el artículo 733 del Código Fiscal; el numeral 7 del artículo 13 de la Ley 5 de 15 de abril de 1988, el numeral 2 del artículo 2 de la Ley 4 de 17 de mayo de 1994, el artículo 28 de la Ley 45 de 14 de noviembre de 1995, el numeral 4 del literal f del artículo 71 del Decreto-Ley 2 de 10 de febrero de 1998, el artículo 5 del Decreto-Ley 7 de 10 de febrero de 1998 y el numeral 32 del artículo 5 y los artículos 64, 65 y 66 de la Ley 56 de 6 de agosto de 2008.

Adiciona el numeral 49 al artículo 2, un párrafo final al artículo 11, el numeral 15 al artículo 12, el artículo 24-A, los artículos 43-A y 72-A, un párrafo al artículo 75 y los artículos 99-A y 103-A a la Ley 22 de 28 de diciembre de 2006, el artículo 80-A al Código

Fiscal, así como dos definiciones al artículo 5 y el artículo 64-A a la Ley 56 de 6 de agosto de 2008.

Deroga el literal c del artículo 60 de la Ley 41 de 21 de noviembre de 2004, modificado por la Ley 31 de 22 de junio de 2009, y el artículo 120 de la Ley 56 de 6 de agosto de 2008.

Artículo 45. Esta Ley comenzará a regir el día siguiente al de su promulgación.

COMUNÍQUESE Y CÚMPLASE.

Proyecto 1 de 2009 aprobado en tercer debate en el Palacio Justo Arosemena, ciudad de Panamá, a los 31 días del mes de octubre del año dos mil nueve.

El Presidente,

José Luis Varela R.

El Secretario General,

Wigberto E. Quintero G.

ÓRGANO EJECUTIVO NACIONAL. PRESIDENCIA DE LA REPÚBLICA.
PANAMÁ, REPÚBLICA DE PANAMÁ, 6 DE noviembre DE 2009.

RICARDO MARTINELLI B.
Presidente de la República

FRANK DE LIMA
Ministro de Economía y Finanzas,
encargado
