# Supplementary material for: The Association of Tobacco Control Policies and the Risk of Acute Myocardial Infarction Using Hospital Admissions Data
Source: PLoS One. 2014 Feb 10;9(2):e88784. doi: 10.1371/journal.pone.0088784 (PMC3919809; doi:10.1371/journal.pone.0088784)
Supplement: Document S5 — PREFREC 2010-Tobacco Consumption Prevalence in Panama. (PDF) [file pone.0088784.s006.pdf]

CAPÍTULO 5

CONSUMO DE PRODUCTOS DE TABACO

La prevalencia de vida de productos del tabaco como cigarrillos, puros o pipas se refiere a si alguna vez en la vida el individuo ha fumado por lo menos uno (1) de estos productos. En ese sentido, en esta investigación se encontró en la muestra en estudio una prevalencia de vida para el consumo de productos de tabaco de 29.2% (IC95%=27.7-30.7). De todos los hombres que participaron (1074), la prevalencia de vida fue de 58.0%, en tanto que del total de las mujeres (2516) fue de 16.9% (424), con diferencias estadísticamente significativas entre hombres y mujeres (x^2=615.13; p=0.0000). El 42.0% de los hombres manifestó no haber fumado nunca en la vida, al igual que el 83.1% de las mujeres.

Del total de las personas entrevistadas en el área urbana (1688), 3 de cada 10 (507) habían fumado algún producto de tabaco alguna vez en su vida; el 27.6% (469) de los encuestados en el área rural (1699) también lo hicieron, así como el 35.0% (71) de las 203 personas que residían en el área indígena, con diferencias estadísticamente significativas entre el área rural-indígena (x^2=4.49; p=0.034). No se encontraron diferencias estadísticamente significativas entre las áreas urbana-rural y urbano-indígena.

De acuerdo a la clasificación de la adultez, de los individuos entre 18 – 29 años o adultos jóvenes (735), el 21.8% manifestó haber fumado algún producto de tabaco alguna vez en su vida. La misma condición fue reportada por el 26.1% de los adultos (2064) y por el 44.1% de los adultos mayores (791), encontrando diferencias estadísticamente significativas para el comportamiento de estos indicadores entre la condición de adultos-adultos jóvenes (x^2=5.12; p=0.0237); adultos - adultos mayores (x^2=86.21; p=0.0000) y adultos jóvenes – adultos mayores (x^2=84.63; p=0.0000).

El análisis según nivel de escolaridad refleja que de las 1047 personas que indicaron haber fumado alguna vez en su vida, el 37.0% tenía la educación primaria como máximo nivel académico alcanzado, el 34.7% manifestó que la educación secundaria, el 14.9% reportó tener estudios universitarios, en tanto que el 3.0% y el 1.7% habían alcanzado el nivel técnico-primer año de universidad y estudios de post-grado, respectivamente. El 7.8% (82) nunca asistió a la escuela. No especificó su respuesta el 1.0% (10 individuos). El resultado de las pruebas de hipótesis entre las personas que habían fumado alguna vez en su vida y su nivel de escolaridad indica diferencias estadísticamente significativas para las siguientes relaciones:

- Sin escolaridad (nunca asistió) – primaria: x^2=8.97; p=0.0027.
- Primaria – secundaria: x^2=22.60; p=0.000002.
- Universidad – secundaria: x^2=6.34; p= 0.0118

Según el estado conyugal, cerca de 7 de cada 10 entrevistados (67.7% o 709) que habían fumado alguna vez en su vida estaban unidos o casados, mientras que el 32.0% (335) no lo estaban (sin diferencias estadísticamente significativas), siendo el 62.1% solteros, 19.4% viudos y un 18.5% separados o divorciados. El 0.3%(3) no especificó su respuesta.

En cuanto al medio sociocultural de los encuestados que habían fumado alguna vez en su vida, 1 de cada 2 se identificó como mestizaje (54.3%), 2 de cada 10 (20.1%) afroamericano, 1 de cada 10 (12.3%) manifestó ser blanco, el 11.1% indicó ser indígena y el 1.0% (10) asiático. La opción de respuesta “otros” fue indicada por el 1.1% (12), en tanto que una persona (0.1%) no especificó su respuesta. A pesar de que este fue el comportamiento según medio sociocultural de los 1047 individuos con historia de tabaquismo alguna vez en su vida, llama la atención que la prevalencia de vida específicamente entre los medios socioculturales fue mayor entre los asiáticos 37.0%. El mestizaje, afroamericano, indígena, blancos y otros, presentaron prevalencias de vida similares al comportamiento de la muestra (29.4%, 27.7%, 29.7%, 29.9%, 27.3%, respectivamente).

El ingreso familiar mensual en las personas que habían fumado alguna vez en su vida reflejó que 4 de cada 10 encuestados (42.8%, es decir, 448 personas) tiene un ingreso familiar mensual inferior a B/. 250.00. El 36.8% (385 individuos) indicó que su ingreso estaba entre B/. 250.00 – B/. 600.00. Un 8.8% (92 encuestados) manifestó un ingreso familiar entre B/. 601.00 – B/. 999.99, en tanto que el 8.4% (88) reportó un ingreso superior a B/.1000.00. Tres personas indicaron no estar trabajando (0.3%), mientras que el 3.0% (31) dijo que no sabe.

Del 29.2% (1047) de los individuos que sí han consumido productos del tabaco alguna vez en su vida, 1 de cada 4 (25.2%) residía en la RS de Colón, así como el 25.0% (262) que vivía en la RS Metropolitana. El 19.5% pertenecía a la RS de Panamá Este, un 17.6% a la RS de Panamá Oeste, en tanto que el 12.7% a la RS de San Miguelito. Estos datos permiten estimar prevalencias de vida de consumo de productos de tabaco según RS, como lo muestra la gráfica No.1.

Gráfica No.1

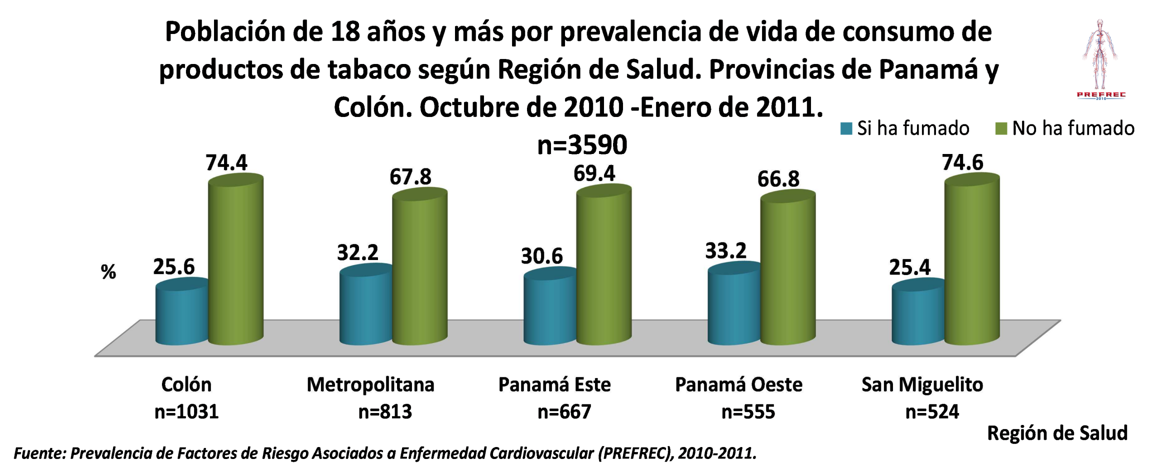

Fuente: Prevalencia de Factores de Riesgo Asociados a Enfermedad Cardiovascular (PREFREC), 2010-2011.

Edad de inicio de consumo de productos de tabaco

Al preguntarle a los 1047 entrevistados que habían consumido productos de tabaco alguna vez en su vida, ¿qué edad tenían cuando fumaron por primera vez?, un 45.6% (477) indicó tener menos de 18 años, en tanto que el 54.3% (568) manifestó que cuando fumó por primera vez ya era mayor de edad (18 años o más). Dos personas (0.2%) no especificaron su respuesta.

De acuerdo al género, de los 623 hombres y 424 mujeres que indicaron haber fumado alguna vez en su vida, el 50.6% (315) y un 38.2% (162), respectivamente, lo hicieron antes de los 18 años de edad. El 49.3% (307) de los hombres fumadores y el 61.6% (261) de las mujeres fumadoras iniciaron su consumo luego de tener la mayoría de edad (18 años), con diferencias estadísticamente significativas (x^2=14.97; p=0.0001).

De acuerdo a las áreas de dominio de esta encuesta, de las 507 personas que manifestaron haber fumado alguna vez en su vida en el área urbana, el 45.4% (230) inició el consumo de productos de tabaco cuando tenía menos de 18 años, mientras que el 54.4% (276) lo hizo al tener 18 años y más. Una persona no especificó su respuesta (0.2%). En el área rural, el 45.6% de los 469 individuos que reportaron el consumo de productos de tabaco alguna vez en su vida lo hicieron antes de los 18 años y el 54.2% (254) lo hizo siendo mayor de edad; una persona (0.2%) no especificó su respuesta. Para el área indígena (n=203), el 46.5% inició el consumo de estos productos con edades inferiores a los 18 años, en tanto que el 53.5% lo hizo después de haber cumplido los 18 años de edad, sin diferencias estadísticamente significativas entre las áreas.

La edad promedio inicio de consumo de productos de tabaco fue 18.8 años de edad, mientras que la mediana fue de 18 años.

El análisis según clasificación de la adultez refleja que del 15.3% (160) de los adultos jóvenes que habían fumado alguna vez en su vida, el 52.5% (84) tenía menos de 18 años cuando fumó por primera vez y el 47.5% (76) era mayor de edad. En el caso de los 538 adultos fumadores, 4 de cada 10 (43.7% o 235 personas) eran menores de edad cuando fumaron por primera vez, mientras que el 55.9% (301) tenían 18 años o más; dos adultos no especificaron su respuesta (0.4%). Del 33.3% de los adultos mayores fumadores (349), 4 de cada 10 (158 individuos) iniciaron el consumo de productos de tabaco antes de los 18 años de edad, en tanto que 5 de cada 10 (54.7%) lo hicieron con 18 años o más de edad. Hubo diferencias estadísticamente significativas entre la condición de adulto joven – adulto mayor y la edad de inicio de consumo de productos de tabaco (x^2=22.99; p=0.0000016), mas no para la condición de adulto-adulto joven y adulto-adulto mayor.

De los individuos que iniciaron el consumo de productos de tabaco antes de los 18 años (477), cerca de 4 de cada 10 encuestados (37.1%; 177 individuos) manifestó haber cursado solamente el nivel educativo primario, en tanto que 3 de cada 10 (34.4%, es decir 164 de individuos) indicaron tener estudios correspondientes a la escuela secundaria. El nivel de formación técnica o haber asistido solo hasta el primer año de la universidad fue

reportado solo por el 3.4% (16). La educación universitaria (a excepción del primer año) fue alcanzada por el 13.8% (66), mientras que el 1.5% (7) indicó tener estudios de post grado. Un 9.2% (44) nunca asistió a la escuela. No especificaron su respuesta el 0.6% de las personas (3).

Un comportamiento similar en el nivel de escolaridad de las personas que iniciaron el consumo de productos de tabaco antes de los 18 años se encontró entre los individuos que empezaron a fumar con 18 años o más, tal como se muestra en la gráfica No. 2. De las dos personas que no especificaron la edad de inicio de fumar, una nunca asistió a la escuela y la otra tenía un nivel de educación secundaria.

Gráfica No.2

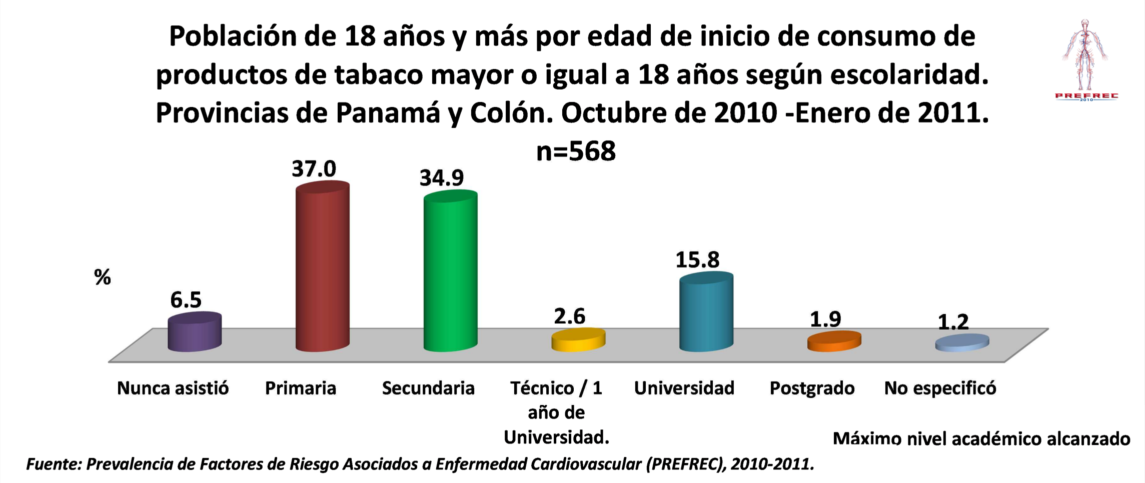

El análisis general según RS, indica que de las 264 personas que manifestaron haber fumado alguna vez en su vida en la RS de Colón, 42.4% era menor de 18 años cuando fumó por primera vez y el 57.6% tenía una edad igual o mayor de 18 años. Porcentajes similares predominaron en el resto de las RS, estimándose relaciones entre la edad de inicio de consumo de productos de tabaco igual o mayor a 18 años respecto a la de menor a 18 años de 1.3:1, 1.2:1 y 1.2:1 para las RS Metropolitana, Panamá Oeste y San Miguelito. En la RS de Panamá Este, el comportamiento fue inverso al resto de las Regiones Sanitarias, donde de los individuos que reportaron haber fumado alguna vez en su vida (204), 1 de cada 2 (51.5%) lo hizo por primera vez antes de los 18 años y el 48.5% restante siendo mayor de edad.

Consumo de tabaco por más de 10 años en las personas con historia de tabaquismo alguna vez en su vida.

De los 1047 entrevistados que indicaron haber fumado alguna vez en su vida, el 14.5% de los fumadores (152) tenían menos de 10 años de estar consumiendo o haber consumido productos de tabaco, en tanto que 8 de cada 10 (85.3%) reportaron tener más de 10 años de consumo. Un 0.2% (2 personas) no especificó su respuesta.

Del total de hombres (623) que señalaron fumar alguna vez en su vida, 1 de cada 10 (12.5% ó 78 individuos) tenía menos de 10 años de estar consumiendo o haber consumido productos de tabaco, en tanto que cerca de 9 de cada 10 (87.3%) reportaron tener más de 10 años de estar fumando. Un 0.2% (1 hombre) no especificó su respuesta.

En el caso de las mujeres con historia de tabaquismo alguna vez en su vida (424), el 17.5% (74) había fumado en los últimos 10 años. Ocho de cada 10 (82.3%) reportó que había fumado por más de 10 años. Una mujer no especificó su respuesta. Las pruebas de significancia aplicadas a la condición de género y el consumo de productos de tabaco por 10 años o menos y más de 10 años reflejaron diferencias estadísticamente significativas (x^2=4.58; p=0.0323).

De las personas que habían fumado alguna vez en su vida encontramos que en el área urbana (507), el 15.2% (77) mencionó que tenían 10 años o menos de estar consumiendo o haber consumido productos de tabaco, al contrario de 8 de cada 10 personas en la misma área (84.6%), que indicaron tener más de 10 años de estar fumando. Un 0.2% (1 individuo) no especificó su respuesta.

Entre los entrevistados que manifestaron historia de tabaquismo alguna vez en la vida en el área rural (469), el 14.3% (67) indicó tener 10 años o menos de estar fumando. El 85.5% respondió tener más de 10 años de estar consumiendo productos de tabaco. Un 0.2% (1 individuo) no especificó su respuesta.

Uno de cada 10 (11.3%) de los individuos que fumaron alguna vez en su vida y que vivían en el área indígena (71) señalaron tener 10 años o menos de estar fumando, en tanto que cerca de 9 de cada 10 (88.7%) reportaron el consumo por más de 10 años.

Consumo diario de productos de tabaco en las personas con historia de tabaquismo alguna vez en su vida.
Se formuló una pregunta para conocer si las personas que fuman o fumaban lo hacían a diario. En ese sentido se encontró que de las 1047 personas que indicaron haber fumado alguna vez en su vida, el 44.6% (467) fuma o fumaba a diario.

Del total de hombres encuestados que indicaron haber fumado alguna vez en su vida (623), el 49.6% (309) lo hacía a diario; en tanto que cerca de 4 de cada 10 del total de mujeres (424) que reportaron el historial de tabaquismo alguna vez en su vida, también fumaban a diario (37.3%, es decir 158), presentando diferencias estadísticamente significativas entre género (x^2=15.04; p=0.0001).

En el área urbana, de las 507 personas que reportaron fumar alguna vez en la vida, el 44.0% lo hacía a diario. Esta condición también se presentó en el 45.8% de los 469 individuos consumidores de productos de tabaco en el área rural, en tanto que de los 71 entrevistados residentes en el área indígena que fumaron alguna vez en la vida, 40.8%

también lo hacía a diario. No se encontraron diferencias estadísticamente significativas entre las áreas.

De los 160 adultos jóvenes que habían fumado alguna vez en su vida, el 21.3% (34) fumaba a diario. En el caso de los 538 adultos fumadores, 4 de cada 10 (41.9% ó 224 individuos) también fumaban a diario. La condición de fumar a diario fue mayor entre los adultos mayores que manifestaron fumar alguna vez en su vida (349), donde cerca de 6 de cada 10 entrevistados (59.4% ó 209 personas) lo hacía. Hubo diferencias estadísticamente significativas entre la condición de adulto – adulto joven y haber fumado a diario (x^2=21.13; p=0.0000043), adulto – adulto mayor (x^2=27.49; p=0.0000002) y para la condición adulto mayor – adulto joven (x^2=64.10; p=0.0000).

El análisis entre las personas que fumaban a diario (467) y el máximo nivel de escolaridad indica que el mayor porcentaje de fumadores a diario alcanzaron solo el nivel primario (43.7%), cerca de 3 de cada 10 (29.6%) el nivel secundario, en tanto que 1 de cada 10 (10.9%) nunca asistió a la escuela y un 10.7% tenía nivel universitario. El 3.2% había culminado estudios técnicos o solo realizó el primer año de universidad, un 0.4% (2) realizó estudios de postgrado, mientras que 7 personas (1.5%) no especificaron su respuesta.

Al evaluar a los individuos que fumaban a diario en las RS encontramos que de las personas que indicaron haber fumado alguna vez en su vida:

- En la RS de Colón (n=264), el 49.2% (130) fumaba a diario.
- El 43.1% (113) de los 262 individuos que fumaron alguna vez en la vida en la RS Metropolitana, también fumaba a diario.
- En el caso de la RS de Panamá Este, el 47.1% de las 204 personas que manifestaron haber fumado alguna vez en su vida, lo hacían a diario.
- La RS de Panamá Oeste reportó el menor porcentaje de entrevistados que fumaban a diario (32.1%) respecto a las 184 personas con historial de tabaquismo alguna vez en su vida.
- La RS de San Miguelito presentó la mayor cantidad de individuos que fumaban a diario, donde 1 de cada 2 encuestados (51.9%) que habían consumido productos de tabaco alguna vez en su vida (133), fumaban a diario.

Edad de inicio de consumo de productos de tabaco a diario

A los entrevistados que manifestaron fumar a diario, se les preguntó ¿qué edad tenían cuando empezaron a fumar a diario? De los 467 individuos que respondieron que fuman o fumaban a diario, el 1.7% (8) inició entre los 6 – 9 años de edad, 1 de cada 2 (54.8%, es decir, 256 personas) tenían entre 10 y 19 años cuando empezaron a fumar a diario cigarrillos, pipas o puros. Uno de cada tres (33.2%) reportó que entre los 20 – 29 años empezó a fumar a diario. El 10.1% indicó tener entre 30 y 59 años cuando inició el consumo de productos de tabaco a diario (7.7% entre los 30 – 39 años, 2.1% entre los 40 – 49 años y un 0.2% (1) entre los 50 a 59 años). Una persona no especificó su respuesta (0.2%).

El análisis respecto a la totalidad de hombres y mujeres que participaron en este estudio y que habían fumado alguna vez en la vida, indica que el 28.8% (309) de los hombres y el 6.3% de las mujeres (158) fumaban a diario.

En el caso de los hombres el 59.5% (184) inició el consumo entre los 6 y 19 años de edad (1.6% entre los 6 - 9 años y un 57.9% entre los 10 – 19 años). Un 33.0% (102) inició el consumo de productos de tabaco a diario entre los 20 – 29 años, el 6.1% (19) entre los 30 – 39 años y un 1.3% (4) entre los 40 y 49 años.

En cuanto a las mujeres, el 1.9% (3) empezó a fumar a diario cuando tenía entre 6 a 9 años de edad. La mayor cantidad de fumadoras inició el consumo a diario con edades comprendidas entre los 10 y 19 años (48.7%), mientras que 1 de cada 3 (33.5%) lo hizo entre los 20 y 29 años de edad. Un 15.8% (25) inició el consumo de productos de tabaco a diario a partir de los 30 años de edad (10.8% entre los 30 - 39 años, 3.8% entre los 40 a 49 años y un 0.6% entre los 50 y 59 años). Una mujer no especificó su respuesta.

Del total de las personas residentes en el área urbana (1688), el 13.2% (223) fumaba a diario, al igual que el 12.7% (215) del total de los habitantes del área rural (1699) y el 14.3% (29) del total de los entrevistados en el área indígena (203), los cuales totalizaron los 467 individuos que fumaban a diario.

De los 223 individuos que indicaron fumar a diario en el área urbana, el 1.3% (3 personas) empezó a fumar a diario cuando tenía entre 6 a 9 años de edad. La mayor cantidad de estos fumadores inició el consumo a diario con edades comprendidas entre los 10 y 19 años (54.7%), mientras que cerca de 1 de cada 3 (32.7%) lo hizo entre los 20 y 29 años de edad. Un 11.2% (25 personas) inició el consumo de productos de tabaco a diario a partir de los 30 años de edad (8.5% entre los 30 - 39 años y un 2.7% entre los 40 a 49 años).

En el área rural, de los 215 entrevistados que fumaban a diario, el 56.7% (112) inició el consumo entre los 6 y 19 años de edad (2.3% entre los 6 - 9 años y un 54.4% entre los 10 – 19 años). Uno de cada 3 individuos (33.5%) inició el consumo de productos de tabaco a diario entre los 20 – 29 años, el 7%(15) entre los 30 – 39 años, un 1.9% (4) entre los 40 y 49 años, mientras que el 0.5% (1) lo hizo entre los 50 – 59 años. Un encuestado no especificó su respuesta.

El comportamiento encontrado en el área urbana y rural, no dista de lo que respondieron los encuestados en el área indígena, donde de 29 individuos que manifestaron fumar a diario, el 58.6% (17) lo hizo entre los 10 y 19 años, 1 de cada 3 (34.5%) entre los 20 y 29 años, mientras que el 6.9% (2) indicó haber iniciado el consumo de productos de tabaco entre los 30 y 39 años.

En cuanto a la clasificación de la adultez, de los 34 adultos jóvenes que indicaron fumar a diario el 88.2% inició entre los 10 y 19 años, mientras que el 11.8% lo hizo entre los 20 a 29 años.

El 48.0% de los individuos que fumaban a diario eran adultos (224), de los cuales el 0.9% inició a fumar a diario entre los 6 y 9 años, 1 de cada 2 (53.6%) lo hizo entre los 10 y 19 años, 1 de cada 3 (33.9%) empezó a fumar a diario con edades entre los 20 y 29 años, un 9.4% inició entre los 30 y 39 años, mientras que el 2.2% manifestó empezar a fumar a diario entre los 40 y 49 años.

De lo 209 adultos mayores que fumaban a diario, el 2.9% inició entre los 6 – 9 años de edad, 1 de cada 2 (50.7%) tenía entre 10 y 19 años cuando empezaron a fumar a diario cigarrillos, pipas o puros. Un 35.9% reportó que entre los 20 – 29 años empezó a fumar a diario. El 10.0% indicó tener entre 30 y 59 años cuando inició el consumo de productos de tabaco a diario (7.2% entre los 30 – 39 años, 2.4% entre los 40 – 49 años y un 0.5% entre los 50 a 59 años). Una persona no especificó su respuesta (0.5%).

Para todas las RS que formaron parte de este estudio, la mayor cantidad de los fumadores (54.8% en promedio), inició el consumo a diario con edades comprendidas entre los 10 y 19 años, mientras que 1 de cada 3 (33.2% en promedio) lo hizo entre los 20 y 29 años de edad, representando porcentajes superiores a 80 por cada RS (gráfica No.3).

Gráfica No.3

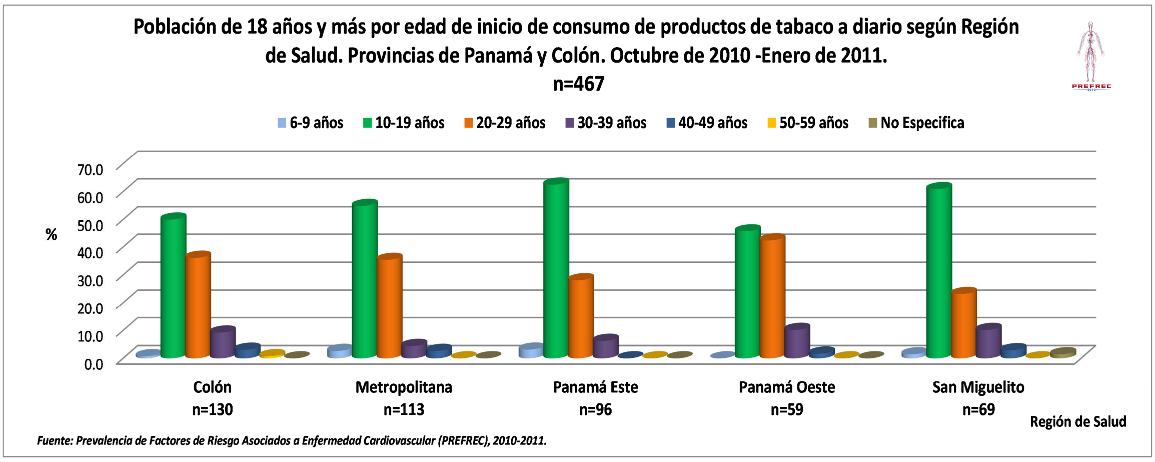

Consumo de productos de tabaco en los últimos 30 días

La prevalencia actual de productos de tabaco como cigarrillos, otros productos fumados de tabaco (cigarros, pipas, tiparillos) y tabaco no fumado, indica el consumo de éstos en los últimos treinta (30) días o el último mes con respecto a la fecha de aplicación de la encuesta, por parte de los 3590 entrevistados. En ese sentido, en esta investigación se estimó una prevalencia actual para el consumo de productos de tabaco de 6.4% (228 personas), presentándose el mayor consumo en el área indígena, seguida del área

urbana y por último el área rural, para una relación entre el área indígena-urbana de 1.0:1, indígena-rural de 1.4:1 y urbano-rural de 1.3:1 (gráfica No. 4).

Gráfica No. 4

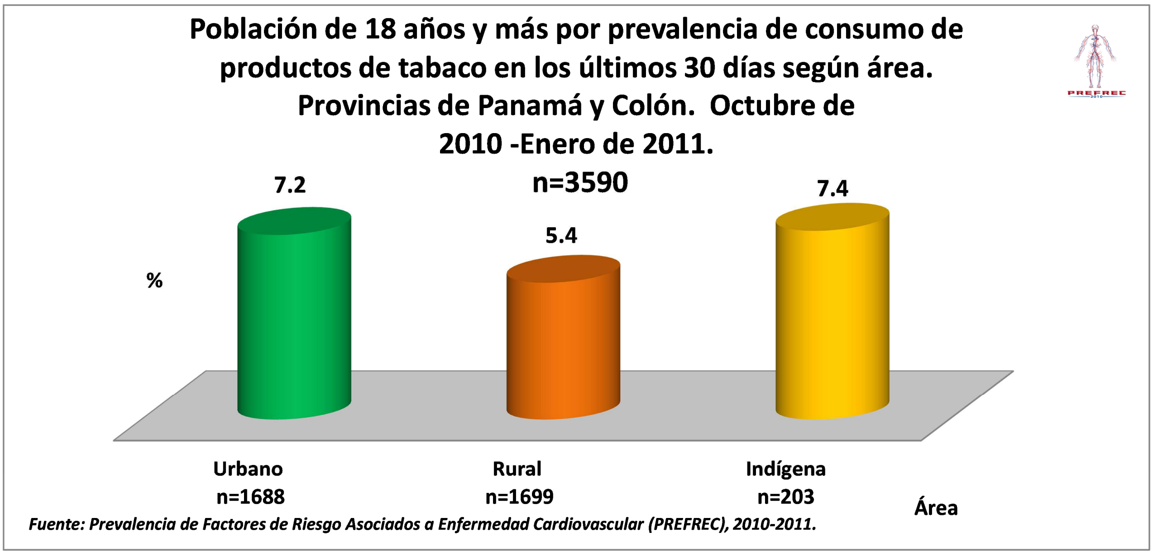

El análisis por género refleja que más hombres que mujeres consumieron productos de tabaco en los últimos 30 días, siendo altamente significativos los estadísticos utilizados (x^2=151.26; p=0.0000), gráfica No. 5.

Gráfica No. 5

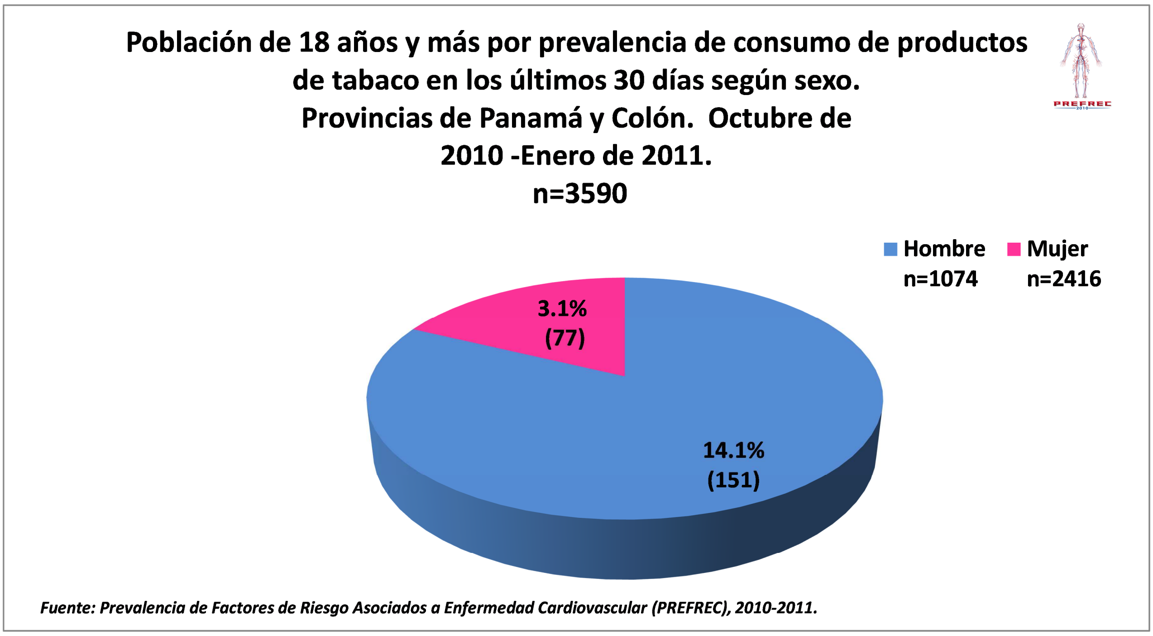

En el caso de la clasificación de adultez, de los 228 individuos que manifestaron el consumo de productos de tabaco en los últimos 30 días, el 18.0% (41) eran adultos

jóvenes, 1 de cada 2 (50.4%) adultos y cerca de 1 de cada 3 (31.6%) adultos mayores, que a su vez representaron el 5.6%, 0.7% y 3.7% del total de adultos jóvenes (735), adultos (2064) y adultos mayores (791) que constituyeron la muestra.

Las prevalencias actuales de consumo de productos de tabaco encontradas en las RS donde se realizó este estudio fueron de:

- 9.2% (48) en la RS de San Miguelito (n=524).
- 6.2% (64) para la RS de Colón (n=1031).
- 6.2% (50) fue el resultado de este indicador en la RS Metropolitana.
- 5.4% tanto para la RS de Panamá Oeste (n=555) y la RS de Panamá Este (n=667).

De acuerdo al tipo de consumo de productos de tabaco, se estimaron las siguientes prevalencias actuales:

- Consumo de cigarrillos: 5.2%.
- Otros productos fumados de tabaco (cigarros, pipas, tiparillos): 0.7%.
- Tabaco no fumado: 0.4%.

Consumo de cigarrillos en el último mes

De los 1047 individuos que manifestaron haber fumado productos de tabaco alguna vez en su vida, 188 entrevistados (18.0%) indicaron el consumo de cigarrillos en los últimos 30 días, 8 de cada 10, es decir, 80.6% (844) manifestaron no haberlos consumido, en tanto que 15 personas (1.4%) no especificaron su respuesta.

Del total de hombres (623) y mujeres (424) que indicaron fumar alguna vez en su vida, 1 de cada 5 (20.1% ó 125 individuos) eran hombres y el 14.9% (63) eran mujeres que habían fumado en los últimos 30 días cigarrillos, presentando diferencias estadísticamente significativas entre género (x^2=4.32; p=0.0377). Tanto el 1.4% de los hombres y mujeres (9 y 6 encuestados, respectivamente) no especificaron su respuesta.

El consumo de cigarrillos en el último mes fue de 20.1% entre los 507 entrevistados del área urbana que respondieron haber fumado alguna vez en su vida. En el área rural fue de 15.8% entre las 469 personas con historia de tabaquismo alguna vez en su vida, en tanto que en el área indígena, el 16.9% de los 71 entrevistados fumadores, consumió productos de tabaco en el último mes, sin diferencias estadísticamente significativas entre las áreas de dominio en este estudio.

El análisis según clasificación de la adultez refleja que de las 188 personas que manifestaron haber fumado en los últimos 30 días, el 18.6%(35) eran adultos jóvenes, 1 de cada 2 (52.1%) eran adultos y cerca de 3 de cada 10 (29.3% ó 55 entrevistados) eran adultos mayores, sin diferencias estadísticamente significativas entre las clasificaciones de adultez y el consumo de cigarrillos en el último mes.

En cuanto a la escolaridad, 3 de cada 10 (31.9%), 4 de cada 10 (43.1%) y 1 de cada 10 (14.9%) de los individuos que habían fumado cigarrillos en los últimos 30 días manifestaron tener como máximo nivel académico la escuela primaria, secundaria y universitaria, respectivamente. Un 3.2% indicó mantener un nivel técnico o haber asistido hasta el primer año de la universidad, mientras que el 5.9% nunca asistió a la escuela. Dos personas no especificaron su respuesta (1.1%).

Las prevalencias actuales estimadas para el consumo de cigarrillos en las RS fueron de 5.1% en Colón, 4.9% en la Metropolitana, 4.0% en Panamá Este, 5.0% en Panamá Oeste y 7.6% en San Miguelito.

Consumo de cigarrillos por más de 10 años

De los 188 entrevistados que manifestaron haber fumado cigarrillos en los últimos 30 días, 2 de cada 10 (21.8%, es decir, 41 individuos) tenían menos de 10 años de estar fumando cigarrillos, mientras que cerca de 8 de cada 10 (77.7%) indicaron tener más de 10 años de consumo. Una persona 0.1% no especificó su respuesta.

De acuerdo al total de hombres y mujeres encuestados que fumaron cigarrillos en los últimos 30 días, se encontró una relación mujer-hombre de 1.1:1 para el tiempo de consumo de 10 años y menos, en tanto que la relación hombre-mujer fue de 1.0:1 para el tiempo de consumo de más de 10 años, sin diferencias estadísticamente significativas entre géneros, (tabla No. 1).

Tabla No.1

Población de 18 años y más por consumo de cigarrillos en los últimos 30 días según tiempo de consumo y sexo. Provincias de Panamá y Colón. Octubre de 2010 – Enero de 2011.
n=188

Tiempo de consumo de cigarrillos	Consumo en los últimos 30 días de cigarrillos					
	TOTAL	%	SEXO			
			Hombre		Mujer	
			Frecuencia	%	Frecuencia	%
**TOTAL**	188	100.0	125	66.5	63	33.5
**10 años o menos**	39	20.7	25	20.0	14	22.2
**Más de 10 años**	148	78.7	99	79.2	49	77.8
**No especificado**	1	0.5	1	0.8	0	0.0

Fuente: Prevalencia de Factores de Riesgo Asociados a Enfermedad Cardiovascular (PREFREC), 2010-2011

Dos de cada 10 individuos tanto en el área urbana como rural, tenía 10 años o menos de estar fumando cigarrillos, mientras que en las mismas áreas, encontramos porcentajes entre 70 y 80 para el tiempo de consumo de más de 10 años (75.5% y 79.7%, respectivamente). En el área indígena, los 12 individuos que reportaron el consumo de cigarrillos en el último mes, tenían más de 10 años de consumo (tabla No.2).

Tabla No.2

Población de 18 años y más por consumo de cigarrillos en los últimos 30 días según tiempo de consumo y área. Provincias de Panamá y Colón.
Octubre de 2010 – Enero de 2011.
n=188

Tiempo de consumo de cigarrillos	Consumo de cigarrillos en los últimos 30 días							
	TOTAL	%	Área					
			Urbana		Rural		Indígena	
			Frecuencia	%	Frecuencia	%	Frecuencia	%
**TOTAL**	**188**	**100.0**	**102**	**54.3**	**74**	**39.4**	**12**	**6.4**
10 años o menos	39	20.7	24	23.5	15	20.3	0	0.0
Más de 10 años	148	78.7	77	75.5	59	79.7	12	100.0
No especificado	1	0.5	1	1.0	0	0.0	0	0.0

Fuente: Prevalencia de Factores de Riesgo Asociados a Enfermedad Cardiovascular (PREFREC), 2010-2011

En el caso del consumo de cigarrillos en el último mes y su relación con el tiempo de consumo de estos productos según RS, encontramos que en la RS de Panamá Este se ubicó la mayor cantidad de fumadores con más de 10 años de consumo, seguido de la RS Metropolitana, San Miguelito, Colón y la RS de Panamá Oeste (gráfica No.6).

Gráfica No.6

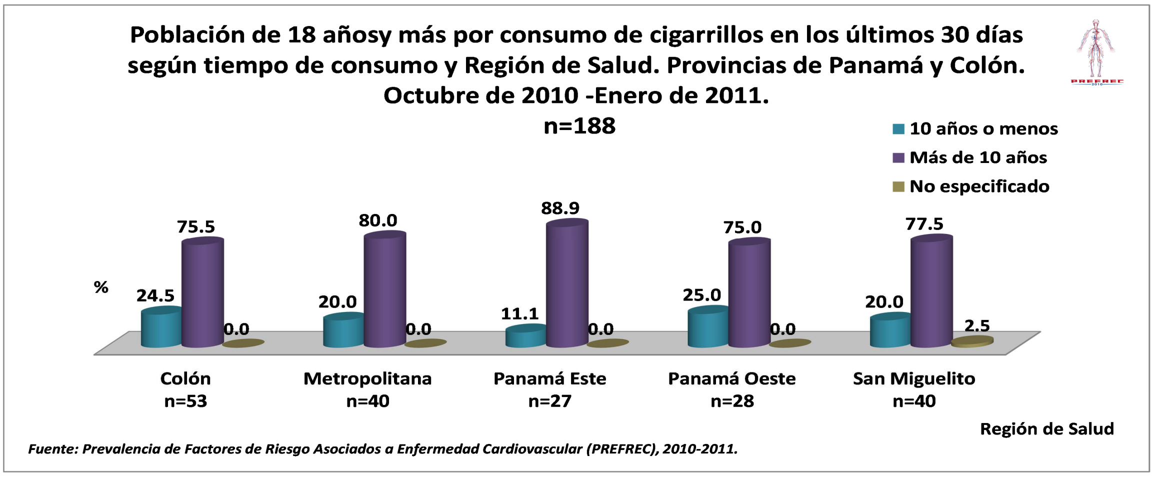

Consumo diario de cigarrillos

Un análisis de las variables seleccionadas en esta investigación, permite estimar entre los 188 individuos que fumaron cigarrillos en los últimos 30 días, cuántos lo hacían a diario, encontrándose que 6 de cada 10 fumadores de cigarrillos (65.4%) lo hacían a diario, mientras que el 34.6% (65 personas) no fumaban a diario.

Del total de hombres y mujeres encuestados que indicaron haber fumado en el último mes (125 y 63, respectivamente), cerca de 7 de cada 10 (69.6%, es decir, 87 hombres) fumaban a diario, en tanto que cerca de 6 de cada 10 mujeres (57.1% ó 36) también tenían esta práctica, sin diferencias estadísticamente significativas entre género.

Como indica la tabla No.3, el consumo diario de cigarrillos durante el último mes respecto a la fecha de aplicación de la encuesta, fue mayor en el área urbana, seguida por el área rural e indígena, estimándose relaciones urbano-rural, urbano-indígena y rural-indígena de 1.1:1, 1.2:1 y 1.1:1, respectivamente.

Tabla No. 3
Población de 18 años y más por consumo de cigarrillos en los últimos 30 días según consumo diario y área. Provincias de Panamá y Colón.
Octubre de 2010 – Enero de 2011.
n=188

Consumo de cigarrillos a diario	Consumo en los últimos 30 días de Cigarrillos							
	TOTAL	%	Área					
			Área Urbana n=102		Área Rural n=74		Área Indígena n=12	
			Frecuencia	%	Frecuencia	%	Frecuencia	%
**TOTAL**	188	100	102	54.3	74	39.4	12	6.4
**SI Fuma o Fumaba a diario**	123	65.4	69	67.6	47	63.5	7	58.3
**No fuma o fumaba a diario**	65	34.6	33	32.4	27	36.5	5	41.7

Fuente: Prevalencia de Factores de Riesgo Asociados a Enfermedad Cardiovascular (PREFREC), 2010-2011.

De acuerdo a la clasificación de adultez, de los 123 individuos que fumaron a diario durante el último mes, 1 de cada 10 (11.4%) era adulto joven, el 56.9% (70) adultos y cerca de 1 de cada 3 (31.7% o 39 individuos) eran adultos mayores. Hubo diferencias estadísticamente significativas entre la condición de adulto – adulto joven y haber fumado a diario (x^2=9.64; p=0.0019) y para la condición adulto mayor – adulto joven (x^2=7.21; p=0.0072).

El análisis entre las personas que fumaron cigarrillos a diario en el último mes (123) y el máximo nivel de escolaridad indica que el mayor porcentaje de fumadores a diario alcanzaron el nivel secundario (36.6%), 1 de cada 3 (33.3% o 41 individuos) el nivel primario, mientras que un 15.4% (19) el nivel universitario. El 8.1% (10 personas) manifestó nunca haber asistido a la escuela, en tanto que el 4.9% (6) había culminado estudios técnicos o solo realizó el primer año de universidad. Dos personas (1.6%) no especificaron su respuesta.

A nivel de las RS, los resultados de esta encuesta, evidencian que para la muestra en estudio, la RS de Colón presentó el mayor porcentaje de fumadores de cigarrillo a diario, seguido de la RS de Panamá Este, Metropolitana, San Miguelito y Panamá Oeste (gráfica No.7).

Consumo de otros productos fumados de tabaco en el último mes

De los entrevistados que indicaron fumar productos de tabaco alguna vez en su vida (1047) solo el 2.5% (26) reportó el consumo en los últimos 30 días de otros productos fumados de tabaco, tales como cigarros, pipas y tiparillos. Nueve de cada 10 (95.0%) negó este consumo, en tanto que el 2.5% (26) no especificó su respuesta.

Gráfica No. 7

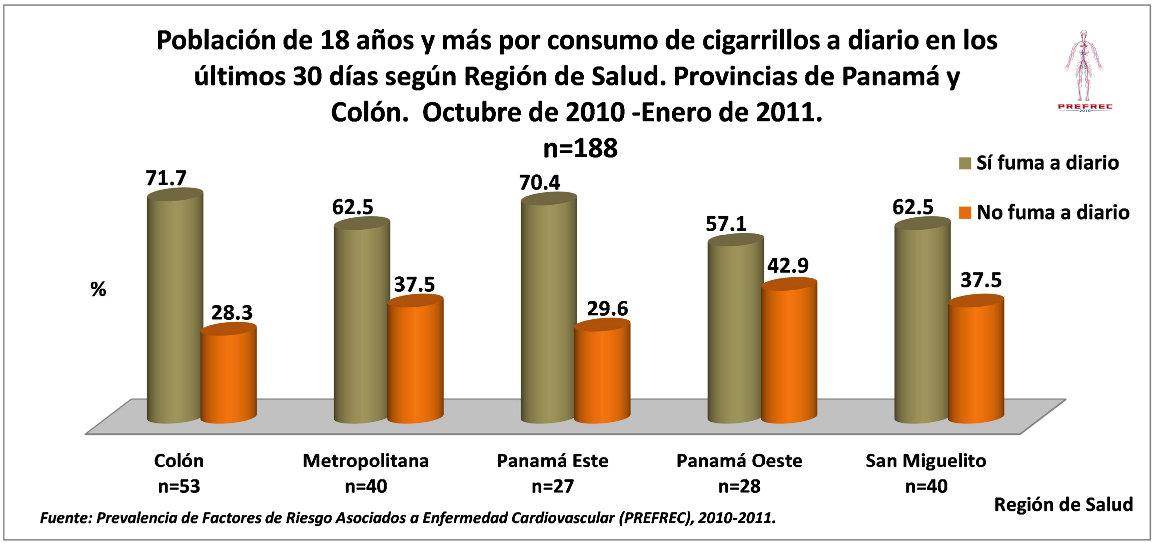

De estas 26 personas, el análisis de acuerdo al total de hombres (623) y mujeres (424) que indicaron fumar alguna vez en su vida, refleja que el 2.6% (16) de estos consumidores eran hombres y el 2.4% (10) mujeres, sin diferencias estadísticamente significativas entre géneros. De los que no especificaron su respuesta (26), el 1.9% era hombre y un 3.3% mujeres.

Con relación al total de los fumadores en cada una de las áreas, el 2.0% (10) de los habitantes del área urbana, 3.2% (15) de los residentes del área rural y el 1.4% (1) de los entrevistados en el área indígena habían consumido otros productos de tabaco fumado en el último mes.

La mayor cantidad de consumidores de cigarros, pipas y tiparillos eran adultos mayores (1 de cada 2, es decir el 50.0%). Tres de cada 10 eran adultos (30.8%) y cerca de 1 de cada 5 eran adultos jóvenes (19.2%).

En cuanto a la escolaridad, cerca de 3 de cada 10 (26.9%) y 2 de cada 10 (23.1%) de los individuos que habían fumado otros productos de tabaco en los últimos 30 días tenían como máximo nivel académico la escuela primaria y secundaria. Un 7.7% (2) y el 3.8% (1) habían alcanzado el grado universitario y el nivel de post grado, respectivamente. Tres de cada 10 (34.6%) no asistieron a la escuela, en tanto que el 3.8% no especificó su respuesta (1).

Para las RS, las prevalencias actuales de otros productos fumados de tabaco fueron de 0.9% en Colón, 0.6% en la Metropolitana, 0.8% en Panamá Este, 0.4% en Panamá Oeste y 1.0% en San Miguelito.

Consumo de tabaco no fumado en el último mes
Solo el 1.3% (14 individuos) de los 1047 individuos que reportaron haber fumado productos de tabaco alguna vez en su vida, también consumieron tabaco no fumado. El 95.7% indicó que no ha consumido tabaco no fumado, en tanto que 31 entrevistados no especificaron su respuesta.

De los 14 encuestados que manifestaron el consumo de tabaco no fumado, el 1.6% era hombre y el 0.9% mujer, respecto al total de hombres (623) y mujeres (424) que indicaron fumar alguna vez en su vida. El 2.4% de los hombres y el 3.8% de las mujeres (15 y 16 encuestados, respectivamente) no especificaron su respuesta.

En cuando a las áreas de dominio de este estudio, el 2.0% (10); 0.4% (2) y un 2.8% (2) de los entrevistados fumadores, habían consumido en los últimos 30 días tabaco no fumado en las áreas urbana, rural e indígena, respectivamente.

De acuerdo a la clasificación de la adultez utilizada en esta investigación, el mayor consumo de tabaco no fumado se dio en adultos (64.3%), seguido de los adultos mayores (28.6%) y los adultos jóvenes (7.1%).

El máximo nivel académico de las personas que consumen tabaco no fumado reflejó ser el secundario en un 35.7%, universitario en un 28.6% y técnico o primer año de la universidad fue indicado por el 7.1%. El 21.4% de estos consumidores nunca asistió a la escuela, mientras que el 7.1% no especificó su respuesta.

Las prevalencias actuales de tabaco no fumado según RS fueron de 0.2% en Colón, 0.6% en la Metropolitana, 0.6% en Panamá Este y 0.6% en San Miguelito. No hubo consumo entre los entrevistados de la RS de Panamá Oeste.

Cantidad de cigarrillos que fuma o fumaba y el número de días al mes
Fumadores livianos: comportamiento general
En esta encuesta, una pregunta se formuló para conocer la cantidad de cigarrillos y el número de días al mes que el individuo los consumía. En ese sentido, encontramos que de las 1047 personas que reportaron el consumo alguna vez en su vida de productos de tabaco, 6 de cada 10 (63.8% ó 668) manifestaron ser fumadores livianos (fuman 5 cigarrillos o menos al día), de los cuales:

- El 72.2% (482) manifestó hacerlo de 1 a 7 días al mes.
- Un 6.1% (41) fuma entre 8 a 15 días al mes.
- El 1.3% (9) indicó fumar de 16 a 21 días al mes.
- Uno de cada 4 (20.4%) respondió fumar entre 22 a 31 días al mes.

En el caso de las personas que indicaron el consumo de cigarrillos en los últimos 30 días con referencia al momento de aplicación de la encuesta (188), 6 de cada 10 (60.1%, es decir, 113 individuos) eran fumadores livianos. El análisis diferenciado según cantidad de días de consumo se presenta la tabla No.4.

Fumadores livianos: Análisis según sexo

El análisis según género refleja que del total de personas que han consumido productos de tabaco alguna vez en su vida (623 hombres y 424 mujeres) el 58.3% de los hombres (363) y el 71.9% de las mujeres (305), son fumadores livianos, presentando diferencias estadísticamente significativas entre géneros (x^2=19.82; p=0.0000085).

- En el caso de los hombres, cerca de 7 de cada 10 (69.1%) fuma 5 cigarrillos o menos entre 1 a 7 días al mes, un 7.7% lo hace entre 8 a 15 días al mes, el 1.9% fuma entre 16 a 21 días al mes, mientras que 1 de cada 5 (21.2%) fuma cigarrillos entre 22 a 31 días al mes.
- De las mujeres que son fumadores livianos, 3 de cada 4 (75.5%) fuma entre 1 a 7 días al mes, un 4.3% y el 0.7% lo hacen entre 8 a 15 días y de 16 a 21 días al mes, respectivamente, en tanto que cerca de 1 de cada 5 (19.3%), fuma cigarrillos entre 22 a 31 días al mes.

El mismo análisis sobre el total de hombres y mujeres (125 y 63, respectivamente) que reportaron haber fumado en los últimos 30 días indica que el 56.8% de los hombres (71) y el 66.7% de las mujeres (42), son fumadores livianos, sin diferencias estadísticamente significativas entre géneros. La cantidad de días de consumo según género se presenta en la tabla No.4.

Tabla No.4

Población de 18 años y más por consumo de 5 cigarrillos o menos en los últimos 30 días según tiempo de consumo y sexo. Provincias de Panamá y Colón.
Octubre de 2010 – Enero de 2011.
n=113

Cantidad de días en los que fuma	Consumo de 5 cigarrillos o menos en los últimos 30 días					
	TOTAL		SEXO			
			Hombre		Mujer	
	Frecuencia	%	Frecuencia	%	Frecuencia	%
**TOTAL**	**113**	**100.0**	71	**62.8**	42	**37.2**
1 a 7 días	64	56.6	37	52.1	27	64.3
8 a 15 días	15	13.3	13	18.3	2	4.8
16 a 21 días	0	0.0	0	0.0	0	0.0
22 a 31 días	34	30.1	21	29.6	13	31.0

Fuente: Prevalencia de Factores de Riesgo Asociados a Enfermedad Cardiovascular (PREFREC), 2010-2011.

Fumadores livianos: Análisis según clasificación de adultez

Con base a la clasificación de adultez, de las personas que indicaron el consumo de productos de tabaco alguna vez en la vida, 668 individuos eran fumadores livianos, de los cuales:

- 482 fumaban entre 1 a 7 días al mes, de los que 19.1% eran adultos jóvenes, 1 de cada 2 (53.1%) eran adultos y 27.8% adultos mayores.
- 41 personas fumaban entre 8 a 15 días al mes, donde cerca de 1 de cada 5 (19.5%) era adulto joven, cerca de 6 de cada 10 (58.5%) eran adultos y el 22.0% adulto mayor.

- 9 entrevistados consumían cigarrillos entre 16 a 21 días al mes, siendo el 11.1% adulto joven, 2 de cada 3 (66.7%) adulto y el 22.2% adulto mayor.
- 136 encuestados fumaban cigarrillos entre 22 a 31 días al mes. De éstos, cerca de 1 de cada 5 (19.1%) era adulto joven, un 46.3% adulto, en tanto que 1 de cada 3 (34.6%) era adulto mayor.

De los entrevistados que reportaron el consumo de cigarrillos en el último mes y que eran fumadores livianos (113), cerca de 1 de cada 4 (24.8%) era adulto joven, cerca de 5 de cada 10 (48.7%, es decir, 55 personas) eran adultos y un 26.5% (30) eran adultos mayores. La cantidad de días de consumo indicadas por las personas que fumaban menos de 5 cigarrillos al mes, se presenta en la gráfica No. 8.

Gráfica No. 8

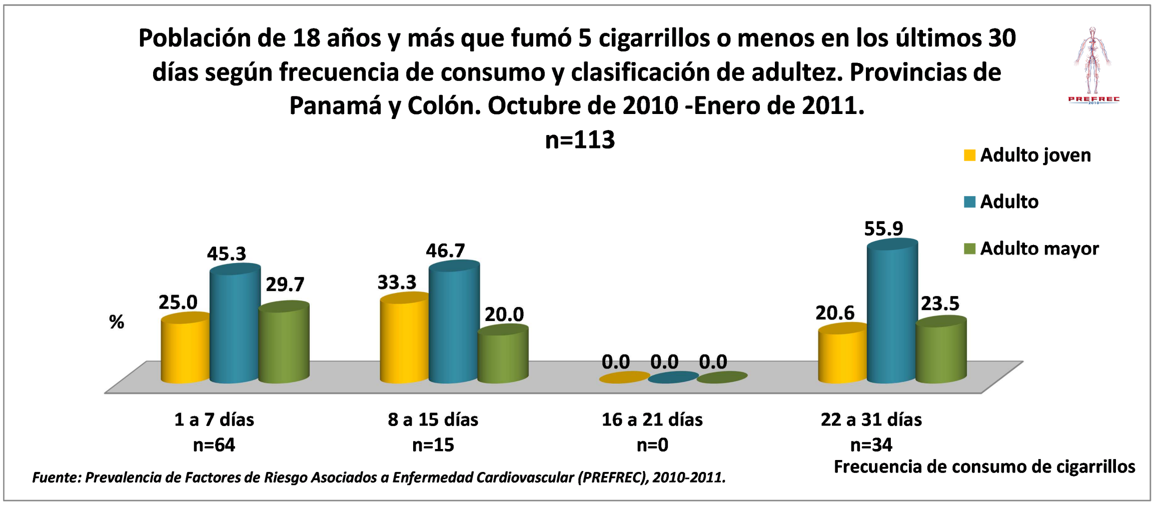

Fumadores livianos: Análisis según nivel de escolaridad

En cuanto al máximo nivel académico alcanzado por los individuos con historia de tabaquismo alguna vez en la vida y que eran fumadores livianos, 1 de cada 3 (34.6%) había culminado la escuela primaria, un 36.2% tenía estudios secundarios, el nivel técnico (incluido el primer año de la universidad) fue indicado como la escolaridad del 3.6% de los individuos que consumen 5 cigarrillos o menos al mes. El nivel universitario fue manifestado por el 15.1%, un 2.2% tenía estudios de postgrado, mientras que el 1.0% no especificó su respuesta. Un 7.2% reportó que nunca fue a la escuela.

El comportamiento de la variable máximo nivel educativo entre los fumadores de 5 cigarrillos o menos al mes y que manifestaron fumar en el último mes con relación al momento de la aplicación de la encuesta reflejó que de los 113 fumadores livianos el 42.5% (48) tenía el nivel secundario como máxima escolaridad, estimándose relaciones secundario-primaria de 1.3:1; secundario-técnico (I año de universidad) de 12.0:1; secundario-universidad de 3.0:1 y secundario-nunca asistió a la escuela de 8.0:1 (gráfica No.9).

Gráfica No. 9

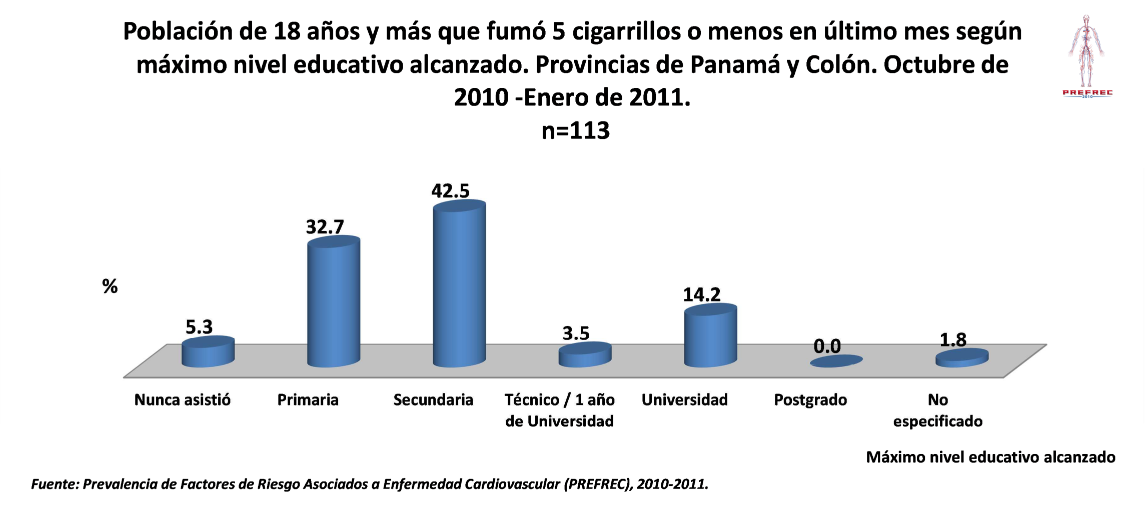

Fumadores medianos: comportamiento general

El 11.6% de las personas con historia de tabaquismo alguna vez en su vida (121) indicó fumar de 6 a 15 cigarrillos (fumadores medianos), revelando que:

- 3 de cada 10 (31.4%) fuma de 6 a 15 cigarrillos durante 1 a 7 días al mes.
- El 8.3% (10) fuma la misma cantidad de cigarrillos pero entre 8 a 15 días al mes.
- Entre 16 a 21 días al mes fuma el 3.3% (4) de los consumidores de 6 a 15 cigarrillos.
- Cerca de 6 de cada 10 (57.0%) indicaron fumar entre 22 a 31 días al mes.

De los consumidores de cigarrillo en el último mes (188), el 18.6% (35 personas) eran fumadores medianos, de los cuales, cerca de 6 de cada 10 (57.1% o 20 entrevistados) fumaban entre 22 a 31 días al mes, tal como se presenta en la tabla No.5.

Fumadores medianos: Análisis según sexo

El comportamiento según sexo indica que del total de los entrevistados con historia de tabaquismo alguna vez en su vida (623 hombres y 424 mujeres) el 13.5% de los hombres (84) y el 8.7% de las mujeres (37), son fumadores medianos, con diferencias estadísticamente significativas entre géneros (x^2=5.13; p=0.0235).

- Tres de cada 10 hombres (31.0%) fuma entre 6 a 15 cigarrillos de 1 a 7 días al mes, un 9.5% entre 8 a 15 días al mes, el 4.8% fuma entre 16 a 21 días al mes, mientras que 1 de cada 2 (54.8%) fuma cigarrillos entre 22 a 31 días al mes.
- De las mujeres que son fumadores medianas, cerca de 1 de cada 3 (32.4%) fuma de 6 a 15 cigarrillos entre 1 a 7 días al mes, un 5.4% lo hace entre 16 a 21 días al mes, en tanto que cerca de 6 de cada 10 (62.2%), fuma cigarrillos entre 22 a 31 días al mes.

Bajo este mismo enfoque se realizó el análisis sobre el total de hombres y mujeres (125 y 63, respectivamente) que reportaron haber fumado en los últimos 30 días, encontrándose

que el 18.4% de los hombres (23) y el 19.0% de las mujeres (12), son fumadores medianos, sin diferencias estadísticamente significativas entre géneros. La cantidad de días de consumo según género se presenta en la tabla No.5.

Tabla No.5
Población de 18 años y más por consumo de 6 a 15 cigarrillos en los últimos 30 días según tiempo de consumo y sexo. Provincias de Panamá y Colón. Octubre de 2010 – Enero de 2011.
n=35

Cantidad de días en los que fuma	Consumo de 6 - 15 cigarrillos o menos en los últimos 30 días					
	TOTAL		SEXO			
			Hombre		Mujer	
	Frecuencia	%	Frecuencia	%	Frecuencia	%
**TOTAL**	**35**	**100.0**	23	**65.7**	12	**34.3**
1 a 7 días	10	28.6	5	21.7	5	41.7
8 a 15 días	5	14.3	4	17.4	1	8.3
16 a 21 días	0	0.0	0	0.0	0	0.0
22 a 31 días	20	57.1	14	60.9	6	50.0

Fuente: Prevalencia de Factores de Riesgo Asociados a Enfermedad Cardiovascular (PREFREC), 2010-2011.

Fumadores medianos: Análisis según clasificación de adultez

Según a la clasificación de adultez, de los individuos con historia de tabaquismo alguna vez en su vida, 121 eran fumadores de 6 a 15 cigarrillos al mes, de los cuales:

- 38 personas consumían cigarrillos entre 1 a 7 días al mes, de los que 1 de cada 10 (10.5%) era adulto joven, cerca de 5 de cada 10 (47.4%) eran adultos y 4 de cada 10 (42.1%) eran adultos mayores.
- 10 entrevistados fumaban entre 8 a 15 días al mes, donde cerca de 1 de cada 10 (10.0%) era adulto joven, 7 de cada 10 (70.0%) eran adultos y 2 de cada 10 (20.0%) eran adultos mayores.
- 4 individuos fumaban cigarrillos entre 16 a 21 días al mes, siendo el 25.0% adultos y el 75.0% adultos mayores.
- 69 encuestados fumaban entre 22 a 31 días al mes. De éstos, el 8.7% era adulto joven, 1 de cada 2 (52.2%) era adulto y cerca de 4 de cada 10 (39.1%) eran adultos mayores.

De los fumadores medianos (35) que señalaron el consumo de cigarrillos en el último mes, el 14.3% (5) era adulto jóven, cerca de 5 de cada 10 (48.6%, es decir, 17 entrevistados) eran adultos y un 37.1% (13) eran adultos mayores, siendo los que más fuman durante el mes (22 a 31 días) los adultos, seguido de los adultos mayores y por último los adultos jóvenes (gráfica No.10).

Fumadores medianos: Análisis según nivel de escolaridad

De las personas que indicaron el consumo de cigarrillos alguna vez en su vida y que fueron clasificados como fumadores medianos, 4 de cada 10 (43.8%) había culminado la escuela primaria, 2 de cada 7 (28.9%) tenía estudios secundarios, el nivel técnico (incluido el primer año de la universidad) fue indicado solo por el 0.8% de los fumadores medianos, en tanto que el nivel universitario fue manifestado por el 12.4%. Un 2.5% no especificó su respuesta, mientras que un 11.6% nunca asistió a la escuela.

Gráfica No. 10

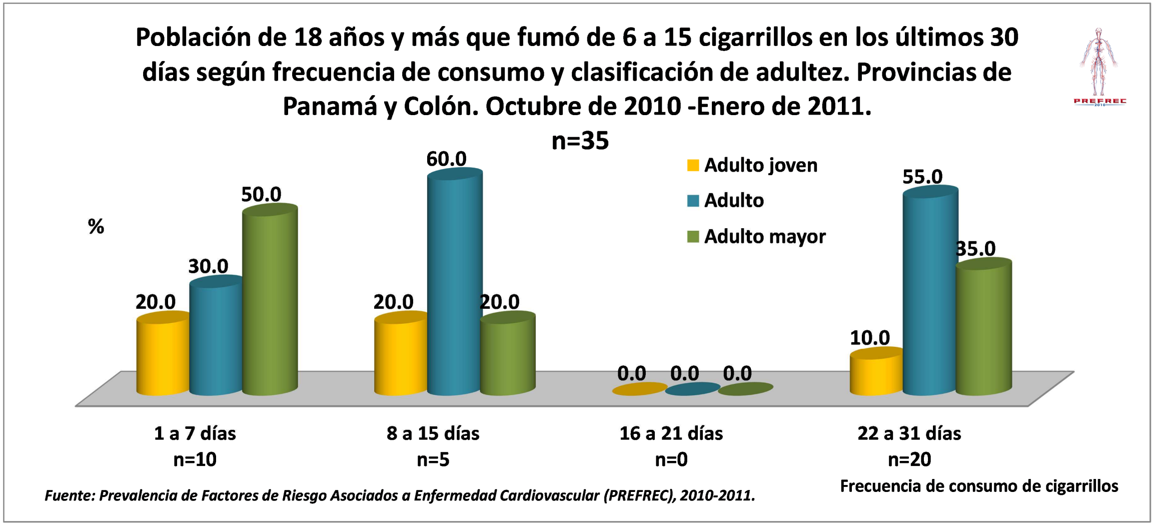

Fuente: Prevalencia de Factores de Riesgo Asociados a Enfermedad Cardiovascular (PREFREC), 2010-2011.

En el caso de los fumadores medianos que manifestaron el consumo de cigarrillos en los últimos 30 días (35), 4 de cada 10 (42.9%) tenía como máximo nivel académico alcanzado el secundario, seguido por el nivel primario con un 31.4% (11 personas). Un 14.3% (5) nunca asistió a la escuela y el 11.4% (4 individuos) tenía un nivel universitario.

Fumadores pesados: comportamiento general

De los encuestados que señalaron el consumo de productos de tabaco alguna vez en la vida, cerca de 1 de cada 5 entrevistados (19.2% es decir, 201 individuos) respondió fumar 16 cigarrillos y más (fumador pesado), con la siguiente frecuencia:

- El 23.9% (48) indicó hacerlo de 1 a 7 días al mes.
- Un 1.0% (2) fuma entre 8 a 15 días al mes.
- El 1.5% (3) manifestó fumar de 16 a 21 días al mes.
- Siete de cada 10 (73.6%) respondieron fumar entre 22 a 31 días al mes.

Los consumidores de 16 y más cigarrillos que fumaron en los últimos 30 días (40) al momento de aplicar la encuesta, representaron el 21.3% de los 188 individuos que fumaron en el último mes, donde cerca de 8 de cada 10 (77.5%, es decir 31 individuos) fumaban entre 22 a 31 días al mes (tabla No.6).

Fumadores pesados: Análisis según sexo

El análisis según sexo muestra que del total de personas que han consumido productos de tabaco alguna vez en su vida (623 hombres y 424 mujeres), el 23.1% de los hombres (144) y el 13.4% de las mujeres (57), son fumadores pesados, presentando diferencias estadísticamente significativas entre géneros (x^2=14.59; p=0.0001334).

- Uno de cada 4 hombres (25.0%) fuma 16 cigarrillos y más entre 1 a 7 días al mes, el 1.4% lo hace entre 8 a 15 días al mes, un 2.1% fuma entre 16 a 21 días al mes, en tanto que 7 de cada 10 (71.5%) fuma cigarrillos entre 22 a 31 días al mes.

- De las mujeres, 1 de cada 5 (21.1%) fuma 16 y más cigarrillos entre 1 a 7 días al mes, mientras que cerca de 8 de cada 10 (78.9%), fuma cigarrillos entre 22 a 31 días al mes.

De acuerdo al total de hombres y mujeres (125 y 63, respectivamente) que manifestaron haber fumado en los últimos 30 días, el 24.8% de los hombres (31) y el 14.3% de las mujeres (9), eran fumadores pesados, sin diferencias estadísticamente significativas entre géneros. La cantidad de días de consumo según género se presenta en la tabla No.6.

Tabla No.6

Población de 18 años y más por consumo de 16 cigarrillos o más en los últimos 30 días según tiempo de consumo y sexo. Provincias de Panamá y Colón.
Octubre de 2010 – Enero de 2011.
n=40

Cantidad de días en los que fuma	Consumo de 16 cigarrillos y más en los últimos 30 días					
	TOTAL		SEXO			
			Hombre		Mujer	
	Frecuencia	%	Frecuencia	%	Frecuencia	%
**TOTAL**	**40**	**100.0**	31	**77.5**	9	**22.5**
1 a 7 días	6	15.0	5	16.1	1	11.1
8 a 15 días	0	0.0	0	0.0	0	0.0
16 a 21 días	3	7.5	3	9.7	0	0.0
22 a 31 días	31	77.5	23	74.2	8	88.9

Fuente: Prevalencia de Factores de Riesgo Asociados a Enfermedad Cardiovascular (PREFREC), 2010-2011.

Fumadores pesados: Análisis según clasificación de adultez

Al realizar el análisis entre las personas con historia de tabaquismo alguna vez en su vida que consumen 16 y más cigarrillos según la clasificación de adultez, encontramos que de los 201 encuestados fumadores pesados:

- 48 individuos fumaban cigarrillos entre 1 a 7 días al mes, de los cuales el 4.2% era adulto joven, 4 de cada 10 (43.8%) eran adultos y 1 de cada 2 (52.1%) eran adultos mayores.
- 2 entrevistados fumaban entre 8 a 15 días al mes, los cuales eran adultos mayores.
- 3 individuos fumaban cigarrillos entre 16 a 21 días al mes, siendo en su totalidad adultos mayores.
- 148 encuestados fumaban entre 22 a 31 días al mes. De éstos, el 4.7% era adulto joven, 1 de cada 2 (54.7%) era adulto y 4 de cada 10 (40.5%) eran adultos mayores.

De los encuestados que indicaron haber fumado cigarrillos en el último mes, en cantidades de 16 o más (40), un 5% (2) eran adultos jóvenes, cerca de 2 de cada 3 (65.0%, o sea, 26 individuos) eran adultos y 3 de cada 10 (30%) eran adultos mayores. No hubo fumadores en las categorías de 8 a 15 días y de 16 a 21 días. La cantidad de días de consumo para estos fumadores se presenta en la gráfica No. 11.

Gráfica No. 11

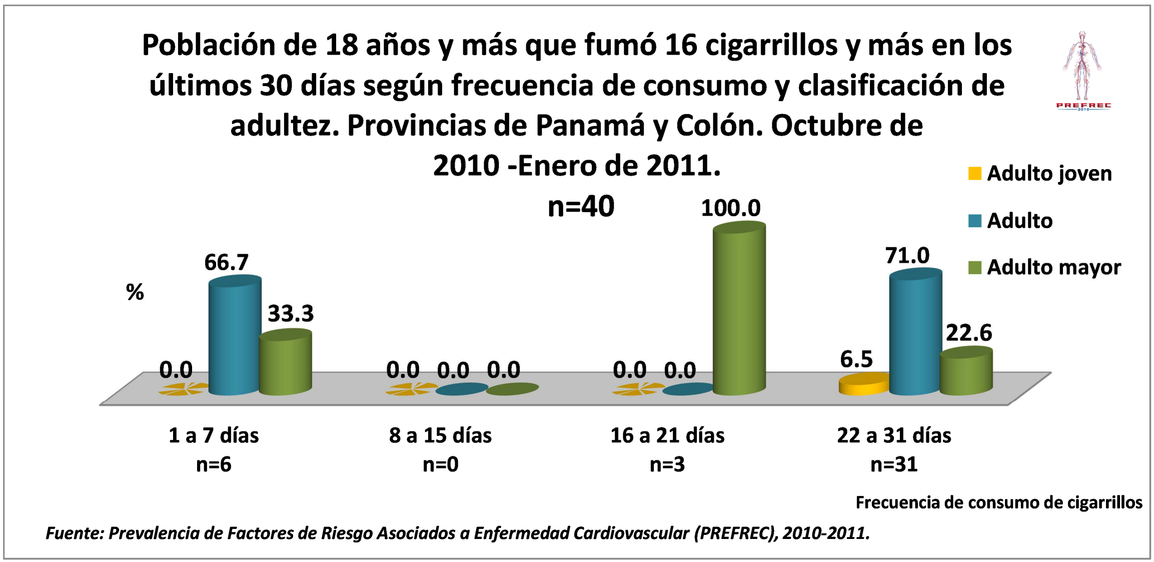

Fumadores pesados: Análisis según nivel de escolaridad

De los individuos que indicaron fumar alguna vez en la vida, 4 de cada 10 y 1 de cada 3 de los que fuman 16 y más cigarrillos al mes, tienen como máximo nivel académico alcanzado los estudios primarios y secundarios, respectivamente. El nivel técnico (incluido el primer año de la universidad) fue indicado como la escolaridad del 3.0% de estos individuos. Un 12.8% manifestó tener estudios universitarios, mientras que el 7.0% nunca fue a la escuela.

Respecto a las 188 personas que manifestaron fumar en el último mes (con referencia a la fecha de aplicación de la encuesta), encontramos que según el nivel académico, las personas con nivel técnico-1 año de universidad y las universitarias, reflejaron el mayor consumo. La gráfica No. 12 indica este comportamiento, así como su comparación con los fumadores medianos y livianos.

Gráfica No. 12

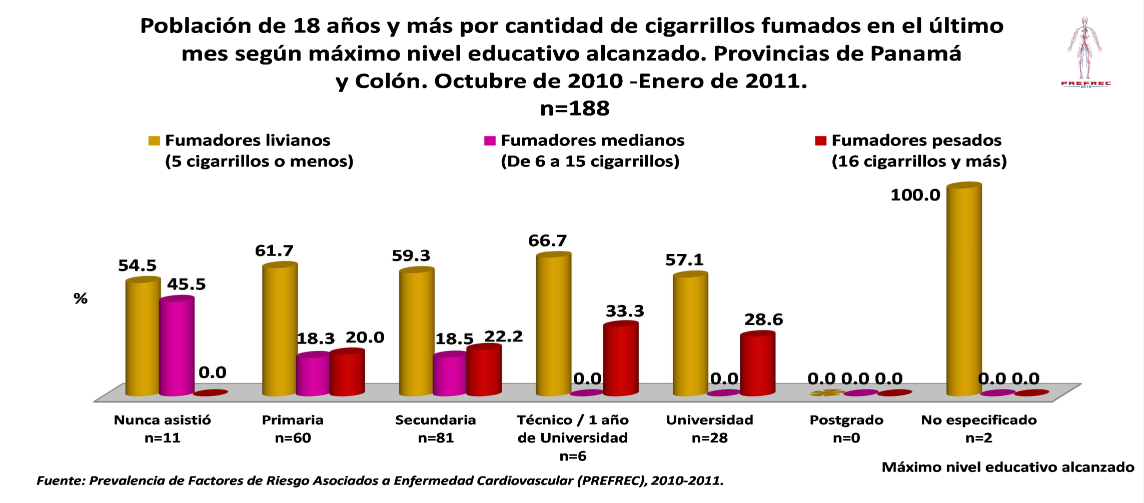

No respuesta

El 5.4% (57) de los entrevistados que manifestaron fumar alguna vez en su vida, no respondieron a esta pregunta. De ellos, el 22.8% era adulto joven, 4 de cada 10 (43.9%) eran adultos y 1 de cada 3 (33.3%) eran adultos mayores.

De estas personas, 1 de cada 10 (10.5%) nunca asistió a la escuela, el 26.3% tenía estudios primarios, un 31.6% estudios secundarios, la universidad fue el nivel de escolaridad manifestado por el 26.3% de los entrevistados, en tanto que un 5.3% tenía estudios de post grado.

Fumadores livianos, medianos y pesados según área

El análisis según las áreas de dominio de esta investigación, refleja que:

- De las personas que alguna vez en su vida habían fumado en el área urbana (507), el 62.1% fumaba 5 cigarrillos o menos al mes, un 10.8% de 6 a 15 cigarrillos, mientras que el 20.3% consumía 16 cigarrillos y más al mes. Un 6.7% (34) de los entrevistados en esta área, no especificaron su respuesta.

    - De las 315 personas que mencionaron fumar 5 cigarrillos o menos, 7 de cada 10 (70.5%) lo hace de 1 a 7 días al mes, el 6.3% entre 8 a 15 días al mes, un 2.2% manifestó fumar de 16 a 21 días al mes, en tanto que 2 de cada 10 (21.0%) indicaron el consumo de cigarrillos entre 22 a 31 días al mes.

    - En el caso de los que fumaban de 6 a 15 cigarrillos al mes (55 entrevistados), cerca de 3 de cada 10 (29.1%) manifestaron hacerlo de 1 a 7 días al mes, 1 de cada 10 (12.7%) fuma entre 8 a 15 días al mes, el 3.6% indicó fumar de 16 a 21 días al mes, mientras que 1 de cada 2 (54.5%) respondió fumar entre 22 a 31 días al mes.

    - De las personas que consumían 16 cigarrillos y más (103), el 18.4% lo hacía entre 1 a 7 días al mes y el 81.6% restante fumaba entre 22 a 31 días al mes.

- En el caso del área rural, señalaron ser fumadores 469 individuos, de los cuales, el 64.8% fumaba 5 cigarrillos o menos al mes, un 12.4% de 6 a 15 cigarrillos, mientras que el 17.9% consumía 16 cigarrillos y más al mes. El 4.9% de los encuestados (23) no especificaron su respuesta.

    - De los entrevistados que fumaban 5 cigarrillos o menos (304), el 73.7% que corresponde a 7 de cada 10, lo hacía de 1 a 7 días al mes, el 4.9% entre 8 a 15 días al mes, un 0.3% manifestó fumar de 16 a 21 días al mes, en tanto que el 21.1% indicó el consumo de cigarrillos de 22 a 31 días al mes.

- De los que consumían de 6 a 15 cigarrillos al mes (58 personas), 1 de cada 3 (34.5%) indicó hacerlo entre 1 a 7 días al mes, el 5.2% y el 3.4% fumaban entre 8 a 15 días al mes y de 16 a 21 días al mes, respectivamente. Cerca de 6 de cada 10 (56.9%) fumaba cigarrillos entre 22 a 31 días al mes.

- De las personas que fumaban 16 cigarrillos y más (84), cerca de 1 de cada 3 (31.0%) lo hacía entre 1 a 7 días al mes, el 2.4% fumaba entre 8 a 15 días al mes, el 3.6% indicó fumar de 16 a 21 días al mes, en tanto que 6 de cada 10 (63.1%) respondió fumar entre 22 a 31 días al mes.

- En el área indígena, las personas con historia de tabaquismo alguna vez en su vida fueron 71. De éstos, el 69.0% fumaba 5 cigarrillos o menos al mes, un 11.3% de 6 a 15 cigarrillos, mientras que el 19.7% consumía 16 cigarrillos y más al mes.

    - De las 49 personas que reportaron fumar 5 cigarrillos o menos, 7 de cada 10 (73.5%) lo hace entre 1 a 7 días al mes, el 12.2% entre 8 a 15 días al mes, un 2.0% manifestó fumar de 16 a 21 días al mes, en tanto que 1 de cada 10 (12.2%) indicó el consumo de cigarrillos entre 22 a 31 días al mes.

    - De los que consumían de 6 a 15 cigarrillos al mes (8 individuos), 1 de cada 4 (25.0%) indicó hacerlo de 1 a 7 días al mes, mientras que 3 de cada 4 (75.0%) fumaba de 6 a 15 cigarrillos entre 22 a 31 días al mes.

    - De las personas que consumían 16 cigarrillos y más (14), el 21.4% lo hacía entre 1 a 7 días al mes y el 78.6% fumaba entre 22 a 31 días al mes.

En el caso de las personas que señalaron el consumo de cigarrillos en el último mes (188), el análisis según área refleja que hubo más fumadores livianos en el área rural (n=74), seguida de la indígena (n=12) y el área urbana (n=102), con un 67.6% (50), 66.7% (8) y 53.9% (55).

En el caso de los fumadores medianos, el mayor consumo estuvo en el área indígena, donde 1 de cada 4 (25.0%) eran fumaban de 6 a 15 cigarrillos al mes. En el área urbana se reportó esta cantidad de consumo en un 19.6% (20) mientras que en el área rural fue de 16.2% (12).

El análisis de los fumadores pesados indica que la mayor cantidad se ubicó en el área urbana con un 26.5% (27), seguido por el área rural donde fue indicado por el 16.2% (12) de los fumadores actuales residentes en dicha área, mientras que un 8.3% (1) de las personas que fuman 16 cigarrillos y más se ubicó en el área indígena. El comportamiento de estas variables según cantidad de días, se presenta en la gráfica No.13.

Gráfica No. 13

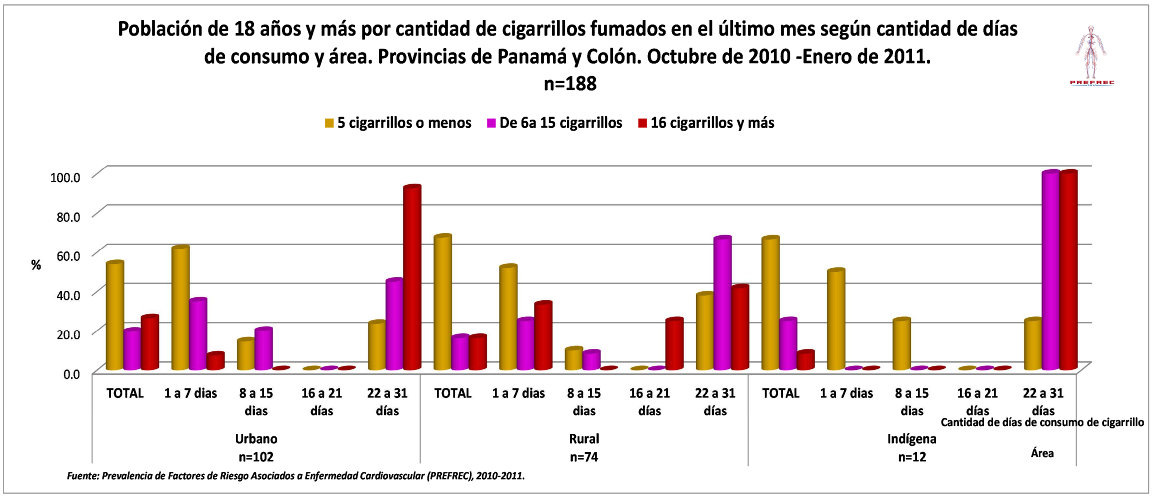

Consumo de cigarrillos según RS

En general, de las personas que manifestaron consumir alguna vez en la vida productos de tabaco, la mayor cantidad de fumadores livianos fueron encontrados en la RS de Panamá Oeste, seguida por la RS Metropolitana y Colón. Para los fumadores medianos, los porcentajes oscilaban entre 9.1% y 15.0%, siendo la RS de San Miguelito la del mayor porcentaje y Colón con el más bajo en la escala. En cuanto a los fumadores pesados, porcentajes superiores a 15 encontramos en las RS de Colón, Panamá Este, San Miguelito y Metropolitana, tal como se muestra en el gráfica No.14.

Gráfica No.14

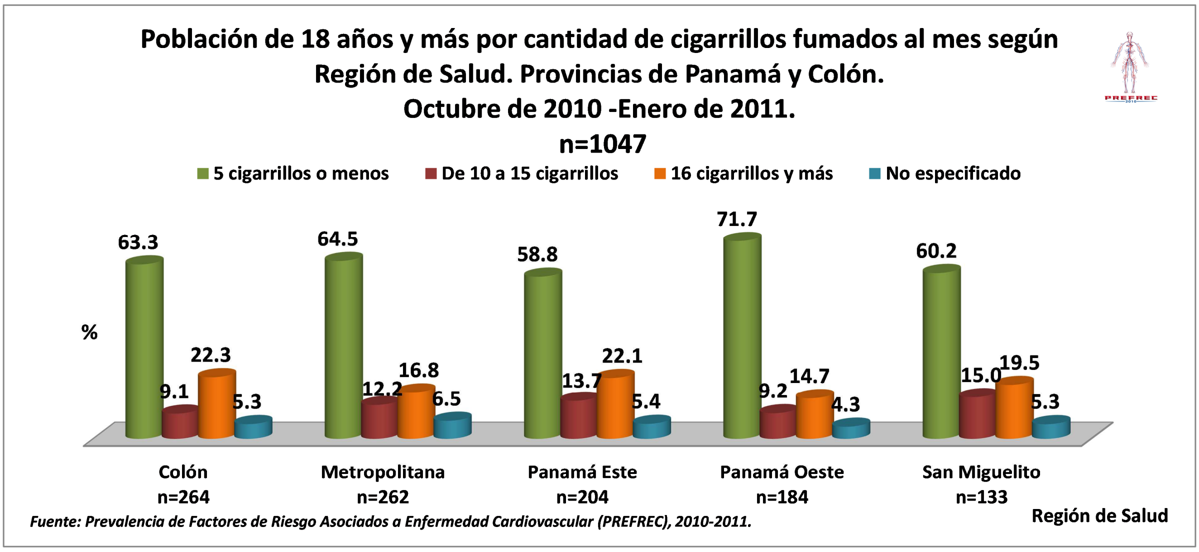

En el caso de los fumadores actuales (fumadores en el último mes, n=188), hubo un 21.3% (40) de fumadores pesados, los cuales pertenecían a las siguientes RS (gráfica No. 15):

- La RS de Panamá Oeste (n=24) con un 28.6% (8).

- En la RS de Colón (n=53) se ubicó un 28.3% (15).
- Panamá Este (n=27) registró un 22.2% (6).
- La RS Metropolitana (n=40) reportó un 17.5% (7).
- En la RS de San Miguelito (n=40) se identificó un 10.0% (4).

No especificó su respuesta 1 persona en la RS de Colón, 1 en la RS Metropolitana y 2 en la RS de Panamá Este.

Gráfica No. 15

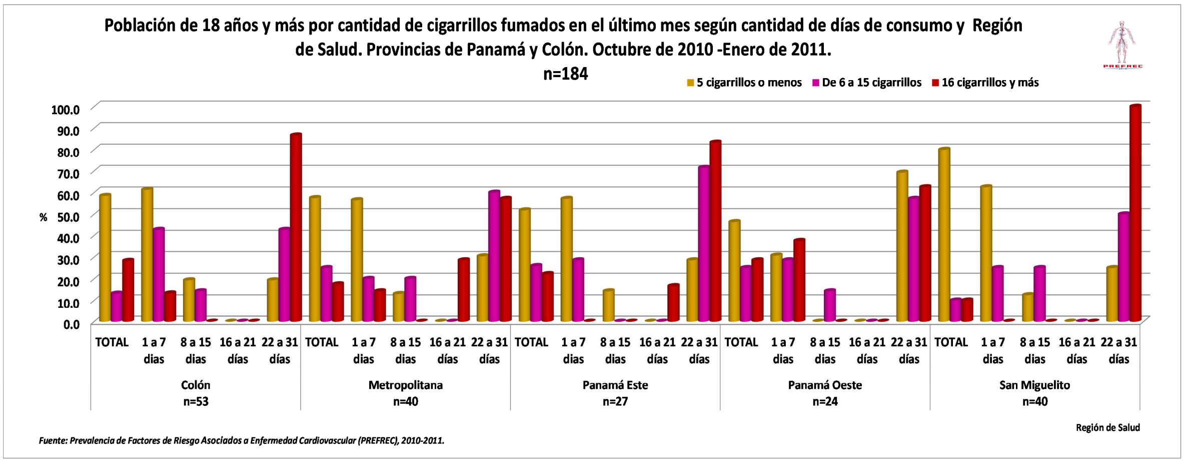

Tiempo transcurrido después de despertar para fumar cigarrillo

A los entrevistados que manifestaron haber consumido alguna vez en su vida productos de tabaco, se les abordó para conocer ¿qué tiempo después de despertar fumaban su primer cigarrillo? El 9.4% (98) de los encuestados indicaron que su primer cigarrillo lo fumaban menos de 10 minutos después de despertar; el 8.7% (91) manifestó que entre 10 a 30 minutos después de despertar fumaba su primer cigarrillo, en tanto que el 5.0% (52) indicó que entre 31 a 60 minutos. Cerca de 5 de cada 10 (46.0% ó 482 individuos) respondieron que el primer cigarrillo lo fumaban después de 60 minutos de haber despertado. Tres de cada 10 individuos (30.9%, es decir 324 personas) no especificaron su respuesta.

Respecto al total de hombres y mujeres que habían fumado alguna vez en su vida (623 y 424, respectivamente), 1 de cada 10 hombres (10.8%) fumaba su primer cigarrillo menos de 10 minutos después de despertar. El 10.1% (63) lo hacía de 10 a 30 minutos después de despertar, mientras que el 4.5% (28) lo hacía entre 31 a 60 minutos. Un 45.4% (283) de los hombres encuestados y que mencionaron fumar productos de tabaco alguna vez en la vida, consumía su primer cigarrillo después de 60 minutos, luego de despertar. Cerca de 3 de cada 10 (29.2%), no especificó su respuesta.

En el caso de las mujeres, el 19.6% (83) fumaba su primer cigarrillo durante los primeros 60 minutos luego de despertarse; 7.3% (31) menos de 10 minutos después de despertar; 6.6% (24) entre 10 a 30 minutos después de despertar y un 5.7% (24) entre 31 a 60

minutos después de despertar. Cerca de 5 de cada 10 (46.9%) consumían su primer cigarrillo después de 60 minutos de haber despertado. Hubo diferencias estadísticamente significativas entre el género y fumar el primer cigarrillo entre 30 minutos y menos después de despertar y más de 30 minutos después de despertar (x^2=6.09; p=0.0136).

Tal como lo indica la gráfica No. 16, de las 507 personas en el área urbana, que tenían historia de tabaquismo alguna vez en su vida, 1 de cada 10 (10.1%) fumaba su primer cigarrillo menos de 10 minutos después de despertar, un 8.3% (42) y el 5.3% (27) lo hacían de 10 a 30 minutos y de 31 a 60 minutos después de despertar, respectivamente. Cuatro de cada 10 (42.4%) después de 1 hora de haberse despertado, fumaba su primer cigarrillo. Uno de cada 3 entrevistados en esta área, no especificó su respuesta (33.9%).

En el área rural, el 9.6% (45), un 9.2% (43) y el 4.3% (20) de los 469 entrevistados que fumaron alguna vez en su vida, consumen su primer cigarrillo menos de 10 minutos, de 10 a 30 minutos y entre 31 y 60 minutos después de despertar, respectivamente. Cerca de 5 de cada 10 (48.6%) lo hace posterior a 60 minutos, luego de haber despertado. Dos de cada 7 entrevistados (28.4%) no especificaron su respuesta.

Para el área indígena, el 18.3% (13) de las 71 personas que indicaron haber fumado alguna vez en su vida, fuma o fumaba su primer cigarrillo durante la primera hora después de despertarse (2.4% menos de 10 minutos después de despertar; 8.5%(6) entre 10 a 30 minutos después de despertar y un 7.0%(5) entre 31 a 60 minutos después de despertar. Uno de cada 2 (54.9%) consumía su primer cigarrillo después de 60 minutos de haber despertado. El 26.8% (19) no especificó su respuesta.

Gráfica No. 16

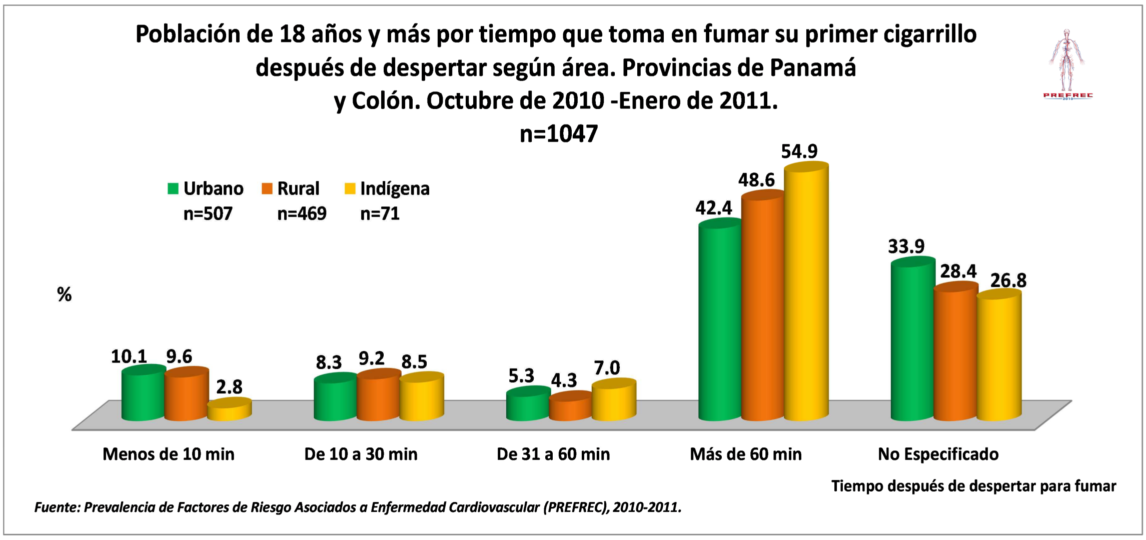

No se encontraron diferencias estadísticamente significativas entre las áreas de dominio de este estudio y las personas que fumaban su primer cigarrillo entre 30 minutos y menos después de despertar y más de 30 minutos después de despertar.

De acuerdo a la clasificación de la adultez, del total de los adultos jóvenes con historia de tabaquismo alguna vez en su vida (160), el 4.4%, 3.1% y 1.3% fuman su primer cigarrillo menos de 10 minutos, de 10 a 30 minutos y entre 31 a 60 minutos después de despertarse. Uno de cada 2 adultos jóvenes (54.4%) fuma su primer cigarrillo después de 60 minutos, luego de despertar. El 36.9% (59) no especificó su respuesta.

Con relación a los adultos, el 21.4% (115) consume su primer cigarrillo 60 minutos antes de despertar [10.0% (54) menos de 10 minutos después de despertar; 5.6%(30) entre 10 a 30 minutos después de despertar y un 5.8%(31) entre 31 a 60 minutos después de despertar]. El 46.5% (250) fuma su primer cigarrillo 1 hora después de haberse despertado. Cerca de 1 de cada 3 (32.2%, es decir, 173 individuos) no especificó su respuesta.

En cuanto a los adultos mayores que reportaron el consumo de productos de tabaco alguna vez en su vida (349), 1 de cada 10 hombres (10.6%) fumaba su primer cigarrillo menos de 10 minutos después de despertar. El 16.0% lo hacía de 10 a 30 minutos después de despertar, mientras que el 5.4% lo hacía entre 31 a 60 minutos. Cuatro de cada 10 (41.5%) de los adultos mayores encuestados consumían su primer cigarrillo después de 60 minutos, luego de despertar. Un 26.4% no especificó su respuesta.

Las pruebas de hipótesis formuladas reflejan diferencias estadísticamente significativas entre los adultos-adultos jóvenes que fumaban su primer cigarrillo 30 minutos antes y después de 30 minutos de despertar (x^2=5.33; p=0.0209); al igual que entre los adultos-adultos mayores (x^2=12.22; p=0.0005) y los adultos jóvenes-adultos mayores (x^2=19.51; p=0.0.00001) que fumaban su primer cigarrillo 30 minutos antes y después de 30 minutos de despertar.

A nivel de las RS, Colón presentó el mayor porcentaje entre las personas que fumaban su primer cigarrillo menos de 10 minutos después de despertarse (14.8%), seguido por la RS de San Miguelito con un 9.8% y la RS de Panamá Este (7.4%), gráfica No.17.

Gráfica No. 17

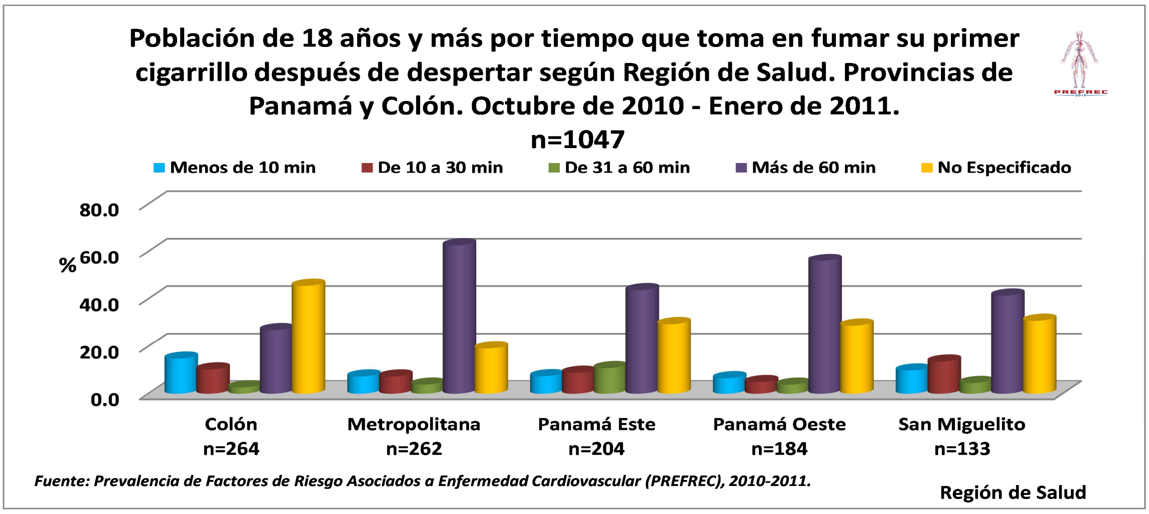

Edad de inicio de consumo de productos de tabaco a diario y su relación con el tiempo que toma el individuo para fumar su primer cigarrillo después de despertar

De los 467 entrevistados que manifestaron fumar a diario, el 1.7% (8) empezó a fumar a diario entre los 6 – 9 años, de los cuales un 25.0% (2) fuma su primer cigarrillo menos de 10 minutos después de despertar; 1 de cada 4 (25.0%) indicó que entre 10 a 30 minutos después de despertar fumaba su primer cigarrillo, dos personas (25.0%) respondieron que el primer cigarrillo lo fumaban después de 60 minutos de haber despertado, en tanto que el 25.0% restante (2 individuos) no especificaron su respuesta.

Las personas que empezaron a fumar a diario entre los 10 – 19 años fueron 256, de los cuales cerca de 1 de cada 5 (19.5%) fumaba su primer cigarrillo menos de 10 minutos después de despertar. El 16.0% (41) lo hacía de 10 a 30 minutos después de despertar, mientras que el 5.5% (14) lo hacía entre 31 a 60 minutos. Un 38.7% (99) de estos encuestados consumía su primer cigarrillo después de 60 minutos, luego de despertar. Uno de cada 5 (20.3%), no especificó su respuesta.

De los 155 individuos que iniciaron el consumo de productos de tabaco a diario entre los 20 y 29 años, el 37.4% (58) fumaba su primer cigarrillo durante los primeros 60 minutos luego de despertarse [11.6% (18) menos de 10 minutos después de despertar; 13.5%(21) entre 10 a 30 minutos después de despertar y un 12.3% (19) entre 31 a 60 minutos después de despertar]. Cuatro de cada 10 (43.2%) consumía su primer cigarrillo después de 60 minutos de haber despertado. No especificó su respuesta el 19.4% (30) de estos entrevistados.

En cuanto a los encuestados que empezaron a fumar a diario entre los 30 – 39 años (36), cerca de 1 de cada 5 (19.4%) fumaba su primer cigarrillo menos de 10 minutos después de despertar. El 11.1% (4) lo hacía de 10 a 30 minutos después de despertar, mientras que el 5.6% (2) lo hacía entre 31 a 60 minutos. Uno de cada 2 (50.0%) de estos entrevistados que fumaban a diario (18), consumían su primer cigarrillo después de 60 minutos, luego de despertar. El 13.9% (5) no especificó su respuesta.

Después de los 40 años de edad, empezaron a fumar a diario 11 personas, de las cuales el 45.5% (5) fumaba su primer cigarrillo durante los primeros 60 minutos luego de despertarse [9.1% (1) menos de 10 minutos después de despertar; 18.2%(2) entre 10 a 30 minutos después de despertar y un 18.2%(2) entre 31 a 60 minutos después de despertar]. Un 45.5% (5) lo hace posterior a 60 minutos, luego de haber despertado. El 9.1% de estos entrevistados (1) no especificó su respuesta.

Cantidad de cigarrillos fumados y tiempo que toma el individuo para fumar su primer cigarrillo después de despertar

Un análisis circunscrito a las personas que indicaron la cantidad de cigarrillos que fuman al mes y su relación con el tiempo que toma el individuo para fumar después de despertar indica que de las 668 personas que fumaban 5 cigarrillos o menos al mes, el 5.1% (34) fumaba su primer cigarrillo menos de 10 minutos después de despertar. El 6.0% (40) lo hacía de 10 a 30 minutos después de despertar, mientras que el 3.6% (24) lo hacía entre 31 a 60 minutos. Uno de cada 2, 52.5% (351) de los individuos encuestados consumían

su primer cigarrillo después de 60 minutos, luego de despertar. Cerca de 1 de cada 3 (32.8%, es decir, 219 personas), no especificaron su respuesta.

En el caso de los individuos que manifestaron fumar de 6 a 15 cigarrillos al mes (121 entrevistados), el 38.0% (46) fumaba su primer cigarrillo durante los primeros 60 minutos luego de despertarse (15.7% (19) menos de 10 minutos después de despertar; 11.6%(14) entre 10 a 30 minutos después de despertar y un 10.7%(13) entre 31 a 60 minutos después de despertar). Un 45.5% (55) lo hace posterior a 60 minutos, luego de haber despertado. El 16.5% de estos entrevistados (20) no especificaron su respuesta.

De los 201 fumadores pesados (personas que fuman 16 cigarrillos y más al mes) 2 de cada 10 (21.4%) fumaba su primer cigarrillo menos de 10 minutos después de despertar, un 17.4% (35) y el 6.0% (12) lo hacían de 10 a 30 minutos y de 31 a 60 minutos después de despertar, respectivamente. Uno de cada 3 (33.8% ó 68 encuestados) después de 1 hora de haberse despertado, fumaba su primer cigarrillo. El 21.4% (43) de estos individuos no especificó su respuesta.

De las 57 personas que no especificaron su respuesta, el 3.5% (2) de los encuestados indicaron que su primer cigarrillo lo fumaban menos de 10 minutos después de despertar; el 3.5%(2) manifestó que entre 10 a 30 minutos después de despertar fumaba su primer cigarrillo, en tanto que el 5.3%(3) indicó que entre 31 a 60 minutos. Un 14.0% (8 individuos) respondió que el primer cigarrillo lo fumaba después de 60 minutos de haber despertado. Siete de cada 10 individuos (73.7%, es decir 42 personas) no especificaron su respuesta.

Tiempo transcurrido después de despertar para fumar cigarrillo en las personas que fumaron en los últimos 30 días

La variable: “tiempo transcurrido después de despertar para fumar el primer cigarrillo” fue analizada con la variable “consumo de cigarrillos en el último mes o últimos 30 días”, evidenciando que el 16.5% (31) de estas personas reportaron que su primer cigarrillo lo fumaban menos de 10 minutos después de despertar; el 16.0% (30) manifestó que entre 10 a 30 minutos después de despertar fumaba su primer cigarrillo, en tanto que el 6.4% (12) indicó que entre 31 a 60 minutos. Uno de cada 2 (51.6% o 97 individuos) señaló que el primer cigarrillo lo fumaba después de 60 minutos de haber despertado. Un 9.6% (18 personas) no especificó su respuesta.

De acuerdo a la gráfica No. 18, un análisis comparativo con relación al total de hombres y mujeres que habían fumado cigarrillos en el último mes (125 y 63, respectivamente), evidenció que porcentualmente, más mujeres que hombres fumaban su primer cigarrillo menos de 10 minutos después de despertar, en tanto que más hombres fumaban su primer cigarrillo después de 60 minutos de despertar, sin diferencias estadísticamente significativas.

Gráfica No. 18

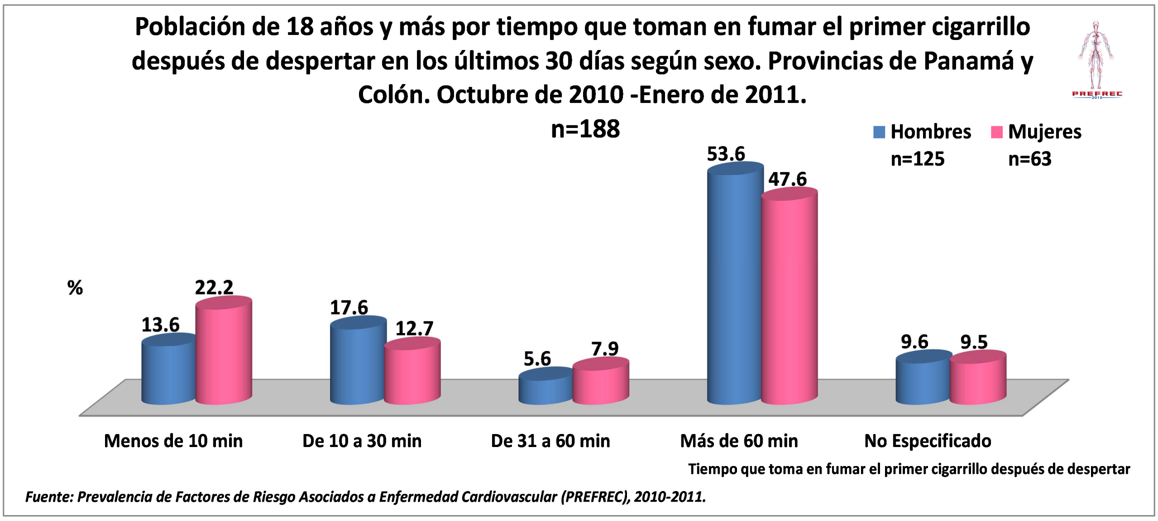

De las 188 personas que indicaron el consumo de cigarrillos en el último mes, el 54.3% (102) residían en el área urbana, de las cuales el 36.3% (37 individuos) fumaba su primer cigarrillo 60 minutos o menos después de despertar, mientras que 1 de cada 2 (55.9% o 57 personas) lo hacían 1 hora después de haberse despertado.

En el área rural (74), el 44.6% (33) fumaba su primer cigarrillo 60 minutos o menos después de despertar, en comparación con el 43.2% (32 individuos) que lo hacía 60 minutos posterior a despertarse; 9 personas (12.2%) no especificaron su respuesta.

En el caso del área indígena (12), 1 de cada 4 (25.0%, es decir, 3 individuos) fumaba su primer cigarrillo 1 hora o menos después de despertarse, en tanto que 2 de cada 3 fumadores en esta área (66.7%) lo hacía pasados los 60 minutos después que se habían despertado. Una persona no especificó su respuesta (gráfica No. 19).

Gráfica No. 19

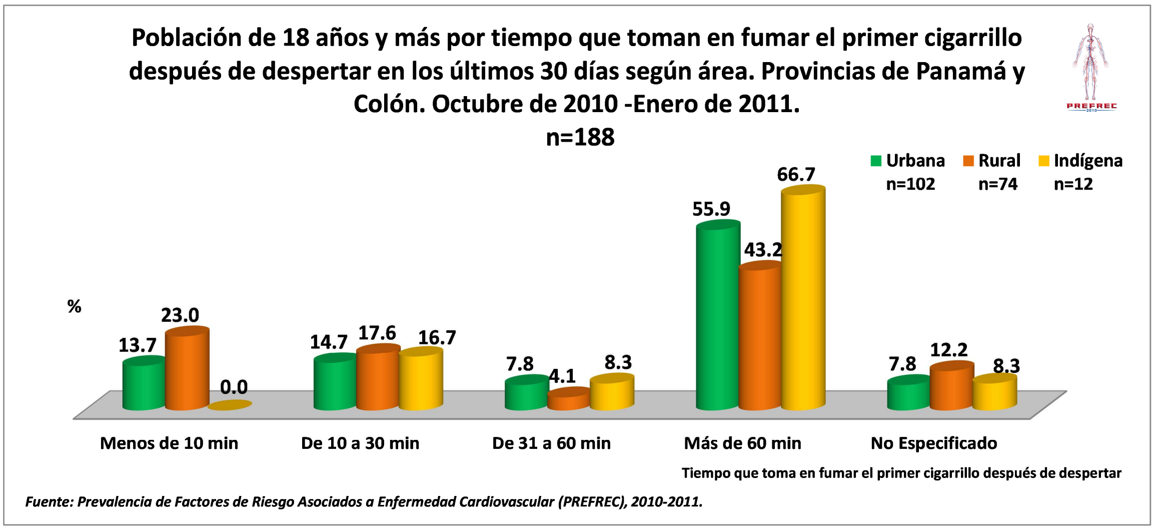

No se encontraron diferencias estadísticamente significativas entre las áreas de dominio de este estudio y las personas que fumaban su primer cigarrillo entre 30 minutos y menos después de despertar y más de 30 minutos después de despertar.

De acuerdo a la clasificación de la adultez, del total de los adultos jóvenes con historia de tabaquismo en el último mes (35), el 2.9% (1) y el 8.6% (3) fuman su primer cigarrillo menos de 10 minutos y entre 10 a 30 minutos después de despertarse.

Ocho de cada 10 adultos jóvenes (80.0%, es decir, 28 individuos) fuma su primer cigarrillo después de 60 minutos, luego de despertar. El 8.6% (3) no especificó su respuesta.

Con relación a los adultos, el 37.8% (37) consume su primer cigarrillo 60 minutos antes de despertar [19.4% (19) menos de 10 minutos después de despertar; 9.2% (9) entre 10 a 30 minutos después de despertar y un 9.2% (9) entre 31 a 60 minutos después de despertar]. El 57.1% (56) fuma su primer cigarrillo 1 hora después de haberse despertado. El 5.1% (5) no especificó su respuesta.

En cuanto a los adultos mayores que reportaron el consumo de cigarrillos en los últimos 30 días respecto al momento de aplicación de la encuesta (55), 2 de cada 10 hombres (20.0%) fumaba su primer cigarrillo menos de 10 minutos después de despertar. El 32.7% (18) lo hacía de 10 a 30 minutos después de despertar, mientras que el 7.3% (4) lo hacía entre 31 a 60 minutos. Uno de cada 5 (21.8%) de los adultos mayores encuestados consumía su primer cigarrillo después de 60 minutos, luego de despertar. Un 18.2% (10) no especificó su respuesta.

Las pruebas de hipótesis formuladas reflejan diferencias estadísticamente significativas entre los adultos y adultos mayores que fumaban su primer cigarrillo 30 minutos antes y después de 30 minutos de despertar (x^2=13.37; p=0.0003); al igual que entre los adultos jóvenes y adultos mayores (x^2=18.54; p=0.00002) que fumaban su primer cigarrillo 30 minutos antes y después de 30 minutos de despertar.

A nivel de las RS, Colón presentó el mayor porcentaje entre las personas que fumaban su primer cigarrillo menos de 10 minutos después de despertarse y que habían fumado en el último mes (28.3%), seguido por la RS Metropolitana (15.0%), la RS de Panamá Este (14.8%), Panamá Oeste (10.7%) y la RS de San Miguelito con un 7.5%, gráfica No.20.

Hace qué tiempo dejó de fumar

A las personas que mencionaron haber fumado alguna vez en su vida (1047), se les interrogó para saber si habían dejado de fumar y hace qué tiempo lo hicieron. Las opciones de respuesta que presentó la encuesta fueron clasificadas como: no he dejado de fumar, un año o menos, y más de 1 año.

Gráfica No. 20

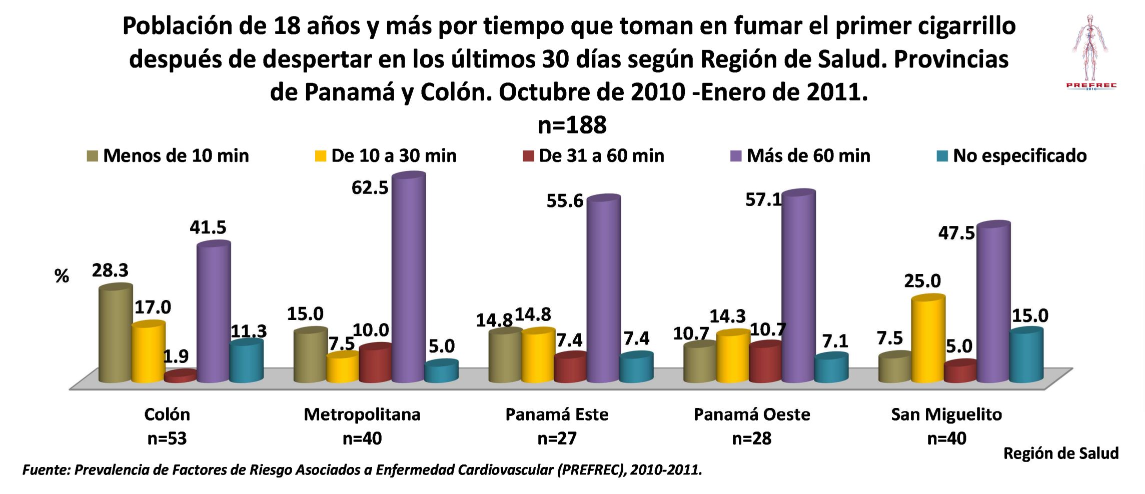

No he dejado de fumar

Los resultados indican que el 17.5% (183) de los entrevistados respondieron que no habían dejado de fumar y 26 personas no especificaron su respuesta (2.5%). El análisis según sexo muestra que del total de personas que han consumido productos de tabaco alguna vez en su vida (623 hombres y 424 mujeres) 19.9% de los hombres (124) y 13.9% de las mujeres (59), aun eran fumadores, presentando diferencias estadísticamente significativas entre géneros, la condición de ser fumador y haber dejado de fumar (x^2=5.23; p=0.0221). No especificaron su respuesta el 1.8% de los hombres y el 3.5% de las mujeres.

De las personas que habían fumado alguna vez en su vida encontramos que en el área urbana (507), el 20.1% (102) mencionó que no había dejado de fumar, al igual que el 14.3% (67) de los entrevistados que manifestaron historia de tabaquismo alguna vez en su vida en el área rural (469). Cerca de 1 de cada 5 (19.7%) de los individuos que fumaron alguna vez en su vida y que vivían en el área indígena (71) no habían dejado de fumar. No especificaron su respuesta el 3.9% y 1.3% de estos entrevistados en el área urbana y rural, respectivamente.

Manifestaron no haber dejado de fumar, según RS:

- El 18.2% de los encuestados en la RS de Colón (n=264).
- El 17.6% de los entrevistados en la RS Metropolitana (n=262).
- Un 15.7% del total de individuos que participaron en el estudio en la RS de Panamá Este (n=204).
- El 16.3% de los participantes de la RS de Panamá Oeste (n=184).
- Uno de cada 5 (20.3%) de los individuos encuestados en la RS de San Miguelito (n=133).

Un año o menos
Esta opción de respuesta fue manifestada por el 3.4% (36) de los encuestados. El comportamiento según sexo indica que del total de los entrevistados con historia de tabaquismo alguna vez en su vida (623 hombres y 424 mujeres) el 3.5% de los hombres (22) y el 3.3% de las mujeres (14), tenían un año o menos de haber dejado de fumar, sin diferencias estadísticamente significativas entre géneros.

De los individuos que habían consumido productos de tabaco alguna vez en su vida, se evidenció que en el área urbana (507), el 3.4% (17) reportó que había dejado de fumar hace doce meses o menos, al igual que el 3.8% (18) de todos los entrevistados que manifestaron historia de tabaquismo alguna vez en su vida en el área rural (469), así como el 1.4% de las personas que vivían en el área indígena y que fumaron alguna vez en su vida (71).

Según RS, manifestaron haber dejado de fumar hace un año o menos:

- El 4.9% de los individuos que fumaron alguna vez en la vida en la RS de Colón (n=264).
- El 5.0% de los entrevistados en la RS Metropolitana que indicaron haber fumado alguna vez en su vida (n=262).
- Un 1.5% del total de individuos que participaron en el estudio en la RS de Panamá Este (n=204) y que habían fumado alguna vez en la vida.
- El 2.2% de los participantes que habían fumado alguna vez en la vida y que residían en la RS de Panamá Oeste (n=184).
- Un 2.3% de los individuos que fumaron alguna vez en la vida y que fueron encuestados en la RS de San Miguelito (n=133).

Más de 1 año
Cerca de 3 de cada 4 (74.6%, es decir, 781 personas) manifestaron que habían dejado de fumar hace más de 1 año. No especificaron su respuesta el 2.0% (21) de los encuestados.

El distribución según sexo refleja que del total de los entrevistados con historia de tabaquismo alguna vez en su vida (623 hombres y 424 mujeres) el 72.9% de los hombres (454) y el 77.1% de las mujeres (327), tenían más de un año de haber dejado de fumar, con diferencias estadísticamente significativas entre géneros (x^2=5.33; p=0.0209).

Haber dejado de fumar hace más de 1 año ó 12 meses, fue la respuesta dada por 7 de cada 10 encuestados (70.8%) de las 507 personas que habían consumido productos de tabaco alguna vez en su vida en el área urbana. Cerca de 8 de cada 10 entrevistados (78.0% y 78.9%, respectivamente), tanto en el área rural (n=469) e indígena (n=71) indicaron la misma respuesta. No especificaron su respuesta el 1.8% y 2.6% de estos entrevistados en el área urbana y rural, respectivamente.

A nivel de las RS, reportaron haber dejado de fumar hace más de un año:

- Siete de cada 10 (72.0%) de los encuestados que fumaron alguna vez en la vida en la RS de Colón (n=264).
- El 76.3% de los entrevistados en la RS Metropolitana (n=262) y que habían fumado alguna vez en su vida.
- Un 76.0% del total de individuos que participaron en el estudio en la RS de Panamá Este (n=204) y con historia de tabaquismo alguna vez su vida.
- Ocho de cada 10 (81.0%) de los participantes de la RS de Panamá Oeste (n=184) que fumaron alguna vez en su vida.
- Seis de cada 10 (65.4%) de los individuos encuestados que habían fumado alguna vez en su vida y que eran residentes en la RS de San Miguelito (n=133).

Exposición al humo de productos de tabaco en los últimos 30 días

A los participantes de esta investigación se les preguntó si en los últimos 30 días o en el último mes, alguien había fumado en su presencia, ya sea en su casa, trabajo, centro de recreación u otros sitios públicos (gráfica No.21).

Gráfica No. 21

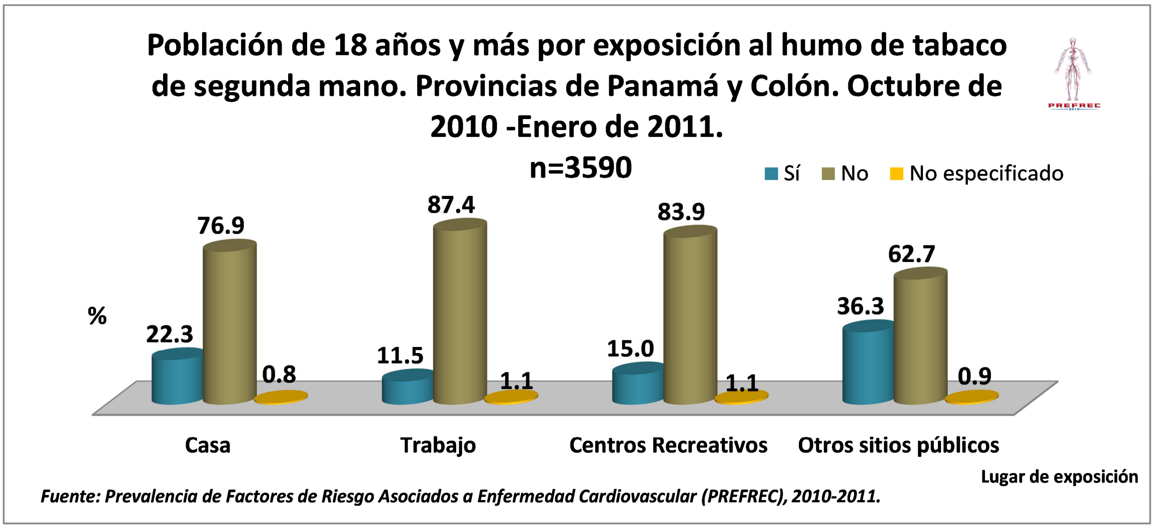

En casa

De los 3590 entrevistados, el 22.3% (799) manifestó que sí habían fumado en su presencia (en su casa); un 76.9% indicó que no habían fumado en su presencia dentro de su casa, mientras que el 0.8% (29 personas) no especificó su respuesta. De todos los hombres que participaron en este estudio (1074), el 16.7% (179), indicó que había estado expuesto al humo de tabaco de segunda mano en casa, en tanto que el 24.6% (620) del total de las mujeres (2516) también lo afirmaron, con diferencias estadísticamente significativas entre mujeres y hombres (x^2=27.79; p=0.0000001). El 0.6% de los hombres (6) no especificó su respuesta, al igual que el 0.9% de las mujeres (23).

Del total de las personas entrevistadas en el área urbana (1688), 2 de cada 10 (22.7%) indicaron que en los últimos 30 días alguien ha fumado en su presencia dentro de su

casa. Uno de cada 5 (21.0%, es decir, 357 individuos) de los encuestados en el área rural (1699) también lo manifestaron, así como el 29.1% (59) de las 203 personas que residían en el área indígena, con diferencias estadísticamente significativas entre el área rural-indígena (x^2=6.34; p=0.0118).

Según la clasificación de la adultez, del total de adultos jóvenes (735), cerca de 1 de cada 4 (24.4%) reportó que alguien había fumado en su presencia, estando en casa; en el caso del total de los adultos (2064), el 21.5% (443) también lo señaló; así como el 22.4% (177) del total de los adultos mayores (791). No se encontraron diferencias estadísticamente significativas entre la condición de adulto-adulto joven; adulto-adulto mayor y adulto joven-adulto mayor.

El comportamiento del indicador: exposición al humo de tabaco de segunda mano en los últimos 30 días en la casa, según RS indica que manifestaron esta condición:

- El 19.7% de los encuestados en la RS de Colón (n=1031).
- El 23.0% de los entrevistados en la RS Metropolitana (n=813).
- Uno de cada 4 (25.5%) del total de individuos que participaron en el estudio en la RS de Panamá Este (n=667).
- El 19.8% de los participantes de la RS de Panamá Oeste (n=555).
- Cerca de 1 de cada 4 (24.6%) de los individuos encuestados en la RS de San Miguelito (n=524).

En el trabajo

Esta opción de respuesta fue indicada por el 11.5% (412) del total de los encuestados, respondiendo que sí habían fumado en su trabajo, estando el entrevistado presente. El 87.4% reportó que no habían fumado en su presencia en el área laboral, mientras que el 1.1% (39) no especificó su respuesta.

De acuerdo al total de hombres y mujeres (1074 y 2516), más individuos del sexo masculino respecto al femenino manifestaron que en los últimos 30 días, alguien había fumado en el trabajo y en su presencia (24.2% y 6.0%), respectivamente. Las pruebas de hipótesis utilizadas señalan diferencias altamente significativas entre géneros (x^2=241.64; p=0.0000). El 0.8% de los hombres (9) no especificó su respuesta, al igual que el 1.2% de las mujeres (30).

El análisis según área refleja que del total de las personas entrevistadas en el área urbana (1688), el 12.7% (215) indicaron que en los últimos 30 días alguien ha fumado en su presencia en su área laboral. Esta situación también fue reportada por uno de cada 10 (10.5% ó 179) de todos los encuestados en el área rural (1699). De igual manera, el 8.9% (18) de las 203 personas que residían en el área indígena, también estuvieron expuestas al humo de tabaco de segunda mano en el trabajo. Solo se encontraron diferencias estadísticamente significativas entre el área urbano-rural (x^2=4.01; p=0.0451).
De acuerdo a la clasificación de la adultez, del total de adultos jóvenes (735), el 8.8% (65) indicó que alguien había fumado en su presencia, estando en su trabajo en los últimos 30

días; del total de los adultos (2064), el 14.1% (291) también lo manifestó; al igual que el 7.1% (56) del total de los adultos mayores (791). Pruebas de significancia estadística evidencian diferencias estadísticamente significativas entre la exposición a humo de tabaco de segunda mano en el trabajo y la condición de adulto-adulto joven (x^2=13.07; p=0.0003), y para la condición de adulto-adulto mayor (x^2=25.48; p=0.0000004).

En cuanto al comportamiento del indicador: exposición al humo de tabaco de segunda mano en los últimos 30 días en el trabajo, podemos mencionar que manifestaron esta condición según RS:

- El 11.5% de los encuestados en la RS de Colón (n=1031).
- El 11.1% de los entrevistados en la RS Metropolitana (n=813).
- Un 9.9% del total de individuos que participaron en el estudio en la RS de Panamá Este (n=667).
- El 13.0% de los participantes de la RS de Panamá Oeste (n=555).
- Un 12.4% de los individuos encuestados en la RS de San Miguelito (n=524).

Centros recreativos

La exposición del encuestado al humo de tabaco de segunda mano en centros recreativos fue afirmado por el 15.0% (537) de los entrevistados. Ocho de cada 10 (83.9%) indicó que no habían fumado en su presencia en centros recreativos, en tanto que el 1.1% (40) no especificó su respuesta.

De todos los hombres encuestados (1074), el 22.1% (237), indicó que habían estado expuestos al humo de tabaco de segunda mano en centros recreativos en los últimos 30 días, mientras que el 11.9% (300) del total de las mujeres (2516) también lo afirmaron, con diferencias estadísticamente significativas entre hombres y mujeres (x^2=59.94; p=0.0000). El 1.0% de los hombres (11) no especificó su respuesta, al igual que el 1.2% de las mujeres (29).

En el área urbana, de los 1688 individuos que allí residían, el 14.9% (251) manifestaron que en los últimos 30 días, estando en centros recreativos, alguien había fumado en su presencia. En el caso de los 1699 entrevistados del área rural, el 13.9% (237) indicó estar expuesto al humo de tabaco de segunda mano en centros recreativos. El mayor porcentaje de las personas que reportaron la exposición al humo de tabaco de segunda mano, se encontraban en el área indígena, siendo el 24.1% (49) del total de encuestados en dicha área (203). Se encontraron diferencias estadísticamente significativas en el área urbano-indígena (x^2=10.56; p=0.0012) y entre el área rural-indígena (x^2=13.76; p=0.0002).

Del total de adultos jóvenes (735) que participaron en este estudio, 1 de cada 5 (20.8%) reportó que alguien había fumado en su presencia, estando en centros recreativos; del total de los adultos (2064), el 14.0% (288) también lo indicó, al igual que el 12.1% (96) del total de los adultos mayores (791). Las relaciones entre las clasificaciones de adultez fueron altamente significativas, siendo la condición de:

- Adulto-adulto joven: (x^2=18.69; p=0.0000154).

- Adulto-adulto mayor: (x^2=24.60; p=0.0000007).
- Adulto joven-adulto mayor: (x^2=59.24; p=0.0000).

La pregunta:¿en los últimos 30 días alguien ha fumado en su presencia en un Centro Recreativo?, evidencia que señalaron esta condición según RS:

- El 13.9% de los encuestados en la RS de Colón (n=1031).
- El 15.7% de los entrevistados en la RS Metropolitana (n=813).
- Un 14.2% del total de individuos que participaron en el estudio en la RS de Panamá Este (n=667).
- El 18.7% de los participantes de la RS de Panamá Oeste (n=555).
- Un 12.8% de los individuos encuestados en la RS de San Miguelito (n=524).

Otros sitios públicos

El 36.3% (1304) de los encuestados en esta investigación manifestaron que en los últimos 30 días alguien ha fumado en su presencia mientras se encontraban en otros sitios públicos y seis de cada 10 (62.7%) indicaron que no habían fumado en su presencia en otros sitios públicos. Un 0.9% (34) no especificó su respuesta.

El análisis según género evidencia que del total de hombres y mujeres (1074 y 2516), 4 de cada 10 hombres (41.3%) y 3 de cada 10 mujeres (34.2%) señalaron que en los últimos 30 días, alguien había fumado en su presencia estando en otros sitios públicos, presentando diferencias altamente significativas entre géneros (x^2=15.50; p=0.0000826). El 0.6% de los hombres (6) no especificó su respuesta, al igual que el 1.1% de las mujeres (28).

El 39.0% (658) del total de habitantes en el área urbana (1688) indicó que en los últimos 30 días alguien había fumado en su presencia al encontrarse en "otros" sitios públicos. Uno de cada 3 (580 encuestados) que representan el 34.1% del total de entrevistados en el área rural (1699) también manifestaron esta condición, al igual que el 32.5% (66) del total de individuos que respondieron a esta pregunta en el área indígena (203). Las pruebas de hipótesis aplicadas, reflejaron diferencias estadísticamente significativas solo en la relación del área urbana-rural (x^2=13.76; p=0.0002).

En cuanto a la clasificación de la adultez, del total de adultos jóvenes (735), el 37.0% (272) indicó que alguien había fumado en su presencia, estando en otros sitios públicos en los últimos 30 días; del total de los adultos (2064), el 37.8% (781) también lo señaló; al igual que el 31.7% (251) del total de los adultos mayores (791). Pruebas de significancia estadística solo evidencian diferencias estadísticamente significativas entre la exposición a humo de tabaco de segunda mano en otros sitios públicos y la condición de adulto-adulto mayor (x^2=8.31; p=0.0039368) y adulto joven-adulto mayor (x^2=4.01; p=0.0453).

En cuanto a la exposición de humo de tabaco de segunda mano en otros sitios, según RS, esta investigación evidencia que esta condición fue reportada por:

- El 35.4% de los encuestados en la RS de Colón (n=1031).

- Cerca de 4 de cada 10 (37.6%) de los entrevistados en la RS Metropolitana (n=813).
- Un 14.2% del total de individuos que participaron en el estudio en la RS de Panamá Este (n=667).
- El 18.7% de los participantes de la RS de Panamá Oeste (n=555).
- Un 12.8% de los individuos encuestados en la RS de San Miguelito (n=524).

Tiempo de exposición al humo de tabaco de segunda mano
También se les preguntó a los participantes de este estudio si recordaban cuánto tiempo hacía que alguien había fumado en su presencia. Las respuestas fueron clasificadas como 1 mes (30 días o menos) y más de 1 mes o más de 30 días.

Un mes (30 días o menos)
De las 3590 personas encuestadas, 1 de cada 2 (55.2%, es decir 1983 individuos) manifestó que alguien había fumado en su presencia hacía un mes (30 días o menos), mientras el 44.8% (1607) indicó que no habían fumado en su presencia en los últimos 30 días.

De todos los hombres que participaron en este estudio (1074), cerca de 6 de cada 10 (59.7%), indicaron que alguien había fumado en su presencia hacía 30 días o menos, en tanto que 1 de cada 2 (53.3%) del total de las mujeres (2516) también lo afirmaron, con diferencias estadísticamente significativas entre hombres y mujeres (x^2=12.00; p=0.0005).

El comportamiento de esta variable en las áreas de dominio de este estudio refleja que de los 1688 entrevistados en el área urbana, cerca de 6 de cada 10 (58.4%) señalaron que hace 30 días o menos alguien fumó en su presencia y 4 de cada 10 individuos (41.6%) dijo que no habían fumado en su presencia.

En el área rural (1699 entrevistados) 1 de cada 2 (53.1%) reportó que hace 30 días o menos alguien consumió productos de tabaco fumado en su presencia. El 46.9% indicó que no habían fumado en su presencia.

En el caso del área indígena (203), se encontró el menor porcentaje de las tres áreas, donde el 46.8% de los entrevistados dijeron que hace 30 días o menos alguien fumó en su presencia. Uno de cada 2 entrevistados en esta área (53.2%) mencionó que no habían fumado en su presencia en el último mes.

El estar expuesto al humo de tabaco de segunda mano en los últimos 30 días o el último mes en las RS, fue manifestado por:

- Seis de cada 10 entrevistados (61.1%) de los encuestados en la RS de Colón (n=1031).
- Uno de cada 2 (53.6%) de los entrevistados en la RS Metropolitana (n=813).
- Cerca de 5 de cada 10 (49.8%) del total de individuos que participaron en el estudio en la RS de Panamá Este (n=667).
- El 56.2% de los participantes de la RS de Panamá Oeste (n=555).

- Uno de cada 2 (52.1%) de los individuos encuestados en la RS de San Miguelito (n=524).

Más de 30 días o más de 1 mes
Respecto la exposición al humo de tabaco se segunda mano hace más de 1 mes o más de 30 días, el 26.4% (946) reportó que hace más de 1 mes o más de 30 días, alguien había fumado en su presencia, en tanto que el 55.2% (1983) indicó que no habían fumado en su presencia hace más de 1 mes. No especificaron su respuesta 661 individuos, los cuales representan el 18.4%.

De acuerdo al total de hombres y mujeres que participaron en esta investigación (1074 y 2516), más individuos del sexo femenino respecto al masculino (mínimamente) manifestaron que hace más de 1 mes, alguien había fumado en su presencia (25.3% y 26.8%), respectivamente. El 59.7% de los hombres y el 53.3% de las mujeres indicaron que hace más de 1 mes, no habían fumado en su presencia, sin diferencias estadísticamente significativas. Un 15.0% de los hombres cerca de 1 de cada 5 mujeres (19.9%), no especificaron su respuesta.

El análisis del tiempo de exposición al humo de tabaco de segunda mano según área indica que de los 1688 entrevistados en el área urbana, el 23.3% (393) mencionó que hace más de 1 mes o más de 30 días alguien fumó en su presencia; cerca de 6 de cada 10 individuos (58.4%) contestó que no habían fumado en su presencia. El 18.3% (309) no especificaron su respuesta.

En el área rural (1699 entrevistados) cerca de 2 de cada 7 individuos (27.5%, es decir, 468) manifestaron que hace más de 1 mes alguien consumió productos de tabaco fumado en su presencia; uno de cada 2 (53.1%) refirió que no habían fumado en su presencia. Las personas que no especificaron su respuesta sumaron un 19.4%.

Entre los encuestados de las tres áreas de estudio, los habitantes del área indígena reportaron el mayor tiempo de exposición al humo de tabaco, señalando 4 de cada 10 individuos (41.9%) del total de los entrevistados en esta área (203) que alguien había fumado en su presencia hace más de 1 mes. El 46.8% de los entrevistados en esta área (95) indicaron que no habían fumado en su presencia hace más de 1 mes. La no respuesta (no especificado) alcanzó el 11.3%.

Que alguien haya fumado en presencia del entrevistado hace más de 1 mes o más de 30 días en las RS, fue señalado por:

- Cerca de 1 de cada 4 entrevistados (24.8%) de los encuestados en la RS de Colón (n=1031).
- Un 27.8% de los entrevistados en la RS Metropolitana (n=813).
- El 14.2% del total de individuos que participaron en el estudio en la RS de Panamá Este (n=667).
- Uno de cada 5 (20.4%) de los participantes de la RS de Panamá Oeste (n=555).

- Tres de cada 10 (30.0%) de los individuos encuestados en la RS de San Miguelito (n=524).
