# Supplementary material for: The Association of Tobacco Control Policies and the Risk of Acute Myocardial Infarction Using Hospital Admissions Data
Source: PLoS One. 2014 Feb 10;9(2):e88784. doi: 10.1371/journal.pone.0088784 (PMC3919809; doi:10.1371/journal.pone.0088784)
Supplement: Document S6 — Tobacco Importation in Panama Report. (PDF) [file pone.0088784.s007.pdf]

INSTITUTO CONMEMORATIVO GORGAS DE ESTUDIOS DE LA SALUD

Análisis de la demanda de tabaco en Panamá y el control del efecto asequibilidad con medidas fiscales y control del contrabando

Implicaciones para Política Fiscal 2000-2011

Informe de investigación

345.023

H433 Herrera Ballesteros, Víctor Hugo

Análisis de la demanda de tabaco en Panamá y el control del efecto asequibilidad con medidas fiscales y control del contrabando : Implicaciones para Política Fiscal 2000-2011 / Víctor Hugo Herrera Ballesteros . – Panamá : Instituto Conmemorativo Gorgas de Estudios de la Salud, 2013.

112p. ; 28 cm.

ISBN 978-9962-699-03-3

Tabla de contenido

Página

Agradecimientos ... 5

Investigadores ... 6

Prólogo ... 7

Introducción ... 8

I. Caracterización de la actividad económica del comercio de tabaco ... 11

1.1 Empleo que genera el comercio de tabaco ... 12

1.2 Determinación de los precios y su estructura ... 12

1.3 Caracterización de la importación y distribución de cigarrillos ... 14

II. Evolución del consumo de productos derivados del tabaco ... 15

2.1 Comportamiento del consumo de cigarrillos ... 15

2.2 Análisis de prevalencias ... 17

2.2.1 Prevalencia nacional y por zona geográfica ... 17

2.2.2 Prevalencias por sexo ... 18

2.3 Medidas de control del consumo de productos derivados del tabaco ... 19

III. Comercio de productos del tabaco ... 21

3.1 Evolución de las importaciones de productos del tabaco ... 21

3.2 Origen de las importaciones de productos del tabaco ... 23

3.3 Restricciones al comercio ... 24

3.4 Situación del contrabando de productos del tabaco ... 24

IV. Evolución de los precios, impuestos, ingresos y elasticidades precio e ingreso de la demanda. 26

4.1 Evolución de los precios reales de los cigarrillos ... 26

4.2 Participación del gasto en cigarrillos respecto del gasto total 28

4.3 Evolución de la recaudación del Impuesto Selectivo al Consumo de Cigarrillos y otros productos derivados del tabaco (ISC) 28

4.4 Elasticidades precio e ingreso de demanda y simulación del impacto fiscal del aumento del ISC. 32

4.4.1. Análisis de la demanda y variables explicativas 35

4.4.2. Formalización de las variables de los modelos de elasticidades 37

4.4.3. Resultados de las estimaciones 41

4.4.4. Simulación del impacto fiscal de los incrementos del ISC 49

V. Resultados generales de las encuestas de marcas y de locales de atención al público 53

5.1. Encuesta de marcas 53

5.2. Encuesta de locales de atención al público 86

5.3. Implicaciones para futuras intervenciones en materia de control del consumo de tabaco .. 97

Conclusiones y recomendaciones en materia de política fiscal 99

Bibliografía 102

Anexos 104

Agradecimientos

Deseamos extender nuestro más sincero agradecimiento a quienes hicieron posible materializar este esfuerzo investigativo intitulado “Análisis de la demanda de tabaco en Panamá y el control del efecto asequibilidad con medidas fiscales y control del contrabando: Implicaciones para la Política Fiscal. Años 2000-2011”. Un especial agradecimiento al International Development Research Centre de Canadá, que bajo su auspicio se obtuvieron los fondos para el desarrollo de este proyecto y de igual manera al Instituto Conmemorativo Gorgas de Estudios de la Salud, que con su prestigio y capacidad administrativa se pudo contar con toda la logística y apoyo interinstitucional.

De igual forma es dable extender nuestro agradecimiento a aquellas instituciones que proporcionaron información y asesoría técnica, como el Ministerio de Salud, la Dirección General de Ingresos y el Instituto Nacional de Estadística y Censos, cuyo personal siempre estuvo anuente de atender nuestras consultas respecto al uso e interpretación de la información suministrada y a quienes pretendemos entregar este esfuerzo investigativo.

De manera muy especial deseamos extender nuestro agradecimiento a la Dra. Reina Roa Rodríguez, Directora de Provisión de Servicios de Salud del Ministerio de Salud, por su apoyo incondicional, en el desarrollo de esta investigación y poder contar con su experiencia y conocimientos indiscutibles en el tema del consumo de tabaco.

Investigadores

Víctor Hugo Herrera Ballesteros

Investigador principal

Inocencio Chávez
Investigador

Dra. Reina Roa Rodríguez
Investigadora

Carlos Gordón
Investigador

Beatriz Gómez
Coordinadora de proyectos

Prólogo

En Panamá al igual que en otros países de la región el tabaquismo alcanzó una connotación de epidemia, que ha tenido un impacto económico significativo sobre el sistema público sanitario, al igual que con relación a la carga social que representan las enfermedades crónicas relacionadas al consumo de tabaco, sobre todo respecto a los años de vida potencialmente perdidos.

Por otro lado es todavía exiguo el aporte fiscal de la industria lo cual justifica de manera indiscutible la aplicación de medidas fiscales y en general restrictivas al consumo de cigarrillos, que inhiban la demanda de los grupos etarios más jóvenes, quienes se convierten en víctimas potenciales del tabaquismo con nefastas consecuencias sociales y económicas. No obstante, Panamá es uno de los países a la vanguardia en la región, respecto de la adopción de medidas fiscales y de control de espacios libres de humo, al igual que de las restricciones en las promociones y publicidad de productos derivados del tabaco, mismas que se reforzaron a partir del año 2004 cuando el país se adhiere al Convenio Marco de la Organización Mundial de la Salud, mediante el cual se dota de nuevas herramientas normativas para el control del tabaquismo.

Los resultados de esta investigación han demostrado la efectividad y eficacia de las medidas de control del tabaquismo adoptadas desde principios de la década pasada, con una caída sostenida de las importaciones y de la prevalencias de consumo de tabaco en el ámbito nacional, al emplear las fuentes estadísticas disponibles, como por ejemplo, la Encuesta Nacional de Salud y Calidad de Vida de 2007 realizada por el ICGES y la Encuesta de Prevalencia de Factores de Riesgo Cardiovascular de 2010, realizada por el ICGES. De igual manera se aportaron datos frescos con las encuestas de marcas y de locales de atención al público, que validan los resultados anteriores, en lo que respecta a las tendencias de consumo, salvadas las diferencias metodológicas.

Por otro lado, un aspecto importante a destacar respecto de las encuestas realizadas de marcas y de percepciones en materia de conocimientos y actitudes contra el consumo de tabaco en locales de atención al público, fue la alta aceptación de la ley antitabaco, al igual que una favorable percepción de los efectos de la misma en materia económica y ambiental, aunque marginalmente en el caso de los restaurantes existe la presión de tener espacios para fumadores de acuerdo a estos resultados, lo cual no deja de ser preocupante. Otro resultado importante, es la medición general del contrabando, en un 28% en los dominios considerados (Panamá Centro, San Miguelito, David, Barú y Ciudad de Colón), lo cual deja en claro la asequibilidad de cigarrillos ilegales baratos (incluidos cigarrillos falsificados), especialmente para los grupos de menor ingreso.

Finalmente también resulta relevante el conocer que los cigarrillos ilegales han penetrado el canal de venta formal, especialmente en los supermercados, lo cual justifica reforzar las medidas de control de venta ilegal en el sector formal. Por tal razón es importante seguir con la aplicación de las medidas de control del consumo, fiscales y aduaneras para reducir las externalidades negativas del consumo de tabaco.

Introducción

Panamá es uno de los países en la región que ha mostrado avances significativos en el uso de medidas de control contra el tabaquismo, desde mediados de la década pasada a raíz de la adopción del Convenio Marco de la OMS para el control del tabaco en el año 2004, con medidas de diversa índole, tales como restricciones a la publicidad, prohibición de máquinas expendedoras de cigarrillos, uso de advertencias sanitarias, ambientes libres de humo, en adición a un fuerte aumento del Impuesto Selectivo al Consumo de Cigarrillos (ISC) y otros productos derivados del tabaco, entre otras, que han puesto al país a la vanguardia en la región.

Por otro lado, es importante señalar que la estructura productiva en materia de productos del tabaco, cambió a finales de la década del noventa, cuando la industria se reconvierte a importadora, pero de igual manera bajo una estructura duopólica, dado que el control del mercado tradicionalmente ha sido de las empresas British American Tobacco y la Phillip Morris. Por consiguiente, durante el período de investigación, que va de 2000 a 2011, se analiza la dinámica del mercado de una industria que es importadora y solo de cigarrillos, como el principal producto de consumo.

El impacto de las medidas de control a lo largo de más de una década han dejado su efecto en el consumo, mediante una reducción sistemática de las importaciones acompañada de una baja prevalencia nacional del 9.4%[1], que dan fe de los avances en dicha materia, sobre todo con miras a reducir los impactos negativos en el sector sanitario.

En ese sentido la reforma fiscal adoptada mediante la Ley 69[2] de noviembre de 2009, establece la medida fiscal más importante de las dos últimas décadas mediante el incremento del 100% del Impuesto Selectivo al Consumo de Cigarrillos y otros productos derivados del tabaco (ISC), mediante el cual el 50% de lo recaudado se distribuye de la siguiente manera: 40% al Ministerio de Salud, 40% al Instituto Oncológico Nacional y el 20% restante a la Autoridad Nacional de Aduanas, con lo cual se dota de recursos a estas entidades para combatir las externalidades del tabaquismo.

Con todo, lo más importante es que a dos años de aplicadas dichas medidas han podido evidenciarse los efectos en materia fiscal y dejar en claro su efectividad en el aumento de la recaudación del ISC y en la reducción de la demanda ante el incremento consabido del precio de los productos derivados del tabaco, específicamente de los cigarrillos.

No obstante, el incremento de la renta nacional por habitante en períodos cortos, han podido haber neutralizado en parte el efecto del incremento del ISC y hacer más asequible el consumo de cigarrillos y otros derivados del tabaco a corto plazo.

---

[1] Ministerio de Salud. Encuesta Nacional de Salud y Calidad de Vida 2007.

[2] Ley 69 de 11 de noviembre de 2009: Que reforma el Código Fiscal y adopta otras medidas fiscales. Gaceta Oficial Digital N° 26402-C de 6 de noviembre de 2009.

Por consiguiente el objetivo central de esta investigación es medir el efecto del incremento del ISC a finales del año 2009 y dejar en evidencia el impacto tienen las medidas fiscales con fundamento en la Curva de Laffer, a manera de justificar la necesidad de su uso continuado ante los incrementos en el ingreso nacional por habitante, dado que el mismo se constituye en un elemento neutralizador a corto y mediano plazo de las medidas fiscales para reducir el consumo. De igual manera mediante la aplicación de las encuestas de marcas y de locales de atención al público, se pretende medir el grado de contrabando y características del consumo al igual que de actitudes frente a las medidas de control de consumo de tabaco, en las principales zonas comerciales del país expuestas a la venta ilegal, en este caso, los distritos de Panamá, Colón, David, San Miguelito y Barú, las cuales serán analizadas en el último apartado, donde queda en evidencia como uno de los resultados generales, una medición del contrabando de cigarrillos del 28%, en adición a que el 54% de los entrevistados desconoce la ley antitabaco, dejando en evidencia la necesidad de seguir reforzando la aplicación de medidas fiscales y de combate al contrabando, el cual es funcional a la industria, como se desprende de los análisis pertinentes.

Las fuentes de información utilizadas para los análisis descriptivos y la construcción de los modelos de demanda de cigarrillos, fueron las series de comercio exterior del Instituto Nacional de Estadística y Censos, los datos de recaudación tributaria del ISC de la Dirección General de Ingresos del Ministerio de Economía y Finanzas, las Encuestas para medir prevalencias del Ministerio de Salud, los datos de retenciones de importaciones de la Autoridad Nacional de Aduanas, y los agregados macroeconómicos del Sistema de Cuentas Nacionales del Instituto Nacional de Estadística y Censo, entre otras fuentes primarias y secundarias de información.

Para la construcción de los modelos de demanda de cigarrillos se ha utilizado el Toolkit del Banco Mundial[3] para la estimación de las elasticidades de corto y largo plazo, con base en los modelos convencional, corrección de errores, adicción miope y adicción racional, a manera de poder establecer escenarios o simulaciones de la Curva de Laffer y tener un umbral de incremento del ISC que permita el sostenimiento de la recaudación fiscal y la reducción del consumo por otro lado.

De los resultados obtenidos, el modelo que presentó el mejor ajuste fue el modelo convencional, con una elasticidad precio de -0.82, dejando un umbral de incremento del ISC en 12%, a dos años del último incremento del 100%, toda vez que en esta oportunidad se parte de un precio promedio por cajetilla de 20 cigarrillos de B/. 3.63[4], versus el precio promedio de finales de 2009, de B/. 1.83 Empero, la Curva de Laffer, solo plantea un escenario probable sobre la base del descenso de las importaciones a lo largo del período de estudio.

Un elemento tenido en cuenta en nuestras discusiones temáticas, ha sido el signo negativo de la elasticidad ingreso de la demanda en todos los modelos, por el hecho de que las series históricas evidencian una relación inversa entre el ingreso y consumo, desde principios del período de

[3] World Bank. Economics of Tobacco Tool kit N° 3 Demand Analysis.

[4] Se refiere al promedio de las tres marcas más vendidas en la Ciudad de Panamá (Kool, Viceroy y Marlboro).

estudio, lo cual deja abierta la posibilidad de sesgo derivado de sub declaraciones aduaneras y de impuestos o bien de la presencia de contrabando, por lo que este estudio será complementado con los análisis de las encuestas de marcas y de percepción de los efectos de las medidas de control en locales de esparcimiento y diversión. Por otro lado, también se espera contrastar este aspecto con los resultados preliminares de la encuesta GATS en adultos a fines de este año, de manera que se tenga una estimación más reciente de las prevalencias en adultos y en jóvenes.

No obstante, estos resultados reflejan el hecho que las medidas impositivas se han estrechado, momentáneamente ante el último incremento del ISC. Pero un punto importante a discutir, es que las elasticidades no son estáticas en el tiempo, lo cual deja siempre abierta la posibilidad de cambios estructurales en el consumo que pueden afectar la interpretación de la Curva de Laffer, lo cual deja en claro su limitación respecto de consideraciones impositivas a largo plazo.

I. Caracterización de la actividad económica del comercio de tabaco.

La industria del tabaco en Panamá sufrió transformaciones importantes a partir del año 1999, cuando la industria tabaquera productora es desmantelada y reubicada en otros países de la región centroamericana. El cultivo de tabaco y fabricación de productos derivados, en particular cigarrillos, pasó a ser una actividad de importación a partir del año 2000. Ello establece un cambio estructural significativo en este sector, quedando apenas de manera casi imperceptible la producción de cigarros y puros, con fines artesanales. De hecho, su valor agregado es tan bajo que no es sujeto de registro en las encuestas agrícolas e industriales (Gráfica N° 1).

Gráfica N° 1

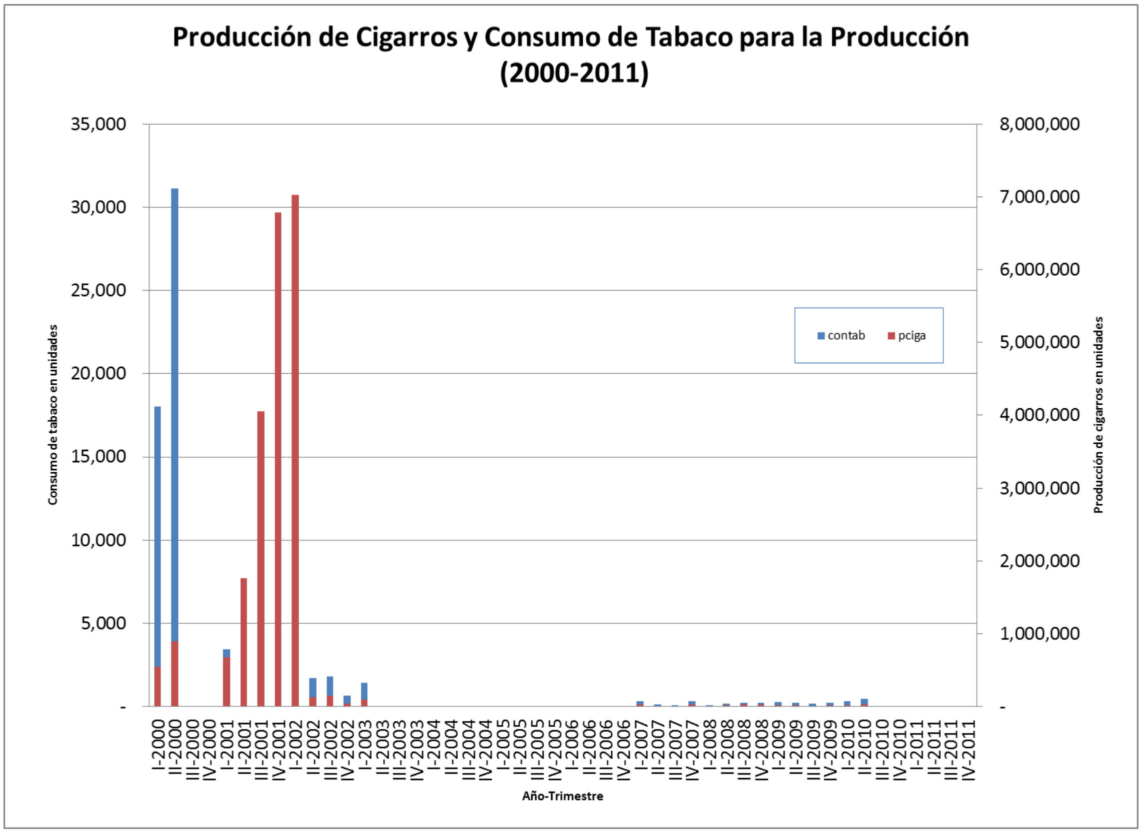

Fuente: Elaboración propia con datos de la Contraloría General de la República.

Con base en lo anterior, las dos empresas productoras Phillip Morris y British American Tobacco, se convirtieron en importadores-distribuidores, hasta la fecha. Siendo la importación de cigarrillos la más importante para ambas empresas y para el mercado de consumo de productos derivados del tabaco en general. No obstante, la estructura oligopólica se mantiene en la distribución tanto como lo fue con relación a la producción y ello deja en claro que las estrategias de fijación de los precios, es cónsona con este tipo de estructura de mercado.

1.1 Empleo que genera el comercio de tabaco.

Al convertirse las empresas productoras en importadoras-distribuidoras, de igual manera desaparece el empleo agrícola e industrial que las mismas generaban, quedando solo el empleo relacionado con la venta al por mayor y al por menor, por lo que no es posible establecer el empleo que genera esta actividad, pues está fusionada a la venta de otros productos tales como alimentos y bebidas, típicos de la venta mayorista y minorista en la cual se incluye la venta de productos derivados del tabaco, tal como se establece en el Directorio Nacional de Establecimientos de 2009.

Tabla N° 1
Empleo relacionado a productos derivados del tabaco
Ramas de actividad

Rama de actividad comercial	Código-Directorio de Establecimientos (2009)
1. Venta al por mayor de alimentos, bebidas y tabaco.	46309
2. Venta al por menor de alimentos, bebidas y tabaco en zonas francas.	48300
3. Venta al por menor en puestos de ventas y mercados de alimento, bebidas y tabaco.	47810

Fuente: Directorio Nacional de Establecimientos de 2009. Contraloría General de la República.

Por consiguiente al quedar solo la actividad comercial, el empleo en esta actividad es casi imperceptible o en el mejor de los casos es difícil de cuantificar, por el hecho de tener costos laborales y de distribución en general, compartidos con otros productos.

1.2 Determinación de los precios y su estructura.

Debido a que se trata de una actividad en la que se integra la importación, la distribución mayorista y venta minorista, la estructura impositiva se corresponde con la forma en que dicho comercio está constituido. Por otro lado, tal como se presenta en la siguiente tabla, la estructura impositiva es ad valorem y abarca los tres niveles de comercialización mencionados.

Tabla N° 2

Estructura impositiva a los cigarrillos y otros productos derivados del tabaco vigente desde noviembre de 2009

IMPUESTO	TASA VIGENTE	BASE DE IMPOSICIÓN	EN VIGENCIA POR PRIMERA VEZ	ÚLTIMA MODIFICACIÓN
Impuesto Selectivo al Consumo de Cigarrillos y otros Productos del Tabaco (ISC)	100 %	Precio de venta al consumidor declarado por el importador o productor	Noviembre 1995	Noviembre 2009
Arancel de importación	15 %	Valor CIF de importación	n.d.	Enero 1998
ITBMS (IVA)	15 %	Valor CIF más el arancel de importación	Diciembre 1976	Agosto 2005

La estructura impositiva consta de tres gravámenes a saber, el Impuesto Selectivo al Consumo de Cigarrillos y Otros Productos del Tabaco (ISC), el arancel de importación y el I.V.A. El cambio reciente más relevante en materia de impuestos al tabaco, fue noviembre de 2009, cuando el ISC se elevó del 32.5% al 100%, modificando sensiblemente la estructura de los precios y su repercusión en la venta minorista.

A continuación se presenta una reconstrucción hipotética de la estructura del precio hacia atrás, a partir del precio promedio de venta minorista, tomado en consideración que la composición del precio al por menor, recoge la estructura ad valoren de los impuestos a lo largo del canal de comercialización.

Tabla N° 3

Estructura de precios reconstruida a partir de datos de importaciones por cajetilla de 20 cigarrillos estimada con base en el precio promedio actual de las marcas principales

Valor C.I.F.	B/. 0.84
Arancel del importación	15.0%
I.V.A. Importación	15.0%
Costo de internación	B/. 1.09
**Margen del mayorista**	**B/. 0.47**
Precio del Mayorista	B/. 1.56
Impuesto Selectivo al Cigarrillo	100%
Precio del Mayorista + ISC	B/. 3.12

Margen del minorista	B/. 0.56
Precio del minorista	B/. 3.68
Margen neto del minorista	15.3%

Nota: Margen del mayorista = 43%, Margen del minorista = 18%.
Fuente: Información levantada con comerciantes minoristas y listas de precios al por mayor.

El incremento del ISC provocó una caída en el consumo de cigarrillos y otros productos derivados del tabaco, dada su naturaleza regresiva. Es importante señalar que el precio promedio es de las principales marcas en Panamá (Marlboro, Vicerroy y Kool). Según cálculos propios con datos del comercio minorista, el precio promedio por cajetilla era de B/. 1.83 hasta el año 2009. A partir del año 2010 con el incremento del ISC al 100%, el precio promedio se ha ubicado en B/. 3.63 por cajetilla (mismo que se aproximó a B/. 3.68 mediante la reconstrucción de los márgenes), es decir, como cabría esperar se duplicó.

Al mantenerse los demás impuestos constantes el incremento en el precio se explica por la variación del ISC. Por lo tanto, cabría esperar un efecto en la reducción del consumo, especialmente de los más pobres al igual que de los jóvenes menores de 18 años, aunque siempre queda latente la venta ilegal o contrabando como una opción de mercado que sigue permitiendo el acceso.

1.3 Caracterización de la importación y distribución de cigarrillos.

Como se ha mencionado, la comercialización de cigarrillos tiene un canal de comercialización muy corto, dado que está integrada a la distribución y venta al por menor. Con relación a la importación de cigarrillos, ambas tabacaleras realizan la importación y distribución mayorista. En el caso de la Empresa British American Tobacco, la Tabacalera Istmeña, es la que realiza la importación y distribución mayorista, mientras que Phillip Morris Panamá (Tabacalera Nacional), realiza esta operación para la Phillip Morris International.

Es un mercado oligopólico en el que las empresas distribuidoras están integradas verticalmente con la operación de importación, siendo una parte especializada del canal. Respecto de la venta al por menor, la misma se realiza bajo el formato multiproducto, en el cual en principio no está claro que existan o no relaciones de exclusividad.

Luego de la restructuración de la industria en la región centroamericana a principios de la década pasada, Panamá, quedó establecido como país importador de cigarrillos, producto que representa casi la totalidad de las importaciones, de productos derivados del tabaco, en mucho más de un 95% en promedio anual, para el período de investigación.

II. Evolución del consumo de productos derivados del tabaco.

El consumo de productos derivados del tabaco, ha experimentado reducciones consistentes desde principios de la década pasada, respecto del comportamiento de las importaciones. La posible causa gira en torno a la combinación de las medidas de control de espacios libres de humo de tabaco, al igual que con relación a las medidas fiscales.

No obstante, la gran interrogante gira en torno al contrabando y a la venta ilegal en general, la cual resulta difícil de medir, pero que de igual manera puede producir un sesgo en la estimación de la demanda real, más allá de lo que revelan las estadísticas formales.

Una medida general utilizada son las prevalencias de vida que según los resultados de las encuestas más recientes, tales como la Encuesta Mundial de Tabaco en Jóvenes de 2002 y 2008 (EMTJ-Panamá). Otra fuente de información es la Encuesta Nacional de Salud y Calidad de Vida (ENSCAVI-2007) y a fechas más recientes la Encuesta de Prevalencia de Factores de Riesgo a Enfermedad Cardiovascular (2010), dirigida a la población adulta de 18 años y más, en las provincias de Panamá y Colón, en las cuales se pueden observar reducciones en las prevalencias de vida y a 30 días.

De igual forma conviene hacer un análisis de la evolución histórica de las ventas obtenidas a partir de la base impositiva, la cual en 2009 pasó de 32.5% a 100%, respecto del ISC. Ello ligado al análisis de las prevalencias, permitirá obtener una mejor explicación del comportamiento del consumo para el período analizado.

2.1 Comportamiento del consumo de cigarrillos.

Un análisis aproximado del consumo puede obtenerse con relación a las ventas a partir de las bases impositivas del ISC[5]. En la Gráfica siguiente, se observa como desde el año 2000 la evolución de las ventas muestra una tendencia de largo plazo que es decreciente. No obstante, también se evidencian dos puntos de ruptura que dejan en claro cambios temporales en el patrón de consumo. Los cambios de tendencia observados se dieron en el año 2006 (primer trimestre), y 2009 (primer trimestre). Ello deja en claro que el consumo ha reflejado el impacto de las medidas fiscales y de control (espacios libres de humo) especialmente a partir del año 2004 con la adopción del Convenio Marco en 2004, mediante el cual se promulga el Decreto Ejecutivo N° 17 del 11 de marzo de 2005, introduciendo medidas tales como: prohibir fumar en restaurantes, las advertencias sanitarias y pictogramas que deben ocupar el 50% en los paquetes de productos del tabaco, se prohibió la venta de cigarrillos sueltos y en paquetes de menos de 20 unidades, la

[5] Esta es la información que fue suministrada por la Dirección General de Ingresos del Ministerio de Economía y Finanzas. La explicación ofrecida por el personal de esta institución es que por razones de confidencialidad no se pueden suministrar los datos de ventas de las empresas, toda vez que es una actividad dominada solo por dos empresas, siendo un mercado oligopólico en la importación y distribución de cigarrillos y otros derivados del tabaco.

prohibición del uso de máquinas expendedoras de cigarrillos, se prohíbe la publicidad en vallas y en medios de comunicación masivos, se circunscribe la publicidad únicamente en los puntos de venta y la promoción y el patrocinio solo a actividades de participación de la población adulta (mayores de 18 años).

Posteriormente mediante la Ley 69 de noviembre de 2009, elevó el ISC del 32.5% al 100%, coincidiendo con el segundo punto de ruptura observado en las ventas, con lo cual se marca una caída en las ventas, acompañada de un crecimiento de la recaudación del ISC, sobre todo a partir del primer trimestre del año 2011.

Esta situación abre la discusión respecto a las hipótesis que se quieren verificar más adelante con los modelos de demanda, con relación del consumo miope y racional, y con relación a los signos de la elasticidad ingreso de la demanda.

Gráfica N° 2

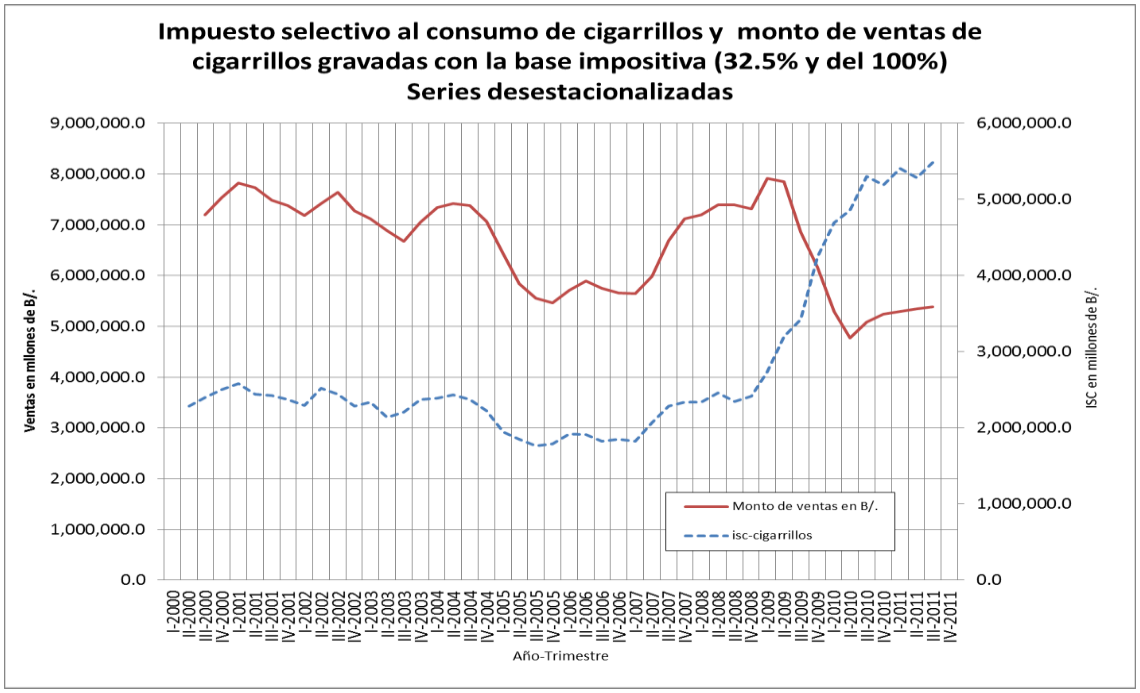

Fuente: Elaboración con datos de la Dirección General de Ingresos del Ministerio de Economía y Finanzas.

Lo interesante es que hasta antes del incremento del ISC al 100%, las ventas y el ISC tenían una tendencia similar, misma que se rompe a partir del cuarto trimestre de 2009, justo con el incremento del 100% y a partir del primer trimestre de 2011, se vuelve a recuperar dicho comportamiento paralelo entre ambas variables. Este comportamiento no es de extrañar dado

que se espera que ambas variables sean pro cíclicas a largo plazo, pero deja en evidencia el cambio de nivel en las ventas, con una ruptura de carácter permanente.

En general se ha evidenciado que el consumo de cigarrillos, como principal producto de consumo en Panamá, ha experimentado una caída a lo largo del período de estudio, con cambios de tendencia coincidentes con medidas de control (espacios libres de humo, publicidad, etc.) y medidas fiscales a partir de los años 2005 y 2010, evidenciando que las mismas realmente se hacen efectivas.

Este resultado deja en claro los incrementos en la recaudación, frente a la caída en las ventas a partir del año 2010, y la posible existencia de un sesgo de medición, abriendo el debate respecto al posible incremento del contrabando.

Los análisis de prevalencias a continuación permitirán complementar el análisis de la evolución del consumo de cigarrillos, con base en los instrumentos de recolección más recientes.

2.2 Análisis de prevalencia.

En este apartado se analizaran las prevalencias con base en los instrumentos disponibles para su medición. Como un marco general, los resultados señalan una reducción de las prevalencias de consumo de productos de tabaco (cigarrillos).

2.2.1 Prevalencia nacional y por zona geográfica.

El análisis de las prevalencias se constituye en otro de los elementos más importantes en la medición del consumo de cigarrillos. En este caso se utilizaran los instrumentos de medición más recientes, comenzando por la Encuesta Mundial de Tabaco en Jóvenes[6], entre 13 y 15 años. De acuerdo a esta encuesta, para el año 2002 las prevalencias en general eran de 32.2%, mientras que en el año 2008, eran de 17.1%, reflejando una reducción del 15.1%. En los varones los porcentajes de 36.2% y 27.1%, mientras que en la mujeres, 27.7% y 13.0%, respectivamente.

Otro instrumento de medición es la Encuesta Nacional de Salud y Calidad de Vida[7] (ENSCAVI-2007), también de cobertura nacional. Con dicha encuesta se obtuvieron las prevalencias a 30 días del consumo de tabaco en el ámbito nacional y por los estratos urbano, rural e indígena, para la población de 18 años. Los resultados se presentan en la siguiente tabla.

[6] Para el año 2002, la muestra fue de 2017 jóvenes y en 2008, de 3,000.

[7] El tamaño de la muestra fue de 25748 individuos.

Tabla N° 4
Población de 18 años y más por prevalencia de consumo de tabaco en los últimos 30 días, por área y sexo en porcentajes.

Área	Total	Hombres	Mujeres
Urbana	9.0	17.1	4.2
Rural	8.6	15.9	2.9
Indígena	13.5	24.8	5.0
Nacional	9.4	17.7	3.9

Fuente: Encuesta Nacional de Salud y Calidad de Vida (ENSCAVI). Año 2007.

La prevalencia nacional es del 9.4%, pero la rural es la más baja de las tres áreas con el 8.6%, mientras que la indígena presenta la más alta con el 13.5%. Es dable señalar que en el caso de las zonas indígenas la situación del tabaquismo es mucho acentuada, por las prevalencias observadas y en donde por la situación de su aislamiento relativo de los centros urbanos, resultan menos efectivas las medidas de control de espacios libres de humo e incluso las de tipo fiscal, donde hay mucho más riesgo de contrabando.

2.2.2 Prevalencias por sexo y edad.

Las prevalencias de los hombres son más altas en todos los estratos, de manera muy significativa en relación a las mujeres, según la ENSCAVI. En el ámbito nacional, la prevalencia de los hombres fue de 17.7% y en las mujeres de 3.9%. Sin embargo, es el área indígena donde se observa la mayor prevalencia respecto de los hombres con el 24.8% y en las mujeres, el 5.0%. Mientras que en el área rural, es donde se observan las menores prevalencias por sexo, con 15.9% y 2.9% respectivamente.

No obstante, tomando como referencia los datos más recientes obtenidos a partir de la Encuesta de Prevalencias de Factores de Riesgo de Enfermedad Cardiovascular (PREFREC-2010), la cual solo se hizo para las provincias de Panamá y Colón, refleja una disminución si se comparan dichas provincias con relación a la ENSCAVI.

Tabla N° 5

Resultados comparativos de las prevalencias (a 30 días) de tabaquismo calculadas para las provincias de Panamá y Colón según la ENSCAVI y PREFREC

Encuesta	Sexo	Panamá	Colón
ENSCAVI	Hombres	17.1	12.8
	Mujeres	4.4	2.4
	Total provincial	9.2	6.5
PREFREC	Hombres	11.9	16.5
	Mujeres	3.2	2.1
	Total provincial	5.9	6.0

Fuente: ENSCAVI (2007) y PREFREC (2010).

En ambas provincias se observan reducciones significativas, pasando las prevalencias en la provincia de Panamá de 9.2% a 5.9%, y en Colón de 6.5% a 6%, según los resultados de la ENSCAVI y de PREFREC en ese mismo orden. Empero, con relación a las prevalencias de los hombres se observa en la provincia de Colón un incremento al pasar de 12.8% a 16.5%, mientras que con relación a las mujeres, las mismas han descendido.

Esta claro que la relativa reducción de las prevalencias provinciales, se corresponden con las menores prevalencias de las mujeres, mientras que las de los hombres se mantienen significativamente altas. No obstante, al ser un dato puntual (de dos años previos) solo de dos momentos de medición, las prevalencias no dejan en claro su posible asociación con las medidas fiscales, especialmente respecto del ISC a partir de su entrada efectiva en el año 2010. En atención a la evolución del contrabando y la venta ilegal que será abordado más adelante.

2.3 Medidas de control del consumo de productos derivados del tabaco.

Es posible que junto a las medidas fiscales, las medidas de control en materia de publicidad y espacios libres de humo, hayan contribuido a la reducción de las prevalencias. A continuación se hace una relación cronológica de las medidas de control de consumo de productos del tabaco.

En Panamá las medidas de control para el consumo de tabaco inicialmente se concentraron en prohibir la venta de derivados del tabaco a los menores de edad, prohibir fumar en oficinas

públicas y lugares cerrados tales como cines, transporte público y selectivo, teatros, escuelas e instalaciones de salud entre otras. La publicidad estaba dirigida a desincentivar a los jóvenes en el inicio del consumo de productos de tabaco, sin embargo, éstas han sido reforzadas con legislaciones recientes de finales del periodo investigado.

Mediante el Decreto Ejecutivo N° 86 de 27 de mayo de 1999 se reglamentan las restricciones y disposiciones sobre la venta de cigarrillos y tabaco a los menores de edad y la impresión de advertencias sobre su uso. Se establece la rotulación obligatoria en la cajetilla de cigarrillos y envoltorios no externos de tabaco, que sean puestos a la venta en el territorio nacional, con las siguientes advertencias: "Prohibida la venta a menores de edad" y "Fumar es nocivo para La salud".

Con la ratificación del Convenio Marco de la Organización Mundial de la Salud (OMS), mediante la Ley 40 del 16 de agosto 2004, se promulga el Decreto Ejecutivo N° 17 del 11 de marzo de 2005, en el que se introducen medidas tales como: prohibir fumar en restaurantes, las advertencias sanitarias y pictogramas que deben ocupar el 50% en los paquetes de productos del tabaco, se prohibió la venta de cigarrillos sueltos y en paquetes de menos de 20 unidades, la prohibición del uso de máquinas expendedoras de cigarrillos, se prohíbe la publicidad en vallas y en medios de comunicación masivos, se circunscribe la publicidad únicamente en los puntos de venta y la promoción y el patrocinio solo a actividades de participación de la población adulta (mayores de 18 años).

El 11 enero de 2008 se promulgó la Ley Nº 13, mediante la cual se adoptan medidas para el control del tabaco y sus efectos nocivos en la salud, la cual fue reglamentada mediante el Decreto Ejecutivo Nº 230 del 6 de mayo de 2008, y establece restricciones adicionales, en refuerzo de las medidas aprobadas y adoptadas en el año 2005, tales como: intensificar las medidas de control de tabaco mediante la prohibición del uso de términos que sugieran que un producto de tabaco es menos dañino que otro en todos los paquetes de productos de tabaco; se prohíbe totalmente cualquier forma de publicidad, promoción y patrocinio de los productos de tabaco, incluida la transfronteriza; se prohíbe el consumo de tabaco en todos los ambientes laborales cerrados, áreas comunes de edificios públicos y privados e incluso en aquellos espacios abiertos dedicados a las actividades deportivas; se fortalece la participación de la sociedad civil en el control del tabaco; se obliga al Estado a poner al acceso del público programas de promoción y abandono del consumo de tabaco, a informar sobre las consecuencias sanitarias, la naturaleza adictiva y la amenaza mortal del consumo de tabaco y de la exposición al humo de tabaco y a integrar en los planes de educación general básica y de la educación media contenidos sobre control de tabaco. En la misma también quedan tipificadas las violaciones y sanciones a la ley y a su reglamento.

Es de esperarse que estas medidas de control, que refuerzan las de 2005, hayan tenido un efecto directo e indirecto en reducir el consumo de tabaco. Con este conjunto de medidas de control del consumo de tabaco, Panamá se puso a la vanguardia en el control de la epidemia del tabaquismo, con reformas de última generación mediante las cuales el país se ha puesto al día en la política de

control del tabaco, adoptando los estándares internacionales y recomendados por los organismos internacionales.

Las medidas de control del consumo del tabaco han tenido un fuerte alcance en el campo laboral y en el entorno cotidiano en espacios cerrados y abiertos, especialmente comerciales. El apoyo del sector privado y grupos de presión antitabaco, ha involucrado a los medios de comunicación de manera amplia, en adición a las campañas informativas y restricciones en la publicidad del tabaco.

Las medidas de control del consumo de tabaco que prohíben fumar en espacios cerrados van ligadas a medidas sancionadoras, lo que induce el cumplimiento de las normas vigentes. No obstante, en el plano familiar la efectividad de estas legislaciones tienen su escollo al no poder controlar el hábito de fumar en las residencias, dado que es un entorno privado dónde las personas tienen pleno derecho de hacerlo. Este es el campo para las campañas de concientización y en el cual aún quedan estrategias más efectivas por desarrollar, respecto de los fumadores pasivos expuestos al humo de segunda y de tercera mano.

III. Comercio de productos del tabaco.

Como se ha evidenciado, Panamá, es un país importador neto de productos derivados del tabaco, especialmente con relación a los cigarrillos, como el principal producto de consumo. Sin embargo, el análisis de las estadísticas de importación y de las incautaciones, permiten matizar la situación del mercado interno, al observar la evolución histórica de estas variables.

3.1 Evolución de las importaciones de productos del tabaco.

Al observar los datos de las importaciones de los productos derivados del tabaco, se evidencia que los cigarrillos representan casi la totalidad de las mismas, dejando otros productos como por ejemplo, los cigarros, con una participación marginal. De igual manera la evolución de las importaciones de cigarrillos, mantienen una caída vertiginosa a partir del año 2004, año en que se adopta el Convenio Marco.

Dicha caída fue del -4.6% promedio trimestral entre el año 2004 y 2011 e incluso se hace mucho más pronunciada a partir del año 2010, cuando se aplica de manera efectiva el incremento del ISC en 100%.

Hasta este punto no queda duda de que la caída de las importaciones, guarda correspondencia con la aplicación de las medidas de control de espacios libres de humo, en adición a las medidas fiscales. De igual manera dado que las prevalencias han bajado en términos relativos, no queda en claro que tan importante puede ser el consumo ilegal, especialmente el contrabando, dado que esta actividad no paga impuestos por lo que es muy difícil de medir.

Es por ello que se incluye el análisis de la encuesta de marcas al igual que la de locales, con relación a las percepciones de los dueños de bares y restaurantes, entre otros tipos de locales, respecto de las medidas de control, siendo en este caso un instrumento de recolección y medición mucho más efectivo que las estadísticas formales y que complementarán este análisis. La única desventaja es que ambas encuestas solo darán una perspectiva muy puntual de la situación en un período de corte transversal. Su inclusión en los análisis posteriores será de gran valía, máxime por que este tipo de instrumentos no han sido empleados en la actualidad.

Gráfica N° 3

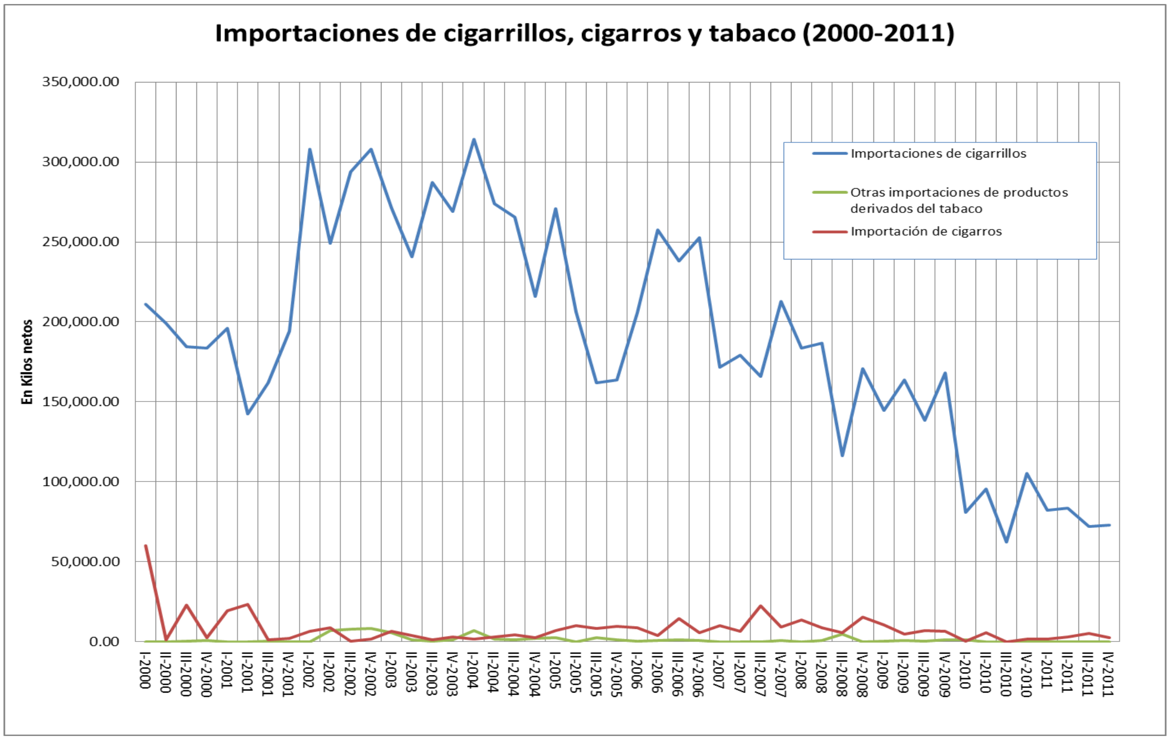

Fuente: Elaboración propia con datos de la Contraloría General de la República.

Por otro lado y como se verá en la sección siguiente, la Economía del tabaquismo también viste ribetes de Economía Política, en el sentido de que precisamente a partir de inicios de la década del 2000, la industria tabacalera centroamericana se re estructura, dejando a Panamá, como un país importador, situación que tendrá implicaciones importantes respecto a la efectividad de la última subida del ISC, al provenir las importaciones de países centroamericanos.

3.2 Origen de las importaciones de productos del tabaco.

En general las importaciones de cigarrillos y otros de los productos del tabaco de mayor consumo, provienen de países de las américas, por ser el mercado natural del país. Tal como se observa en la tabla siguiente, Nicaragua, Honduras, Estados Unidos y Guatemala, se constituyen en los países más consistentes durante el período de estudio.

Con relación a las importaciones provenientes de la Zona Libre de Colón, su origen es diverso e incluye en principio algunos países del caribe no identificados, en esta agrupación. Sin embargo, dicha zona es donde se dan la mayoría de las incautaciones en tránsito por el país.

Tabla N° 6

Importaciones generales de productos del tabaco según países de origen

Descripción	Años		
	2000	2005	2010
CIGARROS (PUROS) (INCLUSO DESPUNTADOS), CIGARRITOS (PURITOS), Y CIGARRILLOS, DE TABACO O DE SUCEDANEOS DEL TABACO.	COSTA RICA	ESTADOS UNIDOS DE AMERICA	COLOMBIA
	CUBA	HONDURAS	ESTADOS UNIDOS DE AMERICA
	ESTADOS UNIDOS DE AMERICA	NICARAGUA	NICARAGUA
	GUATEMALA	PAISES BAJOS	CHILE
	NICARAGUA	VENEZUELA	GUATEMALA
	SUIZA	ZONA LIBRE DE COLON	HONDURAS
	ZONA LIBRE DE COLON	CHILE	MEXICO
	HONDURAS	GUATEMALA	ZONA LIBRE DE COLON
	VENEZUELA	CUBA	BULGARIA
	ESPAÑA	ESPAÑA	
		PERU	

Fuente: Elaboración propia con datos de la Contraloría General de la República.

Esta composición del origen de las importaciones, deja en claro la estrategia de las empresas tabacaleras de utilizar a Panamá, como un país importador y otros países centroamericanos como productores, en especial Honduras y Guatemala. Es importante poder profundizar en esta materia que atañe en parte a la Economía Política del Tabaco, por el hecho de que al provenir importaciones de algunos países, exoneradas del pago del arancel de importación deja como resultado una aplicación parcial del ISC, y que tampoco se descartan problemas de sub-declaración o evasión fiscal en general en adición a que las estrategias comerciales de las tabacaleras son de carácter regional y transcienden a las medidas locales en materia aduanera y tributaria.

3.3 Restricciones al comercio.

La venta de tabaco en Panamá es legal, pero está restringida al cumplimiento de las normas establecidas, especialmente a partir de la aprobación del Convenio Marco, en materia de advertencias sanitarias con pictogramas que deben cubrir el 50% de la cajetilla, por ambas caras. De igual manera es de estricto cumplimiento las medidas de espacios libres de humo, con el fin de mitigar los efectos del humo de segunda y de tercera mano. En materia de publicidad también existen restricciones fuertes, al punto de que la publicidad esta prohibida en el país, en todo tipo de actividades, incluso los productos derivados del tabaco, no pueden estar expuestos a la vista del público en ningún tipo de comercio o local.

Por otro lado las autoridades sanitarias realizan operativos cotidianos para la pesquisa de productos del tabaco de venta ilegal, es decir, contrabando, productos falsificados, venta de cigarrillos sueltos, venta a menores, entre otros. Empero la medición del comercio ilegal ha sido difícil y es uno de los elementos que causa mayor incertidumbre respecto de la reducción del consumo vía importaciones al igual que con relación a las prevalencias analizadas.

3.4 Situación del contrabando de productos del tabaco.

Actualmente resulta difícil tener una estimación del volumen de contrabando en el mercado local, lo cual deja cierto velo de incertidumbre con relación al consumo real.

Gráfica N° 4

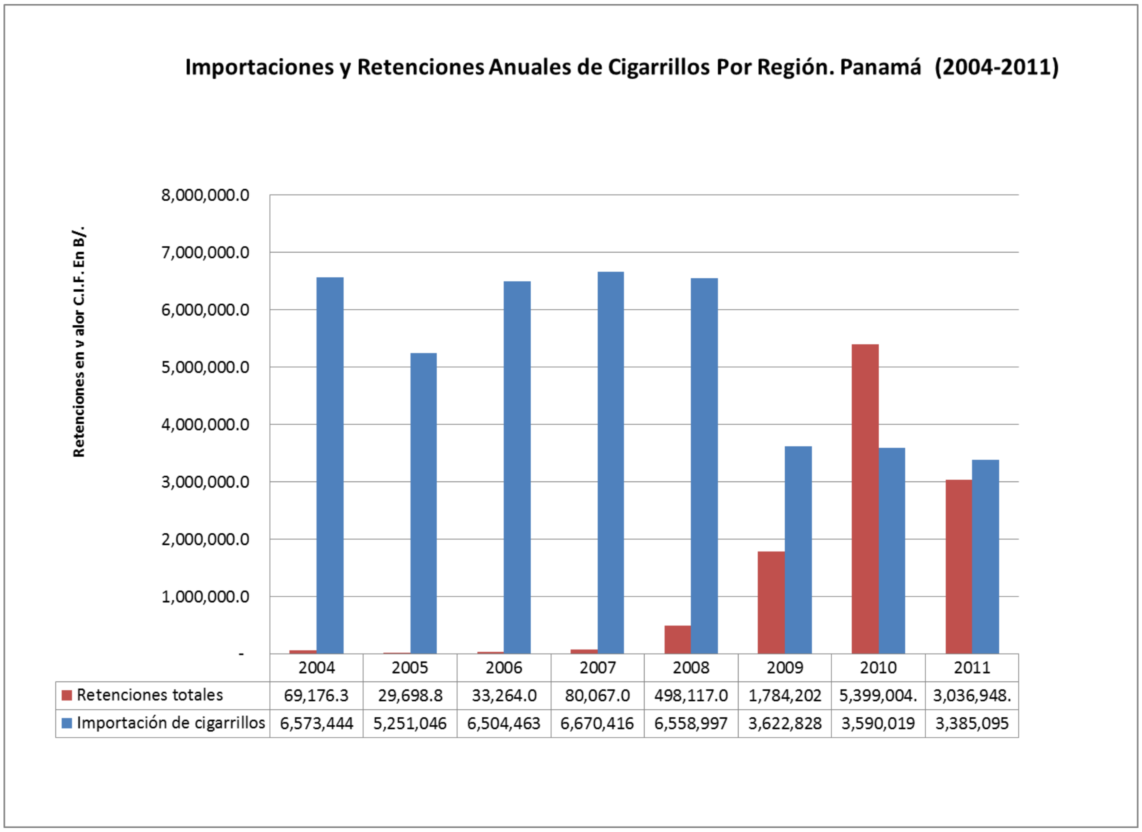

Fuente: Elaboración propia con datos de la Contraloría General de la República.

La gráfica anterior muestra como se incrementaron las incautaciones o retenciones en las zonas libres del país, especialmente en la Zona Libre de Colón. No obstante, las incautaciones poco aportan como variable informativa, respecto del contrabando en el mercado local, dado que dichas retenciones no dejan una clara idea de su destino o mercado final. Es decir, si se trata de incautaciones en tránsito o si tenían como destino el mercado local panameño.

Sin embargo, pueden ser una señal indirecta del posible efecto de las medidas fiscales sobre el comercio ilícito o bien de la mayor eficacia de la Autoridad Nacional de Aduanas en su pesquisa de productos ilegales a partir del año 2010, máxime cuando de acuerdo al último incremento del ISC, se le asigna a esta autoridad el 20% del 50% de lo recaudado.

De lo anterior se deslinda que solo mediante la aplicación de un instrumento de recolección específico como una encuesta, podrá dilucidarse que tanto puede pesar el contrabando o la venta ilegal en general, con base en la encuesta de marcas. La declaración del precio, lugar de compra e identificación del empaque, se constituyen en variables fundamentales para rastrear el contrabando y tener una idea general de su incidencia en el mercado de tabaco de Panamá, entre otras variables de referencia.

IV. Evolución de los precios, impuestos, ingresos y elasticidades precio e ingreso de la demanda.

Para tener una perspectiva de la evolución de los precios de los cigarrillos, se incorpora la representación gráfica de la relación entre el índice de precios al consumidor de tabaco (que en este caso es sobre la base de las marcas más consumidas de cigarrillos) y el índice de precios al consumidor total.

A partir del primer trimestre del año 2010, se observa un incremento significativo del IPC de tabaco, coincidiendo con la entrada efectiva del incremento del ISC. Durante todo el período la tasa de crecimiento histórica fue de 2.6%, sin embargo, entre el cuarto trimestre de 2009 y el cuarto trimestre de 2011, el crecimiento promedio trimestral fue de 11.8%, indicando un cambio de nivel significativo respecto a la evolución del IPC total, el cual se mantuvo más estable, con un promedio trimestral de 0.2% y entre el cuarto trimestre de 2009 y cuarto trimestre de 2011, fue de 1.8% en promedio.

4.1 Evolución de los precios reales de los cigarrillos.

Gráfica N° 5

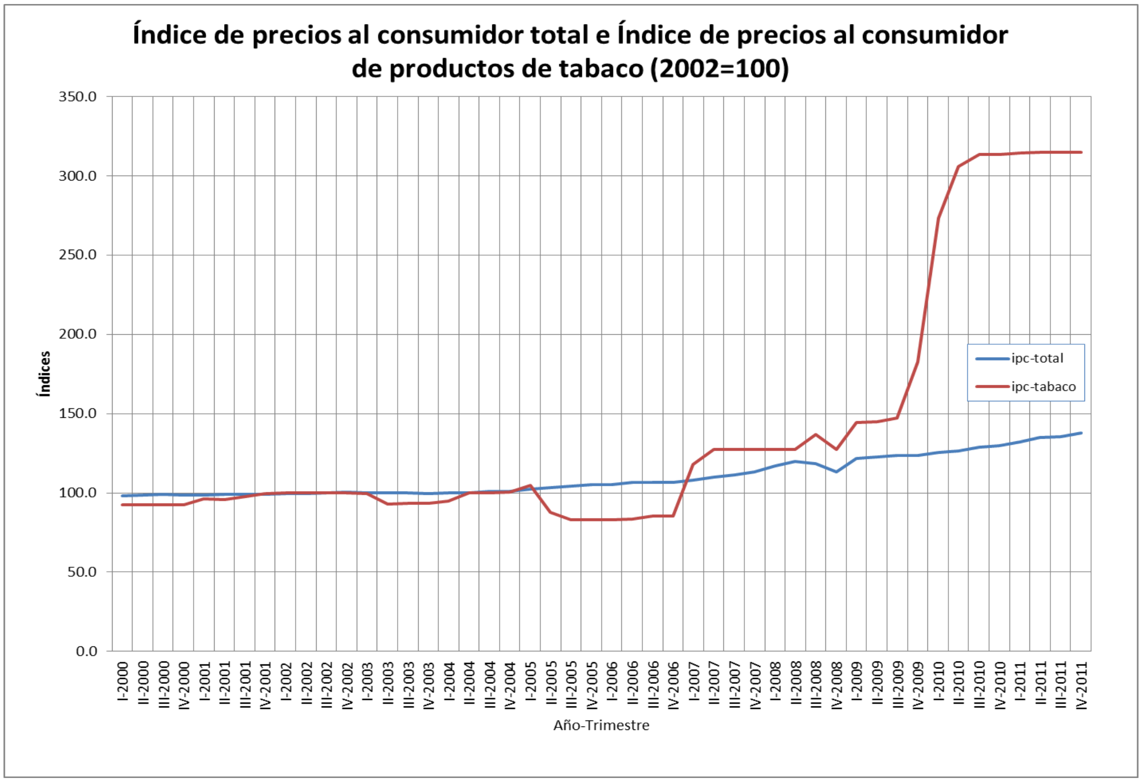

Fuente: Elaboración propia con datos de la Contraloría General de la República.

Por otro lado, entre el primer trimestre de 2005 y el cuarto trimestre de 2006, se observa una reducción del IPC de tabaco, por debajo del IPC total, con una caída en promedio de -2.9%, coincidiendo con la aplicación de las medidas de control a partir del año 2005 mediante el Decreto Ejecutivo N° 17 del 11 de marzo de ese año, con el cual se introducen medidas tales como: prohibir fumar en restaurantes, las **advertencias sanitarias y pictogramas** que deben ocupar el 50% en los paquetes de productos del tabaco, se prohibió la venta de **cigarrillos sueltos y en paquetes de menos de 20 unidades**, la prohibición del uso de **máquinas expendedoras** de cigarrillos, se prohíbe **la publicidad en vallas** y en medios de comunicación masivos, se circunscribe la **publicidad únicamente en los puntos de venta** y la promoción y el patrocinio solo a actividades de participación de la población adulta (mayores de 18 años).

Un argumento esbozado a este respecto tanto de la industria como de la entidades tributarias, fue la existencia de una mayor competencia, como respuesta a dichas medidas de control, pero que posteriormente se da un mayor incremento del IPC de tabaco respecto del IPC total, especialmente a partir del año 2009. Dicho comportamiento, es recogido como cabría esperar respecto del precio real (relación entre el IPC de tabaco e IPC total).

Gráfica N° 6

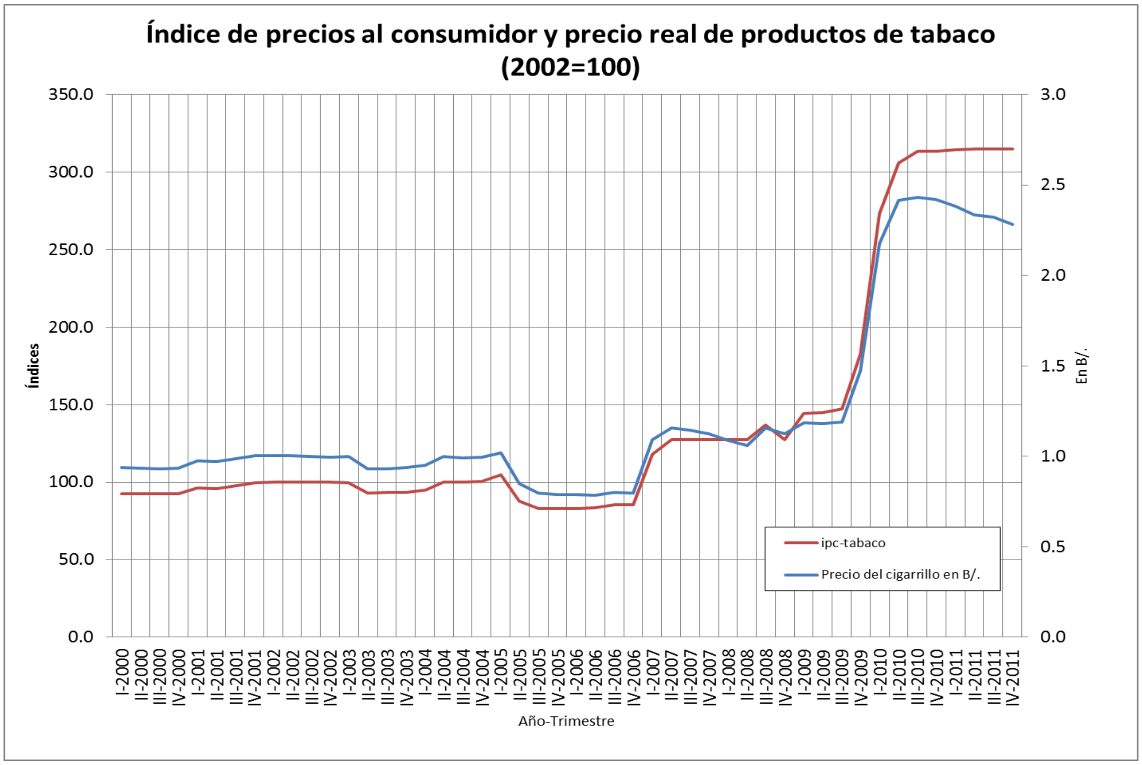

Nota: El precio del cigarrillo es el equivalente al precio real de tabaco, dado que la encuesta de precios en este caso solo recoge el precio de las tres principales marcas de cigarrillos.
Fuente: Elaboración propia con datos de la Contraloría General de la República.

Con todo, lo más importante es el cambio estructural observado a parir del primer trimestre de 2010, el cual refleja un cambio de tendencia permanente y que es coincidente con el incremento del ISC al 100%. Es clara la efectividad de la política fiscal en este caso sobre el precio al consumidor y el efecto regresivo esperado de los impuestos que gravan al consumo, que con relación al consumo de tabaco es fundamental para combatir su externalidad negativa.

4.2 Participación del gasto en cigarrillos respecto del gasto total.

Panamá es un país que se caracteriza por tener una baja participación del gasto en productos del tabaco (cigarrillos) en la estructura de consumo general, de acuerdo al Índice de Precios al Consumidor en los Distritos de Panamá y San Miguelito (que es el que históricamente se ha empleado para medir la inflación en Panamá), de acuerdo al año base actual utilizado (2002 = 100), con un resultado de **0.2%** en la gran división del índice. Mientras que con la base de 1987, era de **0.8%**, indicando que el mismo ha venido experimentando un descenso significativo.

Ello unido a una prevalencia de 9.4%, dejan una clara idea de que en la estructura del consumo de los hogares, el consumo de tabaco ha venido perdiendo importancia relativa. Es precisamente a partir del año 2002 cuando se observa una caída de las importaciones de cigarrillos en Panamá, incluso mucho antes de la ratificación del Convenio Marco y de las medidas de control y fiscales aplicadas, con posterioridad.

Este elemento es de relevancia toda vez que contribuye a explicar en parte el comportamiento observado en el consumo, en adición de incorporar un argumento a contrastar respecto del contrabando o la venta ilegal en Panamá.

4.3 Evolución de la recaudación del Impuesto Selectivo al Consumo de Cigarrillos y otros productos derivados del tabaco (ISC).

El comportamiento de las ventas (derivadas de la base impositiva) y la recaudación, presentan un comportamiento similar en la mayor parte del período, hasta finales del año 2009. Pese a las fuertes fluctuaciones observadas con cambios de tendencias marcadas, la tendencia de largo plazo ha sido decreciente en el caso de las ventas. De igual forma queda reflejada la ruptura marcada a partir del primer trimestre de 2010, cuando se observa un cambio de nivel entre ambas variables, con una caída en las ventas por debajo de los seis millones de Balboas trimestrales y la recaudación alcanza precisamente esa cantidad como reflejo del incremento impositivo al 100%.

Gráfica N° 7

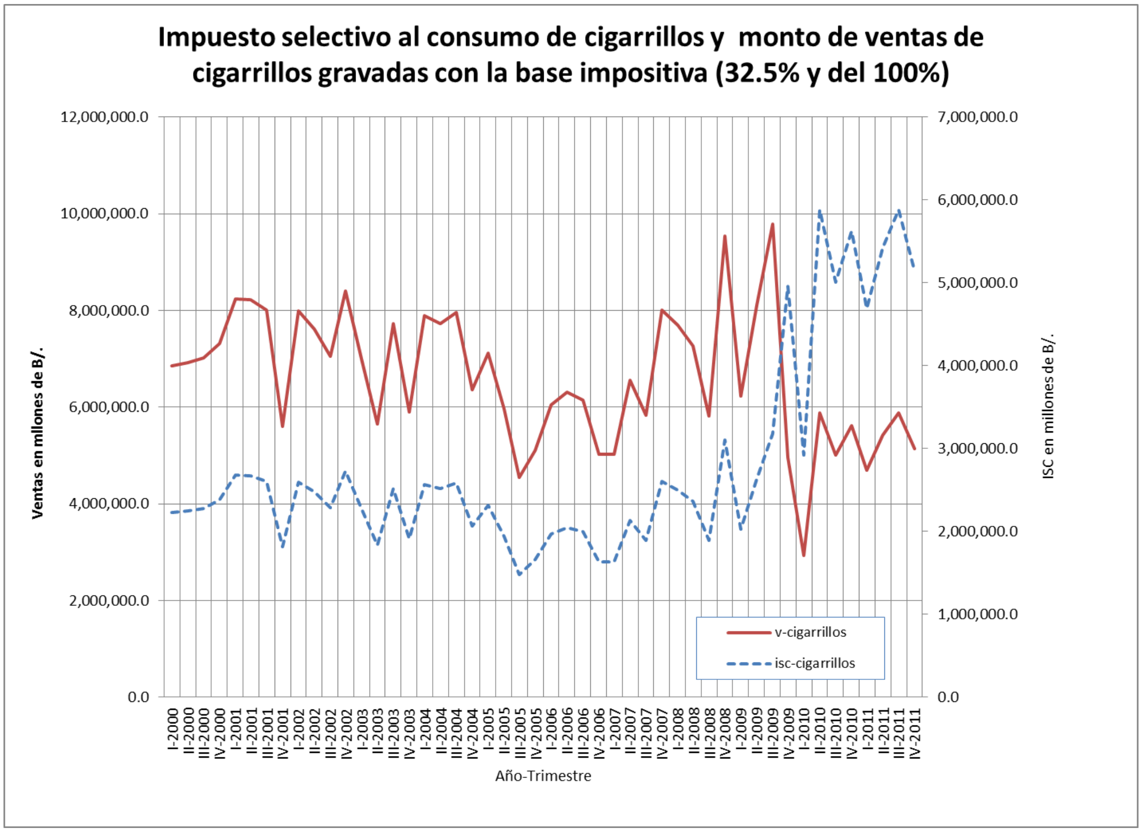

Fuente: Elaboración propia con datos de la Contraloría General de la República.

De igual manera el incremento en la presión tributaria con relación a la participación del ISC respecto del Producto Interno Bruto, refleja un nuevo umbral de aumento desde el tercer trimestre del año 2009. Entre el primer trimestre del año 2000 y el segundo trimestre del año 2009, la presión tributaria respecto del ISC venía con una tendencia decreciente. Hasta el segundo trimestre del 2005, la misma estaba entre el 0.6% y 0.10%. Hasta el tercer trimestre de 2009, la misma se mantuvo en general por debajo del 6%. Pero a partir del tercer trimestre de 2009, se ha mantenido entre el 0.8% y 0.12% respectivamente como respuesta al incremento del 100%.

Ello deja en claro el impacto de la política fiscal y que de igual manera la misma no tiene un efecto permanente de no seguir realizándose nuevos incrementos impositivos. Como se observa con la antigua base impositiva del 32.5%, la presión tributaria venía reduciéndose llegando a su nivel más bajo precisamente en el segundo trimestre de 2009. Ello es de vital importancia respecto del control del efecto asequibilidad que a mediano y largo plazo deja sin efecto las medidas fiscales, ante el incremento de la renta por habitante.

Gráfica N° 8

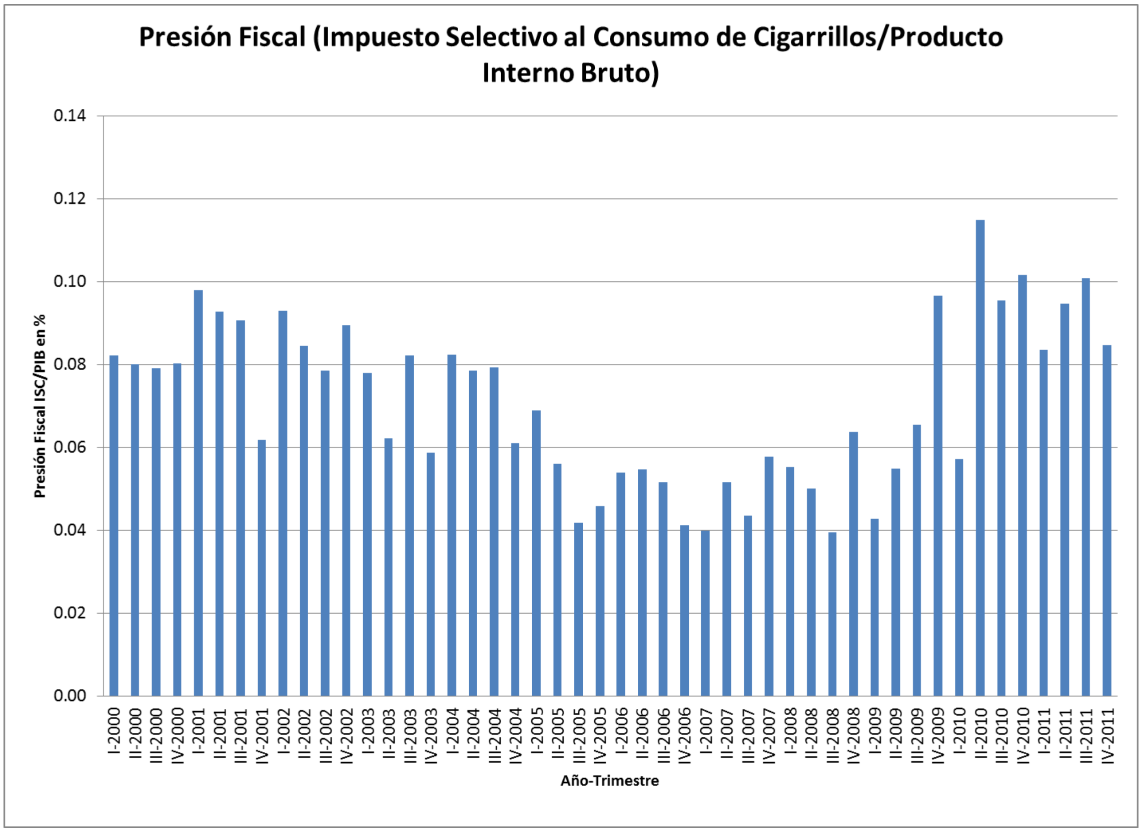

Fuente: Elaboración propia con datos de la Contraloría General de la República.

Entre 2005 y 2008, la presión tributaria tuvo sus menores niveles por debajo del 0.6% y sobre todo entre 2005 y 2006, cuando los precios reales alcanzaron sus menores niveles.

En la gráfica siguiente, se muestra la evolución del Producto Interno Bruto por habitante, el Impuesto Selectivo al Consumo de Cigarrillos y otros productos del tabaco y el I.T.B.M.S (I.V.A.) de importación. Los impuestos han sido transformados a índices simples para observar mejor su evolución sobre un año base común.

Se puede observar que mientras el Producto Interno Bruto por habitante, crece como cabría esperar en el caso de Panamá, el I.V.A. de importación, se incrementó por encima del ISC, entre enero y noviembre de 2009, es decir con la base de 32.5%, hasta la aplicación del incremento del ISC al 100%. Incluso en septiembre de 2009, hubo un incremento transitorio del ISC al 50%. Posteriormente entre noviembre de 2009 y octubre de 2010, se observa un cambio en la recaudación fiscal, en la cual el ISC pasa a estar por arriba del I.V.A. de importación. Luego de noviembre de 2010 en adelante, el I.V.A., de importación supera nuevamente al ISC, tal como ocurría con la vieja base impositiva del 32.5%, antes del incremento del 100% en el cuarto trimestre de 2009.

Gráfica N° 9

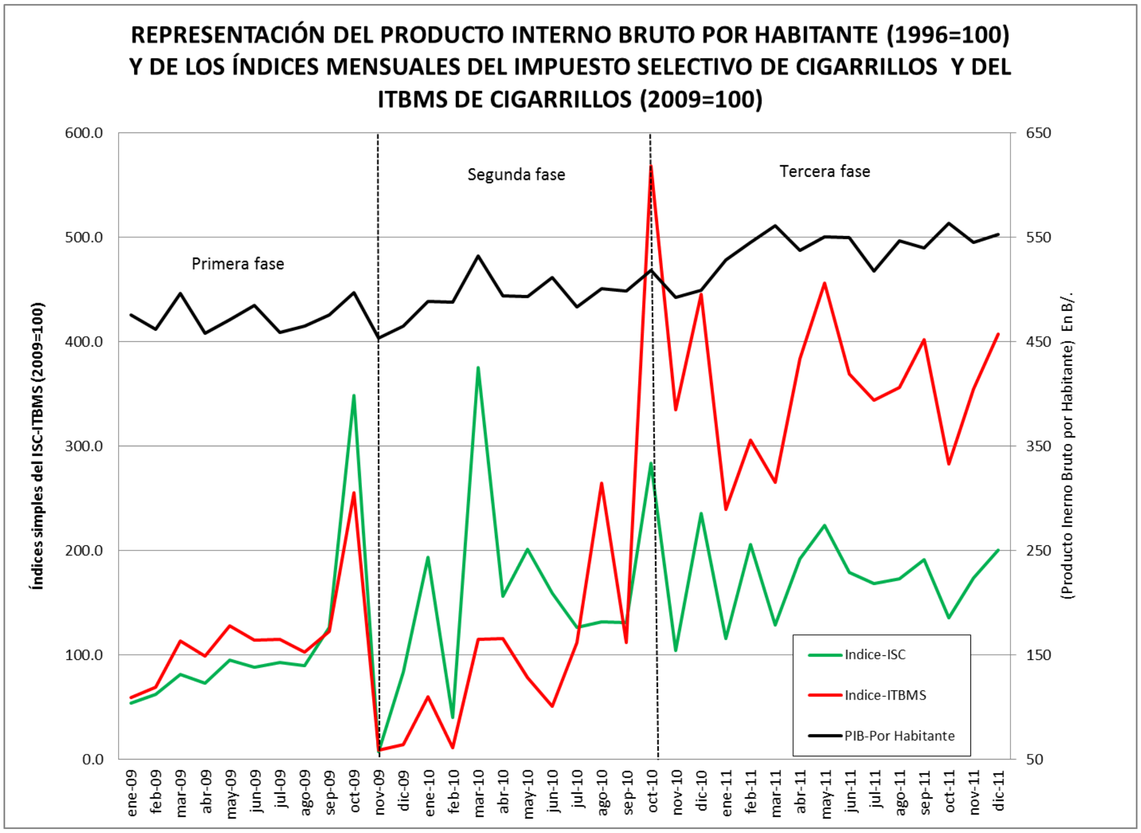

Fuente: Elaboración propia con datos de la Contraloría General de la República.

El índice simple del ISC refleja variaciones amortiguadas a partir de noviembre de 2010, mientras que el I.V.A., de importación parece ser el impuesto más beneficiado con relación a la recaudación fiscal, respecto del comportamiento de las importaciones. Ello deja una clara relación del comportamiento de las ventas derivadas de la base impositiva, pudiendo dejar algún indicio de cambio estructural en el consumo.

Es evidente que las medidas de incremento impositivo han dejado su efecto sobre el consumo, tal como se ha observado en el comportamiento de las importaciones. No obstante, respecto de las medidas de control de espacios libres de humo, publicidad, entre otras, deja en claro que desde 2002 y sobre todo a partir de 2004, se viene dando un comportamiento decreciente sostenido de las importaciones, incluso antes de la ratificación del Convenio Marco, que en principio pudiera ser la expresión de una problemática más compleja y que pareciera que existen otros factores que han incidido en adición de las medidas mencionadas. Pese a que el ISC se incremento entre 2009 y 2011, la recaudación parece haberse estabilizado en un umbral que no supera los B/. 6,000,000.00 trimestrales, después del primer trimestre de 2010 y de hecho el índice simple,

muestra una situación similar al igual que el comportamiento de las importaciones a partir de ese mismo punto temporal, con una ligera tendencia decreciente en el precio real.

4.4 Elasticidades precio e ingreso de demanda y simulación del impacto fiscal del aumento del ISC.

De lo anterior se puede mencionar que la dinámica de los precios reales y de las importaciones, guardan una estrecha relación con lo observado respecto de la recaudación tributaria del I.V.A. y de l ISC, especialmente a partir del primer trimestre de 2010. Al observar las series del precio real y de las importaciones, es evidente el cambio en la elasticidad arco de la demanda en los cuatro períodos establecidos.

Gráfica N° 10

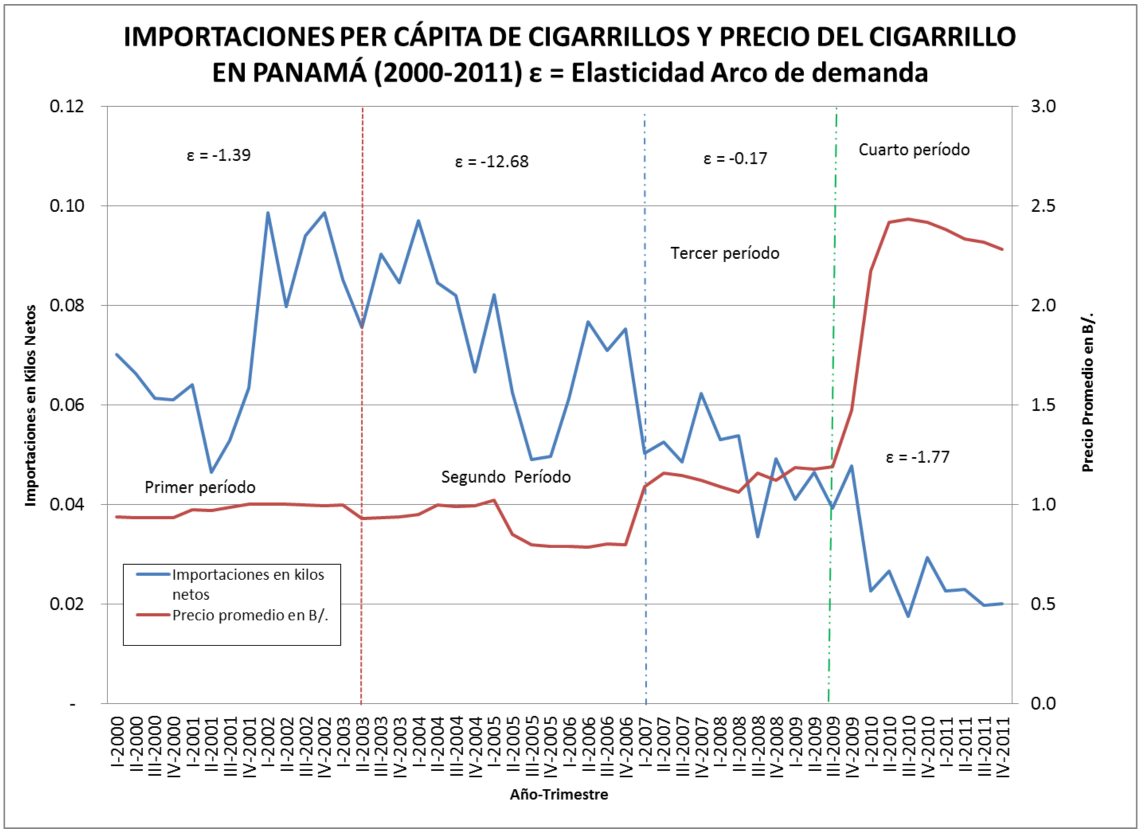

Fuente: Elaboración propia con datos de la Contraloría General de la República.

El primer período abarca desde el primer trimestre de 2000, hasta el segundo trimestre de 2003, en el cual las importaciones tienen una tendencia creciente, mientras que el precio muestra una tendencia ligeramente creciente y una evolución más estable. Es este el período en que recién la industria productora de cigarrillos se desmantela y se reconvierte a importadora. No obstante, es evidente que ante las fuertes variaciones en las importaciones, el coeficiente de elasticidad arco de la demanda (tomando como base las importaciones) sea mayor que uno.

Dicho coeficiente resulta más elevado en el segundo período, entre el segundo trimestre de 2003 y primer trimestre de 2007, con un valor de -12.68 en adición a que en este período se adopta el Convenio Marco en 2004 y se establecen fuertes medidas de control, en materia de publicidad, espacios libres de humo, entre otras, que dejan evidencia de su impacto en las importaciones y en el precio, entre el primer trimestre de 2005 y cuarto trimestre de 2006, cuando el precio real tiende a reducirse. Según entrevista realizada a funcionarios del departamento que elabora el índice de precios al consumidor, manifestaron que la respuesta de las tabacaleras fue una fuerte competencia en precios.

A partir del primer trimestre de 2007, hasta el tercer trimestre de 2009, el precio real tiende a tener una tendencia mucho más creciente que en los trimestres anteriores, mientras que las importaciones continúan con su caída sostenida y mucho más pronunciada. La elasticidad en dicho período tuvo un valor de -0.17, el más bajo del período, indicando que en el mismo la demanda se torno relativamente más inelástica. Este fue el período previo al incremento del 100% del ISC y que a partir del tercer trimestre de 2009, se incrementa de manera significativa el precio real con una reducción marcada de las importaciones (demanda), aunque más amortiguada a partir del primer trimestre de 2011, al igual que se ha observado con el ISC, para el mismo período.

Vale destacar que a partir del cuarto trimestre de 2010 el precio real del cigarrillo comienza a decrecer, siendo la elasticidad de -1.71, reflejando una mayor sensibilidad de la demanda ante el aumento del precio. Este análisis revela que la elasticidad es variable en el tiempo y por ende el grado de respuesta de la misma ante variaciones en el precio. El cuarto período representa el incremento más elevado del precio real, respecto de toda la serie histórica, dejando evidenciando el impacto efectivo de la política fiscal anti tabáquica, ante el incremento sostenido de la renta nacional per cápita, como se observa en la gráfica siguiente.

Mientras la demanda (importaciones), muestra una tendencia decreciente ante los incrementos en el precio, mantiene por el contrario una relación inversa con la renta por habitante. Lo anterior, no deja de tener una connotación controversial, respecto del comportamiento esperado cuando se incrementan los impuestos al consumo de tabaco. Es decir, que se esperaría que el aumento de la recaudación, se acompañe de un incremento en el consumo ante un incremento de la renta por habitante, ante el hecho de que la elasticidad precio de la demanda de largo plazo debe ser menor que uno, como cabría esperar, no obstante, el incremento de la elasticidad arco

del período de aplicación del incremento del ISC deja en perspectiva, que posiblemente la elasticidad de largo plazo, tienda a ser relativamente más alta de lo esperado.

Gráfica N° 11

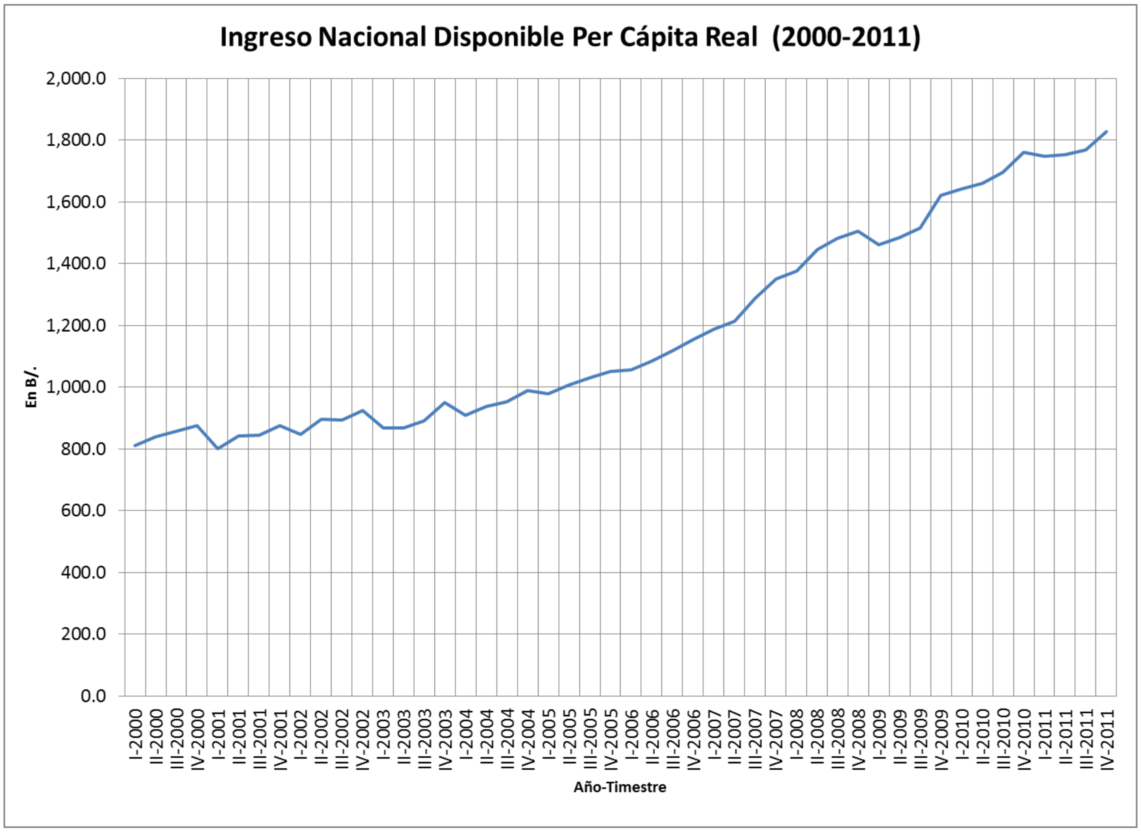

Fuente: Elaboración propia con datos de la Contraloría General de la República.

Los resultados reflejan que el impacto del ISC en el precio ha sido muy visible, aunque no deja lugar para descartar que pese a ese efecto, existe un incremento en la asequibilidad del consumo de tabaco, a muy corto plazo, por lo que el efecto de la política fiscal es temporal y se requiere en consecuencia de incrementos sucesivos del ISC para combatir dicho efecto. Incluso, tal como se observo, el precio real tendió a reducirse ligeramente a partir de 2011, de manera mucho más marcada.

4.4.1. Análisis de la demanda y variables explicativas.

-La demanda de productos de tabaco.

Como se ha mencionado, la demanda de cigarrillos es satisfecha solo por las importaciones, dado que Panamá desde hace más de una década no tiene industria que produzca este producto, al igual que con relación a otros productos derivados del tabaco.

-Precio del cigarrillo.

Es una de las variables fundamentales para estimar la demanda. El índice de precios al consumidor del tabaco no es un ponderado de marcas, sino que en cada establecimiento se toma la marca que más se vende y se releva el precio de la misma.

-Ingreso real disponible Per Cápita.

Es la segunda variable del modelo de demanda con una importancia explicativa determinante del consumo. Es necesaria su inclusión y analizar su interacción con el precio, respecto de los modelos objeto de este estudio, toda vez que es importante estimar el efecto de la renta sobre el consumo.

Como se señaló en los comentarios anteriores, pese a que el incremento en el ISC ha dejado un marcado incremento en el precio, el incremento en la renta por habitante, no deja de tener su efecto a corto plazo con relación a la asequibilidad. En el gráfico siguiente, se muestra que pese al incremento en el ISC la asequibilidad aumentó posteriormente, tomando como base el indicador RIP[8] (Relative Income Price), es decir, el porcentaje de la renta por habitante que se requiere para comprar 100 paquetes de cigarrillos, refleja que a partir del cuarto trimestre de 2010, la medida fiscal comienza a perder efecto.

[8] Este indicador se interpreta en sentido contrario a su representación, es decir, si el RIP es descendente es que la asequibilidad va en aumento y cuando es creciente, el consumo de cigarrillos se hace más costoso en relación a la renta por habitante.

Gráfica N° 12

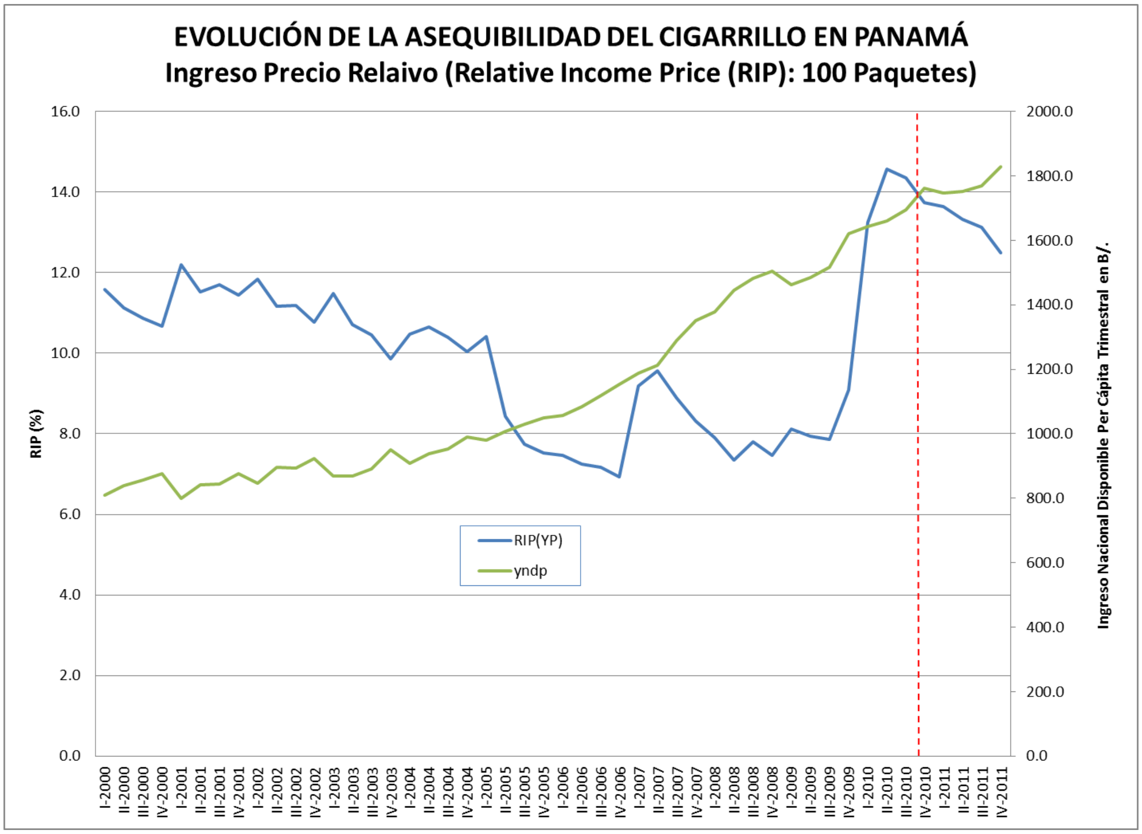

Fuente: Elaboración propia con datos de la Contraloría General de la República.

Estos elementos deben ser considerados respecto de la modelización de la demanda de cigarrillos y el cálculo econométrico de las elasticidades de corto y largo plazo, que se verán a partir de esta sección y escoger el modelo más adecuado para la construcción de la Curva de Laffer, con motivo de incrementar el ISC.

Cabe señalar que entre el primer trimestre del año 2000 y el segundo trimestre de 2005, con el incremento de la renta por habitante, la asequibilidad igualmente venía aumentando, aún con la adopción del Convenio Marco y las medidas restrictivas de 2005. De hecho, entre el segundo trimestre de 2005 y el primer trimestre de 2010, es cuando la asequibilidad (medida con el RIP), alcanza su mayor nivel, para luego caer momentáneamente entre el primer y cuarto trimestre de 2010, luego del incremento del ISC al 100%.

Se evidencia que el efecto de la medida impositiva tuvo una duración de un año, respecto de este indicador y abre la discusión con relación a mantener incrementos impositivos permanentes o periódicos, de manera que este efecto no neutralice el impacto de los incrementos del ISC, que es en todo caso el impuesto más flexible respecto de establecer nuevos incrementos, contrario al arancel de importación y del I.V.A., que requieren de reformas más complejas.

4.4.2. Formalización de las variables de los modelos de elasticidades.

Una vez analizadas las tendencias y comportamientos de las variables más importantes del mercado de los cigarrillos, principal producto de consumo de derivados del tabaco, se pasa a realizar una descripción general de las variables utilizadas para la estimación de las elasticidades de corto y largo plazo, mediante el uso de modelos econométricos, con miras a obtener una estimación de la Curva de Laffer con el mejor ajuste.

Demanda de cigarrillos (Qt): Esta variable se determinó utilizando las importaciones de cigarrillos, una vez separadas de las importaciones de tabaco y otros productos. Toda vez que en el caso de Panamá, desde finales de la década pasada no existe industria tabacalera y ésta a su vez se convirtió en importadora neta, las importaciones resultan ser una fuente directa para estimar el consumo de cigarrillos. Se utilizaron las importaciones en peso neto de cigarrillos (kilos netos) del Instituto Nacional de Estadística y Censos (INEC).

Precio de los cigarrillos (Pt): Esta variable se determinó mediante la relación del Índice de precios del tabaco (este indicador únicamente se construye con precios de cigarrillos), dividido por el Índice de precios total, es decir, IPC-de Tabaco/IPC-Total. El IPC- de tabaco, se construye tomando en consideración la marca de cigarrillos que más se vende en cada establecimiento comercial visitado y ello varía en cada establecimiento y área geográfica, y no es un ponderado de marcas. El año base actual del índice es 2002 y se utilizó la serie elaborada por el INEC.

Precio de los cigarrillos desfasados un período/base impositiva del Impuesto Selectivo del Consumo de Cigarrillos (Pt-1/Taxt): Esta variable se construyó como explicativa del precio de los cigarrillos con fines de poder construir una variable instrumental para el test de exogeneidad del precio.

Impuesto de importación de cigarrillos/importaciones de cigarrillos (TCt/Mt): Esta variable se construyó como explicativa del precio de los cigarrillos con fines de poder construir una variable instrumental para el test de exogeneidad del precio.

Renta Real Nacional Per Cápita (Yt): Esta variable se construyó a partir de la Renta Nacional Real Nacional Per Cápita anual, la cual fue trimestralizada mediante el programa estadístico ECOTRIM 1.01, utilizando como variable relacionada el PIB Trimestral. Se utilizó el método de trimestralización AR(1) Min SSR (Suma de residuos al cuadrado). La fuente de dicha información fueron las series de Cuentas Nacionales del INEC.

Población de 10 años y más: Esta variable se tomo con base en las proyecciones de la Contraloría General de la República de Panamá (INEC), Censo de 2010.

Tendencia lineal (Tt): Variable incluida para mejorar el ajuste de los modelos y construida por secuencia lineal numérica y utilizada en el caso de que fuera estadísticamente significativa.

Variables ficticias (Dt): Variables construidas para capturar el efecto de las medidas de control del consumo de tabaco y para ajustar la regresión ante la presencia de datos atípicos.

-Metodología y construcción de los modelos de demanda

Para la construcción de los modelos se ha utilizado como referencia el *"Economic Analisys of Tobacco Demand"* Toolkit N° 3 del Banco Mundial, en el cual se plantea la metodología a seguir para especificar los modelos de demanda de productos de tabaco y testear las hipótesis de adicción miope y adicción racional, de llegar a obtenerse modelos con buen ajuste.

Se especifican cuatro modelos de demanda doble logarítmicas con base en las cuales se obtendrán las elasticidades de corto y largo plazo, de las series trimestralizadas y que se plantean a continuación.

Modelo Convencional: Es un modelo estático de demanda, en el cual la cantidad demandada está dada para un período determinado, por las variables explicativas, dentro de dicho período, la forma funcional que asume es:

$$lnQ_t = \beta_0 + \beta_1 lnP_t + \beta_2 lnY_t + \beta_3 lnT_t + \beta_4 D_m + e_t \quad (1)$$

Donde:

lnQ_t = Logaritmo natural del consumo per cápita de cigarrillos en el período t.

lnP_t = Logaritmo natural del precio de los cigarrillos en el período t.

lnY_t = Logaritmo natural de la renta real per cápita en el período t.

T_t = Variable de tendencia temporal en el período t.

D_m = Variables ficticias intercepto para captar la introducción de restricciones en el período m, efectos políticas de corto plazo en la demanda de productos de tabaco, o residuos.

e_t = Término de error estocástico.

β_1 y β_2 = Elasticidades precio e ingreso de la demanda, respectivamente. Se espera que el signo de β_1 sea negativo.

Prueba de Engle-Granger: Es la prueba de cointegración que debe ser aplicada sobre los residuos del modelo convencional y determinar si las variables del mismo pueden ser combinación lineal, para obtener estimaciones dinámicas. La prueba se realiza de la siguiente manera:

$$\Delta e_t = \gamma e_{t-1} + \sum_{j=1}^{\rho} \Gamma_j \Delta e_{t-j} + \varepsilon_t \quad (2)$$

Donde: H_0: $\gamma = 0$ => $e_{t,}$ tiene una raíz unitaria. Donde: H_1: $\gamma < 0$ => $e_{t,}$ es estacionario.

Prueba de Hausman: Es importante probar la exogeneidad del precio y determinar si la función de demanda está debidamente identificada. En este caso H_0: Precio exógeno. H_1: Precio endógeno. El test se computa de la siguiente manera:

$$H = \frac{\left(\hat{\beta}_1^{\,IV} - \hat{\beta}_1^{\,OLS}\right)^2}{\left[Var\left(\hat{\beta}_1^{\,IV}\right) - Var\left(\hat{\beta}_1^{\,OLS}\right)\right]} \sim x_1^2 \quad (3)$$

Paso 1: Se estima la ecuación (1) por Mínimos Cuadrados Ordinarios y se obtienen:

$\hat{\beta}_1^{\,OLS}$ y $Var\left(\hat{\beta}_1^{\,OLS}\right)$

Paso 2: Se estima por variables instrumentales (Instrumental Variables, para el precio) la ecuación (1) y se obtienen:

$\hat{\beta}_1^{\,IV}$ y $Var\left(\hat{\beta}_1^{\,IV}\right)$

Modelo de corrección de error: Es un modelo dinámico utilizado para estimar las elasticidades de largo plazo, ante la existencia de cointegración[9] entre variables no estacionarias. Proporciona estimadores robustos. Su forma funcional es la siguiente:

$$\Delta lnQ_t = \rho_0 + \gamma Z_{t-1} + \sum_{j=0}^{k} \alpha_j \Delta\, lnP_{t-j} + \sum_{j=0}^{k} \delta_j \Delta\, lnY_{t-j} + \varepsilon_t \quad (4)$$

Donde el término de error rezagado es igual a:

[9] Para ello es necesario correr primero la prueba de cointegración con los residuos del modelo convencional y luego la hipótesis nula de existencia de raíz unitaria de la prueba de Engle-Granger.

$$\gamma Z_{t-1} = lnQt - \beta_0 - \beta_1 lnP_{t-1} - \beta_2 lnY_{t-1} \text{ (5)}$$

Mientras que:

α_j : Es la elasticidad precio de la demanda de corto plazo.

δ_j : Es la elasticidad ingreso de la demanda de corto plazo.

$\frac{\alpha_j}{\gamma}$: Es la elasticidad precio de la demanda de largo plazo.

$\frac{\delta_j}{\gamma}$: Es la elasticidad ingreso de la demanda de largo plazo.

Se espera además que el signo de γ sea negativo.

Modelo de Adicción Miope: Es un modelo dinámico, que incluye el consumo o demanda rezagada un período como variable explicativa, es decir, incorporando el consumo pasado. La forma funcional es la siguiente:

$$lnQ_t = \beta_0 + \beta_1 lnP_t + \beta_2 lnY_t + \beta_3 lnT_t + \beta_4 D_m + \sum_{j=1}^{k} \beta_{4+j}\, lnQ_{t-J} + \varepsilon_t \text{ (6)}$$

Donde:

β_1 : Es la elasticidad precio de la demanda de corto plazo.

β_2 : Es la elasticidad ingreso de la demanda de corto plazo.

$\frac{\beta_1}{1-\sum_{j=1}^{k}\beta_{4+j}}$: Es la elasticidad precio de la demanda de largo plazo.

$\frac{\beta_2}{1-\sum_{j=1}^{k}\beta_{4+j}}$: Es la elasticidad ingreso de la demanda de largo plazo.

Modelo de Adicción Racional: Es un modelo dinámico que incorpora no solo el consumo pasado, sino también el consumo futuro, mediante el cual el individuo maximiza su función de utilidad en el consumo.

$$lnQ_t = \beta_0 + \beta_1 lnP_t + \beta_2 lnY_t + \beta_3 lnT_t + \beta_4 D_m + \sum_{j=1}^{k} \beta_{4+j}\, lnQ_{t-j} + \sum_{j=k+1}^{m} \beta_{4+j}\, lnQ_{t+j-k} + \varepsilon_t \ (7)$$

Donde:

β_1 : Es la elasticidad precio de la demanda de corto plazo.

β_2 : Es la elasticidad ingreso de la demanda de corto plazo.

$\frac{\beta_1}{1-\sum_{j=1}^{k}\beta_{4+j}-\sum_{j=k+1}^{m}\beta_{4+j}}$: Es la elasticidad precio de la demanda de largo plazo.

$\frac{\beta_2}{1-\sum_{j=1}^{k}\beta_{4+j}-\sum_{j=k+1}^{m}\beta_{4+j}}$: Es la elasticidad ingreso de la demanda de largo plazo.

En el caso de los modelos dinámicos, si el precio es exógeno y hay cointegración de las series, éstos pueden ser estimados. En todos los casos se han realizado las pruebas de cointegración, en adición a otras pruebas de rigor econométrico para los diagnósticos que validen todos los modelos y que se presentan en las tablas de salida del análisis de los resultados a continuación.

4.4.3. Resultados de las estimaciones

A continuación se presentan las estimaciones con base a las principales pruebas econométricas, y los mejores ajustes obtenidos. Luego de hacer una descripción de la metodología a seguir para la estimación de las ecuaciones de demanda, se presentan los resultados de las mismas y su interpretación correspondiente.

Prueba de debilidad del instrumento (Pt):

Salida de regresión N° 1

```
Instrumental variables (2SLS) regression               Number of obs =      47
                                                       Wald chi2(4)  =  286.22
                                                       Prob > chi2   =  0.0000
                                                       R-squared     =  0.8614
                                                       Root MSE      =  .17439

------------------------------------------------------------------------------
          Qt |      Coef.   Std. Err.      z    P>|z|     [95% Conf. Interval]
-------------+----------------------------------------------------------------
          Pt |  -.7758919   .1434545    -5.41   0.000    -1.057058   -.4947262
          Yt |  -2.067324   .5376692    -3.84   0.000    -3.121136   -1.013511
          d1 |  -.4475887   .1034679    -4.33   0.000     -.650382   -.2447954
          Tt |   .0271019   .0094498     2.87   0.004     .0085806    .0456232
       _cons |   11.10269   3.558095     3.12   0.002     4.128952    18.07643
------------------------------------------------------------------------------
Instrumented:  Pt
Instruments:   Yt d1 Tt Pt-1  Tax/Qt Pt/Tax d1(escalón Q2:2005 a Q1: 2006)
```

First-stage regression summary statistics

Variable	R-sq.	Adjusted R-sq.	Partial R-sq.	F(3,40)	Prob > F
Pt	0.9560	0.9494	0.8564	79.5241	0.0000

Minimum eigenvalue statistic = 79.5241

Critical Values	# of endogenous regressors: 1			
**Ho: Instruments are weak**	# of excluded instruments: 3			
	5%	10%	20%	30%
2SLS relative bias	13.91	9.08	6.46	5.39
	10%	15%	20%	25%
2SLS Size of nominal 5% Wald test	22.30	12.83	9.54	7.80
LIML Size of nominal 5% Wald test	6.46	4.36	3.69	3.32

Como se observa en las salidas de regresión luego de contrastar los resultados, el valor de la F y su probabilidad, hacen rechazar H_0 al 5%, incluso con el test de Wald, indicando que el precio no es un instrumento débil y es válido para realizar la estimación por variable instrumental, mediante la técnica de mínimos cuadrados ordinarios en dos etapas y poder realizar la prueba de Hausman.

Prueba de Hausman:

Salida de regresión N° 2

Instrumental variables (2SLS) regression

Source	SS	df	MS
Model	8.87910336	4	2.21977584
Residual	1.43088125	42	.034068601
Total	10.3099846	46	.2241301

Number of obs	= 47
F(4, 42)	= 63.50
Prob > F	= 0.0000
R-squared	= 0.8612
Adj R-squared	= 0.8480
Root MSE	= .18458

Qt	Coef.	Std. Err.	t	P>|t|	[95% Conf. Interval]	
Pt	-.7550566	.1522796	-4.96	0.000	-1.062369	-.447744
Yt	-2.105881	.569474	-3.70	0.001	-3.255126	-.9566359
d1	-.443404	.1095345	-4.05	0.000	-.6644535	-.2223545
Tt	.0274576	.0100036	2.74	0.009	.0072696	.0476457
_cons	11.36234	3.768664	3.01	0.004	3.756868	18.96781

Instrumented: Pt
Instruments: Yt d1(escalón Q2:2005 a Q1: 2006) Tt Pt-1 Tax/Qt (Pt/Tax)-1

(b) Coeficientes betas del modelo convencional (moc)

```
(B)Coeficientes betas obtenidos de la estimación por variables instrumentales (ivreg)

             |      (b)          (B)            (b-B)     sqrt(diag(V_b-V_B))
             |      moc           .           Difference          S.E.
-------------+-------------------------------------------------------------
          Pt |   -.7856088    -.7550566       -.0305522               .
          Yt |   -2.041723    -2.105881        .064158                .
          d1 |   -.4491926     -.443404       -.0057886               .
        That |     .0267609     .0274576       -.0006968               .
---------------------------------------------------------------------------
            b = consistent under Ho and Ha; obtained from regress
            B = inconsistent under Ha, efficient under Ho; obtained from ire

    Test:  Ho:  difference in coefficients not systematic

                  chi2 (2) = (b-B)'[(Vibe-V_B) ^ (-1)] (b-B)
                          =    -0.28    chi2<0
```

Con relación a la prueba de Hauman, se acepta H_0: Es decir, que el precio es una variable exógena y es posible utilizarlo en la estimación de la función de demanda. Esta prueba es importante haberla realizado para eliminar cualquier duda con relación al uso de dicha variable como explicativa. Con base en estos resultados se puede proceder a estimar los modelos a contrastar: modelo convencional, corrección de errores, adicción miope y adicción racional. En este caso se incluyen los resultados de las estimaciones y los problemas de ajuste para los modelos propuestos.

Modelo convencional: La variable explicada (demanda) y las variables explicativas, precio y renta por habitante fueron transformadas en logaritmo natural.

En la salida inicial del modelo convencional, se observa la presencia de auto correlación serial, respecto del estadístico Durbin-Watson.

Salida de regresión N° 3

```
      Source |       SS       df       MS              Number of obs =      48
-------------+------------------------------           F(  4,    43) =   67.37
       Model |  8.95854469     4  2.23963617           Prob > F      =  0.0000
    Residual |  1.42938685    43  .033241555           R-squared     =  0.8624
-------------+------------------------------           Adj R-squared =  0.8496
       Total |  10.3879315    47  .22101982            Root MSE      =  .18232

------------------------------------------------------------------------------
    Qt       |      Coef.    Std. Err.      t     P>|t|      [95% Conf. Interval]
-------------+----------------------------------------------------------------
          Pt |  -.7856092   .1387755    -5.66   0.000    -1.065477   -.5057417
          Yt |  -2.041726   .5395153    -3.78   0.000    -3.129762   -.9536896
          Tt |   .0267609   .0094368     2.84   0.007     .0077298     .045792
          d1 |   -.449193   .1074018    -4.18   0.000    -.6657893   -.2325967
       _cons |   10.93249   3.572421     3.06   0.004     3.728012    18.13696
------------------------------------------------------------------------------
Nota: (escalón Q2:2005 a Q1: 2006)
Durbin-Watson d-statistic (5,48) =  1.628174 dl = 1.335  du = 1.771
```

Para resolver el problema de la autocorrelación serial, se propuso la técnica iterativa de Cochrane-Orcutt, como método correctivo. En este caso se estima el coeficiente **ρ (Rho)**, mediante la regresión de los residuos del primer modelo de la siguiente manera: $\hat{u} = \hat{\rho}\hat{u}_{t-1} + v$

Salida de regresión N° 4

```
Cochrane-Orcutt AR(1) regression -- iterated estimates

      Source |       SS       df       MS              Number of obs =      47
-------------+------------------------------           F(  4,    42) =   44.96
       Model |  5.90522445     4  1.47630611           Prob > F      =  0.0000
    Residual |  1.37898278    42  .032832923           R-squared     =  0.8107
-------------+------------------------------           Adj R-squared =  0.7927
       Total |  7.28420723    46  .158352331           Root MSE      =   .1812

------------------------------------------------------------------------------
    Qt       |      Coef.   Std. Err.      t    P>|t|     [95% Conf. Interval]
-------------+----------------------------------------------------------------
          Pt |  -.8171555   .1647336    -4.96   0.000    -1.149601   -.4847096
          Yt |   -1.83698   .6431544    -2.86   0.007    -3.134918   -.5390418
          d1 |  -.4243289   .1212762    -3.50   0.001    -.6690743   -.1795836
          Tt |   .0233704   .0115862     2.02   0.050    -.0000114    .0467523
       _cons |   9.575304   4.251251     2.25   0.030     .9959324    18.15468
-------------+----------------------------------------------------------------
         rho |   .1999656
------------------------------------------------------------------------------
Nota: Variables transformadas en logaritmo natural. d1(escalón Q2:2005 a Q1:
2006): Variable ficticia que recoge el efecto del Decreto Ejecutivo N° 17 del
11 de marzo de 2005, de la aplicación la prohibición de fumar en restaurantes,
advertencias sanitarias, publicidad, entre otras.

Durbin-Watson statistic (original)    1.628174
Durbin-Watson statistic (transformed) 2.084325
```

Vistos los nuevos resultados en el cual el estadístico Durbin-Watson entra en rango, permite hacer inferencias respecto de los parámetros de las variables explicativas principales, en este caso el precio y la renta por habitante. Todas las variables explicativas fundamentales tuvieron significancia estadística. Respecto del precio, el coeficiente de elasticidad (-0.82), resulta un tanto elevado, aunque es de tomar en cuenta que el incremento en el ISC al igual que las medidas restrictivas has tenido su impacto en el consumo, afectando su dinámica de largo plazo.

Respecto de la renta por habitante, el signo resultante es negativo (-1.84), indicando que este bien se ha tornado como un bien inferior, pero en valor absoluto, es mayor que uno, indicando que el consumo se ha tornado relativamente en un bien de lujo. Este resultado es hasta ahora contradictorio con relación al comportamiento esperado del consumo respecto del ingreso, del cual se esperaría tener un parámetro con signo positivo. Sin embargo, dadas las prevalencias y la reducción de la participación del tabaco en la estructura de consumo general, merece igual consideración.

Un argumento que salta a todas luces es la posible presencia de contrabando, como ya se ha señalado al presentar estos resultados en distintos foros relacionados con la política fiscal

antitabáquica. Queda en todo caso ampliar este hallazgo con los resultados de la encuesta de marcas y de percepción en establecimientos de atención al público con relación a las medidas de control, para dilucidar en parte esta situación. De igual forma los resultados posteriores de la Global Adult Tobacco Survey-GATS en adultos, deben proporcionar estimaciones de las prevalencias a 30 días actualizadas y que contrasten esta nueva hipótesis, respecto al cambio estructural del consumo de cigarrillos en Panamá.

Respecto de la variable ficticia d1 (dummy), la misma recoge eficientemente el impacto de las medidas de control a partir del año 2005 (-0.42), las cuales tuvieron un efecto negativo en el consumo, como cabría esperar, y con lo cual el signo fue consistente, indicando que han influido tanto las medidas restrictivas de ambientes libres de humo, advertencias sanitarias, restricción de la publicidad, entre otras, en adición a las medidas fiscales. Pese a este resultado solo se tienen estimaciones estáticas de las elasticidades y por tal razón se realizó la prueba de cointegración de Engle-Granger, que consiste en una prueba de Dickey-Fuller sobre los residuos del modelo convencional de la primera regresión, para ver la posibilidad de tener estimaciones dinámicas.

La prueba de cointegración se hizo sobre los residuos de la primera regresión del modelo convencional y ver si existe o raíz unitaria. Los resultados se presentan a continuación:

Salida de regresión N° 5

Dickey-Fuller test for unit root Number of obs = 47

	Test Statistic	Interpolated Dickey-Fuller 1% Critical Value	5% Critical Value	10% Critical Value
Z(t)	-5.541	-3.600	-2.938	-2.604

MacKinnon approximate p-value for Z(t) = 0.0000

Los resultados de la prueba indican que no existe raíz unitaria en los residuos del primer modelo convencional estimado, con lo cual es posible tener estimaciones de modelos dinámicos, como el de corrección de errores, adicción miope y adicción racional. Es decir, que en este caso las variables explicativas y dependiente pueden ser combinación lineal y obtener estimaciones dinámicas.

Modelo de corrección de errores: Los resultados del modelo de corrección de errores se presentan a continuación. **Ut** (residuos del modelo convencional inicial).

Salida de regresión N° 6

Source	SS	df	MS		
Model	1.33631323	4	.334078308	Number of obs =	46
Residual	1.39781692	42	.033281355	F(4, 42) =	10.04
				Prob > F =	0.0000
Total	2.73413015	46	.059437612	R-squared =	0.4888
				Adj R-squared =	0.4401
				Root MSE =	.18243

ΔQt-1	Coef.	Std. Err.	t	P>|t|	[95% Conf.	Interval]
ΔPt-1	-.9467925	.3198244	-2.96	0.005	-1.592224	-.3013606
ΔYt-2	1.682651	.8310296	2.02	0.049	.005565	3.359736
ut-1	-.7404278	.1552118	-4.77	0.000	-1.053658	-.4271976
d5	-.3697206	.1877973	-1.97	0.056	-.748711	.0092697

Nota: Variables transformadas en logaritmo natural. d5(Q:3 2008) Variable ficticia que filtra el outlier del tercer trimestre de 2008 y que podría recoger el efecto de la ley 13 del 11 enero de 2008 (prohibición de fumar en espacios cerrados y abiertos).

Durbin-Watson d-statistic(4,46) = 2.093415

En general el modelo tiene un ajuste regular desde el punto de vista estadístico sobre la base del estadístico t-Student, tanto la renta como la variable ficticia están en el límite del nivel de confianza del 5%. Empero el coeficiente del precio, aunque cumple con el signo, esta relativamente elevado respecto de los dos modelos anteriores. Ante el hecho de que el consumo de tabaco es altamente adictivo, cabría esperar un coeficiente de elasticidad precio mucho más bajo. No obstante, es de tener en cuenta el incremento de la elasticidad entre 2010 y 2011, por el incremento del ISC, que ha podido influir en dicho resultado.

El resultado de la elasticidad precio, tiene implicaciones respecto del cálculo de la Curva de Laffer, con miras a establecer un umbral máximo de elevación del ISC y mantener la recaudación fiscal. Elasticidades precio de la demanda bajas, suelen dejar como resultado umbrales mucho más amplios, que a elasticidades cercanas a uno. Empero, conviene realizar otras estimaciones dinámicas, como el modelo de adicción miope y el modelo de adicción racional, con fines de obtener otros ajustes.

Modelo de adicción miope:

El modelo de adicción miope es una propuesta que se basa en la formación de expectativas, sobre la base del consumo pasado. Es decir, que el fumador miope forma expectativas de consumo, trasladando la información conocida del pasado. Los resultados de la corrida reflejan que en general los coeficientes son estadísticamente significativos a excepción de la constante con un valor de p = 0.154.

Salida de regresión N° 7

Source	SS	df	MS
Model	9.01759607	5	1.80351921
Residual	1.29239424	41	.031521811
Total	10.3099903	46	.224130224

Number of obs	= 47
F(5, 41)	= 57.21
Prob > F	= 0.0000
R-squared	= 0.8746
Adj R-squared	= 0.8594
Root MSE	= .17754

Qt	Coef.	Std. Err.	t	P>|t|	[95% Conf.	Interval]
Pt	-.6647696	.1604454	-4.14	0.000	-.9887958	-.3407435
Yt	-.5421184	.1834064	-2.96	0.005	-.9125152	-.1717217
Qt-1	.2374701	.1222359	1.94	0.059	-.0093904	.4843305
d1	-.307209	.1054472	-2.91	0.006	-.5201639	-.0942541
d3	-.4885176	.1848755	-2.64	0.012	-.8618811	-.115154
_cons	1.703436	1.172887	1.45	0.154	-.6652577	4.07213

Nota: Variables transformadas en logaritmo natural. d1 y d3 son variables ficticias, donde d1, es un escalón (Q2:2005 a Q1:2006) y d3 es un outlier (Q:2 2001).

Durbin-Watson d-statistic(6,47) = 2.290066

Al analizar los resultados, respecto del coeficiente del precio (-0.66), el mismo resulta mucho más bajo que con relación al modelo convencional y el modelo de corrección de errores. De igual manera el coeficiente del ingreso, resultó mucho más bajo (-0.54). Se trata de un tipo de fumador menos sensible ante variaciones en el precio y en el ingreso, empero dicho modelo no dio un ajuste esperado con relación al término constante, el cual representa la demanda autónoma y por ende tiene una interpretación importante dentro de la función de demanda. De igual manera es importante el coeficiente de la demanda rezagada (0.24) un período (trimestre), que mide el componente de expectativa, indicando que el consumo pasado influye significativamente en el consumo presente para este tipo de fumadores, pero con un p-value de 0.059 muy al límite.

Modelo de adicción racional:

Finalmente se presenta la estimación del modelo racional. Es el que menor ajuste presenta respecto de todas las variables y es mucho más complejo por incluir la demanda rezagada hacia

atrás y hacia adelante, dado que este tipo de consumidores establecen su consumo presente sobre la base del consumo pasado y futuro.

En general este modelo arroja coeficientes de elasticidades muy bajos, para el precio (-0.31) y el ingreso (-22), mientras que con relación al consumo pasado (0.28) y futuro (.33), no se observa una diferencia significativa.

Salida de regresión N° 8

```
      Source |       SS       df       MS              Number of obs =      46
-------------+------------------------------           F(  5,    40) =   39.48
       Model |  7.76595576     5  1.55319115           Prob > F      =  0.0000
    Residual |  1.57367172    40  .039341793           R-squared     =  0.8315
-------------+------------------------------           Adj R-squared =  0.8104
       Total |  9.33962748    45  .207547277           Root MSE      =  .19835

------------------------------------------------------------------------------
    Qt       |      Coef.   Std. Err.      t    P>|t|     [95% Conf. Interval]
-------------+----------------------------------------------------------------
        Pt-1|  -.3144392   .1740528    -1.81   0.078    -.6662131    .0373346
        Yt-1|  -.2241143    .238195    -0.94   0.352    -.7055244    .2572958
        Qt-1|   .2793375   .1507146     1.85   0.071     -.025268    .5839431
        Qt+1|   .3323318   .1444195     2.30   0.027      .040449    .6242146
          d5|   -.410407   .2089502    -1.96   0.056    -.8327112    .0118972
       _cons |   .4836398   1.413316     0.34   0.734    -2.372777    3.340057
------------------------------------------------------------------------------

Durbin-Watson d-statistic(6,46) =  2.831822
```

Aunque se requiere seguir trabajando con este modelo y mejores datos para obtener resultados más ajustados y que tengan significancia estadística, no deja de tener importancia con relación a la tipificación del consumo, desde un punto de vista dinámico, respecto del posible impacto de las medidas de control y fiscales.

De todos los modelos realizados, hasta ahora los que mejor ajuste ha presentado son el modelo convencional ajustado por autocorrelación serial y el modelo de corrección de errores, éste último con un coeficiente de elasticidad precio de la demanda relativamente alto. Por ende se presenta el cálculo de las elasticidades de largo plazo a partir del modelo convencional corregido (autocorrelacion serial). Los resultados del cálculo para la elasticidad de largo plazo del precio y del ingreso, fueron los siguientes:

Elasticidad precio de la demanda de largo plazo: -0.82

Elasticidad ingreso de la demanda de largo plazo: -1.84

Ambos coeficientes son relativamente altos, y sobre todo respecto del coeficiente del precio, lo cual tendrá implicaciones en la estimación de la Curva de Laffer, para establecer un nuevo umbral de incremento del ISC.

4.4.4. Simulación del impacto fiscal de los incrementos del ISC.

Con fundamento en el cálculo de la elasticidad de largo plazo, obtenida del modelo convencional, se hizo una primera aproximación del umbral de incremento a partir de la construcción de la Curva de Laffer. Debe tomarse en consideración que en el año 2009 se hizo el último incremento sobre la base de una elasticidad de largo plazo de |-1.04| y con un precio promedio de B/. 1.83 para las principales marcas. Los resultados de esas estimaciones se presentan en el anexo. De acuerdo a esa estimación de la Curva de Laffer, el máximo incremento posible, fue del 100%, tal como se plasmo en la última modificación. Es de saber que se trata de una modificación reciente y en análisis previos se ha evidenciado el incremento marcado en el precio, con la continuada caída de las importaciones. El cálculo de la elasticidad arco para el período que va del tercer trimestre de 2009 al cuarto trimestre de 2011, de |-1.77|, indica un cambio estructural en el patrón de demanda, a corto plazo, como respuesta a dicha medida.

Dada su implementación desde hace dos años, las bajas prevalencias registradas en Panamá, entre otros factores, es posible, que el incremento en el ISC resultante sea muy estrecho, respecto de la estimación para el período 1999-2009[10], comparada con la de 2000-2011, con el objetivo de capturar el nuevo efecto de la medida fiscal.

[10] Herrera Víctor Hugo. Estudio de la demanda de Cigarrillos y Tabaco en Panamá CIET-MINSA. Marzo de 2010. http://www.minsa.gob.pa/minsa/tl_files/documents/informacion_salud/control_tabaco/Estudio%20de%20la%20Demanda%20de%20Tabaco%20CIET-Panama-Abril-2010.pdf

Gráfica N° 13

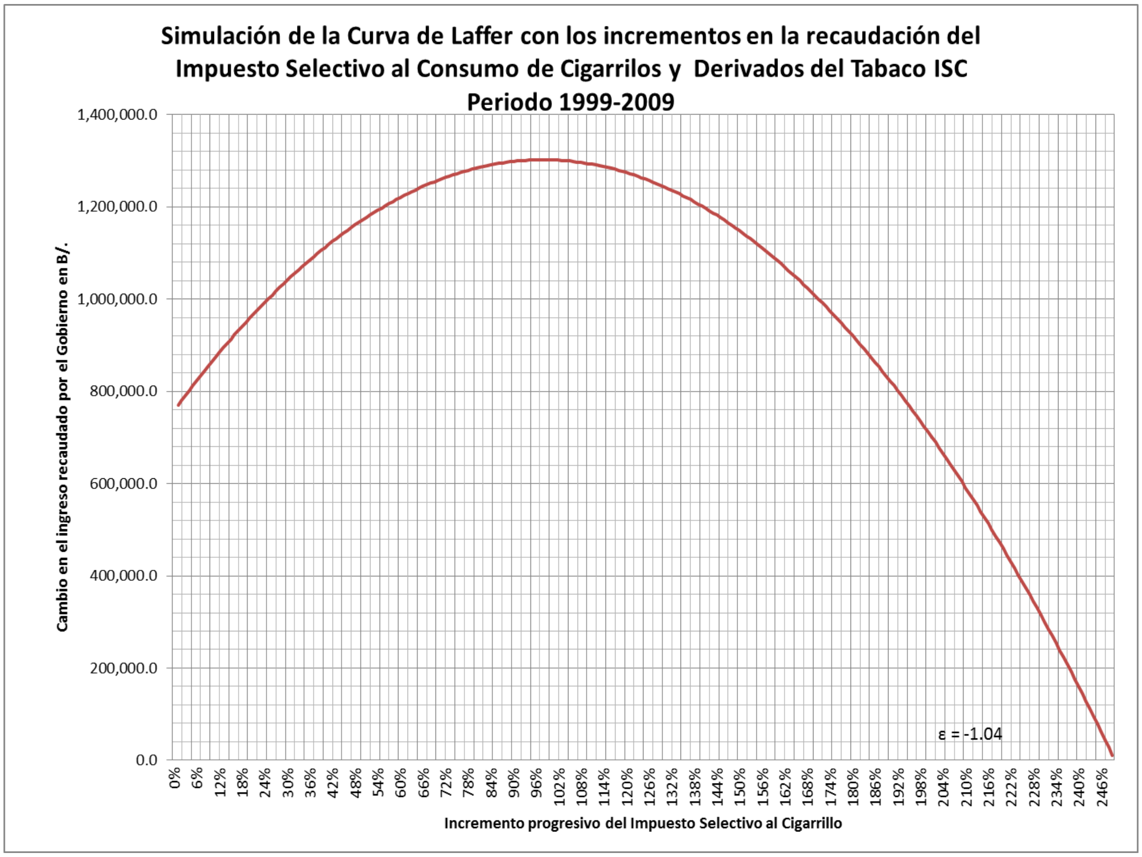

Fuente: Elaboración propia con datos de la Contraloría General de la República y de la Dirección General de Ingresos del Ministerio de Economía y Finanzas.

Como se observa en el primer gráfico, se justificaba un incremento del 100%, con un precio promedio de B/. 1.83 (Gráfica N° 13). Sin embargo, pasados dos años al actualizar los datos y recalcular nuevamente la simulación[11], pese a que la elasticidad precio de largo plazo es menor, el

---

[11] Nota: Para fines prácticos se presenta la conceptualización de la simulación de la Curva de Laffer con las variables correspondientes.

Pr: Precio al por menor de último mes en la data (B/.3.63 por paquete de 20 cigarrillos promedio de seis marcas)

Pr (New) = Pr+Ps*Dt: Es el nuevo precio al por menor derivado de un incremento de 1% en el impuesto (Tax).

Tax: Impuesto Selectivo al Consumo de Cigarrillos-ISC (100%)

Ps = Pr*Tax: Proporción del precio al consumidor que representa el ISC.

Ps(New) = Ps × (1 + Dt)

Dt: Incremento del ISC, comenzando por el 1%.

ε_p: Elasticidad precio de la demanda de largo plazo, del modelo de corrección de errores (–0.82)

ΔPr = [Pr(new)/Pr] − 1

C = Consumo derivado del último mes en la data (derivado por regla de tres de las ingresos tributarios y la base impositiva)

$\Delta C = \Delta Pr \times \varepsilon_p$

C(New) = (1 + ΔC) × C

R: Ingresos colectados por el Gobierno derivados del ISC.

R(new) = C(new) × Ps(new)

precio promedio es más alto, B/. 3.63, lo que implica, que el precio se dobló, como cabría esperar después del incremento de finales de 2009 (Gráfica N° 14).

Gráfica N° 14

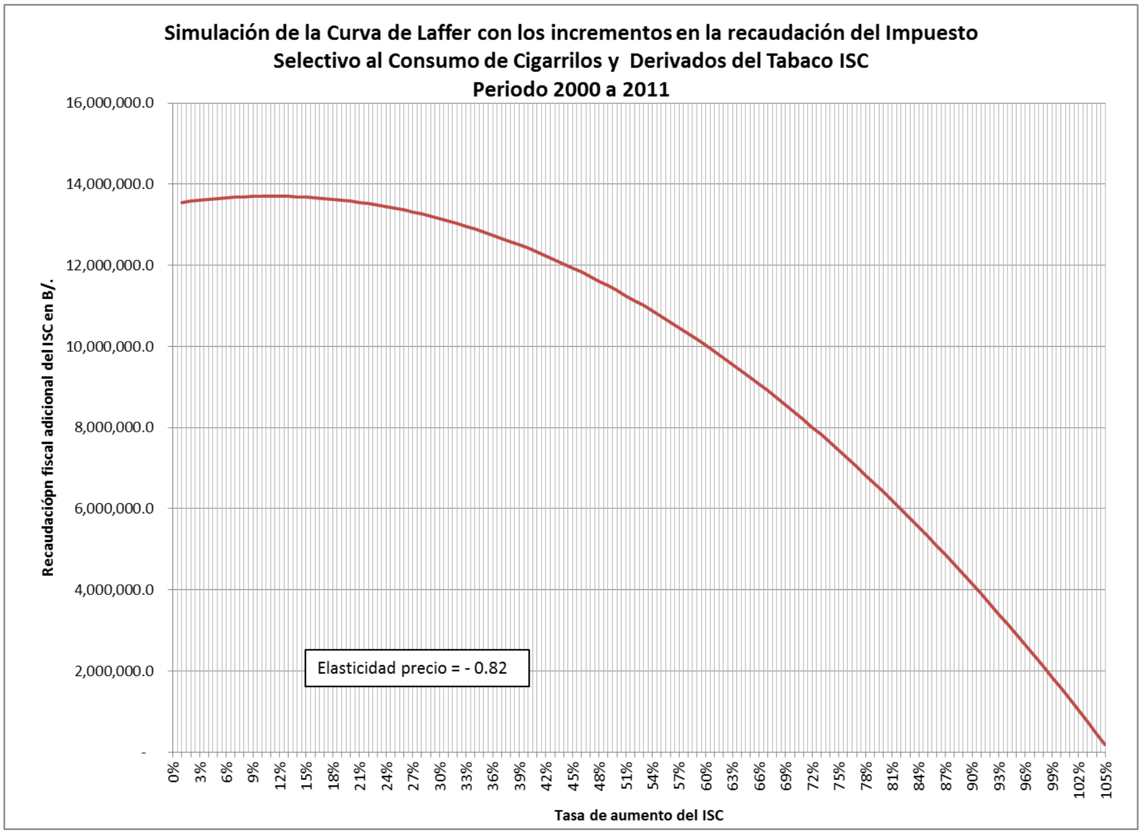

Fuente: Elaboración propia con datos de la Contraloría General de la República y de la Dirección General de Ingresos del Ministerio de Economía y Finanzas.

En este caso se hace mucho más estrecha la Curva de Laffer dejando apenas un umbral del 12%, para futuros incrementos del ISC, como un resultado preliminar. No obstante, se deben seguir afinando las estimaciones respecto de los modelos, convencional, corrección de errores y adicción miope y racional, a fin de obtener mejores estimadores con nuevos datos y para un período mucho mayor al considerado. Este ejercicio revela, que dadas las características de la demanda en Panamá y de los resultados hasta ahora obtenidos, no da lugar para hacer grandes incrementos futuros del ISC a corto plazo, ante la presencia de bajas prevalencias y una caída sostenida de las importaciones. Ello deja un margen de maniobra más estrecho respecto del uso del ISC, aunque también puede explorarse incrementos futuros del I.V.A. (importación de tabaco).

Otro escenario que puede explorarse, es el de la asequibilidad con relación al ingreso por habitante, retomando la discusión propuesta con base en la Gráfica N° 12. Es importante tomar en consideración medidas de política fiscal que contribuyan no solo a encarecer el precio del

tabaco a corto plazo, sino también en neutralizar el efecto que tiene el incremento de la Renta Per Cápita a corto y largo plazo.

Este es uno de los principales retos a tener en cuenta a futuro y la legislación fiscal específica (Impuesto Selectivo al Cigarrillo), debe tomar en consideración este elemento. Como se ha mencionado, la asequibilidad es producto de incrementos en la renta, a mediano y largo plazo, que pueden dejar sin efecto, los incrementos en la tasa impositiva a efectos de reducir el consumo de cigarrillos.

Con base en estos argumentos se incluye un ejercicio de simulación respecto de las medidas que pueden tomarse a futuro para neutralizar el aumento de la asequibilidad de los productos de tabaco a mediano y largo plazo.

Una medida alternativa, es utilizar la tasa de crecimiento histórica anual de la renta nacional disponible per cápita (TCYP) y multiplicarla por la tasa nominal de crecimiento promedio anual del Índice de precios de los cigarrillos (TIPT) y luego restar este producto de 1, con lo cual se obtiene la tasa de incremento anual del impuesto para neutralizar el aumento de la asequibilidad de los cigarrillos. La fórmula general sería [{(1+TCYP)(1+TIPT)-1}X100][12].

Mediante el cálculo interpolado de la tasa histórica trimestral de crecimiento de la renta nacional disponible per cápita y de la tasa de aumento del precio del cigarrillo, las cuales luego se llevaron a valores anuales, se hizo este ejercicio de simulación con el siguiente resultado.

Al remplazar los valores en la fórmula se obtiene como resultado: [{(1.0167)(1.0265)-1} x 100] = 4.36%, anual y acumulada a cinco años 21.8%. Con relación a este escenario, se plantea un incremento nominal del precio del cigarrillo vía el impuesto específico por año, de 4.36%, que de acuerdo a resultados obtenidos en otros países tales como el caso de Sudáfrica y China, son de 12.3% y 18.8% respectivamente, para hacer el consumo de cigarrillos menos asequible por año. En el caso de China, la tasa de crecimiento de la renta per cápita es relativamente más alta, al ser una economía de rápido crecimiento económico, al tener una tasa anual del 10% e inflación del 8%, mientras que con relación a Sudáfrica su tasa de crecimiento anual es 4% y una tasa de inflación similar a la de China, de 8%, más altas en ambos casos que la de Panamá.

Desde esta perspectiva este es otro argumento a tomar en cuenta con relación a otras medidas utilizadas para reducir el consumo y sobre todo por el hecho que se pueden establecer cláusulas de aplicación automática, que se introduzcan en la legislación fiscal del país, con miras a que se establezcan incrementos sucesivos cada año, indexando el ISC de manera automática, potenciando la eficiencia y la eficacia de este tipo de dispositivo o cláusula gatillo (de aplicación automática).

[12] Blecher Evan & Walbeek Corné Van. An Analysis of Cigarrete Affordability. International Tobaco Control Research American Cancer Society & School of Economics, University of Cape Town. 2008. Página 19.

V. Resultados generales de las encuestas de marcas y de locales de atención al público

A continuación se presentan algunos resultados generales de las encuestas realizadas para ampliar lo referente a la demanda y comercialización de cigarrillos, respecto de las percepciones de consumidores y dueños de locales de atención al público, con relación a algunos indicadores básicos como se presentarán en los puntos siguientes. Cabe mencionar que de cada encuesta se ha hecho un informe de los resultados relevantes en dos documentos independientes, que complementan este informe.

Ambas encuestas se realizaron tomando en consideración los distritos de Panamá, San Miguelito (ubicados en la Provincia de Panamá), Colón (distrito cabecera de la Provincia de Colón), David y Barú (en la Provincia de Chiriquí). La razón, es por ser las zonas de mayor comercio de tabaco en el ámbito nacional, pero más importante aún es que son las zonas más proclives de entrada de contrabando. En el caso de las provincias de Colón y Panamá, la Zona Libre de Colón es el principal punto de entrada de contrabando al norte del país (en el Océano Atlántico), mientras que en la Provincia de Chiriquí, la frontera de Paso Canoa, limítrofe con Costa Rica, es otro de los principales puntos de entrada al oeste del país.

En el caso de la encuesta de marcas (consumidores), la muestra fue de 1,893 individuos y en la de locales, un total de 930 locales. Los resultados se presentan en dos secciones separadas y finalmente se ha añadido una sección de balance general para efectos de futuras políticas de intervención en el control del consumo de tabaco.

5.1. Encuesta de marcas

Para la presentación de esta encuesta se han incorporado algunos cuadros básicos, que no pretenden ser exhaustivos respecto de todos los resultados posibles, por lo que solo se pretende con los mismos la consolidación y presentación de algunos resultados generales.

Prevalencias de tabaquismo

Uno de los indicadores fundamentales en materia de consumo de tabaco son las prevalencias a treinta días, las cuales establecen el parámetro internacional de comparación no solo de país, sino también en el ámbito regional local.

Pese a ser una encuesta aplicada en dominios específicos y no de ámbito nacional, las prevalencias generales no se alejan demasiado de las ya analizadas para años anteriores e incluso por sexo para el ámbito nacional. La prevalencia general resultante fue del 14.2%, siendo de 20.7% para los hombres y de 6.5% en las mujeres. Ello implica que la población fumadora activa según los

entrevistados es más alta en los hombres que en las mujeres como cabría esperar y es una tendencia que se mantiene para todos cinco distritos.

Tabla N° 7

Prevalencia de Fumador Según Distrito y Sexo

Distrito y sexo	Total	%	Condición de fumador			
			Fuma		No Fuma	
			Número	%	Número	%
**TOTAL**	**1,893**	**100.0**	**281**	**14.8**	**1,612**	**85.2**
Hombre	1,110	58.6	230	20.7	880	**79.3**
Mujer	783	41.4	51	6.5	732	**93.5**
**Barú**	**124**	**100.0**	**10**	**8.1**	**114**	**91.9**
Hombre	90	72.6	10	11.1	80	**88.9**
Mujer	34	27.4	0	0.0	34	**100.0**
**Colón**	**471**	**100.0**	**71**	**15.1**	**400**	**84.9**
Hombre	279	59.2	58	20.8	221	**79.2**
Mujer	192	40.8	13	6.8	179	**93.2**
**David**	**350**	**100.0**	**70**	**20.0**	**280**	**80.0**
Hombre	252	72.0	66	26.2	186	**73.8**
Mujer	98	28.0	4	4.1	94	**95.9**
**Panamá**	**471**	**100.0**	**64**	**13.6**	**407**	**86.4**
Hombre	267	56.7	47	17.6	220	**82.4**
Mujer	204	43.3	17	8.3	187	**91.7**
**San Miguelito**	**477**	**100.0**	**66**	**13.8**	**411**	**86.2**
Hombre	222	46.5	49	22.1	173	**77.9**
Mujer	255	53.5	17	6.7	238	**93.3**

Fuente: Encuesta de marcas 2012.

Los distritos de Colón y David, presentan las prevalencias generales más altas con 15.1% y 20.1%, siendo precisamente los dos distritos adyacentes a los puntos de entrada de importaciones de cigarrillos. Mientras que los distritos de Panamá y San Miguelito se ubican en el lugar intermedio con el 13.6% y 13.8% respectivamente. Finalmente el distrito de Barú con el tamaño de muestra más bajo, se ubica con la prevalencia más baja con el 8.1%.

Al observar las prevalencias por grupos quinquenales de edades, son los grupos comprendidos entre los 20 a 24 años y de 25 a 29 años, los que presentan las mayores prevalencias con 18.3% y 15.7% respectivamente, indicando que son grupos de población joven y en edad laboral activa. Situación que puede acarrear problemas posteriores sobre la salud pública respecto de la carga social de enfermedades ligadas al tabaquismo.

Tabla N° 8

Prevalencia de Fumador Según Grupos de Edad y Sexo

Grupo de edad y sexo	Total	%	Condición de fumador			
			Fuma	%	No fuma	%
**TOTAL……….**	**1,893**	**100.0**	**281**	**14.8**	**1,612**	**85.2**
Hombre	1,110	58.6	230	**20.7**	880	79.3
Mujer	783	41.4	51	**6.5**	732	93.5
**15 - 19**	**127**	**6.7**	**13**	**10.2**	**114**	**89.8**
Hombre	67	3.5	12	**17.9**	55	82.1
Mujer	60	3.2	1	**1.7**	59	98.3
**20 - 24**	**224**	**11.8**	**41**	**18.3**	**183**	**81.7**
Hombre	94	5.0	28	**29.8**	66	70.2
Mujer	130	6.9	13	**10.0**	117	90.0
**25 - 29**	**274**	**14.5**	**43**	**15.7**	**231**	**84.3**
Hombre	141	7.4	35	**24.8**	106	75.2
Mujer	133	7.0	8	**6.0**	125	94.0
**30 - 34**	**222**	**11.7**	**29**	**13.1**	**193**	**86.9**
Hombre	117	6.2	21	**17.9**	96	82.1
Mujer	105	5.5	8	**7.6**	97	92.4
**35 - 39**	**195**	**10.3**	**31**	**15.9**	**164**	**84.1**
Hombre	112	5.9	25	**22.3**	87	77.7
Mujer	83	4.4	6	**7.2**	77	92.8
**40 - 44**	**189**	**10.0**	**25**	**13.2**	**164**	**86.8**
Hombre	124	6.6	23	**18.5**	101	81.5
Mujer	65	3.4	2	**3.1**	63	96.9
**45 - 49**	**167**	**8.8**	**28**	**16.8**	**139**	**83.2**
Hombre	119	6.3	25	**21.0**	94	79.0
Mujer	48	2.5	3	**6.3**	45	93.8

Grupo de edad y sexo	Total	%	Condición de fumador			
			Fuma	%	No fuma	%
**50 - 54**	**126**	**6.7**	**10**	**7.9**	**116**	**92.1**
Hombre	74	3.9	8	**10.8**	66	89.2
Mujer	52	2.7	2	**3.8**	50	96.2
**55 - 59**	**109**	**5.8**	**25**	**22.9**	**84**	**77.1**
Hombre	75	4.0	21	**28.0**	54	72.0
Mujer	34	1.8	4	**11.8**	30	88.2
**60 - 64**	**107**	**5.7**	**19**	**17.8**	**88**	**82.2**
Hombre	71	3.8	15	**21.1**	56	78.9
Mujer	36	1.9	4	**11.1**	32	88.9
**65 y más**	**153**	**8.1**	**17**	**11.1**	**136**	**88.9**
Hombre	116	6.1	17	**14.7**	99	85.3
Mujer	37	2.0	0	**0.0**	37	100.0

Fuente: Encuesta de marcas 2012.

Este aspecto es de vital importancia con relación a los efectos inter-generacionales del tabaquismo y obliga a seguir estableciendo el uso de medidas de control sobre el consumo, pero sobre todo de concienciación sobre los efectos en la salud respecto de las enfermedades crónicas no transmisibles ligadas al mismo.

Al analizar la situación de acuerdo al nivel educativo, es en el nivel de educación secundaria con 16.2% y de superior universitaria con 12%, en los cuales se observan las mayores prevalencias. Indicando una relación clara entre el acceso al consumo y el nivel educativo. Empero, este es un aspecto importante a tener en cuenta, respecto del efecto de los incrementos del Impuesto Selectivo al Consumo de Cigarrillos y productos derivados del tabaco, en el sentido de que al ser un impuesto ad valoren (sobre el precio) y de tarifa plana, genera un efecto regresivo deseable sobre los grupos de menor ingreso y por ende de menor nivel educativo. De igual manera queda manifiesta la tendencia a una mayor prevalencia de consumo en hombres que en mujeres.

Tabla N° 9

Prevalencia de Fumador Según Nivel Educativo y Sexo

Nivel educativo y sexo	Total	%	Condición de fumador			
			Fuma	%	No fuma	%
**TOTAL………**	**1,893**	**100.0**	**281**	**14.8**	**1,612**	**85.2**
Hombre	1,110	58.6	230	20.7	880	79.3
Mujer	783	41.4	51	6.5	732	93.5
**Ningún grado**	**23**	**1.2**	**5**	**21.7**	**18**	**78.3**
Hombre	16	0.8	4	25.0	12	75.0
Mujer	7	0.4	1	14.3	6	85.7
**Primaria**	**242**	**12.8**	**28**	**11.6**	**214**	**88.4**
Hombre	159	8.4	26	16.4	133	83.6
Mujer	83	4.4	2	2.4	81	97.6
**Vocacional**	**89**	**4.7**	**20**	**22.5**	**69**	**77.5**
Hombre	57	3.0	15	26.3	42	73.7
Mujer	32	1.7	5	15.6	27	84.4
**Secundaria**	**1,046**	**55.3**	**169**	**16.2**	**877**	**83.8**
Hombre	630	33.3	137	21.7	493	78.3
Mujer	416	22.0	32	7.7	384	92.3
**Superior Universitaria**	**493**	**26.0**	**59**	**12.0**	**434**	**88.0**
Hombre	248	13.1	48	19.4	200	80.6
Mujer	245	12.9	11	4.5	234	95.5

Fuente: Encuesta de marcas 2012.

No obstante, siempre queda como un elemento polémico el posible acceso al contrabando de los grupos de menor renta y este aspecto será abordado más adelante cuando se incorporen los cuadros de marcas legales y no legales más consumidas.

Por otro lado, respecto de la condición laboral, aquellos que trabajan son los que tienen la mayor prevalencia, con 16.9%, siendo mayor la de los hombre (21.9%), respecto de las mujeres (7.1%).

Tabla N° 10

Prevalencia de Fumador Según Condición Laboral y Sexo

Condición laboral y sexo	Total	%	Condición de fumador			
			Fuma	%	No fuma	%
**TOTAL.........**	**1,893**	**100.0**	**281**	**14.8**	**1,612**	**85.2**
Hombre	1,110	58.6	230	20.7	880	79.3
Mujer	783	41.4	51	6.5	732	93.5
**Trabaja**	**1,252**	**66.1**	**212**	**16.9**	**1,040**	**83.1**
Hombre	832	44.0	182	21.9	650	78.1
Mujer	420	22.2	30	7.1	390	92.9
**No trabaja**	**641**	**33.9**	**69**	**10.8**	**572**	**89.2**
Hombre	278	14.7	48	17.3	230	82.7
Mujer	363	19.2	21	5.8	342	94.2

Fuente: Encuesta de marcas 2012.

Mientras que quienes declararon que no trabajaban, la misma resultó ser del 10.8%. Para los hombres fue de 17.3% y las mujeres con 5.8%. Ello deja en claro que el poder adquisitivo ligado a la asequibilidad sustenta el uso de medidas fiscales de aumento del ISC, tal como se ha venido planteando en el presente y a futuro, de acuerdo a los análisis de la Curva de Laffer sustentada en las elasticidades precio e ingreso de la demanda.

Conocimiento de la ley antitabaco

Un aspecto relevante con relación a las características de la demanda de tabaco, es el conocimiento de la ley antitabaco y las actitudes y percepciones de los mismos frente a dicha norma.

En la tabla siguiente (N° 11) se observa la distribución de los entrevistados con relación al conocimiento de la ley antitabaco, y en general solo el 46% declaró tener conocimiento de la ley, mientras que el 54% restante no.

De los que dijeron conocer la ley, el 28.8% son hombres y el 12.2% son mujeres (para totalizar el 46%), mientras que quienes declararon no conocerla, 29.8%son hombres y el 24.1% son mujeres (para totalizar el 54%).

Al observar los resultados por distrito, se evidencia una clara tendencia respecto a un bajo conocimiento de la ley, en Barú con apenas el 3.7%, Colón con el 7.9%, David con 11.9%, Panamá con 11.8 y San Miguelito con 10.7%. Los hombres son los que marginalmente tienen algo más de conocimiento de la ley. Ello implica la necesidad de continuar profundizando los programas de divulgación sobre cada uno de sus componentes en todo el país.

Al considerar el conocimiento de la ley por nivel educativo, en términos generales del 46% que señaló tener conocimiento de la ley, solo quienes tienen educación secundaria y universitaria, tuvieron los porcentajes más altos con el 23.4% y 14.6% respectivamente. Sin embargo, como ya se observó también tienen las prevalencias de tabaquismo más altas.

De igual forma son los hombres los que en términos generales tienen mayor conocimiento con el 15.2% y el 8% respecto de estos dos niveles educativos, mientras que las mujeres tuvieron el 8.2% y 6.6% respectivamente.

Tabla N° 11

Total de Encuestados por Distrito Según Conocimiento de la Ley Antitabaco y Sexo

Conocimiento de la ley antitabaco	Total	Distrito				
		Barú	Colón	David	Panamá	San Miguelito
**TOTAL……….**	**1,893**	**124**	**471**	**350**	**471**	**477**
Porcentaje	100.0	6.6	24.9	18.5	24.9	25.2
**Conoce**	**871**	**70**	**149**	**226**	**223**	**203**
Porcentaje	46.0	3.7	7.9	11.9	11.8	10.7
**Sexo**						
Hombre	545	56	94	169	130	96
Porcentaje	28.8	3.0	5.0	8.9	6.9	5.1
Mujer	326	14	55	57	93	107
Porcentaje	17.2	0.7	2.9	3.0	4.9	5.7
**No conoce**	**1,022**	**54**	**322**	**124**	**248**	**274**
Porcentaje	54.0	2.9	17.0	6.6	13.1	14.5
**Sexo**						
Hombre	565	34	185	83	137	126
Porcentaje	29.8	1.8	9.8	4.4	7.2	6.7
Mujer	457	20	137	41	111	148
Porcentaje	24.1	1.1	7.2	2.2	5.9	7.8

Tabla N° 12

Distribución Porcentual de Encuestados por Conocimiento de la Ley Antitabaco y Sexo, Según Distrito y Nivel Educativo

Distrito y nivel educativo	Total	Conocimiento de la ley antitabaco					
		Conoce			No conoce		
		Total	Sexo		Total	Sexo	
			Hombre	Mujer		Hombre	Mujer
**TOTAL……….**	**100.0**	**46.0**	**28.8**	**17.2**	**54.0**	**29.8**	**24.1**
Ningún grado	1.2	0.4	0.3	0.1	0.8	0.5	0.3
Primaria	12.8	5.7	4.1	1.5	7.1	4.3	2.9
Vocacional	4.7	2.0	1.1	0.8	2.7	1.9	0.8
Secundaria	55.3	23.4	15.2	8.2	31.9	18.1	13.8
Universitaria	26.0	14.6	8.0	6.6	11.5	5.1	6.4
**Barú**	**100.0**	**56.5**	**45.2**	**11.3**	**43.5**	**27.4**	**16.1**
Ningún grado	1.6	0.8	0.8	0.0	0.8	0.8	0.0
Primaria	22.6	13.7	12.1	1.6	8.9	6.5	2.4
Vocacional	1.6	1.6	1.6	0.0	0.0	0.0	0.0
Secundaria	51.6	26.6	20.2	6.5	25.0	16.1	8.9
Universitaria	22.6	13.7	10.5	3.2	8.9	4.0	4.8
**Colón**	**100.0**	**31.6**	**20.0**	**11.7**	**68.4**	**39.3**	**29.1**
Ningún grado	1.5	0.6	0.4	0.2	0.8	0.4	0.4
Primaria	13.8	3.8	2.3	1.5	10.0	5.3	4.7
Vocacional	54.1	14.9	10.4	4.5	39.3	24.8	14.4
Secundaria	22.3	8.5	4.7	3.8	13.8	6.4	7.4
Universitaria	8.3	3.8	2.1	1.7	4.5	2.3	2.1
**David**	**100.0**	**64.6**	**48.3**	**16.3**	**35.4**	**23.7**	**11.7**
Ningún grado	1.7	0.3	0.3	0.0	1.4	1.4	0.0
Primaria	16.0	10.6	8.9	1.7	5.4	3.4	2.0
Vocacional	2.3	1.4	1.4	0.0	0.9	0.9	0.0
Secundaria	50.6	30.3	24.6	5.7	20.3	14.3	6.0
Universitaria	29.4	22.0	13.1	8.9	7.4	3.7	3.7
**Panamá**	**100.0**	**47.3**	**27.6**	**19.7**	**52.7**	**29.1**	**23.6**
Ningún grado	1.3	0.4	0.2	0.2	0.8	0.4	0.4
Primaria	8.9	3.2	2.3	0.8	5.7	4.5	1.3
Vocacional	2.5	0.6	0.4	0.2	1.9	1.7	0.2
Secundaria	55.8	25.7	15.5	10.2	30.1	17.0	13.2
Universitaria	31.4	17.4	9.1	8.3	14.0	5.5	8.5
**San Miguelito**	**100.0**	**42.6**	**20.1**	**22.4**	**57.4**	**26.4**	**31.0**
Ningún grado	0.4	0.2	0.2	0.0	0.2	0.0	0.2
Primaria	10.7	4.2	2.1	2.1	6.5	3.1	3.4
Vocacional	5.9	1.9	0.4	1.5	4.0	2.9	1.0
Secundaria	60.2	23.7	11.5	12.2	36.5	15.7	20.8
Universitaria	22.9	12.6	5.9	6.7	10.3	4.6	5.7

Al observar los resultados por distrito, las tendencias del conocimiento de la ley se mantienen en Barú, David, Panamá y San Miguelito, no obstante en Colón, aquellos con nivel de educación vocacional señalaran tener más conocimiento con el 14.9% y también merece destacar en el caso de Barú, que quienes tienen educación primaria representaron el 13.7%.

Lo que estos resultados parciales reflejan, es la necesidad de fortalecer el conocimiento de la ley antitabaco en todos los niveles educativos, pero especialmente en la educación básica, para concientizar a los más jóvenes que son los que se constituyen en los grupos más vulnerables del tabaquismo.

Los resultados de la tabla siguiente, son prueba de lo mencionado, el grupo etario de 15 a 19 años, solo representó el 1.6% de quienes señalaron conocer la ley antitabaco, mientras que entre los que se ubican entre los 25 y 39 años, están los porcentajes más altos entre el 6% y 5%, lo cual es relativamente bajo. De 40 a 65 años y más, se ubican los que estarían en una situación conocimiento intermedio, entre el 3% y 4%.

Tabla N° 13

Distribución Porcentual de Encuestados por Conocimiento de la Ley Antitabaco y Sexo, Según Distrito y Grupos de Edad

Distrito y grupos de edad	Total	Conocimiento de la ley antitabaco					
		Conoce			No conoce		
		Total	Hombre	Mujer	Total	Hombre	Mujer
**TOTAL.........**	**100.0**	**871**	**545**	**326**	**1,022**	**565**	**457**
15 – 19	6.7	1.6	0.7	1.0	5.1	2.9	2.2
20 – 24	11.8	4.9	2.2	2.7	6.9	2.7	4.2
25 – 29	14.5	6.6	3.3	3.3	7.9	4.2	3.8
30 – 34	11.7	5.1	2.7	2.4	6.6	3.4	3.2
35 – 39	10.3	5.2	3.5	1.7	5.1	2.4	2.7
40 – 44	10.0	4.6	3.3	1.4	5.3	3.3	2.1
45 – 49	8.8	4.3	3.2	1.1	4.5	3.1	1.4
50 – 54	6.7	3.8	2.4	1.4	2.9	1.5	1.4
55 – 59	5.8	3.1	2.1	1.0	2.6	1.8	0.8
60 – 64	5.7	3.1	2.4	0.7	2.5	1.4	1.2
65 y más	8.1	3.6	3.0	0.6	4.5	3.2	1.3
**Barú**	**100.0**	**56.5**	**45.2**	**11.3**	**43.5**	**27.4**	**16.1**
15 – 19	4.0	0.8	0.0	0.8	3.2	1.6	1.6
20 – 24	4.8	3.2	2.4	0.8	1.6	0.0	1.6
25 – 29	11.3	4.8	2.4	2.4	6.5	4.8	1.6
30 – 34	6.5	3.2	1.6	1.6	3.2	1.6	1.6
35 – 39	16.9	7.3	6.5	0.8	9.7	5.6	4.0
40 – 44	10.5	7.3	4.8	2.4	3.2	2.4	0.8
45 – 49	8.9	4.8	4.8	0.0	4.0	1.6	2.4

Distrito y grupos de edad	Total	Conocimiento de la ley antitabaco					
		Conoce			No conoce		
		Total	Hombre	Mujer	Total	Hombre	Mujer
50 – 54	8.9	4.8	4.8	0.0	4.0	3.2	0.8
55 – 59	5.6	4.0	3.2	0.8	1.6	1.6	0.0
60 – 64	12.9	10.5	8.9	1.6	2.4	2.4	0.0
65 y más	9.7	5.6	5.6	0.0	4.0	2.4	1.6
**Colón**	**100.0**	**31.6**	**20.0**	**11.7**	**68.4**	**39.3**	**29.1**
15 – 19	4.2	0.8	0.6	0.2	3.4	2.3	1.1
20 – 24	8.3	1.1	0.4	0.6	7.2	3.0	4.2
25 – 29	13.2	4.7	2.3	2.3	8.5	5.1	3.4
30 – 34	10.0	3.0	2.3	0.6	7.0	4.0	3.0
35 – 39	8.5	2.8	1.3	1.5	5.7	2.8	3.0
40 – 44	11.0	3.4	2.1	1.3	7.6	4.2	3.4
45 – 49	10.8	3.6	2.3	1.3	7.2	5.5	1.7
50 – 54	7.6	3.0	1.5	1.5	4.7	2.3	2.3
55 – 59	6.6	2.3	1.7	0.6	4.2	2.1	2.1
60 – 64	7.9	3.0	2.1	0.8	4.9	3.0	1.9
65 y más	11.9	4.0	3.2	0.8	7.9	4.9	3.0
**David**	**100.0**	**64.6**	**48.3**	**16.3**	**35.4**	**23.7**	**11.7**
15 – 19	5.4	2.0	2.0	0.0	3.4	2.0	1.4
20 – 24	11.1	6.9	3.4	3.4	4.3	2.9	1.4
25 – 29	12.3	8.9	5.4	3.4	3.4	2.0	1.4
30 – 34	10.9	6.6	4.6	2.0	4.3	2.9	1.4
35 – 39	11.1	8.9	7.1	1.7	2.3	1.7	0.6
40 – 44	14.0	8.3	5.7	2.6	5.7	4.3	1.4
45 – 49	9.4	5.7	4.9	0.9	3.7	2.3	1.4
50 – 54	6.9	4.6	3.7	0.9	2.3	1.4	0.9
55 – 59	7.1	5.7	4.6	1.1	1.4	1.1	0.3
60 – 64	6.0	4.3	4.3	0.0	1.7	0.6	1.1
65 y más	5.7	2.9	2.6	0.3	2.9	2.6	0.3
**Panamá**	**100.0**	**47.3**	**27.6**	**19.7**	**52.7**	**29.1**	**23.6**
15 – 19	7.4	1.9	0.2	1.7	5.5	2.8	2.8
20 – 24	13.8	4.9	1.7	3.2	8.9	3.0	5.9
25 – 29	14.4	6.6	3.2	3.4	7.9	4.2	3.6
30 – 34	12.3	5.7	2.5	3.2	6.6	3.6	3.0
35 – 39	10.4	5.9	4.2	1.7	4.5	1.7	2.8
40 – 44	8.1	3.8	3.2	0.6	4.2	2.8	1.5
45 – 49	8.9	4.9	3.6	1.3	4.0	3.2	0.8
50 – 54	5.9	4.5	2.5	1.9	1.5	0.4	1.1
55 – 59	6.6	3.0	1.3	1.7	3.6	3.0	0.6
60 – 64	4.5	2.1	1.5	0.6	2.3	1.1	1.3
65 y más	7.6	4.0	3.6	0.4	3.6	3.4	0.2
**San Miguelito**	**100.0**	**42.6**	**20.1**	**22.4**	**57.4**	**26.4**	**31.0**
15 – 19	10.1	2.1	0.4	1.7	8.0	4.4	3.6

Distrito y grupos de edad	Total	Conocimiento de la ley antitabaco					
		Conoce			No conoce		
		Total	Hombre	Mujer	Total	Hombre	Mujer
20 – 24	15.7	7.8	3.6	4.2	8.0	2.9	5.0
25 – 29	18.2	7.1	2.9	4.2	11.1	4.6	6.5
30 – 34	14.9	6.1	2.3	3.8	8.8	3.6	5.2
35 – 39	9.6	3.8	1.7	2.1	5.9	2.3	3.6
40 – 44	7.8	3.4	2.3	1.0	4.4	2.3	2.1
45 – 49	6.3	3.4	2.1	1.3	2.9	1.5	1.5
50 – 54	5.7	2.9	1.5	1.5	2.7	1.5	1.3
55 – 59	3.1	1.9	1.3	0.6	1.3	1.0	0.2
60 – 64	2.5	1.5	0.4	1.0	1.0	0.4	0.6
65 y más	6.1	2.7	1.7	1.0	3.4	1.9	1.5

De igual manera al observar la distribución de edades por sexo, los hombres suelen tener un conocimiento relativamente mayor a las mujeres, salvo en los tramos de 15 a 19 años y de 20 a 24 años. También es dable señalar que las tendencias de menor conocimiento de la ley se mantienen para el mismo grupo etario, de 15 a 19 años, en todos los distritos. Ello revela la mayor vulnerabilidad en cuanto a su exposición al consumo de tabaco de segunda mano, lo cual hace redoblar esfuerzos en cuanto a la restricción de venta a menores de edad y conocimientos y actitudes en torno a las medidas de control de consumo de tabaco.

En general se ha dejado en evidencia el menor conocimiento de la ley antitabaco que han revelado los entrevistados en cada uno de los distritos considerados, con un porcentaje general apenas del 46%, mientras que el 54% restante declaró no tener conocimiento. Es importante seguir fortaleciendo los programas de divulgación, sobre todo con los grupos entre los 12 y 19 años y entre los 20 y 24 años, en adición a fortalecer los mismos en todos los niveles educativos, pero en especial en educación básica, es decir, hasta el noveno grado.

Comercio de tabaco

El comercio y consumo de tabaco tiene sus particularidades en Panamá. Como ya se ha mencionado actualmente ambas empresas tabacaleras son importadoras y distribuidoras, con un canal de comercialización corto.

Respecto al consumo, como es de esperar la demanda es inelástica entre los fumadores, razón por la cual las medidas fiscales tienen una efectividad parcial y deben ser complementadas con las medidas de control, respecto a los espacios libres de humo, publicidad y otras.

Uno de los primeros datos a analizar es el consumo semanal por unidad de cigarrillos. En la tabla a continuación se observa que las mayores frecuencias, son de 1 a 5, de 6 a 10 y de 21 a 40

cigarrillos, respectivamente y es cónsona con la tendencia registrada por distrito, mientras que el promedio de cigarrillos semanales fue de 32, es decir una cajetilla y media aproximadamente. En el caso de estos tres rangos señalados, representaría el consumo de casi dos cajetillas por semana, con el 58% de los 212 fumadores que respondieron a esta pregunta.

La mayor frecuencia de gasto (Tabla N° 15) se observa de B/. 1.00 a B/. 1.99 y de B/. 2.00 a B/. 2.99, señal de que tienen acceso a cigarrillos baratos ya sean de contrabando o falsificados, dado que con el último incremento del ISC a finales del año 2009, el precio promedio de las marcas industriales legales paso a ser B/. 3.63 y como se verá en las tablas siguientes, los precios de estas marcas están en general por encima de los B/. 4.00, lo cual conlleva a que un cigarrillo legal esté como mínimo entre B/. 0.20 y B/. 0.25 por unidad. Sin embargo, dadas las diferencias socioeconómicas regionales al igual que de los costos de distribución implicados, no se descarta que se vendan cigarrillos de contrabando o falsificados por encima de B/. 0.20 en adición a que los industriales también puedan variar en ese mismo sentido.

Tabla N° 14

Consumo Semanal Estimado para Fumadores por Unidad de Cigarrillos por Distrito

Consumo semanal	Distrito					
	Total	Barú	Colón	David	Panamá	San Miguelito
**TOTAL……….**	**281**	**10**	**71**	**70**	**64**	**66**
**Consumidor**	**212**	**8**	**49**	**53**	**53**	**49**
1 - 5	42	2	4	10	17	9
6 - 10	41	1	11	10	6	13
11 - 15	20	0	4	4	6	6
16 - 20	22	1	2	10	6	3
21 - 40	40	2	11	7	10	10
41 - 60	16	1	6	4	3	2
61 - 80	5	0	1	4	0	0
81 - 100	16	1	4	3	4	4
101 - 120	2	0	2	0	0	0
121 - 160	3	0	2	0	0	1
Más de 160	5	0	2	1	1	1
**NR**	**69**	**2**	**22**	**17**	**11**	**17**
**Promedio…..**	**32**	**31**	**46**	**30**	**25**	**29**

Tabla N° 15

Gasto Semanal Estimado para Fumadores por Unidad de Cigarrillos por Distrito

Gasto semanal en B/.	Distrito					
	Total	Barú	Colón	David	Panamá	San Miguelito
**TOTAL………**	**281**	**10**	**71**	**70**	**64**	**66**
**Consumidor (1)**	**151**	**5**	**36**	**36**	**33**	**41**
Menos de 1.00	17	1	1	1	11	3
1.00 - 1.99	34	1	9	8	2	14
2.00 - 2.99	20	0	6	6	6	2
3.00 - 3.99	18	0	6	3	3	6
4.00 - 499	10	0	2	3	4	1
5.00 - 599	10	0	1	4	2	3
6.00 - 9.99	17	0	3	6	2	6
10.00 - 19.99	14	2	4	2	2	4
20 y más	11	1	4	3	1	2
**NR**	**130**	**5**	**35**	**34**	**31**	**25**
**Promedio…..**	**6.22**	**10.14**	**8.35**	**6.03**	**3.46**	**6.24**

(1) El cálculo se basa en los fumadores que declaran las unidades de cigarrillos diarios y el precio correspondiente.

Los datos de la Tabla N° 16 revelan que si bien el precio promedio por unidad es de B/. 0.22, entre los B/. 0.05 y los B/.0.15 hay una frecuencia acumulada del 21%. Para los que pagan de B/. 0.20 a B/. 0.30 la frecuencia resulto de 39%, indicando los dos umbrales más importantes respecto de la adquisición de cigarrillos legales e ilegales.

Tabla N° 16

Pagos por Unidad de Cigarrillo Reportados por los Fumadores por Sexo

Pago por unidad en B/.	Sexo		
	Total	Hombre	Mujer
**TOTAL………**	281	230	51
0.05	16	15	1
0.10	22	19	3
0.15	22	19	3
0.20	14	9	5
0.25	79	59	20
0.30	16	12	4
0.35	7	7	0
0.40	5	5	0
0.50	7	5	2

Pago por unidad en B/.	Sexo		
	Total	Hombre	Mujer
0.60	2	2	0
No elige	91	78	13
**Promedio……..**	0.22	0.21	0.24

Estas son señales inequívocas del mercado respecto a que los consumidores están accediendo a cigarrillos ilegales y conlleva más adelante a un análisis más detallado respecto a la estimación del contrabando en el país. Este dato se complementa con los datos de la tabla siguiente, en la cual se observa que con relación al consumo de cajetillas, la frecuencia acumulada es de 30.4% para los que pagan entre B/. 1.00 y B/. 2.75.

La adquisición de cigarrillos por cajetilla muy por debajo de los B/. 3.00 es un claro indicio de cigarrillos ilegales, pero de igual forma es importante tener en cuenta que los fumadores acceden a ambos mercados, es decir, pueden adquirir tanto cigarrillos legales como ilegales, por consiguiente en muchos casos los precios revelados sean una mezcla de ambos.

Tabla N° 17

Pagos por Unidad de Cajetilla Reportados por los Fumadores por Sexo

**Pago por cajetilla en B/.**	**Sexo**		
	**Total**	**Hombre**	**Mujer**
**TOTAL……….**	281	230	51
**Consumidor**	**233**	**193**	**40**
1.00 - 1.25	20	14	6
1.50 - 1.75	20	18	2
2.00 - 2.25	14	13	1
2.50 - 2.75	17	13	4
3.00 - 3.25	34	26	8
3.50 - 3.75	51	44	7
4.00 - 4.25	33	30	3
4.50 - 4.75	34	29	5
5.00 y más	10	6	4
**No elige**	**48**	**37**	**11**
**Promedio……..**	**3.25**	**3.25**	**3.20**

Empero, entre los B/. 3.50 y B/. 4.75 se obtuvo una frecuencia acumulada de 50.6%, indicando una mayor afluencia hacia los cigarrillos legales en principio. Empero, la estimación del contrabando dará mayores luces de la posible participación de mercado de estos productos entre los entrevistados y una aproximación en los distritos analizados.

En la tabla siguiente se presenta un mayor detalle del pago por cajetilla de cigarrillos industriales. Es de observar la variedad de precios que las distintas marcas presentan en cada distrito. En el distrito de Barú todas las marcas declaradas tienen un precio por encima de B/. 3.50, no obstante, hubo otras no declaradas con precios de B/. 2.63.

En Colón, si bien los fumadores entrevistados declararon precios de compra de mayores a B/. 3.50 llama la atención otros precios muy inferiores e incluso de B/. 1.00 a B/. 1.25 para marcas como Kool, L&M, Marlboro y Vicerroy lo cual deja el cálculo de los precios promedio por debajo de los B/. 3.00. En David en el caso de la marca Lucky Strike, se observa un precio promedio de B/. 1.50, al igual que del Marlboro entre B/. 1.00 y B/. 1.25 inferiores a B/. 3.50, y con un precio promedio cercano a los B/. 4.00. Con relación a la marca Vicerroy, la misma presentó un precio promedio de B/. 3.83. Respecto al distrito de Panamá, en la marca Kool también se observaron precios por debajo de B/. 3.50, y un promedio de B/. 3.56 por cajetilla. En la marca Marlboro se obtuvo un precio promedio de B/. 3.46 y en Vicerroy, un precio promedio de B/. 3.43. Finalmente en el distrito de San Miguelito en las marcas Kool, Marlboro y Vicerroy, también se reportaron precios inferiores a los B/. 2.00 y tener precios promedios de B/. 2.91, B/. 3.47 y B/. 3.58 respectivamente.

Los datos analizados dejan entrever que aún en las llamadas marcas industriales legales se pudieran estar vendiendo falsificaciones o bien contrabandeadas, dados los precios tan bajos reportados en todos los distritos para estas marcas que son tradicionales en el país. En las tablas 18 y 19 se puede observar con mayor detalle todos los precios revelados que respaldan estas apreciaciones.

Tabla N° 18

Pagos Efectuados por Cajetilla de Cigarrillos Industriales Según Distrito y Marca

Distrito y marca	Pagos efectuados (en Balboas)	
	Unidad	Cantidad
**TOTAL……….**		**281**
**Barú**		**10**
**Elige**	**3.65**	**9**
**Kool**	**3.71**	**1**
	3.50 - 375	1
**Marboro**	**4.03**	**3**
	3.50 - 3.75	1
	3.50 - 375	1
	4.50 - 4.75	1
**Vicerroy**	**3.92**	**4**
	3.00 - 3.25	1
	4.00 - 4.25	1
	4.50 - 4.75	1

Distrito y marca	Pagos efectuados (en Balboas)	
	Unidad	Cantidad
	NR	1
**Otros**	**2.63**	**2**
	2.50 - 2.75	2
**Colón**		**71**
**Elige**	**2.78**	**56**
**Kool**	**2.94**	**49**
	1.00 - 1.25	4
	1.50 - 1.75	1
	150 - 1.75	1
	2.00 - 2.25	7
	2.50 - 2.75	6
	3.00 - 3.25	10
	3.50 - 3.75	1
	4.00 - 4.25	2
	4.00 - 425	2
	4.50 - 4.75	4
	5.00 y más	3
	NR	8
**L&M**	**2**	**1**
	2.00 - 2.25	1
**Marboro**	**2.94**	**11**
	1.00 - 1.25	2
	2.00 - 2.25	2
	3.50 - 3.75	1
	3.50 - 375	1
	4.00 - 425	1
	4.50 - 4.75	1
	5.00 y más	1
	NR	2
**Vicerroy**	**1.00**	**1**
	1.00 - 1.25	1
**Otro**	**1.00**	**3**
	1.00 - 1.25	2
	NR	1
**No elige**	**1.80**	**6**
	1.00 - 1.25	1
	2.50 - 2.75	1
	NR	4
**David**		**70**

Distrito y marca	Pagos efectuados (en Balboas)	
	Unidad	Cantidad
**Elige**	**3.76**	**58**
**Kool**	**3.83**	**3**
	2.50 - 2.75	1
	4.50 - 4.75	2
**L&M**	**3.50**	**1**
	3.50 - 3.75	1
**Lucky Strike**	**1.50**	**2**
	1.50 - 1.75	1
	NR	1
**Marlboro**	**3.99**	**33**
	1.00 - 1.25	1
	1.50 - 1.75	1
	3.00 - 3.25	2
	3.50 - 3.75	2
	3.50 - 375	1
	4.00 - 4.25	9
	4.00 - 425	6
	4.50 - 4.75	9
	5.00 y más	1
	NR	1
**Vicerroy**	**3.83**	**25**
	1.50 - 1.75	1
	2.50 - 2.75	1
	3.00 - 3.25	3
	3.50 - 3.75	2
	3.50 - 375	5
	4.00 - 4.25	2
	4.00 - 425	1
	4.50 - 4.75	5
	5.00 y más	1
	NR	4
**No elige**	**1.83**	**6**
	1.50 - 1.75	2
	2.50 - 2.75	1
	NR	3
**Panamá**		**64**
**Elige**	**3.14**	**47**
**Kool**	**3.56**	**10**
	1.50 - 1.75	1
	3.00 - 3.25	2

Distrito y marca	Pagos efectuados (en Balboas)	
	Unidad	Cantidad
	3.50 - 375	3
	4.00 - 4.25	1
	4.50 - 4.75	2
	NR	1
**L&M**	**…**	**1**
	NR	1
**Lucky Strike**	**4.00**	**1**
	4.00 - 4.25	1
**Marboro**	**3.46**	**19**
	1.00 - 1.25	1
	3.00 - 3.25	5
	3.50 - 3.75	1
	3.50 - 375	3
	4.00 - 4.25	1
	4.00 - 425	1
	4.50 - 4.75	2
	NR	5
**Pall Mall**	**…**	**1**
	NR	1
**Phillip Morris**	**1.00**	**1**
	1.00 - 1.25	1
**Vicerroy**	**3.43**	**14**
	2.50 - 2.75	1
	3.00 - 3.25	2
	3.50 - 3.75	3
	3.50 - 375	4
	NR	4
**No elige**	**2.31**	**17**
	1.00 - 1.25	3
	150 - 1.75	1
	2.00 - 2.25	1
	3.50 - 375	2
	4.50 - 4.75	2
	NR	5
**San Miguelito**		**66**
**Elige**	**3.15**	**60**
**Kool**	**2.91**	**8**
	1.00 - 1.25	1
	1.50 - 1.75	1

Distrito y marca	Pagos efectuados (en Balboas)	
	Unidad	Cantidad
	3.00 - 3.25	4
	3.50 - 375	1
	4.00 - 4.25	1
**Lucky Strike**	**3.20**	**2**
	3.00 - 3.25	1
	3.50 - 375	1
**Marboro**	**3.47**	**22**
	1.00 - 1.25	1
	1.50 - 1.75	1
	2.00 - 2.25	1
	2.50 - 2.75	2
	3.00 - 3.25	3
	3.50 - 3.75	2
	3.50 - 375	5
	4.00 - 425	1
	4.50 - 4.75	2
	5.00 y más	3
	NR	1
**Vicerroy**	**3.58**	**23**
	1.00 - 1.25	1
	2.00 - 2.25	1
	2.50 - 2.75	1
	3.00 - 3.25	1
	3.50 - 3.75	6
	3.50 - 375	3
	4.00 - 4.25	2
	4.50 - 4.75	3
	5.00 y más	1
	No elige	4
**No elige**	**1.88**	**11**
	1.00 - 1.25	1
	1.50 - 1.75	6
	2.00 - 2.25	1
	2.50 - 2.75	1
	4.00 - 425	1
	NR	1

Tabla N° 19

Pagos Efectuados por Cajetilla de Cigarrillos Ilegales Según Marca

Marca	Pagos efectuados (en Balboas)

	Unidad	Cantidad
**TOTAL.........**	**2.54**	**281**
**Elige**	**0.21**	**79**
**Blitz**	**1.50**	**1**
	1.50 - 1.75	1
**Elephant**	**2.00**	**14**
	1.00 - 1.25	3
	1.50 - 1.75	1
	2.50 - 2.75	3
	3.50 - 375	1
	No elige pago	6
**Ibiza**	**2.53**	**10**
	1.50 - 1.75	1
	2.00 - 2.25	2
	2.50 - 2.75	4
	4.50 - 4.75	1
	No elige pago	2
**Jaisalmer (verde)**	**2.72**	**34**
	1.00 - 1.25	3
	1.50 - 1.75	9
	150 - 1.75	1
	2.00 - 2.25	1
	3.00 - 3.25	3
	3.50 - 3.75	4
	3.50 - 375	1
	4.00 - 4.25	1
	4.00 - 425	2
	4.50 - 4.75	3
	5.00 y más	1
	No elige pago	5
**Jaisalmer(rojo)**	**2.23**	**15**
	1.00 - 1.25	4
	1.50 - 1.75	2
	2.00 - 2.25	1
	3.00 - 3.25	1
	3.50 - 3.75	1
	3.50 - 375	1
	5.00 y más	1
	No elige pago	4
**Kentucky**	**4.50**	**1**
	4.50 - 4.75	1
**Silver**	**3.08**	4

Marca	Pagos efectuados (en Balboas)	
	Unidad	Cantidad
	1.50 - 1.75	1
	3.50 - 3.75	1
	4.00 - 4.25	1
	No elige pago	1
**No elige marca**	**3.48**	**202**
	1.00 - 1.25	10
	1.50 - 1.75	2
	150 - 1.75	2
	2.00 - 2.25	10
	2.50 - 2.75	10
	3.00 - 3.25	30
	3.50 - 3.75	14
	3.50 - 375	28
	4.00 - 4.25	18
	4.00 - 425	11
	4.50 - 4.75	29
	5.00 y más	8
	No elige pago	30

Esta situación deja entrever la venta falsificada o de contrabando de marcas industriales legales e incluso a precios por unidad similares a las marcas ilegales. En la tabla siguiente se presenta un resumen de los precios por unidad de las marcas ilegales reconocidas en el país, por no cumplir con los requisitos como por ejemplo, pictogramas y otros.

La venta ilegal está presente en todos los distritos considerados. En el caso de Barú, se reportó un precio promedio por unidad de B/. 0.23. En Colón la marca Elephant con B/. 0.18 por unidad y Jailsalmer (rojo) con B/. 0.17, fueron las marcas ilegales detectadas entre los fumadores y a precios mucho más asequibles, en cambio el Jailsalmer (verde) se ubica como la más cara con B/. 0.31. En David las marcas Blitz, Elephant e Ibiza aparecen como las marcas con precios por unidad más baratos, con B/. 0.10, B/. 0.13 y B/. 0.23 respectivamente. Cabe destacar que la marca Jailsermer (verde) aparece con un precio promedio de B/. 0.25 al nivel del precio de un cigarrillo industrial legal. Ello deja entrever que existen estrategias de venta en las cuales se unifica el precio de los cigarrillos ilegales con los legales, dejando posiblemente una percepción de similar calidad en el consumidor. Por otro lado, ello puede reflejar que de igual manera al subir el impuesto selectivo (ISC) sube de precio tanto el cigarrillo legal como el ilegal en algunos casos, pero los datos son limitados para poder confirmar esa hipótesis.

Con relación al distrito de Panamá, Ibiza con un precio promedio de B/. 0.15, Jailsalmer (verde) con B/. 0.19, y Jailsalmer (rojo) con B/. 0.20, se ubican como las marcas adquiridas por los informantes con el precio más asequible. La marca Silver con un precio promedio de B/. 0.25 es la más costosa y equiparable a los precios de los cigarrillos legales.

Finalmente en San Miguelito todas las marcas reportadas, Ibiza con B/. 0.29, Jailsalmer (verde) con B/. 0.23 y Jailsalmer (rojo) con B/. 0.23, se presentan como marcas con precios unitarios equiparables a los cigarrillos legales.

Estos resultados dejan evidencia de que en todos los distritos la comercialización de los cigarrillos ilegales se equipara a las de las marcas industriales legales con diferenciaciones de precios para capturar a fumadores con distintos niveles de ingresos, con lo cual son funcionales a la industria. Es difícil ubicar el origen de todas estas marcas y sus puntos de entrada, dado que en las visitas de campo se pudieron en todos los puntos de entrada de mercancía al país (Paso Canoa y Zona Libre de Colón). De manera muy informal se ha indagado con relación a las marcas Jailsalmer y Elephant y se ha determinado su posible origen de India y China, pero no es un dato certificado por autoridad competente.

Tabla N° 20

Pagos Efectuados por Unidad de Cigarrillos Ilegales Según Distrito y Marca

Distrito y marca	Pagos efectuados (en Balboas)	
	Unidad	Cantidad
**TOTAL……….**	**0.23**	**281**
**Consumidor**	**0.21**	**67**
**Barú**		**10**
**Elige**	**0.00**	**0**
**No elige**	**0.23**	**10**
	0.10	1
	0.25	4
	0.30	1
	NR	4
**Colón**		**71**
**Elige**	**0.18**	**18**
**Elephant**	**0.18**	**11**
	0.05	5
	0.10	1
	0.20	2
	0.25	1
	0.50	2
**Jaisalmer (verde)**	**0.31**	**2**

Distrito y marca	Pagos efectuados (en Balboas)	
	Unidad	Cantidad
	0.25	1
	0.40	1
**Jaisalmer(rojo)**	**0.17**	**5**
	0.05	2
	0.10	1
	0.15	2
**No elige**	**0.18**	**53**
	0.05	5
	0.10	4
	0.15	8
	0.20	6
	0.25	9
	0.50	1
	NR	20
**David**		**70**
**Elige**	**0.13**	**21**
**Blitz**	**0.10**	**1**
	0.10	1
**Elephant**	**0.13**	**2**
	0.10	1
	0.15	1
**Ibiza**	**0.23**	**2**
	0.20	1
	0.25	1
**Jaisalmer (verde)**	**0.25**	**4**
	0.15	1
	0.20	1
	0.25	1
	0.40	1
**Kentucky**	**0.25**	**1**
	0.25	1
**silver**	**0.23**	**3**
	0.20	1
	0.25	2
**No elige**	**0.27**	**57**
	0.20	2
	0.25	23
	0.30	5
	0.35	3
	0.40	1
	**NR**	**23**

Distrito y marca	Pagos efectuados (en Balboas)	
	**Unidad**	**Cantidad**
**Panamá**		**64**
**Elige**	**0.19**	**16**
**Elephant**	**…**	**1**
	NR	1
**Ibiza**		**3**
	0.15	1
	NR	2
**Jaisalmer (verde)**	**0.19**	**13**
	0.10	6
	0.15	2
	0.25	1
	0.40	1
	0.50	1
	NR	2
**Jaisalmer(rojo)**	**0.20**	**5**
	0.05	2
	0.50	1
	NR	2
**Silver**	**0.25**	**1**
	0.25	1
**No elige**	**0.25**	41
	0.05	2
	0.10	1
	0.15	4
	0.20	1
	0.25	10
	0.30	2
	0.35	1
	0.60	1
	NR	19
**San Miguelito**		**66**
**Elige**	**0.24**	**20**
**Ibiza**	**0.29**	**5**
	0.25	3
	0.40	1
	NR	1
**Jaisalmer (verde)**	**0.23**	**15**
	0.10	6
	0.25	2
	0.30	2

Distrito y marca	Pagos efectuados (en Balboas)	
	**Unidad**	**Cantidad**
	0.50	1
	0.60	1
	NR	3
**Jaisalmer(rojo)**	**0.23**	**5**
	0.15	1
	0.25	3
	NR	1
**No elige**	**0.27**	**41**
	0.15	2
	0.25	16
	0.30	6
	0.35	3
	0.50	1
	NR	13

En las tablas N° 21 y 22 se detallan los lugares de compra tanto de los cigarrillos legales como ilegales. Respecto de los cigarrillos ilegales la compra en abarroterías, bares, cantinas y puestos callejeros, son las principales vías de salida. No obstante, llama la atención que en Barú, Colón, David, Panamá y San Miguelito, se haya reportado la venta de ciertas marcas en supermercados, como por ejemplo, Ibiza, Jailsarmer (rojo y verde), Silver y Elephant.

Una posible explicación es que dada la restricción legal de no exhibir los cigarrillos en los puntos de venta, dado que los mismos deben estar ocultos a la vista del público, posiblemente sea un aliciente para vender también cigarrillos ilegales en los supermercados y mini súper. Esta situación hace considerar la necesidad de hacer requisas periódicas en los distintos puntos de venta para detectar este tipo de productos.

Tabla N° 21

Compras de Cigarrillos Reportadas de Marcas no Legales Según Distrito y Lugar de Compra

Distrito, marca y lugar de compra		Total
**TOTAL……….**		**281**
**Barú**		**10**
**No elige**		**10**
	Abarroterías	4

Distrito, marca y lugar de compra		Total
	Bares o cantinas	1
	Supermercados	5
**Colón**		**71**
**Elephant**		**11**
	Abarroterías	2
	Puesto de venta en la calle	7
	Una persona conocida	2
**Jaisalmer (verde)**		**2**
	Puesto de venta en la calle	1
	Supermercados	1
**Jaisalmer(rojo)**		**5**
	Puesto de venta en la calle	5
**No elige**		**53**
	Abarroterías	7
	Bares o cantinas	1
	Puesto de venta en la calle	23
	Supermercados	19
	Una persona conocida	2
	NR	1
**David**		**70**
**Blitz**		**1**
	Una persona conocida	1
**Elephant**		**2**
	Supermercados	1
	Una persona conocida	1
**Ibiza**		**2**
	Abarroterías	1
	Puesto de venta en la calle	1
**Jaisalmer (verde)**		**4**
	Abarroterías	1
	Bares o cantinas	2
	Puesto de venta en la calle	1
**Kentucky**		**1**
	Abarroterías	1
**Silver**		**3**
	Abarroterías	1

Distrito, marca y lugar de compra		Total
	Puesto de venta en la calle	1
	Supermercados	1
**No elige**		**57**
	Abarroterías	13
	Bares o cantinas	3
	Puesto de venta en la calle	4
	Restaurantes u hoteles	1
	Supermercados	28
	Una persona conocida	5
	NR	3
**Panamá**		**64**
**Elephant**		**1**
	Abarroterías	1
**Ibiza**		**3**
	Abarroterías	1
	Puesto de venta en la calle	1
	Supermercados	1
**Jaisalmer (verde)**		**13**
	Abarroterías	2
	Puesto de venta en la calle	6
	Supermercados	2
	Una persona conocida	3
**Jaisalmer(rojo)**		**5**
	Abarroterías	1
	Puesto de venta en la calle	3
	Supermercados	1
**Silver**		**1**
	Puesto de venta en la calle	1
**No elige**		**41**
	Abarroterías	27
	Puesto de venta en la calle	5
	Supermercados	9
**San Miguelito**		**66**
**Ibiza**		**5**
	Abarroterías	3
	Supermercados	1
	Una persona conocida	1

Distrito, marca y lugar de compra		Total
**Jaisalmer (verde)**		**15**
	Abarroterías	5
	Puesto de venta en la calle	5
	Supermercados	4
	Una persona conocida	1
**Jaisalmer(rojo)**		**5**
	Abarroterías	3
	Puesto de venta en la calle	1
	Una persona conocida	1
**No elige**		**41**
	Abarroterías	20
	Puesto de venta en la calle	5
	Restaurantes u hoteles	1
	Supermercados	8
	Una persona conocida	7

Tabla N° 22

Compras de Cigarrillos Reportadas de Marcas Legales Según Distrito y Lugar de Compra

Distrito, marca y lugar de compra		Total
**TOTAL……….**		**281**
**Barú**		**10**
**Kool**		**1**
	Supermercados	1
**Marboro**		**3**
	Abarroterías	1
	Supermercados	2
**Vicerroy**		**4**
	Abarroterías	3
	Bares o cantinas	1
**Otro**		**2**
	Supermercados	2

Distrito, marca y lugar de compra			Total
**Colón**			**71**
	**Kool**		**49**
		Abarroterías	7
		Bares o cantinas	1
		Puesto de venta en la calle	26
		Supermercados	13
		Una persona conocida	2
	**L$M**		**1**
		Puesto de venta en la calle	1
	**Marboro**		**11**
		Abarroterías	2
		Puesto de venta en la calle	3
		Supermercados	6
	**Vicerroy**		**1**
		Puesto de venta en la calle	1
	**Otro**		**3**
		Puesto de venta en la calle	2
		Supermercados	1
	**No elige**		**6**
		Puesto de venta en la calle	3
		Una persona conocida	2
		NR	1
**David**			**70**
	**Kool**		**3**
		Supermercados	3
	**L$M**		**1**
		Una persona conocida	1
	**Lucky Strike**		**2**
		Abarroterías	1
		Puesto de venta en la calle	1
	**Marboro**		**33**
		Abarroterías	10
		Bares o cantinas	2
		Puesto de venta en la calle	2
		Supermercados	17
		Una persona conocida	2

Distrito, marca y lugar de compra			Total
	**Vicerroy**		**25**
		Abarroterías	5
		Bares o cantinas	3
		Puesto de venta en la calle	3
		Restaurantes u hoteles	1
		Supermercados	10
		Una persona conocida	3
	**No elige**		**6**
		Abarroterías	1
		Puesto de venta en la calle	1
		Una persona conocida	1
		NR	3
**Panamá**			**64**
	**Kool**		**10**
		Abarroterías	4
		Puesto de venta en la calle	3
		Supermercados	3
	**L$M**		**1**
		Puesto de venta en la calle	1
	**Lucky Strike**		**1**
		Abarroterías	1
	**Marboro**		**19**
		Abarroterías	14
		Puesto de venta en la calle	1
		Supermercados	4
	**Pall Mall**		**1**
		Puesto de venta en la calle	1
	**Phillip Morris**		**1**
		Abarroterías	1
	**Vicerroy**		**14**
		Abarroterías	9
		Puesto de venta en la calle	1
		Supermercados	4
	**No elige**		**17**
		Abarroterías	3
		Puesto de venta en la calle	9
		Supermercados	2
		Una persona conocida	3

Distrito, marca y lugar de compra		Total
**San Miguelito**		**66**
**Kool**		**8**
	Abarroterías	3
	Puesto de venta en la calle	2
	Supermercados	1
	Una persona conocida	2
**Lucky Strike**		**2**
	Abarroterías	1
	Supermercados	1
**Marboro**		**22**
	Abarroterías	12
	Puesto de venta en la calle	1
	Restaurantes u hoteles	1
	Supermercados	6
	Una persona conocida	2
**Vicerroy**		**23**
	Abarroterías	10
	Puesto de venta en la calle	4
	Supermercados	5
	Una persona conocida	4
**No elige**		**11**
	Abarroterías	5
	Puesto de venta en la calle	4
	Una persona conocida	2

De lo observado en ambas tablas (21 y 22) no queda duda que los cigarrillos ilegales se distribuyen en los mismos canales de los cigarrillos legales o viceversa. Ello obliga a reconsiderar algunas aspectos tales como la no exhibición de productos de tabaco por restricciones legales, lo que obliga a realizar requisas periódicas en puntos de venta más formales como por ejemplo los supermercados de acuerdo a lo revelado por los entrevistados.

Estimación del contrabando

Al analizar los resultados de la tabla siguiente se observa que las marcas declaradas como ilegales y expresamente de contrabando, en términos generales representa el 28.1% en términos

generales. No obstante, en los distritos de Panamá y San Miguelito, representa el 35.9% y 37.9% respectivamente, mientras que en Colón fue del 25.4% y en David del 18.6% respectivamente.

Esto implica que el mayor mercado se concentra en la provincia de Panamá y en especial en la región metropolitana como cabría esperar por ser la más desarrollada y de mayor renta por habitante en el ámbito nacional.

Tabla N° 23

Compras de Cigarrillos Reportadas de Marcas no Legales Según Distrito

Distrito y marca		Total	%
	**TOTAL………………………..**	**281**	**100.0**
**Elige**		**79**	**28.1**
	Blitz	1	0.4
	Elephant	14	5.0
	Ibiza	10	3.6
	Jaisalmer (verde)	34	12.1
	Jaisalmer(rojo)	15	5.3
	Kentucky	1	0.4
	silver	4	1.4
**No elige**		**202**	**71.9**
**Barú**		**10**	**100.0**
**Elige**	**…**	**0**	0.0
**No elige**		**10**	**100.0**
**Colón**		**71**	**100.0**
**Elige**		**18**	**25.4**
	Elephant	11	15.5
	Jaisalmer (verde)	2	2.8
	Jaisalmer(rojo)	5	7.0
**No elige**		**53**	**74.6**
**David**		**70**	**100.0**
**Elige**		**13**	**18.6**
	Blitz	1	1.4
	Elephant	2	2.9
	Ibiza	2	2.9
	Jaisalmer (verde)	4	5.7
	Kentucky	1	1.4
	silver	3	4.3
**No elige**		**57**	**81.4**
**Panamá**		**64**	**100.0**

Distrito y marca		Total	%
**Elige**		**23**	**35.9**
	Elephant	1	1.6
	Ibiza	3	4.7
	Jaisalmer (verde)	13	20.3
	Jaisalmer(rojo)	5	7.8
	silver	1	1.6
**No elige**		**41**	**64.1**
**San Miguelito**		**66**	**100.0**
**Elige**		**25**	**37.9**
	Ibiza	5	7.6
	Jaisalmer (verde)	15	22.7
	Jaisalmer(rojo)	5	7.6
**No elige**		**41**	**62.1**

Al no tener referentes anteriores de línea base, no es posible determinar si a raíz del incremento del ISC el contrabando de marcas ilegales haya aumentando en estos distritos o se mantenga relativamente sin cambios. No obstante, ello permite tener un marco de referencia de hasta donde este tipo de comercio ha podido penetrar en las principales ciudades del país en adición a la posible evasión fiscal subyacente pese a no ser una encuesta de ámbito nacional. Un dato que hubiese sido posible incorporar para contextualizar la medición del contrabando, fue haber contado con los datos de la Autoridad Nacional de Aduanas de Panamá, sin embargo, pese a que esta información se solicitó formalmente mediante nota hace un año, no se obtuvo respuesta.

A manera de conclusión parcial, se puede señalar que las prevalencias de fumadores en el país se mantienen cercanas a resultados de otras encuestas en años anteriores, y pese a su no comparabilidad metodológica, se observan prevalencias relativamente bajas en todos los distritos y en los resultados generales. Empero, el desconocimiento de la ley en especial de la población joven y con menor educación, sigue siendo un problema latente a ser atendido profundizando las campañas educativas al respecto. Por otro lado, la asequibilidad de cigarrillo ilegales y el uso de los mismos canales de distribución de los cigarrillos legales, pone estos productos al alcance de las personas dadas las estrategias de venta con diferenciación de precios y relativa calidad de producto. En este sentido se observa que es necesario continuar con futuros incrementos del ISC dado el efecto asequibilidad en precio, máxime cuando queda en evidencia que un tercio de las ventas posiblemente sea contrabando. Ello implica además fortalecer las acciones aduaneras en materia de control del contrabando para lo cual se deben utilizar los recursos establecidos por ley del ISC.

5.2. Encuesta de locales de atención al público

Con base en esta encuesta se obtuvo información sobre las percepciones de los dueños o del personal de los locales de atención al público como hoteles, pensiones, bares, cantinas, refresquerías, restaurantes, discotecas, respecto de la situación económica del país, de la empresa, efectos positivos o negativos de la ley antitabaco, actitudes con relación a restricciones de promociones y publicidad entre otros aspectos. Al igual que la encuesta de marcas (consumidores), la encuesta de locales se realizó en los distritos de Barú, David, Panamá, San Miguelito y Colón. A continuación se presentan algunos resultados generales, sin desmedro del informe específico de esta encuesta.

Análisis de percepciones

Esta encuesta se basó en una muestra de 930 locales de atención al público, según la distribución que se presenta en la tabla siguiente.

Económica

Tabla N° 24

Total de Locales Comerciales Visitados Según Tamaño de la Empresa y Actividad Comercial

Tamaño de empresa y actividad		Total	%
**TOTAL…………..……………………..……..**		**930**	**100.0**
**Microempresas**		**539**	**58.0**
	Servicio de alojamiento en hoteles	16	1.7
	Servicio de alojamiento en pensiones	15	1.6
	Servicios en bares y cantinas	80	8.6
	Servicios en refresquería	53	5.7
	Servicios en restaurantes	336	36.1
	Servicios en restaurantes de comida rápida	39	4.2
**Pequeñas empresas**		**101**	**10.9**
	Servicio de alojamiento en hoteles	7	0.8
	Servicio de alojamiento en pensiones	2	0.2
	Servicios en bares y cantinas	20	2.2
	Servicios en refresquería	3	0.3
	Servicios en restaurantes	51	5.5
	Servicios en restaurantes de comida rápida	18	1.9

Tamaño de empresa y actividad		Total	%
**Medianas empresas**		**24**	**2.6**
	Servicio de alojamiento en hoteles	2	0.2
	Servicios en bares y cantinas	8	0.9
	Servicios en refresquería	1	0.1
	Servicios en restaurantes	11	1.2
	Servicios en restaurantes de comida rápida	2	
**Grandes empresas**		**76**	**8.2**
	Servicio de alojamiento en hoteles	4	0.4
	Servicio de alojamiento en pensiones	1	0.1
	Servicios en bares y cantinas	19	2.0
	Servicios en refresquería	12	1.3
	Servicios en restaurantes	32	3.4
	Servicios en restaurantes de comida rápida	8	0.9
**NSI**		**190**	**20.4**
	Servicio de alojamiento en hoteles	13	1.4
	Servicio de alojamiento en pensiones	6	0.6
	Servicios en bares y cantinas	14	1.5
	Servicios en refresquería	11	1.2
	Servicios en restaurantes	134	14.4
	Servicios en restaurantes de comida rápida	12	1.3

Las microempresas representan el 58% y en un segundo lugar se ubican las pequeñas empresas con el 10.9%, de acuerdo a la estructura por tipo de empresa y al ingreso. Respecto de los diferentes tipos de locales, los bares y cantinas, representaron el 15.2% y los restaurantes el 60.6%, como los de mayor participación relativa y representando el 75.8% de todos los locales, indicando que las percepciones generales al igual que por distrito, están relacionadas en mayor medida a este tipo de establecimientos.

En la tabla siguiente se presenta la percepción de los dueños o del personal colaborador de los locales visitados, respecto de la situación económica del país. El 35.7% declaró que ha empeorado, 38.7% señaló que está igual y el 23% señaló que ha mejorado. Solo el 2.6% señalo no saber o no respondió.

Respecto a la situación económica se percibe como desmejorada. En Colón el 34.2% señaló que ha empeorado, en David, el 43.7%, en Panamá el 35.6%, y en San Miguelito, el 28.7%. En general algo más de un tercio de los entrevistados señalo que la situación económica del país ha empeorado.

Tabla N° 25

Percepción de la Situación Económica del País Reportada por los Entrevistados de los Locales Comerciales Visitados Según Distrito

Distrito		Total	%
**TOTAL……………………………………..**		**930**	**100.0**
	Ha mejorado	214	23.0
	Esta igual	360	38.7
	Ha empeorado	332	35.7
	NS / NR	24	2.6
**Colón (cabecera)**		**155**	**100.0**
	Ha mejorado	32	20.6
	Esta igual	62	40.0
	Ha empeorado	53	34.2
	NS / NR	8	5.2
**David**		**215**	**100.0**
	Ha mejorado	40	18.6
	Esta igual	77	35.8
	Ha empeorado	94	43.7
	NS / NR	4	1.9
Panamá		**351**	**100.0**
	Ha mejorado	84	23.9
	Esta igual	132	37.6
	Ha empeorado	125	35.6
	NS / NR	10	2.8
**San Miguelito**		**209**	**100.0**
	Ha mejorado	58	27.8
	Esta igual	89	42.6
	Ha empeorado	60	28.7
	NS / NR	2	1.0

Con relación a la percepción de la situación económica de la empresa, el 20.2% del total declaró que la misma ha empeorado, 49.2% mencionó que está igual y el 28.7% declaró que ha mejorado. Solo el 1.8% no supo o no respondió. Lo cual establece que se advierte todavía un clima positivo tanto con relación a la situación económica del país como de la empresa.

Respecto de las percepciones a esta pregunta por distrito se observa igualmente una tendencia más a considerarla como positiva. En Colón solo el 20.2% consideró que ha empeorado, en David el 21.9%, en Panamá, el 20.8% y en San Miguelito, el 17.2%.

Tabla N° 26

Percepción de la Situación Económica de la Empresa Reportada por los Entrevistados de los Locales Comerciales Visitados Según Distrito

Distrito		Total	%
**TOTAL……………………………………**		**930**	**100.0**
	Ha mejorado	267	28.7
	Esta igual	458	49.2
	Ha empeorado	188	20.2
	NS / NR	17	1.8
Colón (cabecera)		155	**100.0**
	Ha mejorado	39	25.2
	Esta igual	79	51.0
	Ha empeorado	32	20.6
	NS / NR	5	3.2
David		215	**100.0**
	Ha mejorado	60	27.9
	Esta igual	103	47.9
	Ha empeorado	47	21.9
	NS / NR	5	2.3
Panamá		351	**100.0**
	Ha mejorado	103	29.3
	Esta igual	170	48.4
	Ha empeorado	73	20.8
	NS / NR	5	1.4
San Miguelito		209	**100.0**
	Ha mejorado	65	31.1
	Esta igual	106	50.7
	Ha empeorado	36	17.2
	NS / NR	2	1.0

Impacto de la ley

Respecto a la percepción del impacto de la ley antitabaco, ha sido positiva, pues en general el 92.2% está de acuerdo con su aplicación y solo el 7.8% está en desacuerdo. En los restaurantes es donde se observo el mayor porcentaje de desacuerdo con el 5.7%.

Respecto de estas percepciones por distritos, en Colón, el 90.3% está de acuerdo y el 9.7% no. Mientras que en los restaurantes el rechazo fue del 8.4%. En el distrito de David la aceptación fue el 94.4% y el rechazo de 5.6%, en el caso de los restaurantes, el rechazo fue e 3.7%. En Panamá, el 91.2% se manifestó de acuerdo y el 8.8% en desacuerdo. Respecto de los restaurantes el rechazo fue del 5.7%. Finalmente en San Miguelito, la aceptación fue del 92.8% y en contra el 7.2%, teniendo los restaurantes un rechazo del 5.7% al igual que en el distrito de Panamá.

Tabla N° 27

Total de Locales Comerciales Visitados que Reportan Estar o no de Acuerdo con la Ley Según Distrito y Actividad Comercial

Distrito y actividad		Total	%	Acuerdo	%	Desacuerdo	%
	**TOTAL…………..………………….……..**	**930**	**100.0**	**857**	**92.2**	**73**	**7.8**
	Servicio de alojamiento en hoteles	42	4.5	41	4.4	1	0.1
	Servicio de alojamiento en pensiones	24	2.6	21	2.3	3	0.3
	Servicios en bares y cantinas	141	15.2	132	14.2	9	1.0
	Servicios en refresquería	80	8.6	75	8.1	5	0.5
	Servicios en restaurantes	564	60.6	511	54.9	53	5.7
	Servicios en restaurantes de comida rápida	79	8.5	77	8.3	2	0.2
**Colón (cabecera)**		**155**	**100.0**	**140**	**90.3**	**15**	**9.7**
	Servicio de alojamiento en hoteles	7	4.5	7	4.5	0	0.0
	Servicio de alojamiento en pensiones	4	2.6	4	2.6	0	0.0
	Servicios en bares y cantinas	31	20.0	29	18.7	2	1.3
	Servicios en refresquería	7	4.5	7	4.5	0	0.0
	Servicios en restaurantes	98	63.2	85	54.8	13	8.4
	Servicios en restaurantes de comida rápida	8	5.2	8	5.2	0	0.0
**David**		**215**	**100.0**	**203**	**94.4**	**12**	**5.6**
	Servicio de alojamiento en hoteles	11	5.1	11	5.1	0	0.0
	Servicio de alojamiento en pensiones	9	4.2	8	3.7	1	0.5
	Servicios en bares y cantinas	47	21.9	44	20.5	3	1.4
	Servicios en refresquería	22	10.2	22	10.2	0	0.0
	Servicios en restaurantes	110	51.2	102	47.4	8	3.7
	Servicios en restaurantes de comida rápida	16	7.4	16	7.4	0	0.0
**Panamá**		**351**	**100.0**	**320**	**91.2**	**31**	**8.8**

Distrito y actividad		Total	%	Acuerdo	%	Desacuerdo	%
	Servicio de alojamiento en hoteles	24	6.8	23	6.6	1	0.3
	Servicio de alojamiento en pensiones	11	3.1	9	2.6	2	0.6
	Servicios en bares y cantinas	40	11.4	37	10.5	3	0.9
	Servicios en refresquería	25	7.1	21	6.0	4	1.1
	Servicios en restaurantes	215	61.3	195	55.6	20	5.7
	Servicios en restaurantes de comida rápida	36	10.3	35	10.0	1	0.3
**San Miguelito**		**209**	**100.0**	**194**	**92.8**	**15**	**7.2**
	Servicios en bares y cantinas	23	11.0	22	10.5	1	0.5
	Servicios en refresquería	26	12.4	25	12.0	1	0.5
	Servicios en restaurantes	141	67.5	129	61.7	12	5.7
	Servicios en restaurantes de comida rápida	19	9.1	18	8.6	1	0.5

Estos resultados revelan que existe una mayor percepción de aceptación de la ley antitabaco en todos los establecimientos de atención al público.

Con relación al beneficio ambiental que ha podido proporcionar la ley, el 88.6% se manifestó a favor y el 9.2% en contra. El 2.2% declaró no saber o no respondió. Respecto a los distritos, en el de Panamá, es donde se observa la mayor aceptación respecto a los beneficios ambientales.

Tabla N° 28

Total de Locales Comerciales Visitados que Reportan lo Beneficioso o no de la Ley en Materia Ambiental Según Actividad Comercial

Distrito	Beneficio ambiental						
	Total	Si	%	No	%	NS / NR	%
**TOTAL………**	930	824	88.6	86	9.2	20	2.2
Colón (cabecera)	155	137	14.7	13	1.4	5	0.5
David	215	197	21.2	14	1.5	4	0.4
Panamá	351	295	31.7	46	4.9	10	1.1
San Miguelito	209	195	21.0	13	1.4	1	0.1

Con relación a las quejas de clientes por no permitir fumar, en general el 21.5% declaró haber recibido quejas de los clientes por no permitir fumar en el local. En el distrito de David es donde más se reportaron quejas con el 34%, lo cual refleja que un tercio de los clientes ha manifestado quejas en este sentido, y hace considerar reforzar la aplicación de la ley en cuanto a mantener ambientes libres de humo.

Tabla N° 29

Total de Locales Comerciales Visitaos que Reportan Quejas de los Clientes por no Permitir Fumar Según Distrito

Distrito	Total	Quejas por no permitir fumar			
		Si	%	No	%
**TOTAL……**	**930**	**200**	**21.5**	**730**	**78.5**
Colón (cabecera)	155	31	20.0	124	80.0
David	215	73	34.0	142	66.0
Panamá	351	76	21.7	275	78.3
San Miguelito	209	20	9.6	189	90.4

Efectos económicos de la ley sobre la empresa

En general el impacto sobre los ingresos de las empresas ha sido más positivo según se ha declarado. En general el 97.1% considera que ley no ha incidido en disminuir sus ingresos y solo un 2.9% indicó lo contrario. En el distrito de Colón fue donde se observó la percepción más negativa, con el 5.2% que declaró que la ley ha incidido en disminuir sus ingresos, mientras que en San Miguelito se registró la percepción más baja con apenas un 0.5%. Esto refleja un resultado positivo a favor de que la ley ha traído más beneficios económicos a las empresas.

Tabla N° 30

Total de Locales Comerciales Visitados que Reportan Haber Disminuido o no sus Ingresos Según porcentaje de disminución por Distrito

Distrito y porcentaje de disminución	Disminuido ingresos				
	Total	Si	%	No	%
**TOTAL……**	**930**	**27**	**2.9**	**903**	**97.1**
**Colón (cabecera)**	**155**	**8**	**5.2**	**147**	**94.8**
**Disminuye**	**8**	**8**	**5.2**	**0**	
Menos del 10 %	3	3	1.9	0	0.0
Del 11 al 20 %	2	2	1.3	0	0.0
Más del 40 %	1	1	0.6	0	0.0
NR	2	2	1.3	0	0.0
**No aplica**	**147**	**0**	**0.0**	**147**	**94.8**
**David**	**215**	**7**	**3.3**	**208**	**96.7**
**Disminuye**	**7**	**7**	**3.3**	**0**	0.0
Menos del 10 %	2	2	0.9	0	0.0
Del 11 al 20 %	2	2	0.9	0	0.0
Del 21 al 40 %	1	1	0.5	0	0.0

Distrito y porcentaje de disminución		Disminuido ingresos				
		Total	Si	%	No	%
	Más del 40 %	1	1	0.5	0	0.0
	NR	1	1	0.5	0	0.0
**No aplica**		**208**	**0**	**0**	**208**	**96.7**
**Panamá**		**351**	**11**	**3.1**	**340**	**96.9**
**Disminuye**		**11**	**11**	**3.1**	**0**	0.0
	Menos del 10 %	3	3	0.9	0	0.0
	Del 11 al 20 %	6	6	1.7	0	0.0
	Del 21 al 40 %	1	1	0.3	0	0.0
	Más del 40 %	1	1	0.3	0	0.0
**No aplica**		**340**	**0**	**0.0**	**340**	**96.9**
**San Miguelito**		**209**	**1**	**0.5**	**208**	**99.5**
**Disminuye**		**1**	**1**		**0**	0.0
	Menos del 10 %	1	1	0.5	0	0.0
**No aplica**		**208**	**0**	**0.0**	**208**	**99.5**

Respecto del posible impacto negativo en el empleo en dichos locales, en general las disminuciones de personal en la mayoría de los casos apenas pasa del 1% en casi todos los distritos, concentrando en reducciones de menos de cinco empleados, lo cual es congruente con el hecho de que la ley no ha incidido negativamente en los ingresos.

Tabla N° 31

Total de Locales Comerciales Visitados que Reportan Haber Disminuido o no el Personal según Porcentaje de Disminución por Distrito

Distrito	Total	Porcentaje de disminución			
		Menos de 5 empleados	De 5 a 10 empleados	Más de 15 empleados	NS / NR
**TOTAL……………**	**930**	**15**	**3**	**1**	**911**
**Colón (cabecera)**	**155**	**3**	**0**	**0**	**152**
Si	3	3	0	0	0
**%**	**1.9**	**1.9**	**0.0**	**0.0**	**0.0**
No	152	0	0	0	152
**David**	**215**	**3**	**0**	**0**	**212**
Si	5	3	0	0	2
**%**	**2.3**	**1.4**	**0.0**	**0.0**	**0.9**
No	210	0	0	0	210
**Panamá**	**351**	**9**	**3**	**1**	**338**

Distrito		Total	Porcentaje de disminución			
			Menos de 5 empleados	De 5 a 10 empleados	Más de 15 empleados	NS / NR
	Si	13	9	3	1	0
	**%**	**3.7**	**2.6**	**0.9**	**0.3**	**0.0**
	No	338	0	0	0	338
**San Miguelito**		209	0	0	0	209
	Si	0	0	0	0	0
	**%**	**0.0**	**0.0**	**0.0**	**0.0**	**0.0**
	No	209	0	0	0	209

Respecto a la afluencia de clientes, es dable señalar que no se obtuvo una definición a la luz de las respuestas obtenidas, ya que el 95% declaró no saber o no respondió. Cabe mencionar que esta pregunta resulta difícil de responder para los dueños de locales, sobre todo porque la situación económica del país ha sido muy estable en los últimos 6 años.

Tabla N° 32

Total de Locales Comerciales Visitados que Reportan la Situación de Afluencia de Clientes por Distrito

Situación de afluencia	Distrito				
	Total	Colón (cabecera)	David	Panamá	San Miguelito
**TOTAL………**	**930**	**155**	**215**	**351**	**209**
Ha mejorado	17	4	2	9	2
Ha empeorado	10	2	2	6	0
Sin cambios	21	6	9	6	0
NS / NR	882	143	202	330	207

Con relación a la habilitación de espacios para fumadores, apenas el 3.9% declaró haber habilitado algún espacio en el local para fumadores, en especial en restaurantes con el 2.0%. Esta situación también se percibió en todos los distritos, respecto de los restaurantes, teniendo el porcentaje más alto el de Panamá con el 2.6%.

Tabla N° 33

Total de Locales Comerciales Visitados que Reportan la Habilitación o no de Espacios para Fumadores Según Actividad Comercial por Distrito

Distrito y actividad comercial		Habilitar espacios					
		Total	%	Si	%	No	%
**TOTAL………**	**TOTAL……………………**	**930**	**100.0**	**36**	**3.9**	**894**	**96.1**
	Servicio de alojamiento en hoteles	42	4.5	7	0.8	35	3.8
	Servicio de alojamiento en pensiones	24	2.6	2	0.2	22	2.4
	Servicios en bares y cantinas	141	15.2	5	0.5	136	14.6
	Servicios en refresquería	80	8.6	2	0.2	78	8.4
	Servicios en restaurantes	564	60.6	19	2.0	545	58.6
	Servicios en restaurantes de comida rápida	79	8.5	1	0.1	78	8.4
Colón (cabecera)		155	100.0	2	1.3	153	98.7
	Servicio de alojamiento en hoteles	7	4.5	0	0.0	7	4.5
	Servicio de alojamiento en pensiones	4	2.6	0	0.0	4	2.6
	Servicios en bares y cantinas	31	20.0	0	0.0	31	20.0
	Servicios en refresquería	7	4.5	0	0.0	7	4.5
	Servicios en restaurantes	98	63.2	2	1.3	96	61.9
	Servicios en restaurantes de comida rápida	8	5.2	0	0.0	8	5.2
David		215	100.0	9	4.2	206	95.8
	Servicio de alojamiento en hoteles	11	5.1	0	0.0	11	5.1
	Servicio de alojamiento en pensiones	9	4.2	1	0.5	8	3.7
	Servicios en bares y cantinas	47	21.9	2	0.9	45	20.9
	Servicios en refresquería	22	10.2	1	0.5	21	9.8
	Servicios en restaurantes	110	51.2	4	1.9	106	49.3
	Servicios en restaurantes de comida rápida	16	7.4	1	0.5	15	7.0
Panamá		351	100.0	21	6.0	330	94.0
	Servicio de alojamiento en hoteles	24	6.8	7	2.0	17	4.8
	Servicio de alojamiento en pensiones	11	3.1	1	0.3	10	2.8
	Servicios en bares y cantinas	40	11.4	3	0.9	37	10.5
	Servicios en refresquería	25	7.1	1	0.3	24	6.8
	Servicios en restaurantes	215	61.3	9	2.6	206	58.7
	Servicios en restaurantes de comida rápida	36	10.3	0	0.0	36	10.3
San Miguelito		209	100.0	4	1.9	205	98.1
	Servicios en bares y cantinas	23	11.0	0	0.0	23	11.0
	Servicios en refresquería	26	12.4	0	0.0	26	12.4
	Servicios en restaurantes	141	67.5	4	1.9	137	65.6
	Servicios en restaurantes de comida rápida	19	9.1	0	0.0	19	9.1

Percepciones respecto a las advertencias sanitarias y promociones

Con relación al efecto del cigarrillo en la incidencia de cáncer (Tabla N° 34), el 88.3% se manifestó muy de acuerdo. Tendiendo los porcentajes más altos los distritos de David y San Miguelito con el 94.4% y 96.2% respectivamente.

Respecto de las restricciones (Tabla N° 35) a las promociones de cigarrillos es también clara la aceptación de las mismas en un 80% al sumar las respuestas muy de acuerdo y algo de acuerdo. Teniendo David y San Miguelito, la mayor aceptación con 85.6% y 85.2% respectivamente. Sin embargo, ello deja en claro que todavía hay que seguir reforzando la concientización con relación a estas medidas.

Tabla N° 34

Opinión de los Entrevistados con Referencia al Aviso de Advertencia de Cáncer Colocado en las Cajetillas de Cigarrillos por Distrito

Distrito	Total	Muy de acuerdo	Algo de acuerdo	Indiferente	Algo en desacuerdo	Muy en desacuerdo
**TOTAL………..**	**100.0**	**88.3**	**4.1**	**1.7**	**1.1**	**4.8**
Colón (cabecera)	100.0	77.4	7.1	5.2	3.9	6.5
David	100.0	94.9	2.3	0.9	0.0	1.9
Panamá	100.0	84.3	5.1	1.7	0.6	8.3
San Miguelito	100.0	96.2	1.9	0.0	1.0	1.0

Tabla N° 35

Opinión de los Entrevistados con Referencia a las Restricciones a las Promociones y Comunicación en Puntos de Ventas por Distrito

Distrito	Total	Muy de acuerdo	Algo de acuerdo	Indiferente	Algo en desacuerdo	Muy en desacuerdo
**TOTAL………..**	**100.0**	**68.5**	**11.4**	**8.2**	**2.6**	**9.4**
Colón (cabecera)	100.0	63.9	9.0	9.0	5.2	12.9
David	100.0	73.0	12.6	6.0	1.4	7.0
Panamá	100.0	64.7	11.7	8.0	2.6	13.1
San Miguelito	100.0	73.7	11.5	10.0	1.9	2.9

De los resultados generales analizados, se vislumbra que existe una percepción positiva de los dueños de locales o del personal de los mismos, sobre la situación económica del país al igual que de la empresa, en lo referente a la disminución de ingresos, reducción de personal y afluencia de clientes. De igual forma se percibe que han sido mayores los beneficios ofrecidos por la ley antitabaco que sus posibles perjuicios en materia económica y ambiental.

Es también mayor la aceptación de las advertencias sanitarias y la restricción en materia de promociones y publicidad en general, por lo que la percepción general y por distrito es que la ley antitabaco ha sido beneficiosa para la actividad económica en los locales de atención al público. Empero, quedan aún algunas áreas por reforzar y que deben seguir siendo sujeto del reforzamiento de las medidas de control, las cuales serán consideradas en la siguiente sección.

5.3. Implicaciones para futuras intervenciones en materia de control del consumo de tabaco

Después de hacer una descripción sucinta de los resultados de ambas encuestas, conviene considerar algunas intervenciones futuras en materia de prevención y control del tabaquismo. Con relación a la encuesta de marcas (consumidores), merece la pena continuar reforzando los programas de educación y prevención del consumo de tabaco en jóvenes, dado el relativamente alto desconocimiento de la ley anti tabáquica, sobre todo en el nivel de educación básica. Esta es la labor más importante en materia de prevención y concienciación contra el consumo de tabaco que permite neutralizar el efecto asequibilidad por un lado y de mantener bajas las prevalencias de tabaquismo de las nuevas generaciones. Con ello se alcanzaría mayores beneficios de una menor prevalencia de enfermedades relacionadas al tabaquismo y por consiguiente, una mayor reducción del gasto del sector público sanitario.

Por otro lado, dado que las marcas ilegales (contrabando y falsificadas), utilizan el mismo canal de distribución de las marcas legales, tanto en la venta formal como informal, es importante realizar operativos para detectar la venta ilegal, ante la sospecha de que dichas marcas pueden estarse ocultando en los establecimientos (supermercados) por el hecho de que los cigarrillos no pueden estar a la vista directa del público, lo cual puede haber facilitado mucho mas el acceso de las marcas ilegales al sector formal.

En materia fiscal se siguen justificando las alzas al ISC por el hecho que la venta de las marcas ilegales sigue las mismas estrategias de las marcas legales, respecto de aparejarse a los precios de las mismas, especialmente con relación a las marcas falsificadas legales o bien que el contrabando es también funcional a la industria. Por consiguiente el incremento del ISC también puede causar un efecto de incremento paralelo en las marcas ilegales, aunque en algunos casos sean relativamente más baratas que las legales.

Con relación a la encuesta de locales comerciales, si bien se perciben beneficios derivados de la aplicación de la ley antitabaco, es importante realizar inspecciones para detectar la habilitación temporal o permanente de espacios para fumadores, especialmente en los restaurantes, los cuales fueron el tipo de establecimiento en que se percibió una mayor tendencia en esta materia en adición a un mayor rechazo relativo a la aplicación de la ley antitabaco.

Finalmente es necesario continuar los programas de concienciación de no fumar en el hogar, espacio en que aún las políticas de control deben seguir penetrando, sobre todo para proteger a los jóvenes de su acceso al consumo de tabaco.

Conclusiones y recomendaciones en materia de Política Fiscal

Conclusiones

Ha quedado en evidencia que la demanda de cigarrillos en Panamá, es el principal producto de consumo de productos derivados del tabaco, ante la restructuración de una industria que se reconfiguró a finales de la década del noventa. Dentro de las estrategias de las empresas industriales productoras principales (Phillip Morris y British American Tobaco), las cuales constituyen un duopolio puro, se estableció que Panamá quedaría como un país importador de productos derivados del tabaco, donde los cigarrillos, constituyen el principal producto de consumo local.

No obstante, estos cambios estructurales determinarían la composición y dinámica del mercado panameño en dichos productos para las décadas siguientes, matizadas por medidas de intervención en materia de controles en el consumo de productos derivados del tabaco, como por ejemplo, advertencias sanitarias, restricciones en la publicidad, establecimiento de ambientes libres de humo, entre otras medidas, que tuvieron su impacto en el consumo desde mediados de la década del dos mil, luego de la adopción y ratificación del Convenio Marco de la OMS.

Respecto de las medidas fiscales, la más sobresaliente fue el incremento del ISC en noviembre de 2009 de 32.5% a 100%, y que tuvo su reflejo inmediato en los dos últimos años, con un incremento conspicuo en el precio y una caída más pronunciada en el consumo. El incremento en la recaudación fiscal del ISC se incrementó, al pasar de aproximadamente de B/. 12 millones en 2009 a B/. 22 millones en 2011, demostrando la efectividad de dicha medida, en reducir el consumo formal sin reducir la recaudación fiscal.

De igual forma queda demostrado que pese a ese impacto esperado de las medidas de política fiscal, sobre el consumo y la recaudación, el efecto asequibilidad neutraliza dichos resultados, debido al incremento en la renta por habitante, máxime por la pujanza económica que ha demostrado Panamá en las dos últimas décadas. Por consiguiente, no solo vasta considerar los incrementos del ISC per se, sino se toman en cuenta los incrementos en el poder adquisitivo de la población a mediano y largo plazo.

Desde esta perspectiva huelga considerar los resultados de los modelos propuestos con base en el Tool kit del Banco Mundial, en los que se ha podido demostrar que es posible obtener funciones de demanda dinámicas, que evidencien el comportamiento del consumo mediante el cálculo de las elasticidades precio e ingreso de la demanda de corto y largo plazo y que hipotéticamente permiten la construcción de la Curva de Laffer en la búsqueda de umbrales impositivos que no afecten negativamente la recaudación fiscal.

Si bien los resultados del modelo convencional mediante la corrección de auto correlación serial de primer orden, mediante la técnica de Cochrane-Orcutt, permitió obtener la mejor estimación

elasticidad precio de la demanda de largo plazo de -0.82, dejó un margen estrecho para incrementos del ISC al 12% anual, en parte debido al impacto del reciente aumento al 100%.

No obstante, estos resultados contribuyen a establecer claras perspectivas sobre el potencial de las políticas fiscales a corto plazo y revelan sobre todo que también en períodos cortos de tiempo pueden verse los efectos inmediatos. De igual forma se ha podido evidenciar que las elasticidades precio e ingreso de la demanda, cambian de manera significativa en el tiempo, por lo que su estimación econométrica y su uso en la construcción de la Curva de Laffer debe ser para modelar los impactos en la recaudación en períodos cortos, en adición a que el efecto de las medidas fiscales es de corta duración, como se ha podido observar con la medición de la asequibilidad. Es decir, que sigue existiendo un área de intervención en las que deben complementarse las medidas fiscales con las medidas de control.

De igual manera es importante seguir ajustando los modelos de corrección de errores, adicción miope y adicción racional para obtener estimaciones robustas, con miras a verificar estas hipótesis para obtener mediciones de corto y largo de las elasticidades precio e ingreso de la demanda, que evidencien la formación de expectativas de consumo, con miras a establecer otros escenarios para la Curva de Laffer con una serie de tiempo más larga y obtener coeficientes más robustos, con series de tiempo más largas.

Otro de los aspectos a considerar, es con relación al singo del coeficiente de elasticidad ingreso de la demanda, negativo y puede deberse a que Panamá tiene prevalencias de consumo de tabaco muy bajas como ha podido observarse, en parte como respuesta a la combinación de políticas.

Recomendaciones en materia de Política Fiscal

Luego de los análisis derivados de las estimaciones econométricas, se evidencia que la aplicación de nuevos incrementos del ISC es mucho más estrecho a corto plazo, por el fuerte impacto del último incremento del 100% sobre el precio, el consumo y la recaudación de dicho impuesto.

Frente a esta situación de es posible vislumbrar nuevos escenarios de incrementos del ISC al 12%, pero de igual manera, respecto de la neutralización de la asequibilidad con un incremento anual del 4.36%, derivado de [{(1+TCYP)(1+TIPT)-1}X100], refleja que aún existen posibilidades reales de seguir incrementando el ISC con miras a reducir la externalidad negativa del consumo de productos derivados del tabaco, especialmente sobre el sistema sanitario panameño.

No obstante, ante los altos costos de generar futuras iniciativas legislativas para tales fines y ante los nuevos escenarios políticos que se presenten, es dable considerar la aplicación de cláusulas gatillo o de aplicación instantánea que queden establecidas de manera permanente en la legislación fiscal antitabáquica panameña y ello puede establecerse sobre la base del comportamiento histórico de la inflación del precio del tabaco y del crecimiento de la renta por habitante.

De igual forma se hace necesaria una mayor colaboración de las autoridades fiscales y aduaneras en el manejo de información del contrabando por un lado y de las recaudaciones verificadas con relación a las ventas físicas, de tal forma que a futuro pueden obtenerse estimaciones de la demanda de cigarrillos y otros productos derivados del tabaco, más ajustadas a la realidad, permitiendo intervenciones mucho más efectivas en materia de política fiscal.

Finalmente es importante continuar reforzando los programas de educación sobre el conocimiento de la ley anti tabáquica, en especial en el nivel de educación básica y hacer operativos de venta de cigarrillos ilegales en establecimientos formales en adición a la detección de posibles espacios habilitados a fumadores en restaurantes.

La lucha para el control del tabaquismo, requiere de medidas permanentes de control y fiscales, de forma que se neutralice el efecto asequibilidad de cada nueva generación.

Bibliografía

Autoridad Nacional de Aduanas. Series históricas de decomisos anuales de cigarrillos, 2004-2011. Ministerio de Economía y Finanzas. Panamá, República de Panamá.

Belcher, Evan y Van Walbeek, Corné (2008), "An analysis of cigarrette affordability", *International Union Against Tuberculosis and Lung Disease*. Available from: http://www.ingentaconnect.com/content/iuatld/ Accessed: July 14th 2012.

Centro latinoamericano y caribeño de demografía. "Proyecciones de población-Panamá 1950-2050". Available from: http://www.eclac.org/celade/proyecciones/basedatos_BD.htm Accessded: March 8th 2012.

Decreto Ejecutivo N° 86. Por el cual se reglamentan las restricciones y disposiciones sobre la venta de cigarrillos y tabacos a los menores de edad y la impresión de advertencias sobre su uso. Gaceta Oficial N° 23,909. Panamá, República de Panamá, 2 de junio de 1999.

Decreto Ejecutivo N° 17. Por el cual se dictan medidas para la prevención y reducción del consumo de tabaco y exposición al humo de productos del tabaco, por sus efectos nocivos a la salud de la población. Gaceta Oficial Digital N°25262. Panamá, República de Panamá, 22 de marzo de 2005.

Decreto Ejecutivo N° 85. Por el cual se reglamenta el impuesto selectivo al consumo de ciertos bienes y servicios. Gaceta Oficial N° 25,373. Panamá, República de Panamá, 29 de agosto de 2005.

Decreto Ejecutivo N° 230. Que reglamenta la Ley N° 13 de enero de 2008 y dicta otras disposiciones. Gaceta Oficial Digital N° 26033. Panamá, República de Panamá, 6 de mayo de 2008.

Dirección General de Ingresos. Series históricas del Impuesto Selectivo al Consumo de cigarrillos y base impositiva 2000-2011. Ministerio de Economía y Finanzas. Panamá, República de Panamá.

Herrera, Víctor Hugo. Estudio de la demanda de Cigarrillos y Tabaco en Panamá. Ministerio de Salud. Available from: http://www.minsa.gob.pa/minsa/tl_files/documents/informacion_salud/control_tabaco/Estudio%20de%20la%20Demanda%20de%20Tabaco%20CIET-Panama-Abril-2010.pdf Accessed: May 20th 2012.

Instituto Nacional de Estadística y Censo. "Encuesta de Propósitos Múltiples". Marzo de 2006. Contraloría General de la República. Panamá, República de Panamá.

Instituto Nacional de Estadística y Censo. Series históricas del IPC-Total, IPC-Tabaco, Importaciones de productos derivados del tabaco, producción de cigarros, Producto Interno Bruto e Ingreso Nacional Disponible, 1999-2011. Contraloría General de la República. Panamá, República de Panamá.

Ministerio de Salud. "Las Políticas de Control de Tabaco: Una Mirada desde la Encuesta Mundial de Tabaco en Jóvenes", 2002 y 2008. Panamá, República de Panamá.

Ley N° 45. Que deroga los títulos VI y XXI del libro cuarto del código fiscal, los Decretos de gabinete 35 de 1970 y 22 de 1972, se modifican y derogan otras disposiciones, crea impuesto selectivo al consumo de bebidas gaseosas, alcohólicas. Gaceta Oficial N° 22,911. Panamá, República de Panamá, 15 de noviembre de 1995.

Ley N° 28. Que modifica el parágrafo 6 del artículo 1057-V del Código Fiscal sobre el impuesto al cigarrillo, el artículo 24 de la Ley 30 de 1984 sobre contrabando y defraudación aduanera, y dicta otras disposiciones. Gaceta Oficial N° 24,334. Panamá, República de Panamá, 29 de junio de 2001.

Ley N° 40. Por la cual se aprueba el convenio marco de la OMS para el control del tabaco, aprobado por la cuarta sesión plenaria de la organización mundial de la salud, el 21 de mayo de 2003. Gaceta Oficial Digital N° 25,097. Panamá, República de Panamá, 7 de julio de 2004.

Ley N° 13. Que adopta medidas para el control del tabaco y sus efectos nocivos para la salud. Gaceta Oficial Digital N° 25966. Panamá, República de Panamá, 25 de 2008.

Ley N° 49. Que reforma el Código Fiscal y adopta otras medidas fiscales. Gaceta Oficial Digital N° 26,370-C. Panamá, República de Panamá, 17 de septiembre de 2009.

Ley N° 69. Que reforma el Código Fiscal y adopta otras medidas fiscales. Gaceta Oficial Digital N° 26402-C. Panamá, República de Panamá, 6 de noviembre de 2009.

Resolución N° 471. Que Aprueba el Reglamento del Decreto Ejecutivo N° 17 de11 de marzo de 2005. Gaceta Oficial Digital N°25307. Panamá, República de Panamá, 26 de mayo de 2005.

Wilkins, Nick, Yurekli, Ayda, and Hu, Teh-wei (2012). "Economic Analysis of Tobacco Demand". *Economics of Tobacco Toolkit N° 3. World Bank*. Available from: http://web.worldbank.org/WBSITE/EXTERNAL/TOPICS/EXTHEALTHNUTRITIONANDPOPULATION/EXTETC/0,,contentMDK:20365047~menuPK:376607~pagePK:148956~piPK:216618~theSitePK:376601,00.html Accessded: June 12th 2012.

Anexos

1.

Simulación preliminar de la curva de Laffer con la elasticidad de -0.82

Pr (New)	Tax	Ps = pr*tax	Dt	ε_{pd}	Δp	Δc	C(New)	R(New)	ΔR(New)
3.630	100%	3.630	0%	-0.82	0.000	0.000	5,142,160.0	5,142,160.0	
3.666		3.666	1%		0.010	-0.008	5,099,994.3	18,698,109.1	13,555,949.1
3.703		3.703	2%		0.020	-0.016	5,057,828.6	18,727,116.1	13,584,956.1
3.739		3.739	3%		0.030	-0.025	5,015,662.9	18,753,061.9	13,610,901.9
3.775		3.775	4%		0.040	-0.033	4,973,497.2	18,775,946.4	13,633,786.4
3.812		3.812	5%		0.050	-0.041	4,931,331.4	18,795,769.8	13,653,609.8
3.848		3.848	6%		0.060	-0.049	4,889,165.7	18,812,531.9	13,670,371.9
3.884		3.884	7%		0.070	-0.057	4,847,000.0	18,826,232.8	13,684,072.8
3.920		3.920	8%		0.080	-0.066	4,804,834.3	18,836,872.4	13,694,712.4
3.957		3.957	9%		0.090	-0.074	4,762,668.6	18,844,450.8	13,702,290.8
3.993		3.993	10%		0.100	-0.082	4,720,502.9	18,848,968.0	13,706,808.0
4.029		4.029	11%		0.110	-0.090	4,678,337.2	18,850,424.0	13,708,264.0
4.066		4.066	12%		0.120	-0.098	4,636,171.5	18,848,818.7	13,706,658.7
4.102		4.102	13%		0.130	-0.107	4,594,005.7	18,844,152.2	13,701,992.2
4.138		4.138	14%		0.140	-0.115	4,551,840.0	18,836,424.4	13,694,264.4
4.175		4.175	15%		0.150	-0.123	4,509,674.3	18,825,635.4	13,683,475.4
4.211		4.211	16%		0.160	-0.131	4,467,508.6	18,811,785.2	13,669,625.2
4.247		4.247	17%		0.170	-0.139	4,425,342.9	18,794,873.8	13,652,713.8
4.283		4.283	18%		0.180	-0.148	4,383,177.2	18,774,901.1	13,632,741.1
4.320		4.320	19%		0.190	-0.156	4,341,011.5	18,751,867.3	13,609,707.3
4.356		4.356	20%		0.200	-0.164	4,298,845.8	18,725,772.1	13,583,612.1
4.392		4.392	21%		0.210	-0.172	4,256,680.0	18,696,615.8	13,554,455.8
4.429		4.429	22%		0.220	-0.180	4,214,514.3	18,664,398.2	13,522,238.2
4.465		4.465	23%		0.230	-0.189	4,172,348.6	18,629,119.4	13,486,959.4
4.501		4.501	24%		0.240	-0.197	4,130,182.9	18,590,779.3	13,448,619.3
4.538		4.538	25%		0.250	-0.205	4,088,017.2	18,549,378.0	13,407,218.0
4.574		4.574	26%		0.260	-0.213	4,045,851.5	18,504,915.5	13,362,755.5
4.610		4.610	27%		0.270	-0.221	4,003,685.8	18,457,391.8	13,315,231.8
4.646		4.646	28%		0.280	-0.230	3,961,520.1	18,406,806.8	13,264,646.8
4.683		4.683	29%		0.290	-0.238	3,919,354.4	18,353,160.6	13,211,000.6
4.719		4.719	30%		0.300	-0.246	3,877,188.6	18,296,453.2	13,154,293.2
4.755		4.755	31%		0.310	-0.254	3,835,022.9	18,236,684.5	13,094,524.5
4.792		4.792	32%		0.320	-0.262	3,792,857.2	18,173,854.6	13,031,694.6
4.828		4.828	33%		0.330	-0.271	3,750,691.5	18,107,963.5	12,965,803.5

Pr (New)	Tax	Ps = pr*tax	Dt	ε_{pd}	Δp	Δc	C(New)	R(New)	ΔR(New)
4.864		4.864	34%		0.340	-0.279	3,708,525.8	18,039,011.2	12,896,851.2
4.901		4.901	35%		0.350	-0.287	3,666,360.1	17,966,997.6	12,824,837.6
4.937		4.937	36%		0.360	-0.295	3,624,194.4	17,891,922.8	12,749,762.8
4.973		4.973	37%		0.370	-0.303	3,582,028.7	17,813,786.7	12,671,626.7
5.009		5.009	38%		0.380	-0.312	3,539,862.9	17,732,589.4	12,590,429.4
5.046		5.046	39%		0.390	-0.320	3,497,697.2	17,648,330.9	12,506,170.9
5.082		5.082	40%		0.400	-0.328	3,455,531.5	17,561,011.2	12,418,851.2
5.118		5.118	41%		0.410	-0.336	3,413,365.8	17,470,630.2	12,328,470.2
5.155		5.155	42%		0.420	-0.344	3,371,200.1	17,377,188.0	12,235,028.0
5.191		5.191	43%		0.430	-0.353	3,329,034.4	17,280,684.6	12,138,524.6
5.227		5.227	44%		0.440	-0.361	3,286,868.7	17,181,119.9	12,038,959.9
5.264		5.264	45%		0.450	-0.369	3,244,703.0	17,078,494.0	11,936,334.0
5.300		5.300	46%		0.460	-0.377	3,202,537.2	16,972,806.9	11,830,646.9
5.336		5.336	47%		0.470	-0.385	3,160,371.5	16,864,058.6	11,721,898.6
5.372		5.372	48%		0.480	-0.394	3,118,205.8	16,752,249.0	11,610,089.0
5.409		5.409	49%		0.490	-0.402	3,076,040.1	16,637,378.2	11,495,218.2
5.445		5.445	50%		0.500	-0.410	3,033,874.4	16,519,446.1	11,377,286.1
5.481		5.481	51%		0.510	-0.418	2,991,708.7	16,398,452.8	11,256,292.8
5.518		5.518	52%		0.520	-0.426	2,949,543.0	16,274,398.3	11,132,238.3
5.554		5.554	53%		0.530	-0.435	2,907,377.3	16,147,282.6	11,005,122.6
5.590		5.590	54%		0.540	-0.443	2,865,211.6	16,017,105.6	10,874,945.6
5.627		5.627	55%		0.550	-0.451	2,823,045.8	15,883,867.4	10,741,707.4
5.663		5.663	56%		0.560	-0.459	2,780,880.1	15,747,568.0	10,605,408.0
5.699		5.699	57%		0.570	-0.467	2,738,714.4	15,608,207.3	10,466,047.3
5.735		5.735	58%		0.580	-0.476	2,696,548.7	15,465,785.4	10,323,625.4
5.772		5.772	59%		0.590	-0.484	2,654,383.0	15,320,302.3	10,178,142.3
5.808		5.808	60%		0.600	-0.492	2,612,217.3	15,171,758.0	10,029,598.0
5.844		5.844	61%		0.610	-0.500	2,570,051.6	15,020,152.4	9,877,992.4
5.881		5.881	62%		0.620	-0.508	2,527,885.9	14,865,485.6	9,723,325.6
5.917		5.917	63%		0.630	-0.517	2,485,720.1	14,707,757.5	9,565,597.5
5.953		5.953	64%		0.640	-0.525	2,443,554.4	14,546,968.2	9,404,808.2
5.990		5.990	65%		0.650	-0.533	2,401,388.7	14,383,117.7	9,240,957.7
6.026		6.026	66%		0.660	-0.541	2,359,223.0	14,216,206.0	9,074,046.0
6.062		6.062	67%		0.670	-0.549	2,317,057.3	14,046,233.0	8,904,073.0
6.098		6.098	68%		0.680	-0.558	2,274,891.6	13,873,198.8	8,731,038.8
6.135		6.135	69%		0.690	-0.566	2,232,725.9	13,697,103.4	8,554,943.4
6.171		6.171	70%		0.700	-0.574	2,190,560.2	13,517,946.7	8,375,786.7
6.207		6.207	71%		0.710	-0.582	2,148,394.4	13,335,728.9	8,193,568.9
6.244		6.244	72%		0.720	-0.590	2,106,228.7	13,150,449.7	8,008,289.7

Pr (New)	Tax	Ps = pr*tax	Dt	ε_{pd}	Δp	Δc	C(New)	R(New)	ΔR(New)
6.280		6.280	73%		0.730	-0.599	2,064,063.0	12,962,109.4	7,819,949.4
6.316		6.316	74%		0.740	-0.607	2,021,897.3	12,770,707.8	7,628,547.8
6.353		6.353	75%		0.750	-0.615	1,979,731.6	12,576,245.0	7,434,085.0
6.389		6.389	76%		0.760	-0.623	1,937,565.9	12,378,720.9	7,236,560.9
6.425		6.425	77%		0.770	-0.631	1,895,400.2	12,178,135.7	7,035,975.7
6.461		6.461	78%		0.780	-0.640	1,853,234.5	11,974,489.2	6,832,329.2
6.498		6.498	79%		0.790	-0.648	1,811,068.8	11,767,781.4	6,625,621.4
6.534		6.534	80%		0.800	-0.656	1,768,903.0	11,558,012.5	6,415,852.5
6.570		6.570	81%		0.810	-0.664	1,726,737.3	11,345,182.3	6,203,022.3
6.607		6.607	82%		0.820	-0.672	1,684,571.6	11,129,290.8	5,987,130.8
6.643		6.643	83%		0.830	-0.681	1,642,405.9	10,910,338.2	5,768,178.2
6.679		6.679	84%		0.840	-0.689	1,600,240.2	10,688,324.3	5,546,164.3
6.716		6.716	85%		0.850	-0.697	1,558,074.5	10,463,249.2	5,321,089.2
6.752		6.752	86%		0.860	-0.705	1,515,908.8	10,235,112.8	5,092,952.8
6.788		6.788	87%		0.870	-0.713	1,473,743.1	10,003,915.2	4,861,755.2
6.824		6.824	88%		0.880	-0.722	1,431,577.3	9,769,656.4	4,627,496.4
6.861		6.861	89%		0.890	-0.730	1,389,411.6	9,532,336.4	4,390,176.4
6.897		6.897	90%		0.900	-0.738	1,347,245.9	9,291,955.1	4,149,795.1
6.933		6.933	91%		0.910	-0.746	1,305,080.2	9,048,512.6	3,906,352.6
6.970		6.970	92%		0.920	-0.754	1,262,914.5	8,802,008.9	3,659,848.9
7.006		7.006	93%		0.930	-0.763	1,220,748.8	8,552,443.9	3,410,283.9
7.042		7.042	94%		0.940	-0.771	1,178,583.1	8,299,817.7	3,157,657.7
7.079		7.079	95%		0.950	-0.779	1,136,417.4	8,044,130.3	2,901,970.3
7.115		7.115	96%		0.960	-0.787	1,094,251.6	7,785,381.6	2,643,221.6
7.151		7.151	97%		0.970	-0.795	1,052,085.9	7,523,571.7	2,381,411.7
7.187		7.187	98%		0.980	-0.804	1,009,920.2	7,258,700.6	2,116,540.6
7.224		7.224	99%		0.990	-0.812	967,754.5	6,990,768.3	1,848,608.3
7.260		7.260	100%		1.000	-0.820	925,588.8	6,719,774.7	1,577,614.7
7.296		7.296	101%		1.010	-0.828	883,423.1	6,445,719.9	1,303,559.9
7.333		7.333	102%		1.020	-0.836	841,257.4	6,168,603.8	1,026,443.8
7.369		7.369	103%		1.030	-0.845	799,091.7	5,888,426.6	746,266.6
7.405		7.405	104%		1.040	-0.853	756,926.0	5,605,188.1	463,028.1
7.442		7.442	105%		1.050	-0.861	714,760.2	5,318,888.3	176,728.3

Datos en Balboas. El precio es resultado de seis marcas(Marlboro, Viceroy, Kent, Mentolados, LM y Kool en presentaciones de 20)

Fuente: Elaboración propia con datos del Instituto Nacional de Estadística y Censo, Dirección General de Ingresos

2.

Simulación preliminar de la curva de Laffer con la elasticidad de -1.04

Pr (New)	Tax	Ps = Pr*Tax	Dt	ε_p	ΔP	ΔC	C(New)	R(New)	ΔR(New)
1.830	0.325	0.595	0%	-1.04	0.000	0.000	2,814,461.1	914,699.9	-
1.836		0.601	1%		0.003	-0.003	2,804,948.2	1,684,925.4	770,225.5
1.842		0.607	2%		0.006	-0.007	2,795,435.3	1,695,836.9	781,137.0
1.848		0.613	3%		0.010	-0.010	2,785,922.4	1,706,635.2	791,935.3
1.854		0.619	4%		0.013	-0.014	2,776,409.6	1,717,320.4	802,620.5
1.860		0.624	5%		0.016	-0.017	2,766,896.7	1,727,892.4	813,192.5
1.866		0.630	6%		0.020	-0.020	2,757,383.8	1,738,351.3	823,651.4
1.872		0.636	7%		0.023	-0.024	2,747,870.9	1,748,697.0	833,997.1
1.878		0.642	8%		0.026	-0.027	2,738,358.0	1,758,929.5	844,229.7
1.884		0.648	9%		0.029	-0.030	2,728,845.2	1,769,048.9	854,349.1
1.889		0.654	10%		0.033	-0.034	2,719,332.3	1,779,055.2	864,355.3
1.895		0.660	11%		0.036	-0.037	2,709,819.4	1,788,948.3	874,248.4
1.901		0.666	12%		0.039	-0.041	2,700,306.5	1,798,728.2	884,028.3
1.907		0.672	13%		0.042	-0.044	2,690,793.7	1,808,395.0	893,695.1
1.913		0.678	14%		0.045	-0.047	2,681,280.8	1,817,948.6	903,248.7
1.919		0.684	15%		0.049	-0.051	2,671,767.9	1,827,389.1	912,689.2
1.925		0.690	16%		0.052	-0.054	2,662,255.0	1,836,716.4	922,016.5
1.931		0.696	17%		0.055	-0.057	2,652,742.1	1,845,930.5	931,230.7
1.937		0.702	18%		0.059	-0.061	2,643,229.3	1,855,031.5	940,331.7
1.943		0.708	19%		0.062	-0.064	2,633,716.4	1,864,019.4	949,319.5
1.949		0.714	20%		0.065	-0.068	2,624,203.5	1,872,894.0	958,194.2
1.955		0.720	21%		0.068	-0.071	2,614,690.6	1,881,655.6	966,955.7
1.961		0.726	22%		0.071	-0.074	2,605,177.8	1,890,304.0	975,604.1
1.967		0.732	23%		0.075	-0.078	2,595,664.9	1,898,839.2	984,139.3
1.973		0.737	24%		0.078	-0.081	2,586,152.0	1,907,261.2	992,561.4
1.979		0.743	25%		0.081	-0.085	2,576,639.1	1,915,570.1	1,000,870.3
1.985		0.749	26%		0.085	-0.088	2,567,126.2	1,923,765.9	1,009,066.0
1.991		0.755	27%		0.088	-0.091	2,557,613.4	1,931,848.5	1,017,148.6
1.997		0.761	28%		0.091	-0.095	2,548,100.5	1,939,817.9	1,025,118.1
2.002		0.767	29%		0.094	-0.098	2,538,587.6	1,947,674.2	1,032,974.4
2.008		0.773	30%		0.097	-0.101	2,529,074.7	1,955,417.3	1,040,717.5
2.014		0.779	31%		0.101	-0.105	2,519,561.8	1,963,047.3	1,048,347.5
2.020		0.785	32%		0.104	-0.108	2,510,049.0	1,970,564.1	1,055,864.3
2.026		0.791	33%		0.107	-0.112	2,500,536.1	1,977,967.8	1,063,268.0
2.032		0.797	34%		0.111	-0.115	2,491,023.2	1,985,258.3	1,070,558.5
2.038		0.803	35%		0.114	-0.118	2,481,510.3	1,992,435.7	1,077,735.8

Pr (New)	Tax	Ps = Pr*Tax	Dt	ε_p	ΔP	ΔC	C(New)	R(New)	ΔR(New)
2.044		0.809	36%		0.117	-0.122	2,471,997.5	1,999,499.9	1,084,800.0
2.050		0.815	37%		0.120	-0.125	2,462,484.6	2,006,450.9	1,091,751.1
2.056		0.821	38%		0.124	-0.128	2,452,971.7	2,013,288.8	1,098,588.9
2.062		0.827	39%		0.127	-0.132	2,443,458.8	2,020,013.5	1,105,313.7
2.068		0.833	40%		0.130	-0.135	2,433,945.9	2,026,625.1	1,111,925.2
2.074		0.839	41%		0.133	-0.139	2,424,433.1	2,033,123.5	1,118,423.7
2.080		0.845	42%		0.137	-0.142	2,414,920.2	2,039,508.8	1,124,808.9
2.086		0.850	43%		0.140	-0.145	2,405,407.3	2,045,780.9	1,131,081.0
2.092		0.856	44%		0.143	-0.149	2,395,894.4	2,051,939.8	1,137,240.0
2.098		0.862	45%		0.146	-0.152	2,386,381.5	2,057,985.6	1,143,285.8
2.104		0.868	46%		0.150	-0.155	2,376,868.7	2,063,918.3	1,149,218.4
2.110		0.874	47%		0.153	-0.159	2,367,355.8	2,069,737.7	1,155,037.9
2.115		0.880	48%		0.156	-0.162	2,357,842.9	2,075,444.1	1,160,744.2
2.121		0.886	49%		0.159	-0.166	2,348,330.0	2,081,037.2	1,166,337.4
2.127		0.892	50%		0.163	-0.169	2,338,817.2	2,086,517.3	1,171,817.4
2.133		0.898	51%		0.166	-0.172	2,329,304.3	2,091,884.1	1,177,184.3
2.139		0.904	52%		0.169	-0.176	2,319,791.4	2,097,137.8	1,182,438.0
2.145		0.910	53%		0.172	-0.179	2,310,278.5	2,102,278.4	1,187,578.5
2.151		0.916	54%		0.176	-0.183	2,300,765.6	2,107,305.8	1,192,605.9
2.157		0.922	55%		0.179	-0.186	2,291,252.8	2,112,220.0	1,197,520.1
2.163		0.928	56%		0.182	-0.189	2,281,739.9	2,117,021.1	1,202,321.2
2.169		0.934	57%		0.185	-0.193	2,272,227.0	2,121,709.0	1,207,009.2
2.175		0.940	58%		0.189	-0.196	2,262,714.1	2,126,283.8	1,211,583.9
2.181		0.946	59%		0.192	-0.199	2,253,201.2	2,130,745.4	1,216,045.5
2.187		0.952	60%		0.195	-0.203	2,243,688.4	2,135,093.9	1,220,394.0
2.193		0.958	61%		0.198	-0.206	2,234,175.5	2,139,329.2	1,224,629.3
2.199		0.963	62%		0.202	-0.210	2,224,662.6	2,143,451.3	1,228,751.5
2.205		0.969	63%		0.205	-0.213	2,215,149.7	2,147,460.3	1,232,760.4
2.211		0.975	64%		0.208	-0.216	2,205,636.9	2,151,356.1	1,236,656.3
2.217		0.981	65%		0.211	-0.220	2,196,124.0	2,155,138.8	1,240,439.0
2.223		0.987	66%		0.215	-0.223	2,186,611.1	2,158,808.3	1,244,108.5
2.228		0.993	67%		0.218	-0.226	2,177,098.2	2,162,364.7	1,247,664.9
2.234		0.999	68%		0.221	-0.230	2,167,585.3	2,165,807.9	1,251,108.1
2.240		1.005	69%		0.224	-0.233	2,158,072.5	2,169,138.0	1,254,438.1
2.246		1.011	70%		0.228	-0.237	2,148,559.6	2,172,354.9	1,257,655.0
2.252		1.017	71%		0.231	-0.240	2,139,046.7	2,175,458.6	1,260,758.8
2.258		1.023	72%		0.234	-0.243	2,129,533.8	2,178,449.2	1,263,749.4
2.264		1.029	73%		0.237	-0.247	2,120,021.0	2,181,326.7	1,266,626.8
2.270		1.035	74%		0.241	-0.250	2,110,508.1	2,184,090.9	1,269,391.1

Pr (New)	Tax	Ps = Pr*Tax	Dt	ε_p	ΔP	ΔC	C(New)	R(New)	ΔR(New)
2.276		1.041	75%		0.244	-0.254	2,100,995.2	2,186,742.1	1,272,042.2
2.282		1.047	76%		0.247	-0.257	2,091,482.3	2,189,280.0	1,274,580.2
2.288		1.053	77%		0.250	-0.260	2,081,969.4	2,191,704.8	1,277,005.0
2.294		1.059	78%		0.254	-0.264	2,072,456.6	2,194,016.5	1,279,316.6
2.300		1.065	79%		0.257	-0.267	2,062,943.7	2,196,215.0	1,281,515.1
2.306		1.071	80%		0.260	-0.270	2,053,430.8	2,198,300.3	1,283,600.5
2.312		1.076	81%		0.263	-0.274	2,043,917.9	2,200,272.5	1,285,572.7
2.318		1.082	82%		0.267	-0.277	2,034,405.0	2,202,131.6	1,287,431.7
2.324		1.088	83%		0.270	-0.281	2,024,892.2	2,203,877.4	1,289,177.6
2.330		1.094	84%		0.273	-0.284	2,015,379.3	2,205,510.2	1,290,810.3
2.336		1.100	85%		0.276	-0.287	2,005,866.4	2,207,029.7	1,292,329.9
2.341		1.106	86%		0.280	-0.291	1,996,353.5	2,208,436.1	1,293,736.3
2.347		1.112	87%		0.283	-0.294	1,986,840.7	2,209,729.4	1,295,029.6
2.353		1.118	88%		0.286	-0.297	1,977,327.8	2,210,909.5	1,296,209.7
2.359		1.124	89%		0.289	-0.301	1,967,814.9	2,211,976.4	1,297,276.6
2.365		1.130	90%		0.293	-0.304	1,958,302.0	2,212,930.2	1,298,230.4
2.371		1.136	91%		0.296	-0.308	1,948,789.1	2,213,770.9	1,299,071.0
2.377		1.142	92%		0.299	-0.311	1,939,276.3	2,214,498.3	1,299,798.5
2.383		1.148	93%		0.302	-0.314	1,929,763.4	2,215,112.7	1,300,412.8
2.389		1.154	94%		0.306	-0.318	1,920,250.5	2,215,613.8	1,300,914.0
2.395		1.160	95%		0.309	-0.321	1,910,737.6	2,216,001.8	1,301,302.0
2.401		1.166	96%		0.312	-0.324	1,901,224.7	2,216,276.7	1,301,576.8
2.407		1.172	97%		0.315	-0.328	1,891,711.9	2,216,438.4	1,301,738.5
2.413		1.178	98%		0.319	-0.331	1,882,199.0	2,216,486.9	1,301,787.1
2.419		1.184	99%		0.322	-0.335	1,872,686.1	2,216,422.3	1,301,722.5
2.425		1.190	100%		0.325	-0.338	1,863,173.2	2,216,244.6	1,301,544.7
2.431		1.195	101%		0.328	-0.341	1,853,660.4	2,215,953.6	1,301,253.8
2.437		1.201	102%		0.332	-0.345	1,844,147.5	2,215,549.6	1,300,849.7
2.443		1.207	103%		0.335	-0.348	1,834,634.6	2,215,032.3	1,300,332.5
2.449		1.213	104%		0.338	-0.352	1,825,121.7	2,214,401.9	1,299,702.1
2.454		1.219	105%		0.341	-0.355	1,815,608.8	2,213,658.4	1,298,958.5
2.460		1.225	106%		0.345	-0.358	1,806,096.0	2,212,801.7	1,298,101.8
2.466		1.231	107%		0.348	-0.362	1,796,583.1	2,211,831.8	1,297,132.0
2.472		1.237	108%		0.351	-0.365	1,787,070.2	2,210,748.8	1,296,049.0
2.478		1.243	109%		0.354	-0.368	1,777,557.3	2,209,552.6	1,294,852.8
2.484		1.249	110%		0.358	-0.372	1,768,044.4	2,208,243.3	1,293,543.5
2.490		1.255	111%		0.361	-0.375	1,758,531.6	2,206,820.8	1,292,121.0
2.496		1.261	112%		0.364	-0.379	1,749,018.7	2,205,285.2	1,290,585.3
2.502		1.267	113%		0.367	-0.382	1,739,505.8	2,203,636.4	1,288,936.6

Pr (New)	Tax	Ps = Pr*Tax	Dt	ε_p	ΔP	ΔC	C(New)	R(New)	ΔR(New)
2.508		1.273	114%		0.371	-0.385	1,729,992.9	2,201,874.5	1,287,174.6
2.514		1.279	115%		0.374	-0.389	1,720,480.1	2,199,999.4	1,285,299.5
2.520		1.285	116%		0.377	-0.392	1,710,967.2	2,198,011.1	1,283,311.2
2.526		1.291	117%		0.380	-0.395	1,701,454.3	2,195,909.7	1,281,209.8
2.532		1.297	118%		0.384	-0.399	1,691,941.4	2,193,695.1	1,278,995.3
2.538		1.303	119%		0.387	-0.402	1,682,428.5	2,191,367.4	1,276,667.5
2.544		1.308	120%		0.390	-0.406	1,672,915.7	2,188,926.5	1,274,226.7
2.550		1.314	121%		0.393	-0.409	1,663,402.8	2,186,372.5	1,271,672.6
2.556		1.320	122%		0.397	-0.412	1,653,889.9	2,183,705.3	1,269,005.4
2.562		1.326	123%		0.400	-0.416	1,644,377.0	2,180,924.9	1,266,225.1
2.567		1.332	124%		0.403	-0.419	1,634,864.2	2,178,031.4	1,263,331.6
2.573		1.338	125%		0.406	-0.423	1,625,351.3	2,175,024.8	1,260,324.9
2.579		1.344	126%		0.410	-0.426	1,615,838.4	2,171,904.9	1,257,205.1
2.585		1.350	127%		0.413	-0.429	1,606,325.5	2,168,672.0	1,253,972.1
2.591		1.356	128%		0.416	-0.433	1,596,812.6	2,165,325.8	1,250,626.0
2.597		1.362	129%		0.419	-0.436	1,587,299.8	2,161,866.6	1,247,166.7
2.603		1.368	130%		0.423	-0.439	1,577,786.9	2,158,294.1	1,243,594.3
2.609		1.374	131%		0.426	-0.443	1,568,274.0	2,154,608.5	1,239,908.7
2.615		1.380	132%		0.429	-0.446	1,558,761.1	2,150,809.8	1,236,109.9
2.621		1.386	133%		0.432	-0.450	1,549,248.2	2,146,897.9	1,232,198.0
2.627		1.392	134%		0.436	-0.453	1,539,735.4	2,142,872.8	1,228,173.0
2.633		1.398	135%		0.439	-0.456	1,530,222.5	2,138,734.6	1,224,034.7
2.639		1.404	136%		0.442	-0.460	1,520,709.6	2,134,483.2	1,219,783.4
2.645		1.410	137%		0.445	-0.463	1,511,196.7	2,130,118.7	1,215,418.8
2.651		1.416	138%		0.449	-0.466	1,501,683.9	2,125,641.0	1,210,941.2
2.657		1.421	139%		0.452	-0.470	1,492,171.0	2,121,050.2	1,206,350.3
2.663		1.427	140%		0.455	-0.473	1,482,658.1	2,116,346.2	1,201,646.3
2.669		1.433	141%		0.458	-0.477	1,473,145.2	2,111,529.0	1,196,829.2
2.675		1.439	142%		0.462	-0.480	1,463,632.3	2,106,598.7	1,191,898.9
2.680		1.445	143%		0.465	-0.483	1,454,119.5	2,101,555.2	1,186,855.4
2.686		1.451	144%		0.468	-0.487	1,444,606.6	2,096,398.6	1,181,698.8
2.692		1.457	145%		0.471	-0.490	1,435,093.7	2,091,128.9	1,176,429.0
2.698		1.463	146%		0.475	-0.493	1,425,580.8	2,085,745.9	1,171,046.1
2.704		1.469	147%		0.478	-0.497	1,416,067.9	2,080,249.8	1,165,550.0
2.710		1.475	148%		0.481	-0.500	1,406,555.1	2,074,640.6	1,159,940.7
2.716		1.481	149%		0.484	-0.504	1,397,042.2	2,068,918.2	1,154,218.3
2.722		1.487	150%		0.488	-0.507	1,387,529.3	2,063,082.6	1,148,382.8
2.728		1.493	151%		0.491	-0.510	1,378,016.4	2,057,133.9	1,142,434.1
2.734		1.499	152%		0.494	-0.514	1,368,503.6	2,051,072.1	1,136,372.2

Pr (New)	Tax	Ps = Pr*Tax	Dt	ε_p	ΔP	ΔC	C(New)	R(New)	ΔR(New)
2.740		1.505	153%		0.497	-0.517	1,358,990.7	2,044,897.1	1,130,197.2
2.746		1.511	154%		0.501	-0.521	1,349,477.8	2,038,608.9	1,123,909.0
2.752		1.517	155%		0.504	-0.524	1,339,964.9	2,032,207.5	1,117,507.7
2.758		1.523	156%		0.507	-0.527	1,330,452.0	2,025,693.1	1,110,993.2
2.764		1.529	157%		0.510	-0.531	1,320,939.2	2,019,065.4	1,104,365.6
2.770		1.534	158%		0.514	-0.534	1,311,426.3	2,012,324.6	1,097,624.8
2.776		1.540	159%		0.517	-0.537	1,301,913.4	2,005,470.7	1,090,770.8
2.782		1.546	160%		0.520	-0.541	1,292,400.5	1,998,503.6	1,083,803.7
2.788		1.552	161%		0.523	-0.544	1,282,887.6	1,991,423.3	1,076,723.4
2.793		1.558	162%		0.527	-0.548	1,273,374.8	1,984,229.9	1,069,530.0
2.799		1.564	163%		0.530	-0.551	1,263,861.9	1,976,923.3	1,062,223.4
2.805		1.570	164%		0.533	-0.554	1,254,349.0	1,969,503.6	1,054,803.7
2.811		1.576	165%		0.536	-0.558	1,244,836.1	1,961,970.7	1,047,270.8
2.817		1.582	166%		0.540	-0.561	1,235,323.3	1,954,324.6	1,039,624.8
2.823		1.588	167%		0.543	-0.564	1,225,810.4	1,946,565.4	1,031,865.6
2.829		1.594	168%		0.546	-0.568	1,216,297.5	1,938,693.1	1,023,993.2
2.835		1.600	169%		0.549	-0.571	1,206,784.6	1,930,707.6	1,016,007.7
2.841		1.606	170%		0.553	-0.575	1,197,271.7	1,922,608.9	1,007,909.0
2.847		1.612	171%		0.556	-0.578	1,187,758.9	1,914,397.1	999,697.2
2.853		1.618	172%		0.559	-0.581	1,178,246.0	1,906,072.1	991,372.2
2.859		1.624	173%		0.562	-0.585	1,168,733.1	1,897,634.0	982,934.1
2.865		1.630	174%		0.566	-0.588	1,159,220.2	1,889,082.7	974,382.8
2.871		1.636	175%		0.569	-0.592	1,149,707.3	1,880,418.2	965,718.4
2.877		1.642	176%		0.572	-0.595	1,140,194.5	1,871,640.6	956,940.8
2.883		1.647	177%		0.575	-0.598	1,130,681.6	1,862,749.9	948,050.0
2.889		1.653	178%		0.579	-0.602	1,121,168.7	1,853,746.0	939,046.1
2.895		1.659	179%		0.582	-0.605	1,111,655.8	1,844,628.9	929,929.0
2.901		1.665	180%		0.585	-0.608	1,102,143.0	1,835,398.7	920,698.8
2.906		1.671	181%		0.588	-0.612	1,092,630.1	1,826,055.3	911,355.4
2.912		1.677	182%		0.592	-0.615	1,083,117.2	1,816,598.8	901,898.9
2.918		1.683	183%		0.595	-0.619	1,073,604.3	1,807,029.1	892,329.2
2.924		1.689	184%		0.598	-0.622	1,064,091.4	1,797,346.2	882,646.4
2.930		1.695	185%		0.601	-0.625	1,054,578.6	1,787,550.2	872,850.4
2.936		1.701	186%		0.605	-0.629	1,045,065.7	1,777,641.1	862,941.2
2.942		1.707	187%		0.608	-0.632	1,035,552.8	1,767,618.7	852,918.9
2.948		1.713	188%		0.611	-0.635	1,026,039.9	1,757,483.3	842,783.4
2.954		1.719	189%		0.614	-0.639	1,016,527.1	1,747,234.7	832,534.8
2.960		1.725	190%		0.618	-0.642	1,007,014.2	1,736,872.9	822,173.0
2.966		1.731	191%		0.621	-0.646	997,501.3	1,726,397.9	811,698.1

Pr (New)	Tax	Ps = Pr*Tax	Dt	ε_p	ΔP	ΔC	C(New)	R(New)	ΔR(New)
2.972		1.737	192%		0.624	-0.649	987,988.4	1,715,809.8	801,110.0
2.978		1.743	193%		0.627	-0.652	978,475.5	1,705,108.6	790,408.7
2.984		1.749	194%		0.631	-0.656	968,962.7	1,694,294.2	779,594.3
2.990		1.755	195%		0.634	-0.659	959,449.8	1,683,366.6	768,666.8
2.996		1.760	196%		0.637	-0.662	949,936.9	1,672,325.9	757,626.1
3.002		1.766	197%		0.640	-0.666	940,424.0	1,661,172.0	746,472.2
3.008		1.772	198%		0.644	-0.669	930,911.1	1,649,905.0	735,205.2
3.014		1.778	199%		0.647	-0.673	921,398.3	1,638,524.8	723,825.0
3.020		1.784	200%		0.650	-0.676	911,885.4	1,627,031.5	712,331.7
3.025		1.790	201%		0.653	-0.679	902,372.5	1,615,425.0	700,725.2
3.031		1.796	202%		0.657	-0.683	892,859.6	1,603,705.4	689,005.5
3.037		1.802	203%		0.660	-0.686	883,346.8	1,591,872.6	677,172.7
3.043		1.808	204%		0.663	-0.690	873,833.9	1,579,926.6	665,226.7
3.049		1.814	205%		0.666	-0.693	864,321.0	1,567,867.5	653,167.6
3.055		1.820	206%		0.670	-0.696	854,808.1	1,555,695.2	640,995.4
3.061		1.826	207%		0.673	-0.700	845,295.2	1,543,409.8	628,709.9
3.067		1.832	208%		0.676	-0.703	835,782.4	1,531,011.2	616,311.4
3.073		1.838	209%		0.679	-0.706	826,269.5	1,518,499.5	603,799.6
3.079		1.844	210%		0.683	-0.710	816,756.6	1,505,874.6	591,174.7
3.085		1.850	211%		0.686	-0.713	807,243.7	1,493,136.5	578,436.7
3.091		1.856	212%		0.689	-0.717	797,730.8	1,480,285.3	565,585.5
3.097		1.862	213%		0.692	-0.720	788,218.0	1,467,321.0	552,621.1
3.103		1.868	214%		0.696	-0.723	778,705.1	1,454,243.4	539,543.6
3.109		1.873	215%		0.699	-0.727	769,192.2	1,441,052.8	526,352.9
3.115		1.879	216%		0.702	-0.730	759,679.3	1,427,748.9	513,049.1
3.121		1.885	217%		0.705	-0.733	750,166.5	1,414,332.0	499,632.1
3.127		1.891	218%		0.709	-0.737	740,653.6	1,400,801.8	486,102.0
3.133		1.897	219%		0.712	-0.740	731,140.7	1,387,158.5	472,458.7
3.138		1.903	220%		0.715	-0.744	721,627.8	1,373,402.1	458,702.2
3.144		1.909	221%		0.718	-0.747	712,114.9	1,359,532.5	444,832.6
3.150		1.915	222%		0.722	-0.750	702,602.1	1,345,549.7	430,849.8
3.156		1.921	223%		0.725	-0.754	693,089.2	1,331,453.8	416,753.9
3.162		1.927	224%		0.728	-0.757	683,576.3	1,317,244.7	402,544.9
3.168		1.933	225%		0.731	-0.761	674,063.4	1,302,922.5	388,222.6
3.174		1.939	226%		0.735	-0.764	664,550.5	1,288,487.1	373,787.2
3.180		1.945	227%		0.738	-0.767	655,037.7	1,273,938.6	359,238.7
3.186		1.951	228%		0.741	-0.771	645,524.8	1,259,276.9	344,577.0
3.192		1.957	229%		0.744	-0.774	636,011.9	1,244,502.0	329,802.2
3.198		1.963	230%		0.748	-0.777	626,499.0	1,229,614.0	314,914.1

Pr (New)	Tax	Ps = Pr*Tax	Dt	ε_p	ΔP	ΔC	C(New)	R(New)	ΔR(New)
3.204		1.969	231%		0.751	-0.781	616,986.2	1,214,612.8	299,913.0
3.210		1.975	232%		0.754	-0.784	607,473.3	1,199,498.5	284,798.7
3.216		1.981	233%		0.757	-0.788	597,960.4	1,184,271.0	269,571.2
3.222		1.986	234%		0.761	-0.791	588,447.5	1,168,930.4	254,230.6
3.228		1.992	235%		0.764	-0.794	578,934.6	1,153,476.6	238,776.8
3.234		1.998	236%		0.767	-0.798	569,421.8	1,137,909.7	223,209.8
3.240		2.004	237%		0.770	-0.801	559,908.9	1,122,229.6	207,529.7
3.246		2.010	238%		0.774	-0.804	550,396.0	1,106,436.3	191,736.5
3.251		2.016	239%		0.777	-0.808	540,883.1	1,090,529.9	175,830.1
3.257		2.022	240%		0.780	-0.811	531,370.3	1,074,510.4	159,810.5
3.263		2.028	241%		0.783	-0.815	521,857.4	1,058,377.6	143,677.8
3.269		2.034	242%		0.787	-0.818	512,344.5	1,042,131.8	127,431.9
3.275		2.040	243%		0.790	-0.821	502,831.6	1,025,772.7	111,072.9
3.281		2.046	244%		0.793	-0.825	493,318.7	1,009,300.5	94,600.7
3.287		2.052	245%		0.796	-0.828	483,805.9	992,715.2	78,015.3
3.293		2.058	246%		0.800	-0.831	474,293.0	976,016.7	61,316.8
3.299		2.064	247%		0.803	-0.835	464,780.1	959,205.0	44,505.2
3.305		2.070	248%		0.806	-0.838	455,267.2	942,280.2	27,580.4
3.311		2.076	249%		0.809	-0.842	445,754.3	925,242.3	10,542.4

Nota: Datos en Balboas. El precio es resultado de seis marcas(Marlboro, Viceroy, Kent, Mentolados, LM y Kool en presentaciones de 20)

Fuente: Elaboración propia con datos del Instituto Nacional de Estadística y Censo, Dirección General de Ingresos
