# Supplementary material for: The Association of Tobacco Control Policies and the Risk of Acute Myocardial Infarction Using Hospital Admissions Data
Source: PLoS One. 2014 Feb 10;9(2):e88784. doi: 10.1371/journal.pone.0088784 (PMC3919809; doi:10.1371/journal.pone.0088784)
Supplement: Document S7 — Air Pollution Emissions Control in Panama. (PDF) [file pone.0088784.s008.pdf]

REPÚBLICA DE PANAMÁ

MINISTERIO DE ECONOMIA Y FINANZAS

DECRETO EJECUTIVO No. 5

(De 4 de febrero de 2009)

"Por el cual se dictan Normas Ambientales de Emisiones de Fuentes Fijas"

EL PRESIDENTE DE LA REPÚBLICA

En uso de sus facultades constitucionales y legales,

CONSIDERANDO:

Que la Constitución Política de la República de Panamá, en su Artículo 118 establece que es deber fundamental del Estado garantizar que la población viva en un ambiente sano y libre de contaminación, en donde el aire, el agua y los alimentos satisfagan los requerimientos de desarrollo adecuado de la vida humana.

Que la Ley 41 de 1 de julio de 1998, General de Ambiente de la República de Panamá, establece en su Artículo 1, que la administración del ambiente es una obligación del Estado; por lo tanto, establece los principios y normas básicas para la protección, conservación y recuperación del ambiente, promoviendo el uso sostenible de los recursos naturales. Además, ordena la gestión ambiental y la integra a los objetivos sociales y económicos, a efecto de lograr el desarrollo humano sostenible en el país.

Que mediante el Artículo 5 de la Ley 41 de 1998, se crea la Autoridad Nacional del Ambiente (ANAM), como la entidad autónoma rectora del Estado en materia de recursos naturales y del ambiente, para asegurar el cumplimiento y aplicación de las leyes, los reglamentos y la política nacional del ambiente.

Que de conformidad a lo establecido en los Artículos 7 y 32 de la precitada Ley, la Autoridad Nacional del Ambiente queda facultada para dictar normas de calidad ambiental con la participación de la Autoridad Competente.

Que el Artículo 56 de la Ley 41 de 1998, establece que el Ministerio de Salud es la autoridad encargada de normar, vigilar, controlar y sancionar todo lo relativo a garantizar la salud humana. Así mismo, desde la perspectiva de la salud ambiental, coordinará con la Autoridad Nacional del Ambiente, las medidas técnicas y administrativas a fin de que las alteraciones ambientales no afecten directamente la salud humana.

Que el Artículo 88 de la Ley 66 de 10 de noviembre de 1947, señala como atribuciones del Ministerio de Salud, la de dictar medidas tendientes a evitar o suprimir las molestias públicas y ubicar en zonas determinadas las industrias peligrosas o molestas.

DECRETA:

TÍTULO I

DISPOSICIONES GENERALES

CAPÍTULO I

Objeto y Ámbito de Aplicación

Artículo 1. El presente Decreto Ejecutivo tiene por objeto establecer los límites máximos permisibles de emisiones al aire producidas por fuentes fijas con el fin de proteger la salud de la población, los recursos naturales, y la calidad del ambiente, de la contaminación atmosférica.

Artículo 2. El ámbito de aplicación es todo el territorio de la República de Panamá.

CAPÍTULO II

Definiciones Básicas

Artículo 3. Para los efectos del presente Decreto Ejecutivo regirán los siguientes términos y definiciones:

1. Aire o aire ambiente: Porción no confinada de la atmósfera consistente en una mezcla gaseosa cuya composición volumétrica normal mínima es de veinte por ciento (20%) de oxígeno, setenta y siete por ciento (77%) nitrógeno, proporciones variables de gases inertes y vapor de agua.
2. Autoridad Competente: Institución pública que por mandato legal, ejerce los poderes, la autoridad y las funciones especializadas, relacionadas con aspectos parciales o componentes del ambiente, con el manejo sostenible de los

recursos naturales, o con la protección de la salud pública.

3. Caracterización de emisiones: Descripción cualitativa y cuantitativa de las emisiones al aire.
4. Carga contaminante: Aporte diario de contaminantes referido al material particulado, de una fuente fija de combustión, la cual es referida por la fórmula CC = A x FE x (1-EC/100) y que es expresada en toneladas por día.
5. Chimenea: Conducto que facilita el transporte hacia la atmósfera de los productos de combustión generados en la fuente fija.
6. Combustión: Oxidación rápida debida a la combinación del oxígeno con materiales o sustancias capaces de oxidarse, resultando generación de gases, partículas, luz y calor.
7. Combustible alterno: Sustituto de los combustibles líquidos tradicionales derivados del petróleo, basado en la mezcla de combustibles basados en alcohol con gasolina, metanol, etanol, gas natural comprimido y otros.
8. Condiciones normales de presión y temperatura: Se consideran condiciones normales de presión de mil trece milibares (1,013 mbar) o ciento uno con tres kilo pascales (101.3 kPa) y temperatura de cero grados centígrados (0 °C) o doscientos setenta y tres con quince grados Kelvin (273.15 ºK), en base seca, corregidos al quince por ciento (15%) de oxígeno.
9. Contaminante del aire: Cualquier elemento, sustancia química, o combinación de éstos, presentes en niveles o concentraciones que representan un peligro para la salud humana, y el ecosistema.
10. Contaminación del aire: Presencia en el aire, de cualquier sustancia química y/o partículas, en niveles o proporciones que alteren negativamente el ambiente y/o amenacen la salud humana o los ecosistemas.
11. Dióxido de azufre: Gas incoloro e irritante formado principalmente por la combustión de combustibles fósiles.
12. Emisión: Transferencia o descarga de sustancias desde la fuente a la atmósfera libre.
13. Fuente Fija: Edificación o instalación, temporal o permanente donde se realizan operaciones que dan origen a la emisión de contaminantes al aire.
14. Fuente fija de combustión: Instalación o conjunto de instalaciones dedicada al desarrollo de operaciones industriales, comerciales o de servicios, en la cual se realiza un proceso de combustión, desde un lugar fijo, del cual se generan o pudiesen generar emisiones al aire.
15. Fuente fija existente: Fuente fija de combustión en operación, o que cuenta con su Estudio de Impacto Ambiental aprobado por la ANAM, antes de la publicación de la presente normativa en Gaceta Oficial.
16. Fuente fija modificada: Fuente fija de combustión existente que experimenta un incremento en su capacidad operativa, implicando la generación de mayores emisiones al aire.
17. Fuente fija no significativa: Fuente fija de combustión cuyo poder calorífico sea menor a tres millones de vatios (3 x 10^6 W) o diez millones de unidades térmicas británicas por hora (10 x 10^6 BTU/h).
18. Fuente fija nueva: Fuente fija de combustión que inicie operaciones, o aquellas fuentes nuevas con un Estudio de Impacto Ambiental aprobado por la ANAM, en fecha posterior a la publicación de esta normativa en Gaceta Oficial.
19. Fuente fija significativa: Fuente fija de combustión cuya potencia calorífica sea igual o mayor a tres millones de vatios (3 x 10^6 W) o diez millones de unidades térmicas británicas por hora (10 x 10^6 BTU/h).
20. Material particulado: Material sólido o líquido en forma de partículas, con excepción del agua no combinada, presente en el aire en condiciones normales de presión y temperatura.
21. Mejor tecnología de control disponible: Limitación de emisiones basada en el máximo grado de reducción alcanzable, considerando los impactos energéticos, ambientales y económicos, mediante la aplicación de los mejores procesos, métodos, sistemas y técnicas disponibles.
22. Método equivalente: Procedimiento de medición y análisis en el cual se pueden obtener resultados similares a los del método de referencia en la determinación de las emisiones de una determinada sustancia, y que es seleccionado para reemplazarlo.
23. Método de referencia: Procedimiento de medición y análisis probado exhaustivamente, señalado en el presente Decreto Ejecutivo, que debe utilizarse para determinar las emisiones de una determinada sustancia y deberá realizarse bajo estrictos parámetros técnicos.
24. Método Ringelmann: Técnica empleada para la medición de la opacidad, mediante el uso de tarjetas que poseen una escala comparativa denominada Escala de Ringelmann.
25. Mínima tasa de emisión posible: La limitación de emisiones más exigente alcanzable en la práctica.
26. Modelo de dispersión: Conjunto de relaciones y ecuaciones matemáticas y físicas de un sistema constituido por la fuente de emisión, así como las condiciones meteorológicas y topográficas de la región en estudio, basado en principios científicos de difusión, que se utilizan para relacionar las emisiones de contaminantes provenientes de una o más fuentes de emisiones, con los niveles esperados de contaminantes en el aire ambiente producto de dichas emisiones.
27. Monitoreo: Proceso programado de forma periódica o continua, para colectar muestras y/o efectuar mediciones, de una o varias características del ambiente o de emisiones, generalmente con el fin de evaluar el cumplimiento de los requisitos regulatorios específicos.
28. Norma Ambiental de Calidad de Aire: Norma que establece los valores de las concentraciones y períodos máximos permisibles de elementos, compuestos, sustancias, derivados químicos o biológicos, o la combinación de ellos, cuya presencia en el aire, en mayor grado que lo establecido, pueda constituir un riesgo para la vida o salud de la población o el ambiente.
29. Normas Ambientales de Emisión o Límites Máximos Permisibles: Valores que establecen la cantidad de emisión máxima permitida de un contaminante medido en la fuente emisora.
30. Opacidad: Es la condición por la cual un material, impide parcial o totalmente el paso de un haz de luz en un grado

apreciable.

31. Operador de fuente fija: Toda persona natural o jurídica que administra y desarrolla actividades o procesos en una fuente fija, bajo la figura de una concesión o a través de cualquier otra figura legal.
32. Operaciones de puesta en marcha y parada: Toda operación realizada al poner una actividad, un elemento del equipo o un dispositivo en servicio o fuera de él, o ponerlo y sacarlo de un estado de reposo.
33. Óxidos de nitrógeno (NOx): Compuestos producto de las reacciones fotoquímicas del óxido nítrico en el aire ambiente que constituyen el principal componente del smog fotoquímico. Se producen en los procesos de combustión de fuentes fijas y móviles y es uno de los principales gases contribuyentes a la formación de ozono en la tropósfera y la deposición ácida.
34. Partículas Totales: Material particulado de dimensiones y procedencia diversa, el cual es liberado a la atmósfera por elementos naturales, procesos mecánicos, industriales, y transporte, que es captado por un sistema de muestreo similar en características al descrito en el método 5 de medición de emisiones de partículas, publicado por la Agencia de Protección Ambiental de los Estados Unidos de América.
35. Puerto de muestreo: Plataformas y orificios que se ubican en las chimeneas o conductos, para facilitar la introducción de los elementos necesarios para mediciones y toma de muestras.
36. Puntos de medición: Puntos específicos, localizados en las líneas de muestreo, en los cuales se realizan las mediciones y se extrae la muestra respectiva.
37. US EPA: Agencia de Protección Ambiental de los Estados Unidos de América.

CAPÍTULO III

Competencias

Artículo 4. Corresponde a la Autoridad Nacional del Ambiente (en adelante, la ANAM) la aplicación del presente Decreto Ejecutivo. Para tales efectos la ANAM coordinará con las siguientes instituciones:

i) Ministerio de Salud, lo concerniente a la afectación a la salud como consecuencia de las emisiones de fuentes fijas y al otorgamiento del permiso sanitario.
ii) Ministerio de Comercio e Industrias, lo concerniente a los reglamentos y normas técnicas complementarias para la aplicación del presente Decreto Ejecutivo; el establecimiento de los requisitos que deben cumplir los organismos de inspección y la acreditación de laboratorios de ensayos; lo relativo a la revisión de la calidad del combustible que ingresa al país; así como los aspectos relacionados al desarrollo y fomento industrial respecto a las emisiones de fuentes fijas de combustión que se puedan producir.
iii) Organismo de consulta: la Universidad Tecnológica de Panamá u otra entidad que designe ANAM, en lo relativo a la medición de emisiones de fuentes fijas.

TÍTULO II

VALORES DE LOS LÍMITES MÁXIMOS PERMISIBLES

CAPÍTULO I

Límites Máximos Permisibles para Fuentes Fijas Existentes

Artículo 5. Se establecen los límites máximos permisibles de emisión para fuentes fijas existentes, conforme a lo indicado en la tabla siguiente.

Límites Máximos Permisibles de Emisiones al Aire para Fuentes Fijas

(Referencia: Guía del Banco Mundial 1998)

Actividad (CIIU)	Límites Máximos Permisibles (mg/Nm^3 a menos que indique otra unidad)[a] Partículas Totales	Óxidos de Azufre	Óxidos de Nitrógeno		
Producción de Cemento (3692)	50	400	600		
Fundiciones (3720)	20[b]	---	---		
Molinos de Papel[c] (3419)	100[d]	---	2 kg/t de PS[e]		
Ingenios Azucareros (3131)	100[i]	2000[h]	Combustibles líquidos: 460[f]<br>Combustibles sólidos: 750[g]		
Generación Termoeléctrica[j] (4101)	50[k, l]	0.2 tpd/MW (hasta 500 MW)<br>0.1 tpd/MW (incrementos arriba de 500 MW)<br>No se puede exceder 2000 mg/Nm^3 ni 500 tpd	Carbón: 750[m]<br>Petróleo: 460<br>Gas: 320		
Generación Termoeléctrica con Turbinas de Gas (4101)					Gas: 125<br>Diesel Nº2: 165<br>Bunker Nº6 y otros: 300
Fabricación de Productos Petroquímicos		20	500		300
Refinación de Petróleo		50	150 (unidades con recuperación de azufre)<br>500 (unidades de combustión)		460
Fabricación de Productos Farmacéuticos		20	---		---
Procesamiento de Aceite Vegetal		50	---		---
Otras Actividades[q]		50[n]<br>100[o]	2000[p]		Carbón: 750<br>Petróleo: 460<br>Gas: 320

a. Se consideran condiciones normales mil trece milibares de presión (1,013 mbar) o ciento uno con tres kilo pascales (101.3 kPa) y temperatura de 0 °C ó 273.15 ºK, en base seca y corregidos a 15% de oxígeno.

b. Si no hay presencia de metales tóxicos, el límite de partículas es de 50.

c. Capacidad de producción mayor o igual a 100 toneladas por día.

d. En hornos de recuperación. Si no resulta costo efectivo llegar a este nivel, se acepta un máximo de 150.

e. PS: Pulpa seca, se define como 90% de fibra seca y 10% de agua.

f. Aplicable a hornos de cal.

g. Aplicable a molinos de sulfito.

h. Aplicable a molinos kraft y demás.

i. El límite aplicable a molinos pequeños, con menos de 8.7 MW de poder calorífico de entrada a las calderas, es de 150.

j. En el caso de plantas térmicas de generación eléctrica con motores de combustión interna se permitirá un máximo de NOx de 2000 mg/Nm3, para aquellas que se instalaron después del año 2,000 y de 2,300 mg/Nm3 para aquellas que se instalaron antes de dicha fecha.

k. El límite aplicable a plantas con capacidad menor de 50 MW es de 100.

l. Para rehabilitación de plantas existentes el límite es 100.

m. Para carbón con menos de 10% de materia volátil, el límite aplicable es 1500.

n. Para instalaciones de capacidad igual o mayor de 50 MW.

o. Para instalaciones de capacidad menor de 50 MW.

p. Expresado como SO2

q. Se refiere a actividades no incluidas en la tabla.

Artículo 6. Las fuentes fijas de combustión que operen con un sistema de combustión dual, deberán cumplir los límites máximos permisibles de emisión, en función del tipo de combustible que utilicen en mayor cantidad o durante el mayor tiempo de operación.

CAPÍTULO II

Límites Máximos Permisibles para Fuentes Fijas Nuevas o Modificadas

Artículo 7. Toda fuente fija nueva o modificada, significativas o no, debe hacer uso de la Mejor Tecnología de Control Disponible, la cual deberá ser autorizada por la ANAM a través de la Resolución Administrativa que aprueba el Estudio de Impacto Ambiental. De no requerir un Estudio de Impacto Ambiental, la empresa al momento de presentar la solicitud de Permiso Sanitario deberá presentar su programa de la Mejor Tecnología de Control Disponible al MINSA, que emitirá concepto, y requerirá la evaluación y aprobación de dicho programa por parte de la ANAM, para la emisión del correspondiente Permiso Sanitario. En ningún caso, las emisiones serán mayores a los límites máximos permisibles señalados en la tabla del Artículo 5 del presente Decreto Ejecutivo.

TÍTULO III

CARACTERIZACIÓN DE EMISIONES DE FUENTES FIJAS DE COMBUSTION

CAPÍTULO I

Plazos y Procedimientos de Caracterización

Artículo 8. Para la determinación de los plazos de caracterización, se tomará como referencia la carga contaminante en toneladas por día de la fuente emisora referida al parámetro de material particulado. La carga contaminante se calculará a partir de la siguiente ecuación:

$$CC = A \times FE \times (1-EC/100)$$

Donde,

CC: Carga contaminante en toneladas por día.

A: Tasa productiva - Dato que la empresa debe proporcionar en función de su actividad productiva. El tipo de dato dependerá de las unidades como se especifique el factor de emisión.

FE: Factor de emisión de material particulado según la fuente fija de combustión que se está evaluando.

EC: Eficiencia de control en porcentaje. Sólo se aplicaría a aquellas empresas que ya tienen instalado algún sistema de control de emisiones, y su valor se determinaría a partir de los datos del fabricante de dicho sistema de control.

Parágrafo: En el caso de aquellas fuentes fijas de combustión, que cuenten con más de una fuente emisora, la carga contaminante se determinará para cada una de estas fuentes, y la suma aritmética de cada una de sus cargas contaminantes dictará el plazo de caracterización. En los casos que se utilice más de un tipo de combustible, la carga de contaminante se estimará de acuerdo al combustible que es más utililizado.

Artículo 9. La carga contaminante (CC) deberá calcularse de acuerdo a las capacidades máximas operativas de la fuente fija de combustión.

Artículo 10. Para efectos de determinar los plazos de caracterización y adecuación aplicables a las fuentes fijas de combustión existentes, se debe presentar el cálculo de la carga contaminante aplicable por actividad, a modo de Declaración Jurada, en un término no mayor de seis (6) meses a partir de la publicación en Gaceta Oficial del presente Decreto Ejecutivo.

Artículo 11. Las fuentes fijas de combustión existentes están obligadas a caracterizar sus emisiones al aire de los contaminantes descritos en la tabla del Artículo 5 y presentar los resultados a la ANAM de conformidad con los plazos señalados en el presente artículo.

Plazos de Caracterización de Emisiones de Fuentes Fijas de Combustión Existente

Carga Contaminante (CC en Ton/día)	Plazo de Caracterización[1]
Mayor de 2.0	12 meses
De 1.0 a 2.0	18 meses
Menor de 1.0	24 meses

[1] Los plazos empiezan a regir una vez finalizado el plazo estipulado en el Artículo 10.

Parágrafo: La ANAM podrá otorgar excepciones a lo anterior, para aquellas empresas que como parte de su Plan de Manejo Ambiental, Auditoría Ambiental y/o Programa de Adecuación y Manejo Ambiental, hayan caracterizado sus emisiones tomando en consideración las metodologías y técnicas utilizadas para ello.

Artículo 12: La ANAM establecerá los procedimientos y formatos específicos para la presentación de la caracterización de las emisiones de fuentes fijas de combustión a través de Resolución Administrativa, en un plazo no mayor de treinta (30) días calendario, a partir de la publicación del presente Decreto Ejecutivo.

Artículo 13: La ANAM evaluará la caracterización presentada por el operador de la fuente fija de combustión, y establecerá su aceptación o rechazo, así como las correcciones que sean requeridas por la institución, a través de Resolución Administrativa.

Artículo 14: En caso de existir varias fuentes fijas de emisión dentro de una misma región, bajo la responsabilidad de un sólo propietario u operador, se calculará la emisión global de las fuentes mediante una fórmula que pondere las diversas fuentes fijas presentes. Para ello se utilizará la siguiente fórmula:

$$E_{global} = \frac{A_1 * E_1 + A_2 * E_2 + \ldots + A_i * E_i}{A_1 + A_2 + \ldots + A_i}$$

Donde,

Eglobal: Emisión global para el conjunto de fuentes fijas de combustión,

Ai: factor de ponderación, que puede ser el consumo de combustible de la fuente número i, o el caudal de gases de combustión de la respectiva fuente número i.

Ei: Emisión actual determinada para cada fuente.

Parágrafo: El resultado obtenido en la ecuación antes indicada, representa el equivalente ponderado para un grupo de fuentes fijas de combustión, el cual deberá ser comparado con el valor máximo de emisión establecido en este Decreto Ejecutivo, siendo este resultado, equivalente a las emisiones para una sola fuente fija de combustión.

CAPÍTULO II

Caracterización de Emisiones al Aire de Fuentes Fijas Significativas Existentes

Artículo 15. Las fuentes fijas significativas existentes deberán caracterizar sus emisiones de contaminantes, mediante la realización de mediciones directas de los gases a la salida de sus chimeneas, para los parámetros regulados en el presente Decreto Ejecutivo. El reporte de caracterización de las emisiones de fuentes fijas significativas existentes, deberá incluir lo siguiente:

a) Identificación de la fuente fija (nombre o razón social, Representante Legal, dirección), que se hará constar mediante Certificado de Existencia Legal expedida por el Registro Público;
b) Ubicación de la fuente fija, incluyendo mapa de localización y descripción del área circundante;
c) Nombres, firmas y sellos del personal autorizado y/o acreditado que efectuó la medición;
d) Introducción: propósito y lugar de la medición, fechas, contaminantes objeto de la medición, detalles de testigos presentes, tanto de la fuente como representantes de la autoridad competente (de aplicarse);
e) Resumen de resultados: incluyendo los resultados en sí obtenidos, datos del proceso de combustión, emisiones máximas permitidas para la fuente;
f) Características de operación de la fuente fija: descripción del proceso y de equipos o técnicas de control o reducción de emisiones (de aplicarse), descripción de materias primas o combustibles utilizados, propiedades relevantes de estos, y cualquier información pertinente sobre la operación de la fuente;
g) Métodos de muestreo y de análisis utilizados: ubicación de los puertos de muestreo y de los puntos de medición al interior de la chimenea, descripción de los equipos y/o accesorios utilizados en la recolección de muestras o medición, certificados de calibración vigente de acuerdo a las especificaciones del fabricante y el grado de incertidumbre del equipo; una breve discusión de los procedimientos de muestreo y de análisis utilizados, incluyendo cualquier desviación en el procedimiento, y las debidas justificaciones técnicas;
h) Modelo de dispersión aplicado y efecto de emisiones en la calidad de aire (de aplicar);
i) Estimación de las emisiones de gases de efecto invernadero según las metodologías que para tal efecto establezca la ANAM;
j) Anexos, los cuales incluirán cualquier información adicional de respaldo, además de los certificados de calibración vigentes, impresión de lecturas de instrumentos y fotos tomadas durante el proceso de medición.

Artículo 16. Las fuentes fijas significativas existentes, deberán evaluar su impacto en la calidad del aire mediante modelos de dispersión, de conformidad con lo establecido para las fuentes nuevas o modificadas, y presentar los resultados de su aplicación junto con la caracterización de sus emisiones.

CAPÍTULO III

Caracterización de Emisiones al Aire de Fuentes Fijas No Significativas Existentes

Artículo 17. Las fuentes fijas no significativas existentes no están obligadas a efectuar mediciones de sus emisiones actuales. No obstante, deberán evaluar sus emisiones, de acuerdo a alguno de los dos métodos contenidos en la siguiente tabla. Estos métodos también se utilizarán para demostrar el cumplimiento con la normativa vigente.

Métodos para la Caracterización y Cumplimiento de Emisiones

**Método 1**	Método 2
a) El registro interno del cumplimiento de las prácticas de mantenimiento de los equipos de combustión, de acuerdo con los programas establecidos por el operador de la fuente y aquellos recomendados por el fabricante del equipo de combustión;<br>b) Resultados del análisis de las características físico-químicas del combustible utilizado; y<br>c) La presentación de certificados del fabricante del equipo de combustión, en lo referente a la tasa esperada de emisiones, basándose en las características del combustible utilizado y la antigüedad del equipo.<br><br>Los resultados de la aplicación de este método, deberán demostrar el cumplimiento de los valores establecidos en la tabla 1.	a) Mediante inspección visual, por personal capacitado para ello, del nivel de opacidad de los gases de escape de la fuente, utilizando el método Ringelmann y considerando el cumplimiento de la normativa cuando los resultados no sobrepasan el valor de 1 de la escala utilizada en dicho método; y<br>b) Mediante el uso de altura de chimenea recomendada por las prácticas de ingeniería y del reglamento vigente de la Oficina de Seguridad del Cuerpo de Bomberos de Panamá, el cual regula a que altura deben colocarse las chimeneas.

Artículo 18. Para efectos de lo señalado en el Artículo 17 de este Decreto Ejecutivo, la Autoridad Nacional del Ambiente, podrá mediante Resolución Administrativa, establecer otros métodos que considere apropiados para determinar el cumplimiento de las fuentes fijas no significativas con los límites máximos permisibles establecidos en el presente Decreto Ejecutivo.

Artículo 19. Para la verificación del cumplimiento de una fuente fija no significativa existente, con alguno de los métodos descritos en el presente Decreto Ejecutivo, el operador o propietario de la fuente deberá mantener a disposición de la ANAM, los debidos registros o certificados y reportar anualmente el cumplimiento de los límites máximos permitidos establecidos en la presente norma.

Artículo 20. Las fuentes fijas no significativas existentes deberán presentar los resultados de su caracterización y/o demostración de cumplimiento bajo el siguiente formato:

a) Identificación de la fuente fija no significativa (nombre o razón social, Representante Legal, dirección), que se hará constar mediante Certificado de Existencia Legal expedida por el Registro Público;
b) Ubicación de la fuente fija, incluyendo mapa de localización y descripción del área circundante;
c) Nombres, firmas y sellos del personal autorizado y/o acreditado que efectuó la caracterización y verificación del cumplimiento;
d) Introducción: objetivos de la caracterización, fechas, contaminantes objeto de la caracterización y/o verificación del cumplimiento, antecedentes de la empresa y de la gestión ambiental desarrollada por la empresa;
e) Características de operación de la fuente fija: descripción del proceso y de equipos o técnicas de control o reducción de emisiones (de aplicarse), descripción de materias primas o combustibles utilizados, propiedades relevantes de éstos, y cualquier información pertinente sobre la operación de la fuente;
f) Método de caracterización y/o verificación de cumplimiento con la normativa vigente;
g) Resumen de resultados que incluya el procedimiento empleado para realizar la caracterización y/o verificación de cumplimiento, descripción de los resultados obtenidos ya sea por inspecciones visuales, verificaciones de las prácticas de mantenimiento del equipo, análisis de las características físico-químicas del combustible empleado, datos del proceso de combustión, emisiones máximas permitidas para la fuente, y opacidad de los gases.
h) Estimación de las emisiones de gases de efecto invernadero según las metodologías que para tal efecto establezca la ANAM;
i) Anexos, los cuales incluirán cualquier información adicional de respaldo, además de los certificados de calibración vigentes de los equipos empleados para la caracterización y de los certificados del fabricante del equipo de combustión, impresión de lecturas de instrumentos y fotos tomadas durante el proceso de medición.

Artículo 21. Adicional a lo establecido en el artículo anterior, cuando los resultados de la aplicación del método 1 ó 2 arrojen resultados dudosos o poco confiables, la ANAM podrá solicitar al propietario de la fuente fija no significativa existente, la medición directa de sus emisiones mediante los procedimientos y métodos aplicados a las fuentes fijas significativas existentes.

CAPÍTULO IV

Caracterización de Emisiones al Aire de Fuentes Fijas Significativas o No Significativas,

Nuevas o Modificadas

Artículo 22. Las fuentes fijas, sean estas significativas o no significativas, nuevas o modificadas, están obligadas a presentar ante la ANAM su caracterización de emisiones, una vez inicien operaciones o realicen alguna modificación.

Parágrafo: Bajo ninguna circunstancia la entrega de la caracterización podrá extenderse por un período mayor a dos (2) meses a partir del inicio de operaciones de la fuente fija nueva o modificada.

Artículo 23. Toda fuente fija que experimente una modificación sustancial de la misma y/o un cambio total o parcial de combustible, deberá informarlo a la ANAM y presentar la caracterización de emisiones correspondientes.

Artículo 24. En los casos en que a una fuente fija significativa, nueva o modificada de emisiones al aire, le corresponda la presentación de un Estudio de Impacto Ambiental de acuerdo a la normativa vigente, deberá incluirse en el mismo la evaluación de su impacto en la calidad del aire mediante el uso de modelos de dispersión avalados por la ANAM. Para los efectos de determinar que los niveles de inmisión determinados mediante el modelo deberán cumplir con la norma de calidad de aire ambiente vigente, o en su defecto, las normas de referencia a seguir que establezcan las Autoridades Competentes.

Artículo 25. Toda fuente fija nueva o modificada, calificada como significativa, deberá evaluar aquellos contaminantes emitidos por la misma, los cuales puedan considerarse significativos con la calidad del aire. Para la evaluación, se deberá utilizar un modelo de dispersión de tipo preliminar, avalado por la ANAM, mediante el cual se verificará si las concentraciones calculadas por el modelo, para cada contaminante, sobrepasan o no los valores establecidos en la norma de calidad de aire ambiente vigente, o en su defecto, las normas de referencia a seguir que establezcan las Autoridades Competentes.

Artículo 26. Si la predicción mediante el modelo preliminar indica que la concentración de un contaminante sobrepasará el valor establecido en la norma de calidad de aire ambiente vigente, se calificará al contaminante como significativo para la fuente, en cuyo caso se deberá aplicar un modelo detallado para aquellos contaminantes significativos.

Artículo 27. En el proceso de evaluación de impacto ambiental requerido, la fuente fija significativa nueva o modificada, podrá incluir o no dentro del modelo de dispersión aplicada, otras fuentes fijas existentes en la misma región, previa aprobación de la ANAM, tomando en consideración criterios técnicos y la calidad de aire ambiente.

Artículo 28. Las fuentes fijas nuevas deberán solicitar a la ANAM, bajo su propio costo, los resultados de la base de datos que administra de las emisiones de fuentes fijas significativas existentes, y de los niveles de concentraciones de contaminantes en el aire ambiente para el área en estudio.

Artículo 29. Para determinar el área de influencia, sea de una sola fuente fija nueva evaluada, o del conjunto de varias fuentes, se trazará una curva de igual concentración a través de la simulación con un modelo de dispersión ya sea del tipo preliminar o más detallado según sea el caso, para todos los contaminantes que sobrepasen los valores establecidos en la norma de calidad de aire ambiente vigente, o en su defecto, las normas de referencia a seguir que establezcan las Autoridades Competentes.

Artículo 30. Cuando sea necesaria la aplicación de un modelo de dispersión del tipo detallado avalado por la ANAM, el mismo deberá tener la capacidad de incluir diferentes fuentes fijas, y de predecir concentraciones de contaminantes para períodos de tiempo mayores a una hora. El uso de un modelo de dispersión del tipo detallado se extenderá también en el caso de un conjunto de fuentes fijas nuevas, o fuentes existentes modificadas, que estuviesen bajo la responsabilidad de una misma organización u operador, y se determine que la emisión global calculada según lo establecido en la presente norma que el conjunto de fuentes sea significativa.

Artículo 31. El modelo de dispersión detallado, evaluará el impacto máximo en la calidad del aire de la fuente fija significativa. Los datos meteorológicos a utilizarse deberán ser representativos de la ubicación geográfica de la fuente fija a evaluar. Ante la ausencia de datos meteorológicos para un área específica, se deberá evaluar el uso de datos correspondientes a otras estaciones cercanas que pudiesen guardar similitud con las condiciones ambientales del área en estudio.

Artículo 32. La instalación de fuentes fijas nuevas significativas cercana a los límites de las áreas protegidas legalmente establecidas, cuyas emisiones podrían afectar la calidad de aire ambiente, deberán ejecutar un programa de monitoreo inicial de concentraciones de contaminantes del aire en el interior de tales áreas, previo al inicio de operaciones. El número y ubicación de los sitios, así como la frecuencia y la época de muestreo se definirán en coordinación con la ANAM.

Artículo 33. Previo a la presentación del Estudio de Impacto Ambiental de toda fuente fija nueva, deberá coordinarse con la ANAM la determinación del número y ubicación de los sitios para la medición de las concentraciones de contaminantes del aire. El programa de monitoreo inicial deberá incluir como mínimo, la determinación de las concentraciones de óxidos de nitrógeno, dióxido de azufre, y partículas totales suspendidas.

Artículo 34. Las fuentes fijas nuevas no podrán instalarse en un área donde las concentraciones de contaminantes del aire no cumplan la norma de calidad de aire ambiente vigente, o en su defecto, las normas de referencia a seguir que establezcan las autoridades.

Parágrafo: Se exceptúan de esta disposición aquellas fuentes fijas nuevas que reemplacen a una o varias fuentes fijas existentes, y que garanticen estándares de emisión y de desempeño (cantidad de contaminante emitido por unidad de combustible utilizado) considerablemente menor al de la o las fuentes que serán reemplazadas. Como parte del Estudio de Impacto Ambiental para una fuente fija nueva, en un área de incumplimiento de la norma de calidad de aire ambiente, se deberán justificar las tecnologías o métodos que implementarán con el fin de alcanzar la mínima tasa de emisión posible, de manera que no se induzca a un mayor incumplimiento de la norma de calidad de aire.

Artículo 35. Las fuentes fijas modificadas utilizarán un esquema similar al descrito en el Artículo 33 del presente Decreto Ejecutivo. La aprobación será otorgada por la ANAM, a través de la Resolución Administrativa que aprueba el Estudio de Impacto Ambiental o el Programa de Adecuación y Manejo Ambiental, según corresponda.

Parágrafo: Cuando no se apliquen ninguno de los instrumentos previamente señalados, la aprobación será otorgada por el MINSA a través del permiso sanitario de operación.

Artículo 36. Las fuentes fijas nuevas significativas determinarán la altura apropiada de chimeneas mediante la aplicación de modelos de dispersión. La altura seleccionada de chimenea deberá considerar el efecto de turbulencia creado por la presencia de edificaciones adyacentes a la chimenea y cumplir con las regulaciones vigentes, establecidas por la Oficina de Seguridad del Cuerpo de Bomberos que regula sobre esta materia.

TÍTULO IV

ADECUACIÓN A LOS LÍMITES MÁXIMOS PERMISIBLES DE EMISIONES DE FUENTES FIJAS DE COMBUSTIÓN

Artículo 37. Toda fuente fija de combustión existente tiene la obligación de adecuar sus emisiones al aire a los límites máximos permisibles establecidos en el presente Decreto Ejecutivo, de conformidad con los plazos señalados en la presenta tabla, tomando en consideración la carga contaminante calculada de acuerdo a lo establecido en los Artículos 8 y 9 de esta norma.

Plazos de Adecuación a los Límites Máximos Permisibles de Emisiones de Fuentes Fijas de Combustión Existentes

Carga Contaminante (CC en Ton/día)	Plazo de Adecuación[1]
Mayor de 2.0	36 meses
De 1.0 a 2.0	48 meses
Menor de 1.0	60 meses

[1] Los plazos empiezan a regir con la promulgación del presente Decreto Ejecutivo en Gaceta Oficial.

Parágrafo: Bajo condiciones excepcionales, considerando las limitantes técnicas y económicas para la adecuación a los límites máximos permisibles establecidos en el presente Decreto Ejecutivo, se podrá ampliar el plazo de adecuación por un período no mayor a seis (6) meses. En tal caso, la fuente fija de combustión deberá notificar con una antelación no menor de nueve (9) meses, previo al plazo de cumplimiento que le corresponda, los motivos por los cuáles no podrá cumplir con los mismos y se acogerá a lo establecido en el Decreto Ejecutivo 57 de 2004, respecto a las auditorías ambientales voluntarias como mecanismo de flexibilización para el cumplimiento de la normativa vigente. La ANAM evaluará caso por caso, y hará saber su decisión mediante Resolución Administrativa, estableciendo las condiciones que deberá cumplir el solicitante.

Artículo 38. Las fuentes fijas de combustión nuevas o modificadas deberán ser planificadas, instaladas, organizadas y puestas en funcionamiento, de modo que sus emisiones en ningún momento excedan los límites máximos permisibles establecidos en el presente Decreto Ejecutivo.

TÍTULO V

CONTROL Y SEGUIMIENTO

Artículo 39. Las fuentes fijas de combustión significativas deberán monitorear permanentemente sus emisiones, y presentar anualmente un informe con los mismos requerimientos señalados en el Artículo 15 del presente Decreto Ejecutivo. Este informe debe elaborarse sobre el período de mayor actividad de la fuente y la duración del monitoreo estará definido según los métodos establecidos en la tabla del Artículo 52. En el caso de las fuentes fijas de combustión existentes, dicho monitoreo deberá iniciar una vez cumplido el plazo de adecuación de la fuente fija; mientras que para el caso de las fuentes fijas nuevas o modificadas, el monitoreo iniciará inmediatamente comience operaciones o se realice la modificación en cuestión.

Parágrafo: En caso que las emisiones al aire reflejen valores que superen los limites permisibles, reportados en el monitoreo e informe anual estipulado en el presente artículo, la ANAM podrá solicitar a la fuente emisora, monitoreos adicionales acompañados de medidas de autocontrol y/o incorporar planes de producción más limpia. Si la fuente emisora se encuentra bajo la aprobación o ejecución de un Plan de Manejo Ambiental (PMA) o Programa de Adecuación y Manejo Ambiental (PAMA), la ANAM verificará las frecuencias establecidas en tales instrumentos y de considerar las frecuencias presentadas como insuficientes, solicitará ampliarlas. En el caso del Programa de Adecuación y Manejo Ambiental, se deberá presentar una adenda al mismo con el fin de cumplir con esta normativa que le será aplicable al día siguiente de la finalización del PAMA, tal como lo establece el Artículo 47 del Decreto Ejecutivo 57 de 10 de agosto de 2004.

Artículo 40. Las fuentes fijas significativas deberán mantener dentro de sus instalaciones un registro con los resultados de las mediciones de sus emisiones. Tales registros deberán estar a disposición de las autoridades competentes, ya sea para cuando realicen inspecciones y/o cuando lo soliciten. El registro deberá contemplar la información correspondiente a los últimos cinco (5) años de medición.

Artículo 41. De demostrarse de acuerdo a los registros históricos, que alguna fuente fija de combustión no genera emisiones significativas, (por debajo del 40% del valor de la norma para los contaminantes regulados en la tabla del Artículo 5), la ANAM podrá disponer que se disminuya la frecuencia de los monitoreos de los contaminantes que cumplan con esta condición, hasta un período no mayor a dos (2) años, siempre y cuando se mantenga la misma capacidad de producción, proceso productivo, volumen de consumo de combustible y puntos de emisión.

Artículo 42. La ANAM está facultada para verificar el cumplimiento de lo establecido en el presente Decreto Ejecutivo, en cualquier momento, ya sea en forma directa o mediante la contratación de un organismo de inspección especializado. Los costos derivados de dicho análisis serán aplicados a la empresa.

Artículo 43. Para el caso de las fuentes fijas significativas, la ANAM establecerá metas anuales, bianuales y quinquenales de reducción de emisiones de los parámetros NO_x, SO_x, y partículas totales por tipo de actividad a través de Resolución Administrativa.

Artículo 44. Para el incumplimiento de las metas de reducción, la ANAM establecerá un canon a través de Resolución Administrativa, por cada tonelada adicional de contaminante que se emita de NO_x, SO_x y partículas totales.

Parágrafo. Para el caso de actividades donde no se emita alguno de estos parámetros, el canon se calculará en función de los que apliquen, sin perjuicio de que la ANAM pueda incluir otros parámetros en la lista de contaminantes regulados; para los cuales se considerará, a través de información técnica, establecer un canon.

Artículo 45. La ANAM coordinará con el MICI, para verificar la calidad del combustible que ingrese al país y mantendrá un registro actualizado de los mismos y de las industrias que lo emplean.

Artículo 46. La ANAM deberá contar con el equipo y el personal técnico capacitado para realizar las inspecciones y mediciones para verificar el cumplimiento de la presente normativa. Igualmente desarrollará programas periódicos de capacitación de su personal, de manera semestral o anual, en lo relativo a las técnicas de medición y conocimiento de la normativa vigente, para la supervisión, control y acreditación de ser necesario, de otras instituciones públicas o privadas. Cualquier otra institución estatal o empresa privada que desee brindar el servicio deberá contar con el equipo y personal debidamente capacitado para cumplir con los métodos y requisitos establecidos en la presente norma y deberán estar debidamente acreditados o autorizados por la Autoridad Competente.

Artículo 47. De detectarse el incumplimiento del presente Decreto Ejecutivo, será potestad de la ANAM, la realización de un monitoreo adicional de verificación, y los costos derivados del mismo se aplicarán a la empresa emisora.

Artículo 48. Las quejas o denuncias referentes a la afectación de la salud debido a las emisiones de las fuentes fijas de combustión, serán atendidas de manera expedita. En dichos casos, el MINSA solicitará a la ANAM, la revisión del grado de cumplimiento de las emisiones de la fuente fija denunciada y en caso de comprobarse cualquier anomalía, esta última podrá solicitar a costo de la empresa las mediciones y análisis necesarios.

Artículo 49. La participación de un organismo de inspección o ente fiscalizador, ya sea un laboratorio y/o empresa especializada en la verificación del cumplimiento de la norma, estaría sujeta a la observancia de los requisitos y exigencias, que para tal efecto dicte la Autoridad Competente mediante Resolución Administrativa. En este sentido, la competencia la ejercerá la ANAM hasta tanto el Consejo Nacional de Acreditación se encuentre en disposición de regular lo relativo a los organismos de inspección en la materia.

Artículo 50. La ANAM mantendrá actualizados los registros o base de datos de las emisiones de fuentes fijas de combustión presentados en las caracterizaciones y reportes de monitoreo, y establecerá los procedimientos de mantenimiento y de control de calidad de la misma.

TÍTULO VI

DE LOS MÉTODOS Y EQUIPOS DE MEDICIÓN DE EMISIONES

DESDE FUENTES FIJAS DE COMBUSTIÓN

Artículo 51. A fin de permitir la medición de las emisiones de contaminantes presentes en las emisiones de fuentes fijas significativas de combustión, las mismas deberán contar con los siguientes requisitos técnicos mínimos:

a) Plataforma de muestreo de acuerdo a las características descritas en el método 1 de la US EPA.
b) Escalera de acceso a la plataforma de muestreo.
c) Suministro de energía eléctrica cercano a los puertos de muestreo.

Artículo 52. Los equipos, métodos y procedimientos de medición de emisiones que utilice la fuente fija significativa de combustión, ya sean para la caracterización de sus emisiones o bien para los monitoreos periódicos, así como los que realice la ANAM o el organismo de inspección autorizado para tal fin, deberán cumplir con los métodos de referencia indicados en la siguiente tabla.

Métodos de Referencia para la Medición de Emisiones de Fuentes Fijas

Fuente	Método	Tema
US EPA	1	Definición de puertos de muestreo y puntos de medición en chimeneas
US EPA	2	Determinación de la velocidad de las emisiones y del flujo volumétrico en chimeneas o ductos con tubo de Pitot estándar
US EPA	5	Determinación de emisiones de partículas
US EPA	6	Determinación de emisiones de dióxido de azufre
US EPA	6C	Determinación de emisiones de dióxido de azufre por analizador instrumental
US EPA	7	Determinación de emisiones de óxidos de nitrógeno
US EPA	7E	Determinación de emisiones de óxidos de nitrógeno por analizador instrumental

Artículo 53. Se podrán utilizar otros métodos equivalentes que proporcionen información exacta y confiable sobre las emisiones de contaminantes por fuentes fijas de combustión, siempre que sean aprobados por la ANAM mediante Resolución Administrativa, y la Dirección General de Normas y Tecnología del Ministerio de Comercio e Industrias, establezca las normas nacionales correspondientes para la medición de las emisiones de fuentes fijas de combustión.

Artículo 54. La persona natural o jurídica que realice el muestreo y análisis de las emisiones de fuentes fijas de combustión, deberá cumplir con los requisitos y procedimientos que establezca el

Consejo Nacional de Acreditación para efectuar dichas actividades respecto a la acreditación de los laboratorios de ensayo y los organismos de inspección.

Artículo 55. De utilizarse analizadores portátiles, ya sea con cualquiera de las técnicas descritas anteriormente, deberán contar con los respectivos certificados de calibración vigentes, otorgados por el fabricante de los mismos.

TÍTULO VII

PROHIBICIONES, INFRACCIONES Y SANCIONES

CAPÍTULO I

PROHIBICIONES

Artículo 56. Se prohíbe expresamente la dilución de las emisiones al aire desde una fuente fija con el fin de alcanzar el cumplimiento de la presente normativa.

Artículo 57. Se prohíbe el uso de aceites lubricantes usados como combustible en calderas, hornos y/o otros equipos de combustión, sin la debida autorización por parte de la Autoridad Competente y sin cumplir con la normativa aplicable.

CAPÍTULO II

INFRACCIONES

Artículo 58. Corresponderá a la ANAM, determinar la concurrencia de una infracción a la presente norma, considerándose como infracciones lo siguiente:

a. Son infracciones leves:

i. El incumplimiento del plazo para notificar a la ANAM de situaciones anómalas respecto a las emisiones al aire establecido en el Artículo 64 de la presente norma.

ii. El incumplimiento de cualquier otra exigencia establecida en la presente norma y que no haya sido catalogada como infracción grave o muy grave.

b. Son infracciones medias:

i. La reincidencia de una infracción leve.

ii. El incumplimiento de los monitoreos establecidos en la presente norma.

iii. El incumplimiento de los plazos de caracterización establecidos en la presente norma.

iv. Rehusar, restringir o impedir la realización de inspecciones por parte de la Autoridad Competente o el organismo de inspección que este designe.

c. Son infracciones graves:

i. La reincidencia de una infracción media.

ii. El incumplimiento de los plazos de adecuación establecidos en la presente norma.

iii. El incumplimiento de los límites máximos permisibles para fuentes fijas de combustión.

iv. La dilución de las emisiones al aire como método de cumplimiento de los límites máximos permisibles establecidos en la presente norma.

d. Son infracciones muy graves:

i. La reincidencia de una infracción grave

ii. Entregar o suministrar a las autoridades competentes información falsa

CAPITULO III

SANCIONES

Artículo 59. Sin perjuicio de las sanciones civiles, penales, y demás sanciones administrativas a que hubiere lugar, la infracción o incumplimiento por parte de la empresa emisora de las obligaciones y compromisos derivados de la presente norma, acarreará la aplicación por parte de la ANAM, de las siguientes sanciones:

a. Amonestación escrita, sólo se aplicará ante la comisión de una infracción leve.

b. Multa impuesta. El monto de la multa será de quinientos balboas (B/.500.00) hasta cien mil balboas (B/. 100,000.00), según el tipo de falta, de la siguiente forma:

i. Leve, desde quinientos balboas (B/.500.00) hasta mil quinientos balboas (B/.1,500.00).

ii. Media, desde mil quinientos un balboas (B/.1,501.00) hasta diez mil balboas (B/.10,000.00).

iii. Grave, desde diez mil un balboas (B/.10,001.00) hasta cincuenta mil alboas (B/.50,000.00).

iv. Muy Grave, desde cincuenta mil un balboas (B/.50,001.00) hasta cien mil balboas (B/.100,000.00).

c. La reincidencia de una falta establecida en el numeral anterior será sancionada con el doble de la multa inicial.

d. Suspensión temporal o definitiva de las actividades de la empresa. La suspensión y cierre temporal, procederá cuando sean susceptibles de ser corregidas las causas que han ocasionado la infracción y podrá prolongarse por el tiempo que demande la corrección de las mismas. La suspensión definitiva procederá cuando se haya impuesto una multa por cometer una infracción muy grave, si esta produciendo afectaciones a la salud y la empresa no haya procedido a hacer las correcciones conforme a los plazos estipulados por las Autoridades Competentes

Artículo 60. La ANAM podrá imponer una o varias de estas sanciones, de acuerdo a la gravedad de la infracción. La imposición de una sanción no exime del cumplimiento de la obligación que generó dicha sanción.

Artículo 61. El monto de la sanción no podrá ser menor al costo en que deja de incurrir la empresa por la no-aplicación de las medidas o acciones requeridas para reducir, eliminar o mitigar las emisiones de fuentes fijas al aire, que sobrepasen los límites máximos permisibles establecidos en la presente norma.

TÍTULO VIII

DISPOSICIÓNES FINALES

Artículo 62. Se podrán utilizar combustibles alternos que han sido previamente autorizados por el MICI, que no contravengan otras disposiciones vigentes sobre esta materia. La fuente fija, sea esta significativa o no, deberá demostrar a través del respectivo estudio técnico, que cuenta con el equipo y los procedimientos para el control de las emisiones producidas por la combustión de este tipo de combustible, con la finalidad de no afectar la calidad del aire ambiente y el cumplimiento de la normativa respectiva.

Artículo 63. Los planos y especificaciones técnicas de la instalación, incluyendo las previsiones de uso de combustibles alternos, utilizado como combustible principal, combustible auxiliar, o la combinación de ambos, estarán sujetas a las disposiciones de la normativa vigente.

Artículo 64. Toda fuente fija, sea significativa o no, deberá comunicar a la ANAM cualquier situación anómala que se presente durante la operación normal de la fuente, y en la que se produzcan emisiones de contaminantes superiores a los límites máximos permisibles establecidos en esta norma. Dicha comunicación deberá realizarse en un plazo no mayor de cinco (5) horas posteriores al incidente. De persistir esta situación anómala, la ANAM creará el Comité de Emergencia Ambiental, cuya composición y funciones están establecidas en el Artículo 33 del Decreto Ejecutivo 58 de 16 de marzo de 2000.

Parágrafo: Este requisito no aplica para las operaciones de puesta en marcha o parada de la fuente, o en el caso del período de limpieza por soplado de hollín acumulado en la fuente, siempre que estos períodos no excedan quince (15) minutos y la operación no se repita más de dos (2) veces al día. Cuando las características de los procesos y/o de los equipos de combustión justifiquen técnicamente que se requiere mayor tiempo para realizar dichas actividades, se deberá obtener la aprobación de la ANAM.

Artículo 65. Para el caso de actividades de incineración y co-incineración de desechos peligrosos, se aplicarán los límites máximos permisibles establecidos en el Decreto Ejecutivo 293 de 23 de agosto de 2004 que rige esta materia.

Artículo 66. La ANAM podrá establecer límites máximos permisibles de emisión, para sectores específicos siguiendo el procedimiento establecido en la normativa vigente.

Artículo 67. De conformidad con lo dispuesto en la Ley General de Ambiente y el Decreto Ejecutivo 58 de 16 de marzo de 2000, el presente Decreto Ejecutivo será revisada cada cinco (5) años.

Artículo 68. El presente Decreto Ejecutivo entrará en vigencia a partir de su promulgación en Gaceta Oficial.

Dado en la ciudad de Panamá, a los cuatro (4) días del mes de febrero de dos mil nueve (2009).

COMUNÍQUESE Y CÚMPLASE

MARTIN TORRIJOS ESPINO

Presidente de la República de Panamá

HÉCTOR ALEXANDER H.

Ministro de Economía y Finanzas

14
