# Supplementary figures and images for: The Association of Tobacco Control Policies and the Risk of Acute Myocardial Infarction Using Hospital Admissions Data
Source: PLoS One. 2014 Feb 10;9(2):e88784. doi: 10.1371/journal.pone.0088784 (PMC3919809; doi:10.1371/journal.pone.0088784)

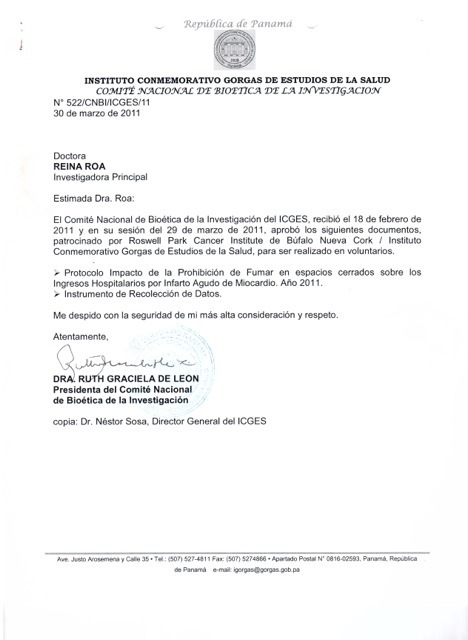

Supplement: Document S8 — IRB approval letter. (JPG) [file pone.0088784.s009.jpg]
